# Supplementary material for: Selective C(sp3)–H arylation/alkylation of alkanes enabled by paired electrocatalysis
Source: Nat Commun. 2023 Dec 2;14:7992. doi: 10.1038/s41467-023-43791-1 (PMC10693613; doi:10.1038/s41467-023-43791-1)
Supplement: Supplementary file 1 — Supplementary Information [file 41467_2023_43791_MOESM1_ESM.pdf]

Selective C(sp^3)−H Arylation/Alkylation of Alkanes Enabled by Paired Electrocatalysis

Long Zou,[1] Siqi Xiang,[1] Rui Sun[1] and Qingquan Lu[1]*

[1]The Institute for Advanced Studies (IAS), Wuhan University, Wuhan 430072, P. R. China.

gci2011@whu.edu.cn

Supplementary Information

Table of Contents

1 Supplementary Methods 3

1.1 General Experimental..........3

1.2 Description of the LED light source 3

2 Supplementary Discussion..........6

2.1 Synthesis of Starting Materials 6

2.2 Experimental Procedures..........9

2.3 Graphical Guide for C(sp^3)-H Alkenylation 11

2.4 Optimization of the Reaction Conditions 14

2.5 Scale-up Experiment 15

2.6 Unsuccessful Substrates..........16

2.7 Mechanistic Studies 16

2.8 Characterization of Products 23

3 NMR Spectra 61

4 Supplementary References..........147

1 Supplementary Methods

1.1 General Experimental.

All reactions were conducted under an argon atmosphere unless otherwise noted. All reagents, unless otherwise stated, were directly used as received from commercial suppliers (Alfa, Tci, Innochem, Aladdin, Energy Chemical, etc.) without further purification. Thin layer chromatography (TLC) employed 0.25 mm glass silica gel plates. Visualization of spots on TLC plate was accomplished with short-wave UV light, and phosphomolybdic acid, $KMnO_4$ or staining over I_2 chamber. Flash chromatography column was packed with 200-300 mesh silica gel in petroleum (bp. 60-90 °C). Proton nuclear magnetic resonance (^1H NMR) spectra and carbon nuclear magnetic resonance (^{13}C NMR) spectra were recorded on JNM ECZ 400 (400 MHz) and AVANCE NEO 600 (600 MHz) spectrometers. All chemical shifts (δ) were reported in ppm and coupling constants (J) in Hz. All Chemical shifts are reported in parts per million downfield from tetramethylsilane and are referenced to residual undeuterated solvent ($CHCl_3$ at 7.26 ppm ^1H NMR, 77.16 ppm ^{13}C NMR,). Data are represented as follows: chemical shift, multiplicity (br = broad, s = singlet, d = doublet, t = triplet, q = quartet, m = multiplet). All compounds were characterized by high resolution mass spectra (HRMS) (Bruker UltiMate3000 & Compact). Gas chromatography-mass spectrometry (GC-MS) were recorded on an Agilent 8890-5977B MSD Series spectrometer. Gas chromatography (GC) were recorded on an Agilent 8890 MSD Series spectrometer. Cyclic voltammetry studies were carried out on a CHI660E potentiostat. A glassy carbon disk, Pt wire, and Ag/AgCl (in saturated potassium chloride) were used as the working, counter, and reference electrodes, respectively.

1.2 Description of the LED light source.

Two kinds of light source appeared in this work. Each light intensity was measured with an energy spectrometer (Photocatalytic energy spectrometer, Model WATTCAS PCS230850). The 20 W 393.1 nm LED light (emitting area: 23 × 23 mm) was assembled using twenty 393.1 nm chips in a compact fashion. The emitting wavelength

of 20 W LED (393.1 nm), 20 W LED (396.4 nm) laser were recorded in Figure S1-S2.

Note: The photocatalytic energy spectrometer was placed vertically directly above the light source, and the light source continued to radiate for three minutes. Meanwhile, fan was enforced for effective thermal management to maintain luminous efficiency and life expectancy of LED light.

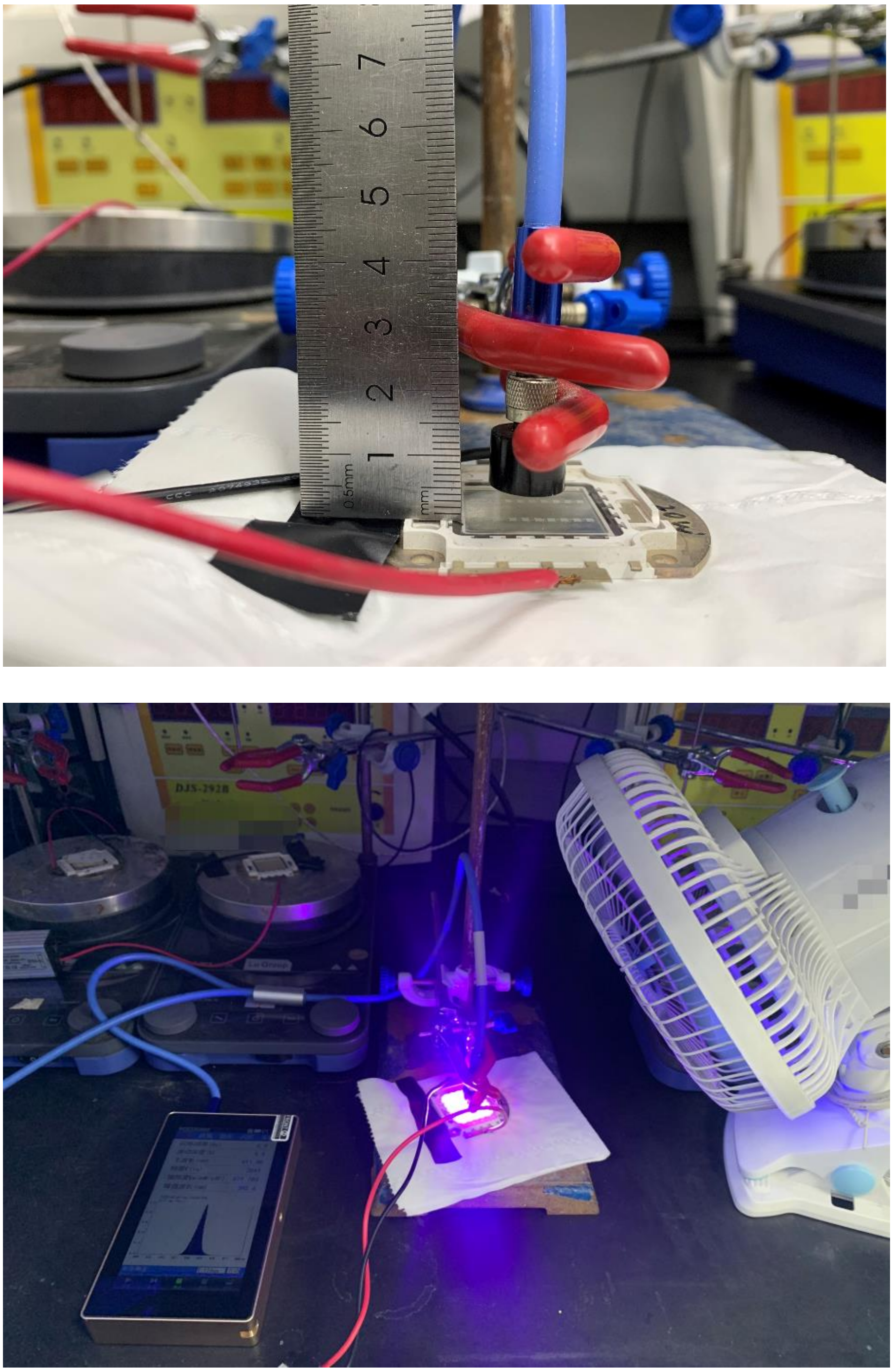

Supplementary Figure 1. Each light intensity was measured with an energy spectrometer

Distance (cm)	Illuminance (lx)	Ee (mW/cm^2)	Peak wavelength (nm)
0.5	1861	867.752	393.1

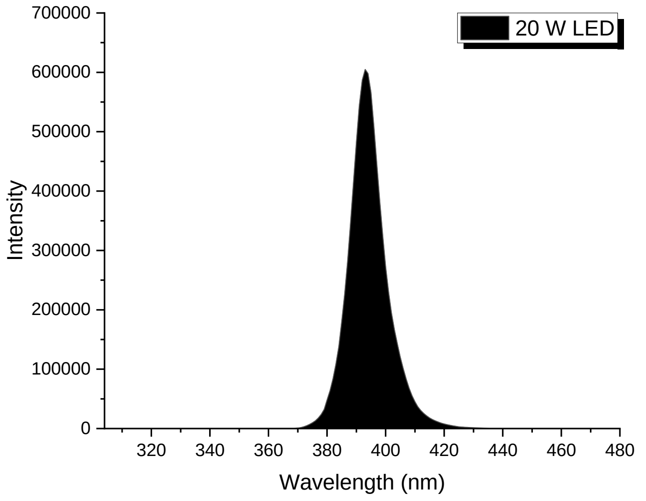

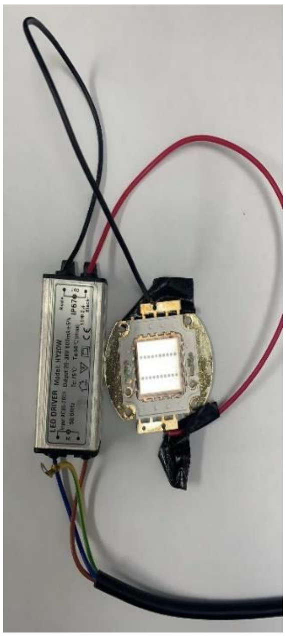

Supplementary Figure 2. The emission spectrum of the 20 W LED (393.1 nm) light.

Distance (cm)	Illuminance (lx)	Ee (mW/cm^2)	Peak wavelength (nm)
0.5	1556.8	495.351	396.4

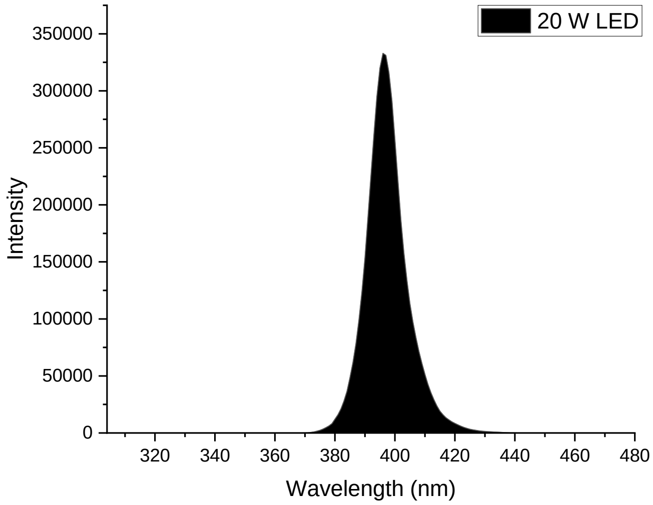

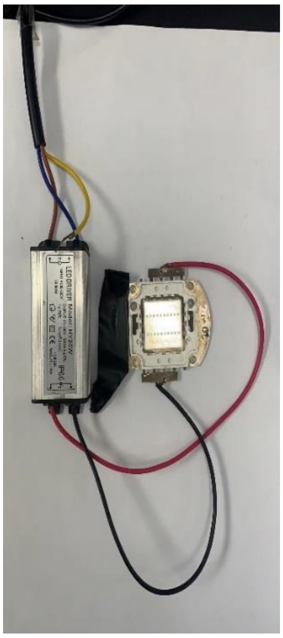

Supplementary Figure 3. The emission spectrum of the 20 W LED (395-400 nm, λ_{max} = 396.4 nm) light.

2 Supplementary Discussion

2.1 Synthesis of Starting Materials

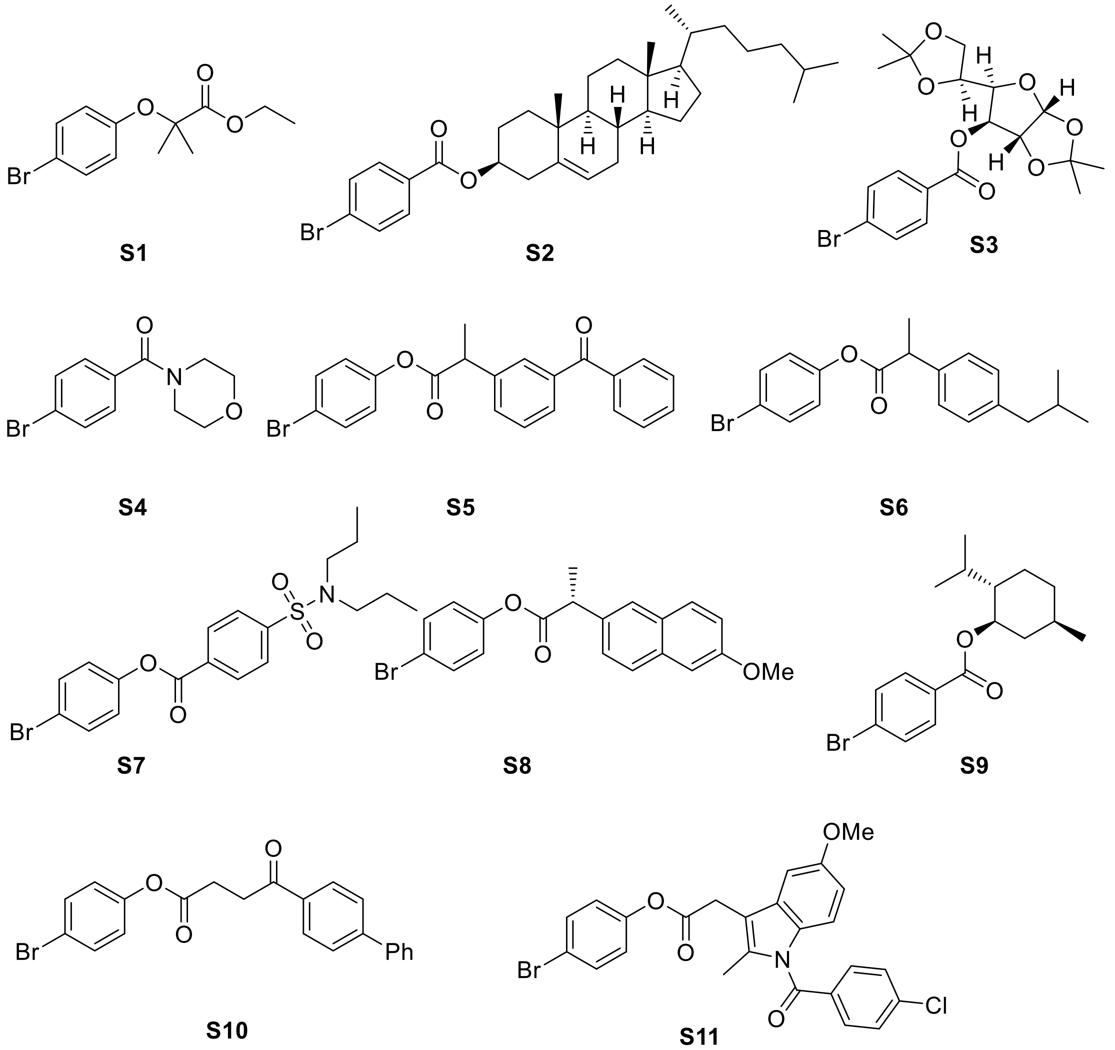

Supplementary Figure 4. Substrates **S1**[1], **S2**[2], **S3**[2], **S4**[3], **S9**[2], **S11**[4] were prepared according to reports in the literature.

General procedure A for synthesis of S5 – S8, S10[5]**.**

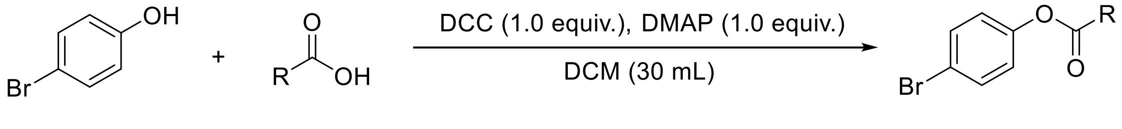

Supplementary Figure 5. General procedure A for synthesis of starting materials.

A flask was charged with 4-bromophenol (1.838 g, 10.63 mmol, 1.25 equiv.), the corresponding acid (10.63 mmol, 1.25 equiv.) and 4-dimethylaminopyridine (1.038 g, 8.50 mmol, 1.0 equiv.) and then vacuum filled under argon. The solids were then

suspended in CH_2Cl_2 (15 mL), and the reaction flask was placed in an ice/water bath. Dicyclohexylcarbodiimide (1.754 g, 8.50 mmol, 1.0 equiv.) in CH_2Cl_2 (15 mL) was then added dropwise to the reaction flask. After stirring for 4 h, the reaction was filtered through a pad of celite, which was washed with additional CH_2Cl_2 (20 mL). The organic solution was then washed with brine, dried over Na_2SO_4, and the solvent was removed via rotary evaporation. The crude solid was purified via silica gel chromatography to yield the desired product.

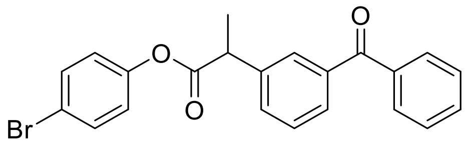

4-Bromophenyl (*R*)-2-(6-methoxynaphthalen-2-yl)propanoate (S5):

This compound was prepared according to general procedure A from 4-bromophenol (1.838 g, 10.63 mmol, 1.0 equiv.), purification by silica gel column chromatography (petroleum ether/ethyl acetate) afforded **S5** (3.87 g, 89%) as white solid. **^{1}H NMR (600 MHz, Chloroform-*d*)** δ 7.85 (s, 1H), 7.80 (dd, *J* = 8.2, 1.4 Hz, 2H), 7.72 (d, *J* = 7.8 Hz, 1H), 7.62 – 7.56 (m, 2H), 7.49 – 7.43 (m, 5H), 6.90 (d, *J* = 8.8 Hz, 2H), 4.03 (q, *J* = 7.0 Hz, 1H), 1.64 (d, *J* = 7.2 Hz, 3H). **^{13}C NMR (151 MHz, Chloroform-*d*)** δ 196.4, 172.3, 149.7, 140.2, 138.2, 137.4, 132.7, 132.5, 131.5, 130.1, 129.4, 129.2, 128.9, 128.4, 123.2, 119.0, 45.5, 18.5. **HRMS (ESI)** m/z calcd. for $C_{22}H_{18}BrO_3$ ($[M+H]^+$): 409.0434, found: 409.0433.

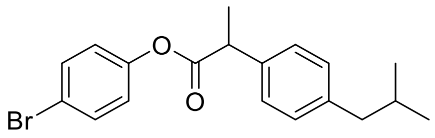

4-Bromophenyl 2-(4-isobutylphenyl)propanoate (S6):

This compound was prepared according to general procedure A from 4-bromophenol (1.838 g, 10.63 mmol, 1.0 equiv.), purification by silica gel column chromatography (petroleum ether/ethyl acetate) afforded **S6** (3.19 g, 83%) as white solid. **^{1}H NMR (600 MHz, Chloroform-*d*)** δ 7.35 (d, *J* = 8.8 Hz, 2H), 7.19 (d, *J* = 7.8 Hz, 2H), 7.06 (d, *J* =

7.8 Hz, 2H), 6.80 (d, J = 8.8 Hz, 2H), 3.83 (q, J = 7.0 Hz, 1H), 2.39 (d, J = 7.2 Hz, 2H), 1.82 – 1.75 (m, 1H), 1.51 (d, J = 7.2 Hz, 3H), 0.83 (d, J = 6.6 Hz, 6H). **^{13}C NMR (151 MHz, Chloroform-*d*)** δ 173.0, 150.0, 141.0, 137.1, 132.5, 129.7, 127.3, 123.4, 118.9, 45.3, 45.2, 30.3, 22.5, 18.6. **HRMS (ESI)** m/z calcd. for $C_{19}H_{22}BrO_2$ ($[M+H]^+$): 361.0798, found: 361.0798.

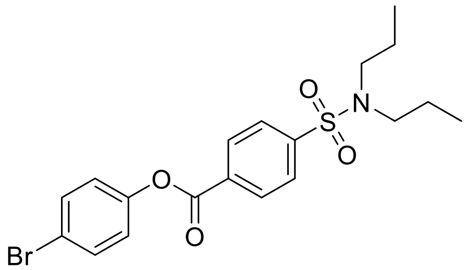

4-Bromophenyl 4-(*N,N*-dipropylsulfamoyl)benzoate (S7):

This compound was prepared according to general procedure A from 4-bromophenol (1.838 g, 10.63 mmol, 1.0 equiv.), purification by silica gel column chromatography (petroleum ether/ethyl acetate) afforded **S7** (4.03 g, 85%) as white solid. **^{1}H NMR (600 MHz, Chloroform-*d*)** δ 8.30 (d, J = 8.4 Hz, 2H), 7.94 (d, J = 8.4 Hz, 2H), 7.56 (d, J = 8.8 Hz, 2H), 7.12 (d, J = 8.8 Hz, 2H), 3.14 – 3.11 (m, 4H), 1.59 – 1.53 (m, 4H), 0.88 (t, J = 7.4 Hz, 6H). **^{13}C NMR (151 MHz, Chloroform-*d*)** δ 163.7, 149.8, 145.3, 132.8, 132.6, 131.0, 127.3, 123.5, 119.5, 50.0, 22.0, 11.3. **HRMS (ESI)** m/z calcd. for $C_{19}H_{23}BrNO_4S$($[M+H]^+$): 440.0526, found: 440.0527.

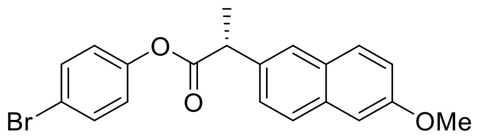

4-Bromophenyl (*R*)-2-(6-methoxynaphthalen-2-yl)propanoate (S8):

This compound was prepared according to general procedure A from 4-bromophenol (1.838 g, 10.63 mmol, 1.0 equiv.), purification by silica gel column chromatography (petroleum ether/ethyl acetate) afforded **S8** (3.24 g, 79%) as white solid. **^{1}H NMR (600 MHz, Chloroform-*d*)** δ 7.78 – 7.75 (m, 3H), 7.50 (dd, J = 8.6, 1.8 Hz, 1H), 7.44 (d, J = 8.8 Hz, 2H), 7.19 (dd, J = 8.8, 2.4 Hz, 1H), 7.16 (d, J = 2.4 Hz, 1H), 6.89 (d, J = 8.8

Hz, 2H), 4.10 (q, J = 7.2 Hz, 1H), 3.93 (s, 3H), 1.71 (d, J = 7.0 Hz, 3H). **^{13}C NMR (151 MHz, Chloroform-*d*)** δ 173.0, 157.9, 149.9, 135.0, 134.0, 132.5, 129.4, 129.1, 127.6, 126.3, 126.1, 123.3, 119.3, 118.9, 105.7, 55.4, 45.6, 18.6. **HRMS (ESI)** m/z calcd. for $C_{20}H_{18}BrO_3$ ([M+H]$^+$): 385.0434, found: 385.0433.

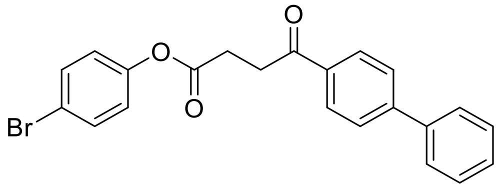

4-Bromophenyl 4-([1,1'-biphenyl]-4-yl)-4-oxobutanoate (S10):

This compound was prepared according to general procedure A from 4-bromophenol (1.838 g, 10.63 mmol, 1.0 equiv.), purification by silica gel column chromatography (petroleum ether/ethyl acetate) afforded **S10** (4.00 g, 92%) as white solid. **^{1}H NMR (600 MHz, Chloroform-*d*)** δ 8.08 (d, J = 8.4 Hz, 2H), 7.70 (d, J = 8.4 Hz, 2H), 7.63 (d, J = 7.0 Hz, 2H), 7.49 – 7.46 (m, 4H), 7.42 – 7.39 (m, 1H), 7.03 (d, J = 8.8 Hz, 2H), 3.45 (t, J = 6.4 Hz, 2H), 3.02 (t, J = 6.4 Hz, 2H). **^{13}C NMR (151 MHz, Chloroform-*d*)** δ 197.5, 171.4, 149.9, 146.2, 139.9, 135.2, 132.5, 129.1, 128.8, 128.4, 127.4(4), 127.3(9), 123.5, 119.0, 33.5, 28.6. **HRMS (ESI)** m/z calcd. for $C_{22}H_{18}BrO_3$([M+H]$^+$): 409.0434, found: 409.0435.

2.2 Experimental Procedures

General Procedure B:

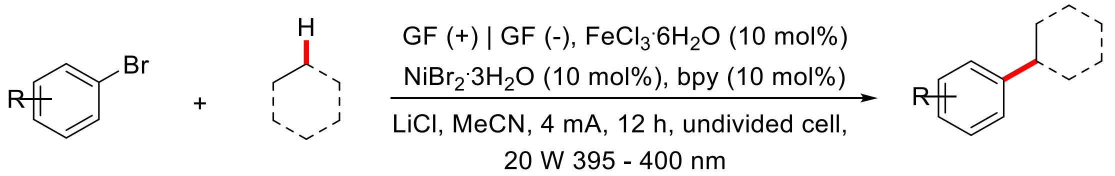

Supplementary Figure 6. General procedure B for synthesis of C(sp^3)−H arylation.

In an oven-dried three-necked cell (20 mL) equipped with a Teflon-coated magnetic stir bar and two graphite felt electrodes (20 mm × 14 mm × 2.5 mm), lithium chloride (25.4 mg, 0.6 mmol, 2.0 equiv.) and $FeCl_3$•$6H_2O$ (8.1 mg, 10 mol%) were added in a glovebox. The reaction cell was sealed and moved out from the glovebox. Afterwards,

pre-catalyst solution (it was prepared by a mix of $NiBr_2$•$3H_2O$ (8.2 mg, 10 mol%), 2,2'-bipyridine (4.7 mg, 10 mol%) in anhydrous MeCN (6.0 mL) under argon atmosphere, and was stirred for 10 minutes.), aryl bromides (0.3 mmol, 1.0 equiv.) and alkanes (3 mmol, 10.0 equiv.) were added to the reaction cell via syringe. The reaction mixture was pre-stirred for 10 minutes and was electrolyzed at a constant current of 4 mA under irradiation by a 20 W purple LED lamp (0.5 cm away, with cooling fan to keep the reaction temperature at 25 ºC) for 12 h. After the reaction, the reaction mixture was concentrated (the residual product on electrodes were rinsed with EtOAc), and purified by column chromatography (eluted with ethyl acetate/petroleum ether) to afford the pure product.

General Procedure C:

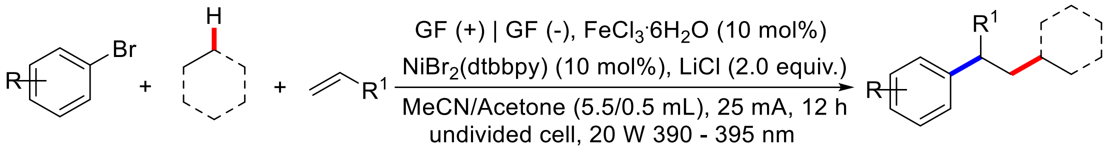

Supplementary Figure 7. General procedure C for synthesis of C(sp^3)–H alkylation.

In an oven-dried three-necked cell (20 mL) equipped with a Teflon-coated magnetic stir bar and two graphite felt electrodes (20 mm × 14 mm × 2.5 mm), lithium chloride (25.4 mg, 0.6 mmol, 2.0 equiv.), $FeCl_3$•$6H_2O$ (8.1 mg, 10 mol%) and $NiBr_2$(dtbbpy) (14.6 mg, 10 mol%) were added in a glovebox. The reaction cell was sealed and moved out from the glovebox. Afterwards, aryl bromides (0.3 mmol, 1.0 equiv.), alkanes (3 mmol, 10.0 equiv.), alkenes (0.6 mmol, 2.0 equiv.), dry MeCN (5.5 mL) and acetone (0.5 mL) were added to the reaction cell via syringe. The reaction mixture was pre-stirred for 10 minutes and was electrolyzed at a constant current of 25 mA under irradiation by a 20 W purple LED lamp (0.5 cm away, with cooling fan to keep the reaction temperature at 25 ºC) for 12 h. After the reaction, the reaction mixture was concentrated (the residual product on electrodes were rinsed with EtOAc), and purified by column chromatography (eluted with ethyl acetate/petroleum ether) to afford the pure product.

2.3 Graphical Guide for C(sp^3)-H Arylation/Alkylation

Materials used for set-up：

Graphite felt was purchased from Inner Mongolia Wanxing Carbon Co., Ltd. (http://wxcarbon.chemcp.com, Product Model: SMZ5MM). Electrode holder was purchased from Wuhan Gaossunion Technology Co., Ltd (www.gaossunion.com, Product Model: pt-3). All undivided cells were custom made by the Wuhan Ruiboer Technology Co., Ltd.

Supplementary Figure 8. Materials used for three-necked cell.

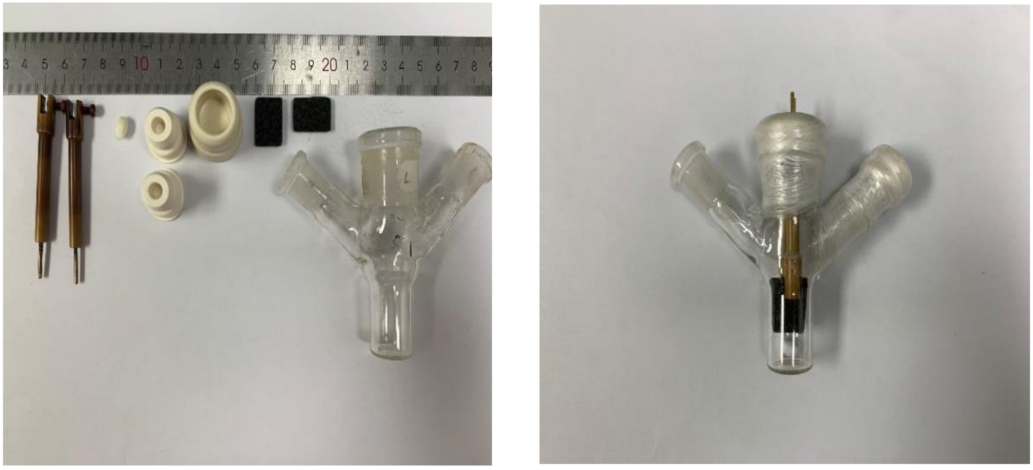

Supplementary Figure 9. Reagents used for reaction: LiCl, $FeCl_3•6H_2O$, $NiBr_2•3H_2O$, 2,2'-bipyridine, anhydrous MeCN and the substrates (such as cyclohexane, 4-bromobenzotrifluoride).

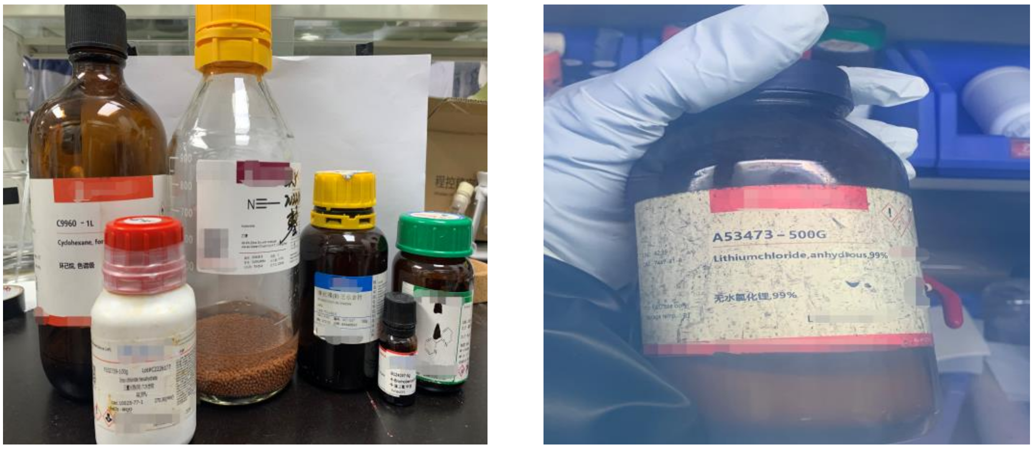

Supplementary Figure 10. $FeCl_3•6H_2O$ (8.1 mg, 10 mol%) and LiCl (25.4 mg, 0.6 mmol, 2.0 equiv.) were added to the oven-dried three-necked cell equipped with a Teflon-coated magnetic stir bar in the glovebox. $NiBr_2•3H_2O$ (8.2 mg, 10 mol%) and

2,2'-bipyridine (4.7 mg, 10 mol%) were added to a PE tube for the preparation of pre-catalyst solution.

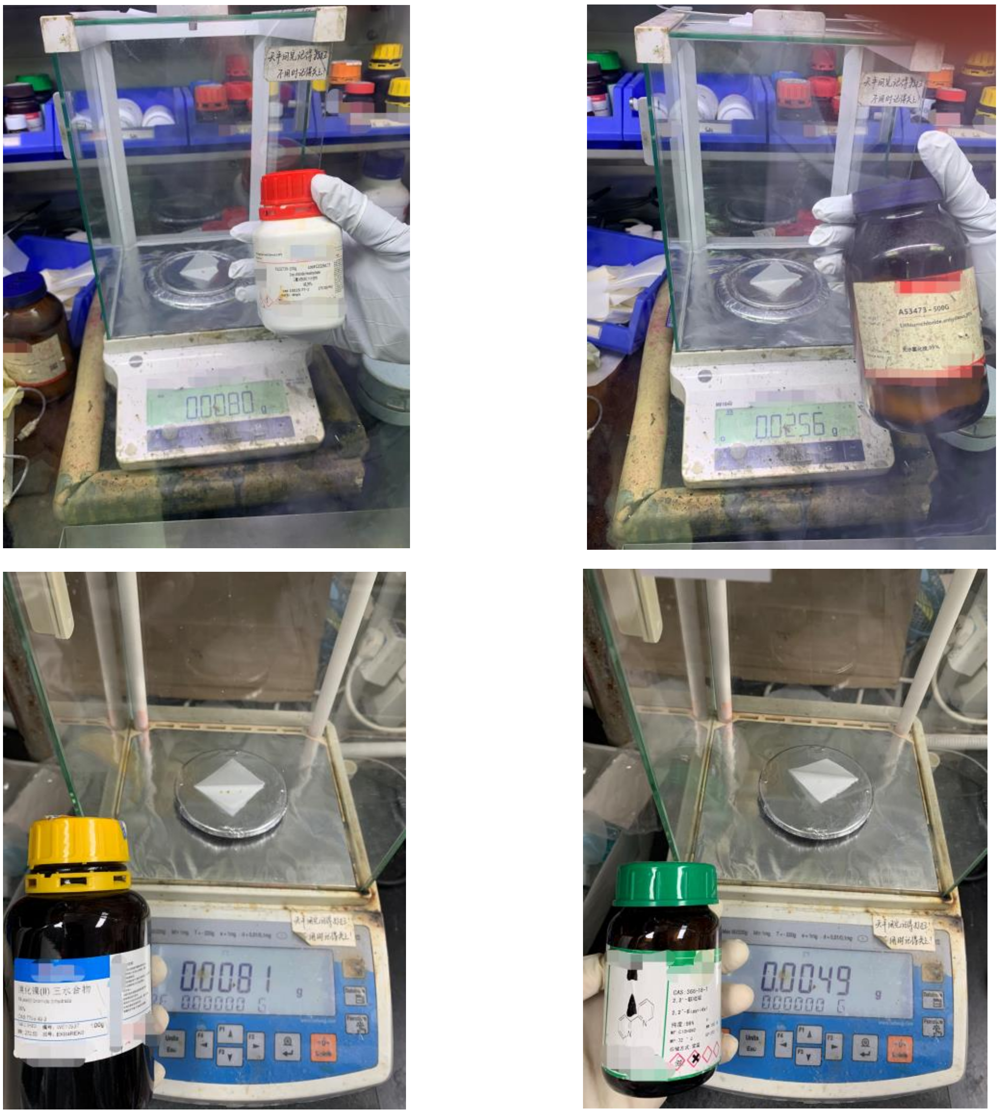

Supplementary Figure 11. The PE tube was sealed and purged with argon. Then, anhydrous acetonitrile (6.0 mL) was added to the PE tube and the pre-catalyst solution was stirred for 10 minutes. Afterwards, pre-catalyst solution, aryl bromides (0.3 mmol, 1.0 equiv.) and alkanes (3 mmol, 10.0 equiv.) were added to the reaction cell via syringe. The reaction mixture was pre-stirred for 10 minutes.

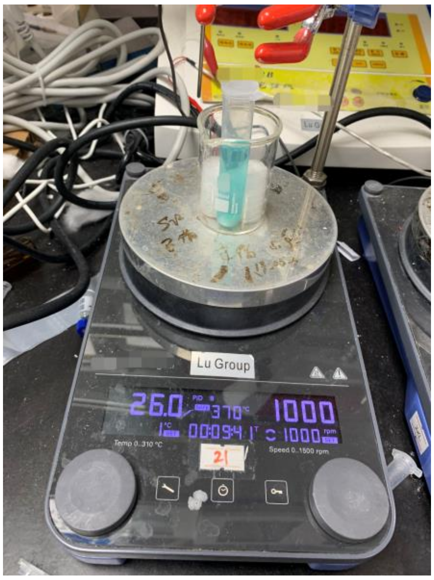

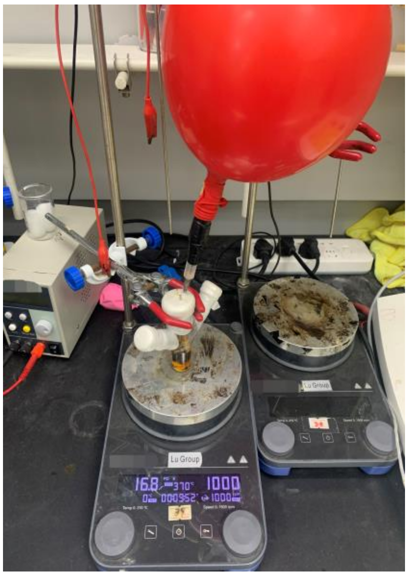

Supplementary Figure 12. The reaction mixture was electrolyzed at a constant current of 4 mA and irradiated with a 20 W purple LED lamp (0.5 cm away, with cooling fan to keep the reaction temperature at 25 ºC) for 12 h.

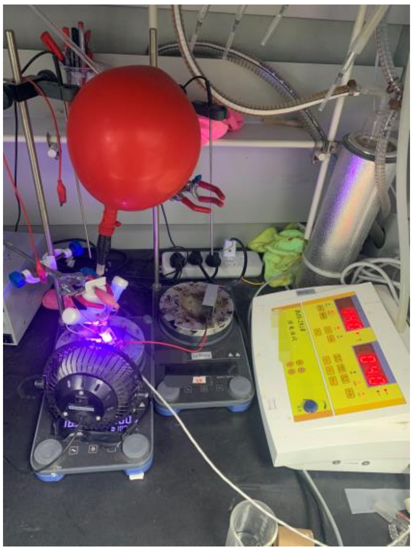

After the reaction, the reaction mixture was concentrated (the residual product on electrodes were rinsed with EtOAc), and purified by column chromatography (eluted with ethyl acetate/petroleum ether) to afford the pure product.

2.4 Optimization of conditions

Supplementary Table 1. Optimization of the reaction conditions.

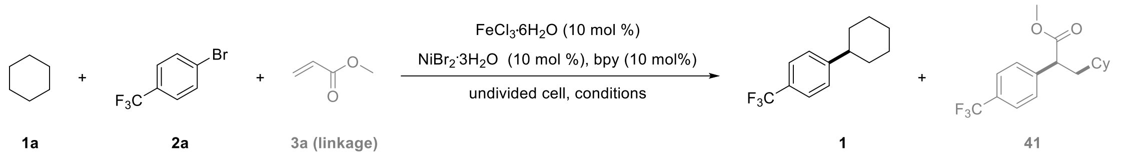

entry	electrodes	linkage	light source	current	electrolyte	solvent	yield (%) / **1** / **41**[b]
1	(+)GF/(-)GF	w/o	20 W 395-400 nm	4 mA	Bu_4NBF_4	MeCN	48/-
2	(+)GF/(-)GF	w/o	20 W 395-400 nm	4 mA	Et_4NCl	MeCN	57/-
3	(+)GF/(-)GF	w/o	20 W 395-400 nm	4 mA	LiCl	MeCN	91/-
4	(+)GF/(-)GF	w/o	30 W 395-400 nm	4 mA	LiCl	MeCN	79/-
5	(+)GF/(-)GF	w/o	10 W 395-400 nm	4 mA	LiCl	MeCN	17/-
6	(+)GF/(-)Pt	w/o	20 W 395-400 nm	4 mA	LiCl	MeCN	69/-
7	(+)C-rod/(-)GF	w/o	20 W 395-400 nm	4 mA	LiCl	MeCN	27/-
8[c]	(+)GF/(-)GF	w/o	20 W 395-400 nm	4 mA	LiCl	MeCN	86/-
9	(+)GF/(-)GF	w/o	20 W 395-400 nm	10 mA	LiCl	MeCN	56/-
10	(+)GF/(-)GF	w	20 W 395-400 nm	4 mA	LiCl	MeCN	41/22
11[d]	(+)GF/(-)GF	w	20 W 395-400 nm	25 mA	LiCl	MeCN	11/59
12[d]	(+)GF/(-)GF	w	20 W 393 nm	25 mA	LiCl	MeCN	7/82
13[d]	(+)GF/(-)GF	w	20 W 393 nm	25 mA	LiCl	MeCN/Acetone	trace/93
14[d,e]	(+)GF/(-)GF	w	20 W 393 nm	25 mA	LiCl	MeCN/Acetone	n.d./n.d.
15[e]	(+)GF/(-)GF	w/o	20 W 395-400 nm	4 mA	LiCl	MeCN	n.d./n.d.

[a]Reaction conditions: **1a** (3 mmol), **2a** (0.3 mmol), **3a** (0.6 mmol), $FeCl_3•6H_2O$ (10 mol%), $NiBr_2•3H_2O$ (10 mol%), 2,2'-Bipyridine (10 mol%), LiCl (2.0 equiv.), dry MeCN (6.0 mL), 4 mA, 12 h, 20 W 395 - 400 nm, argon, graphite felt (GF) as electrodes, undivided cell. [b]GC yields using biphenyl as an internal standard. [c]5,5'-dimethyl-2'-bipyridine

was used as ligand. [d]4,4'-di-tert-butyl-2,2'-bipyridine was used as ligand. [e]w/o paired catalyst or light or electricity. n.d. = not detected.

2.5 Scale-up Experiment.

Supplementary Figure 13. Scale-up experiment for the synthesis of product **1**.

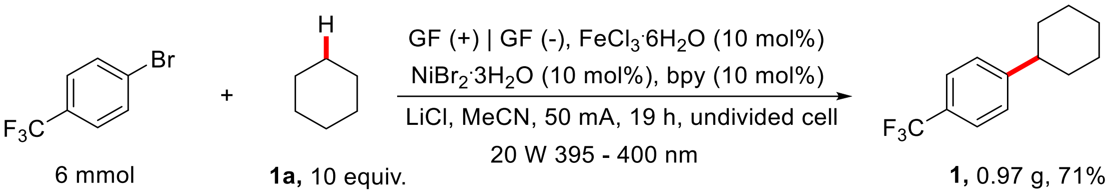

6 mmol   **1a,** 10 equiv.   **1,** 0.97 g, 71%

Supplementary Figure 14. Materials used for scale-up experiment.

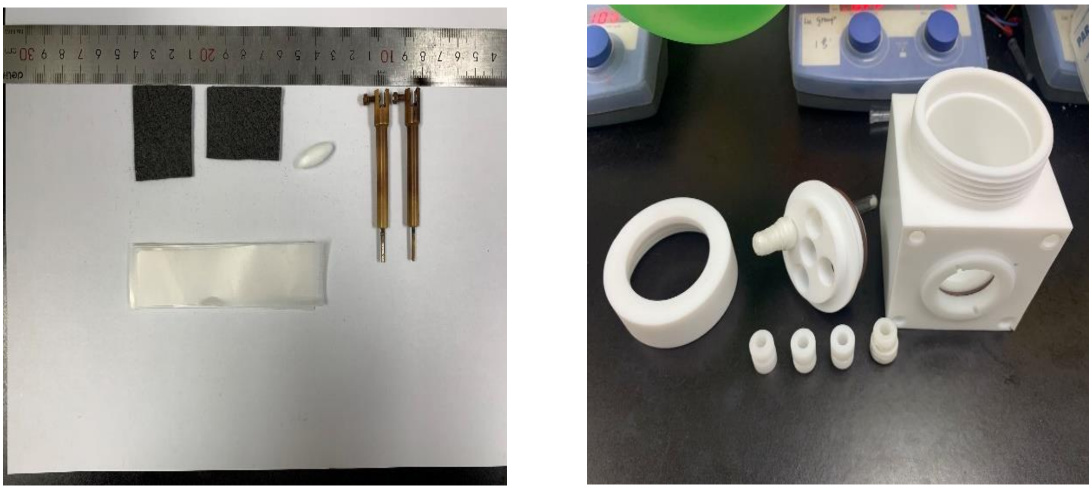

Supplementary Figure 15. Reaction was performed on 6 mmol scale according to the general procedure **B** as above described.

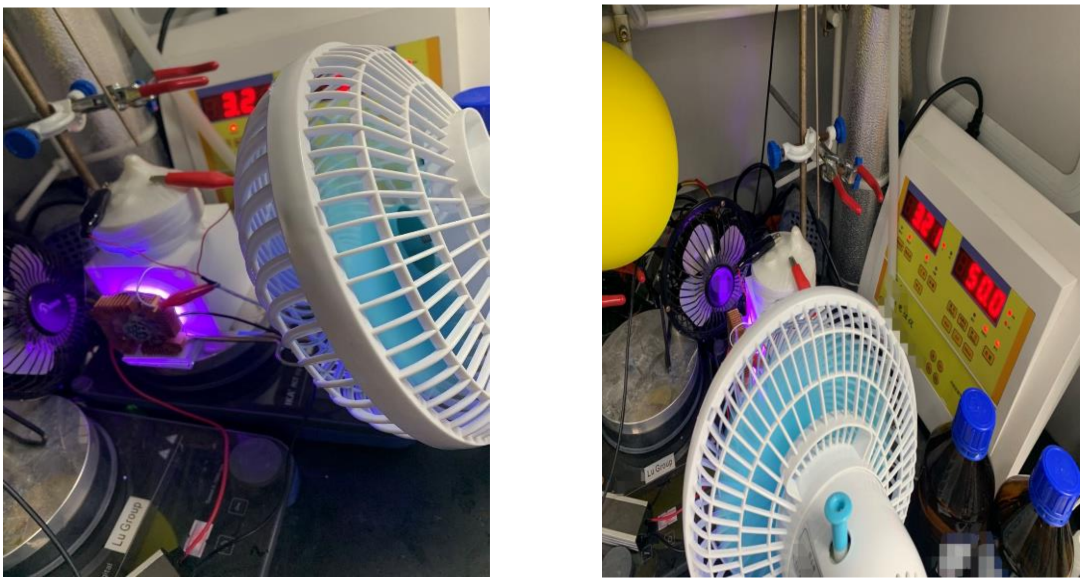

1. The reaction mixture was electrolyzed at a constant current of 50 mA and irradiated

with a 20 W purple LED lamp (0 cm away, with cooling fan to keep the reaction temperature at 25 ºC) for 19 h.

2. After the reaction, the reaction mixture was concentrated (the residual product on electrodes were rinsed with EtOAc), and purified by column chromatography (eluted with ethyl acetate/petroleum ether) to afford the pure desired product.

2.6 Unsuccessful Substrates (no product was dectected)

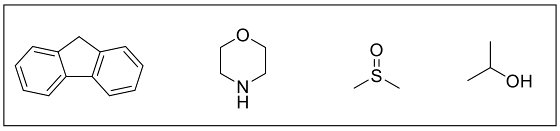

Supplementary Figure 16. Unsuccessful Substrates.

2.7 Mechanistic Studies

2.7.1 Cyclic Voltammetry Experiments

General information: Cyclic voltammetry (CV) experiments were conducted in a 20 mL two-necked cell set-up fitted with a glassy carbon working electrode (3 mm in diameter), an Ag/AgCl reference electrode, and a platinum wire counter electrode. All measurements were carried out in anhydrous MeCN, using a scan rate of 100 mV/s.

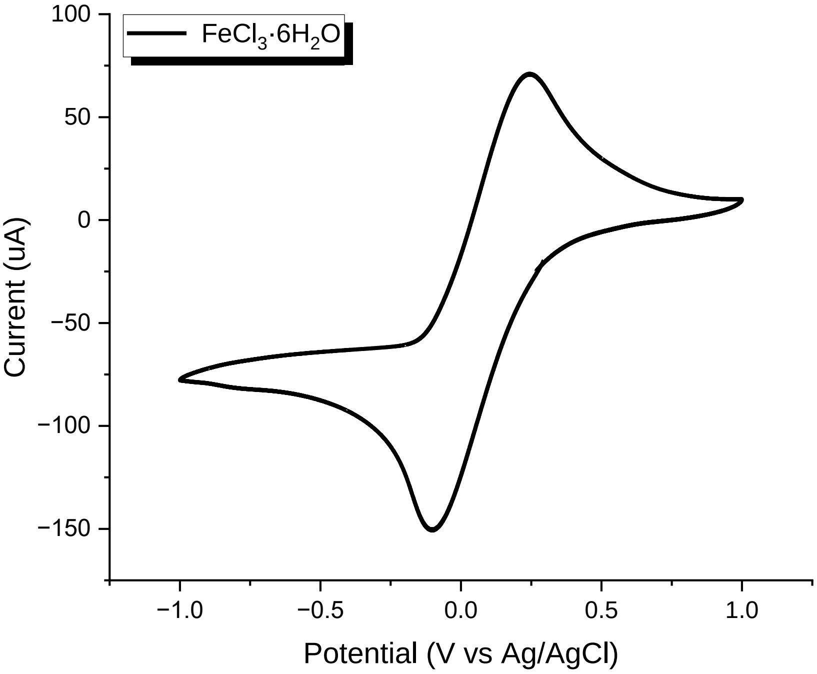

Supplementary Figure 17. Black line: $FeCl_3 \cdot 6H_2O$ (0.10 mmol), $^nBu_4NPF_6$ (0.60 mmol) in 6.0 mL MeCN.

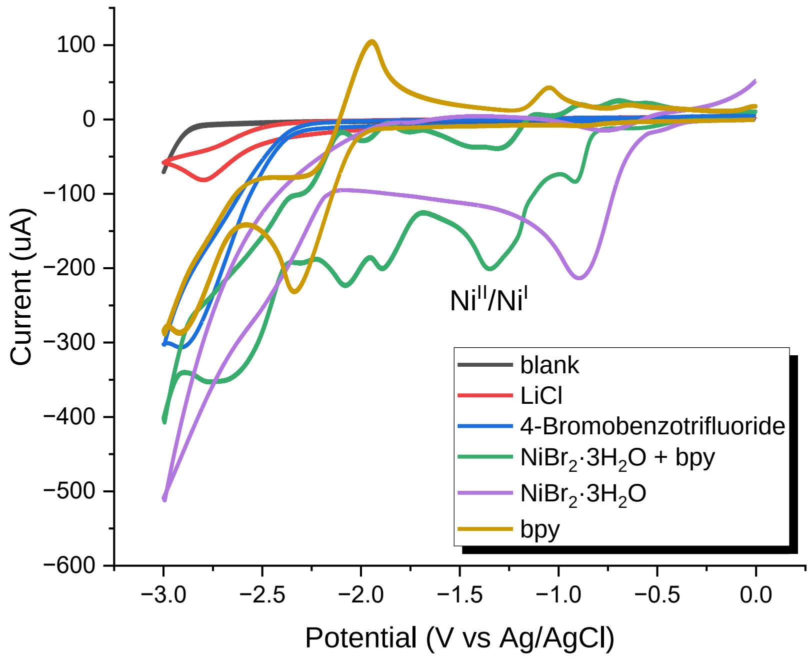

Supplementary Figure 18. Blank line: $^nBu_4NPF_6$ (0.10 mmol) in 6.0 mL MeCN. Red line: LiCl (0.05 mmol), $^nBu_4NPF_6$ (0.10 mmol) in 6.0 mL MeCN. Blue line: 4-Bromobenzotrifluoride (0.05 mmol), $^nBu_4NPF_6$ (0.10 mmol) in 6.0 mL MeCN. Green line: $NiBr_2 \cdot 3H_2O$ (0.05 mmol), bpy (0.05 mmol), $^nBu_4NPF_6$ (0.10 mmol) in 6.0 mL MeCN. Purple line: $NiBr_2 \cdot 3H_2O$ (0.05 mmol), $^nBu_4NPF_6$ (0.10 mmol) in 6.0 mL MeCN. Brown line: Bpy (0.05 mmol), $^nBu_4NPF_6$ (0.10 mmol) in 6.0 mL MeCN.

2.7.2 Radical trapping experiments.

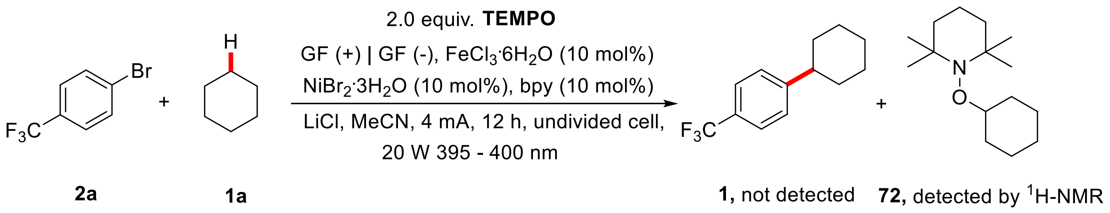

Supplementary Figure 19. Radical trapping experiment.

According to the general procedure **B**, the model reaction was carried out under standard conditions in the presence of TEMPO (93.8 mg, 0.6 mmol, 2.0 equiv.). After the reaction, the reaction mixture was analyzed by GC-MS and ^1H-NMR.

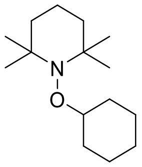

1-(Cyclohexyloxy)-2,2,6,6-tetramethylpiperidine (72)[6]: Colorless oil, **^{1}H NMR (600 MHz, $CDCl_3$)** δ 3.60 – 3.65 (m, 1H), 2.06 – 2.03 (m, 2H), 1.75 – 1.70 (m, 2H), 1.55 – 1.49 (m, 6H), 1.28 – 1.24 (m, 3H), 1.21 – 1.09 (m, 15H).

^{13}C NMR (151 MHz, $CDCl_3$) δ 81.9, 59.7, 40.4, 34.9, 33.0, 26.1, 25.2, 20.4, 17.4.

Supplementary Figure 20. The reaction mixture was analyzed by GC-MS.

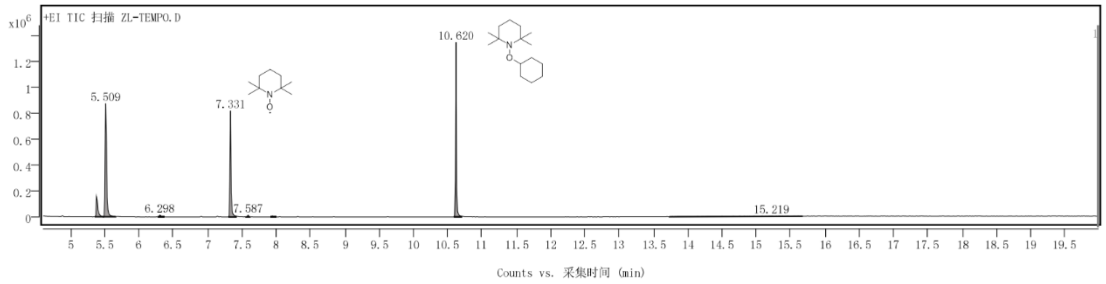

Supplementary Figure 21. Molecular ion peaks for **72.**

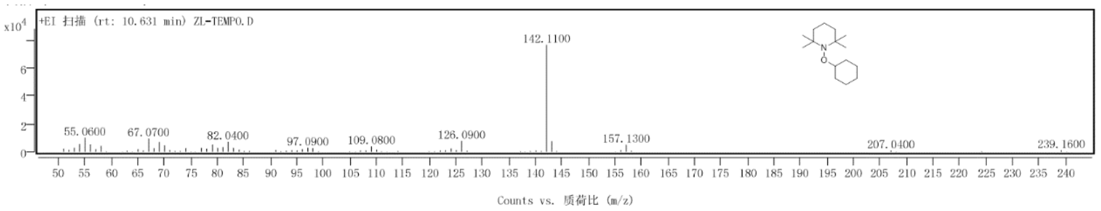

2.7.3 The stoichiometric reaction of Ni (II) aryl complex.

The oxidative addition complex **73** was prepared following literature procedures[2].

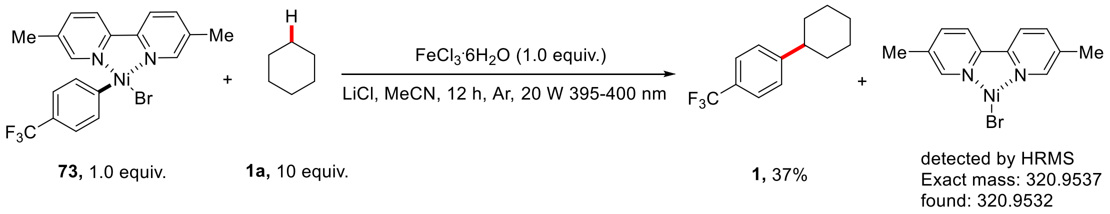

Supplementary Figure 22. The stoichiometric reaction of Ni (II) aryl complex.

To a quartz tube equipped with a Teflon-coated magnetic stir bar were added complex **73** (139.8 mg, 0.3 mmol, 1.0 equiv.), $FeCl_3$•$6H_2O$ (81.1 mg, 0.3 mmol, 1.0 equiv.), LiCl (25.4, 0.6 mmol, 2.0 equiv.), cyclohexane (10.0 equiv.), and MeCN (6.0 mL). The resulting mixture was stirred for 12 h under irradiation by LED lamp (20 W, 395 - 400

nm) with fan cooling. The reaction mixture was then analyzed by gas chromatography to obtain the yield of **1** using biphenyl as an internal standard.

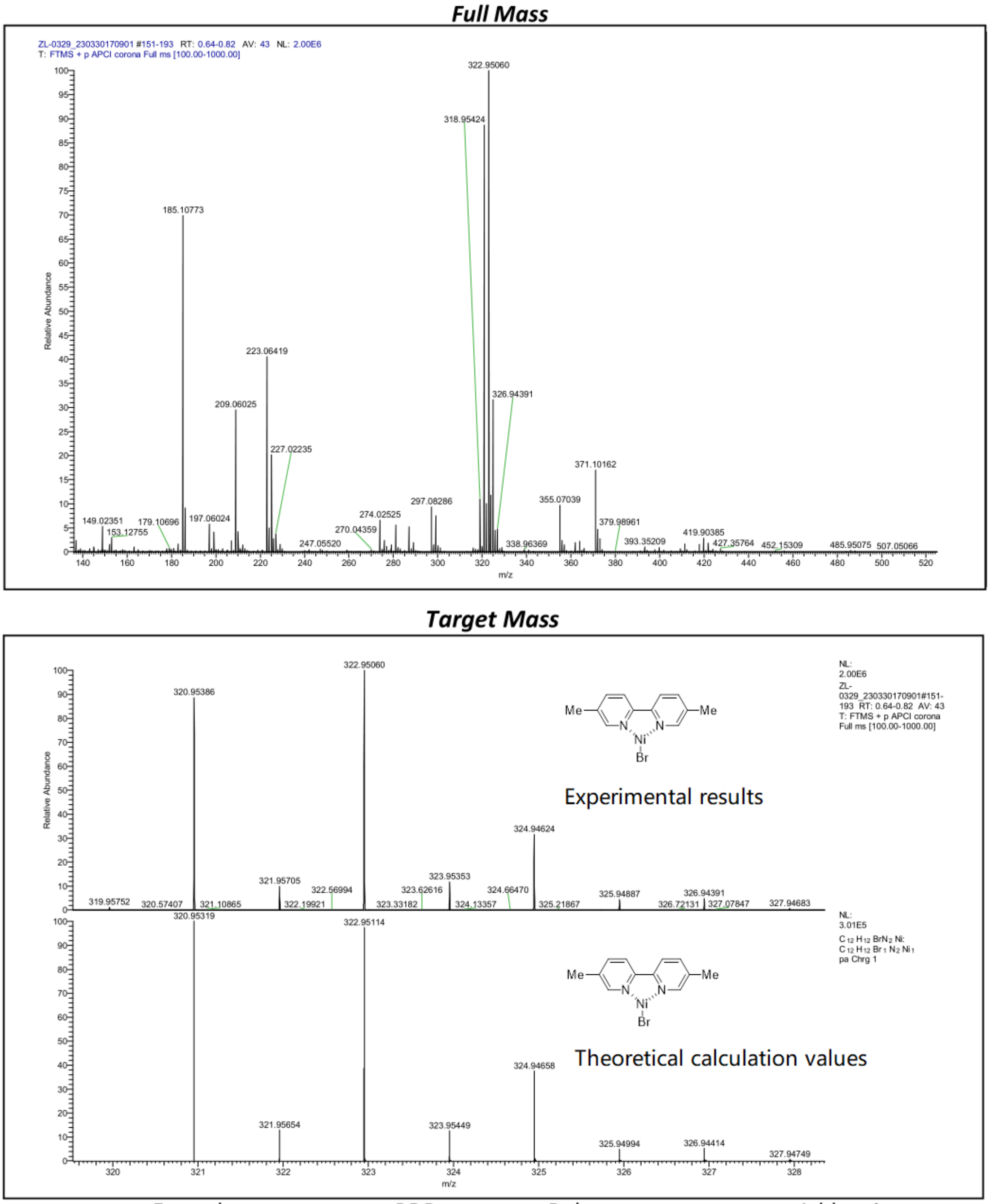

Supplementary Figure 23. The reaction mixture was analyzed by HRMS.

2.7.3.1 The catalytic reaction of Ni (II) aryl complex.

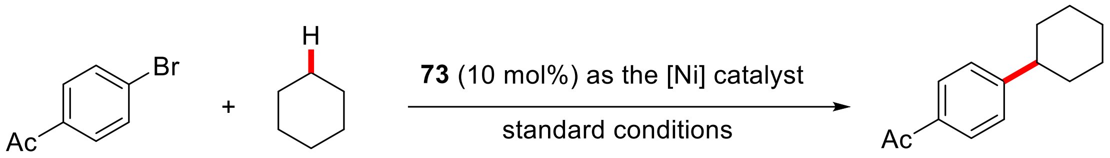

1.0 equiv. **1a,** 10 equiv. **23,** 46%

Supplementary Figure 24. The catalytic reaction of Ni (II) aryl complex.

According to the general procedure **B**, the reaction was carried out under standard conditions by employing 4'-bromoacetophenone (0.3 mmol), **73** (0.03 mmol,10 mol%) and cyclohexane **1a** (3 mmol, 10.0 equiv.) as substrates in the absence of $NiBr_2$•$3H_2O$ and 2,2'-bipyridine. The reaction mixture was then analyzed by gas chromatography to obtain the yield of **23** using biphenyl as an internal standard.

2.7.4 Constant Voltage Electrolysis

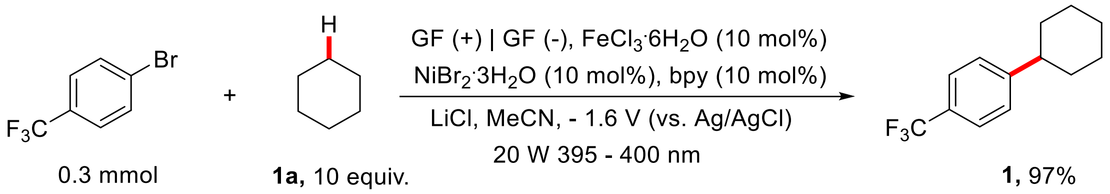

0.3 mmol **1a,** 10 equiv. **1,** 97%

Supplementary Figure 25. Constant Voltage Electrolysis.

According to the general procedure **B**, the reaction mixture was electrolyzed at a constant potential of -1.6 V (vs. Ag/AgCl). The reaction mixture was then analyzed by gas chromatography to obtain the yield of **1** using biphenyl as an internal standard.

2.7.5 The catalytic reaction of $Ni(cod)_2$.

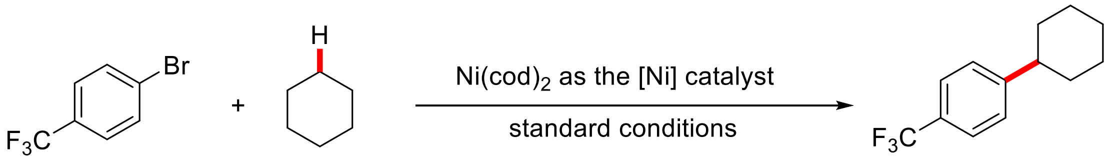

0.3 mmol **1a,** 10 equiv. **1,** 9%

Supplementary Figure 26. The catalytic reaction of $Ni(cod)_2$.

According to the general procedure **B**, the reaction was carried out under standard

conditions by employing $Ni(cod)_2$ as the [Ni] catalyst. The reaction mixture was then analyzed by gas chromatography to obtain the yield of **1** using biphenyl as an internal standard.

2.7.6 Electrode voltage over the course of electrolysis.

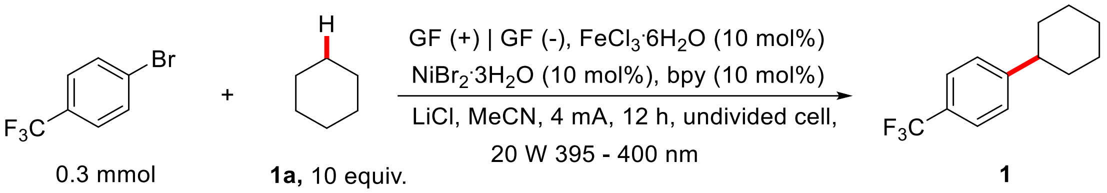

Supplementary Figure 27. Electrode voltage over the course of electrolysis.

According to the general procedure **B**, the electrode potential was monitored under standard conditions using Ag/AgCl as a reference electrode. The electrodes (graphite felts) and reference electrode were inserted into the reaction mixture together during the electrolysis. A multimeter was used to detect electrode potential between anode/cathode and reference electrode respectively (keep anode or cathode with reference electrode as close as possible when testing).

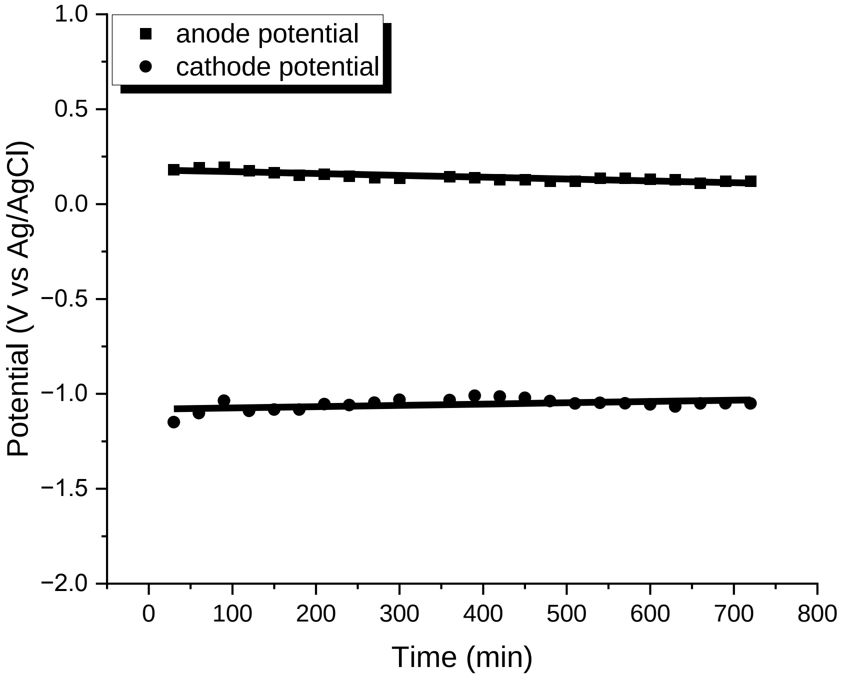

Supplementary Figure 28. Electrode voltage over the course of electrolysis.

2.7.7 Competition study of cycloheptyl chloride and cyclohexane.

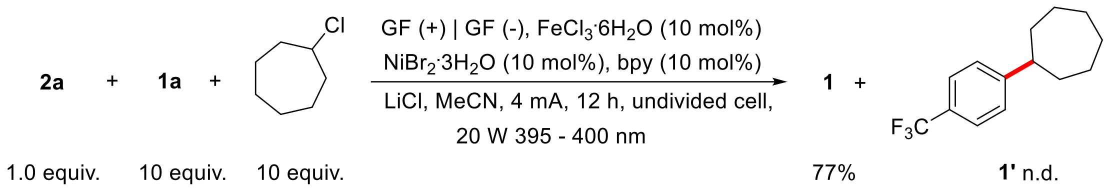

Supplementary Figure 29. Competition study of cycloheptyl chloride and cyclohexane.

According to the general procedure **B**, the reaction was carried out under standard conditions by employing 4'-bromobenzotrifluoride (0.3 mmol), cycloheptyl chloride (3 mmol, 10.0 equiv.) and cyclohexane **1a** (3 mmol, 10.0 equiv.) as substrates. The reaction mixture was then analyzed by gas chromatography to obtain the yield of **1** using biphenyl as an internal standard.

Supplementary Figure 30. The reaction mixture was analyzed by GC-MS.

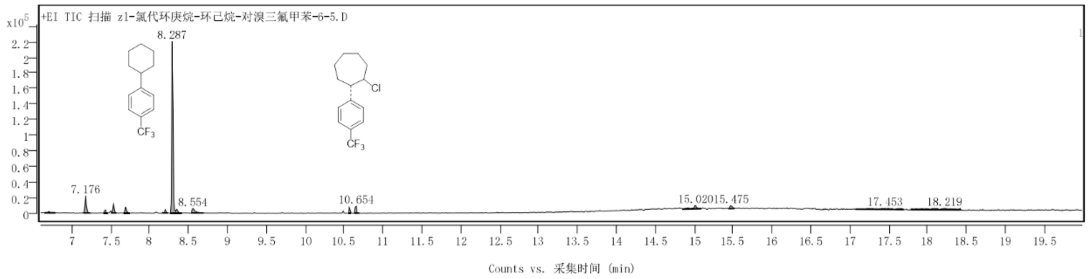

2.7.8 The reaction of *n*-pentane with 4-bromo-2-fluoropyridine.

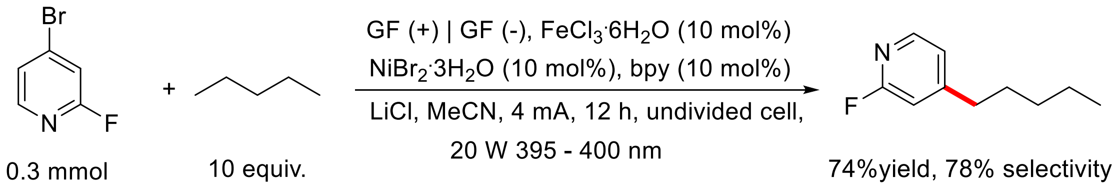

Supplementary Figure 31. The reaction of *n*-pentane with 4-bromo-2-fluoropyridine.

According to the general procedure **B**, the reaction was carried out under standard conditions by employing 4-bromo-2-fluoropyridine (0.3 mmol) and *n*-pentane (3 mmol, 10.0 equiv.) as substrates. The product with different regional selectivity was isolated in 74% yield with 78% primary C(sp^3)–H selectivity (determined by ^{19}F NMR).

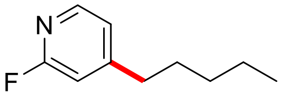

2-Fluoro-4-pentylpyridine: Colorless oil, **^{1}H NMR (400 MHz, Chloroform-*d*)** δ 8.08

(d, J = 5.0 Hz, 1H), 6.98 (d, J = 5.2 Hz, 1H), 6.73 (s, 1H), 2.62 (t, J = 7.6 Hz, 2H), 1.68 – 1.51 (m, 2H), 1.38 – 1.20 (m, 4H), 0.89 (t, J = 6.6 Hz, 3H).

^{13}C NMR (101 MHz, Chloroform-*d*) δ 164.2 (d, J = 235.6 Hz), 158.0 (d, J = 7.8 Hz), 147.2 (d, J = 14.8 Hz), 121.8 (d, J = 3.8 Hz), 109.2 (d, J = 36.6 Hz), 35.2 (d, J = 2.4 Hz), 31.1, 29.9, 22.5, 14.0.

^{19}F NMR (376 MHz, Chloroform-*d*) δ -69.41.

HRMS (ESI) m/z calcd. for $C_{10}H_{15}FN$ ($[M+H]^+$): 168.1183, found: 168.1185.

Supplementary Figure 32. The reaction mixture was analyzed by GC-MS.

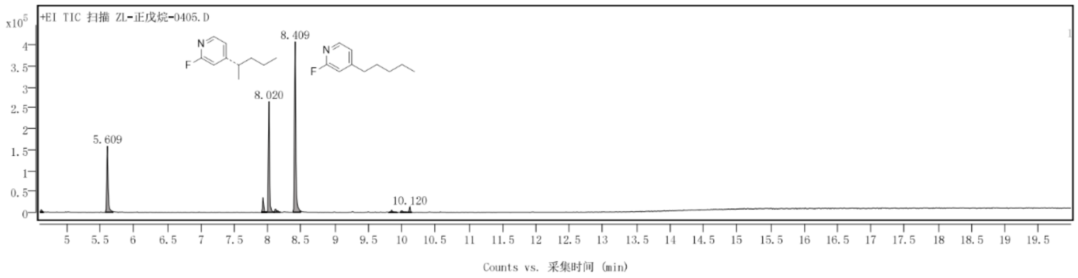

2.8 Characterization of Products

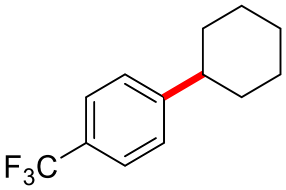

1-Cyclohexyl-4-(trifluoromethyl)benzene (1)[2]: Colorless oil was obtained with 89% isolated yield following the general procedure B (0.3 mmol scale, 60.9 mg, average yield of two times).

^{1}H NMR (600 MHz, Chloroform-*d*) δ 7.55 (d, J = 8.0 Hz, 2H), 7.32 (d, J = 8.0 Hz, 2H), 2.59 – 2.55 (m, 1H), 1.89 – 1.86 (m, 4H), 1.79 – 1.76 (m, 1H), 1.45 – 1.40 (m, 4H), 1.29 – 1.26 (m, 1H).

^{13}C NMR (151 MHz, Chloroform-*d*) δ 152.2, 128.2 (q, J = 32.2 Hz), 127.3, 125.4 (q, J = 3.8 Hz), 124.6 (q, J = 271.8 Hz), 44.7, 34.4, 26.9, 26.2.

^{19}F NMR (565 MHz, Chloroform-*d*) δ -62.25.

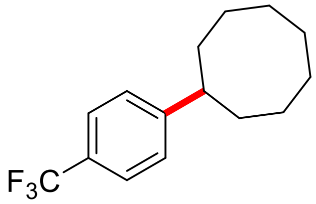

(4-(Trifluoromethyl)phenyl)cyclooctane (2): Colorless oil was obtained with 90% isolated yield following the general procedure B (0.3 mmol scale, 69.2 mg, average yield of two times).

^{1}H NMR (600 MHz, Chloroform-*d*) δ 7.53 (d, *J* = 8.0 Hz, 2H), 7.29 (d, *J* = 8.0 Hz, 2H), 2.85 – 2.81 (m, 1H), 1.86 – 1.75 (m, 6H), 1.69 – 1.67 (m, 3H), 1.63 – 1.58 (m, 5H).

^{13}C NMR (151 MHz, Chloroform-*d*) δ 154.5, 127.9 (q, *J* = 32.2 Hz), 127.4, 125.3 (q, *J* = 3.8 Hz), 124.6 (q, *J* = 272.0 Hz), 44.8, 34.5, 27.0, 26.4, 26.1.

^{19}F NMR (565 MHz, Chloroform-*d*) δ -62.20.

HRMS (APCI) m/z calcd. for $C_{15}H_{20}F_3$ ([M+H]$^+$): 257.1512, found: 257.1512.

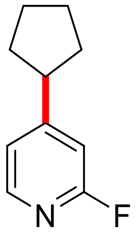

4-Cyclopentyl-2-fluoropyridine (3): Colorless oil was obtained with 84% isolated yield following the general procedure B (0.3 mmol scale, 41.6 mg, average yield of two times).

^{1}H NMR (400 MHz, Chloroform-*d*) δ 8.06 (d, *J* = 5.2 Hz, 1H), 7.01 (d, *J* = 5.0 Hz, 1H), 6.75 (s, 1H), 3.00 (p, *J* = 8.6 Hz, 1H), 2.12 – 2.07 (m, 2H), 1.84 – 1.65 (m, 4H), 1.61 – 1.52 (m, 2H).

^{13}C NMR (101 MHz, Chloroform-*d*) δ 164.2 (d, *J* = 239.0 Hz), 162.0 (d, *J* = 7.6 Hz), 147.2 (d, *J* = 15.4 Hz), 120.6 (d, *J* = 3.8 Hz), 107.8 (d, *J* = 36.8 Hz), 45.2 (d, *J* = 2.8 Hz), 33.9, 25.5.

^{19}F NMR (376 MHz, Chloroform-*d*) δ -69.35.

HRMS (ESI) m/z calcd. for $C_{10}H_{13}FN$ ([M+H]$^+$): 166.1027, found: 166.1032.

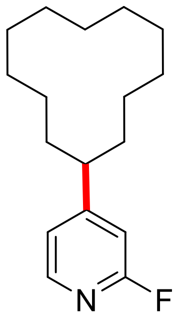

4-Cyclododecyl-2-fluoropyridine (4): White solid was obtained with 64% isolated yield following the general procedure B (0.3 mmol scale, 50.6 mg, average yield of two times).

^{1}H NMR (400 MHz, Chloroform-*d*) δ 8.07 (d, *J* = 5.2 Hz, 1H), 6.99 (d, *J* = 5.2 Hz, 1H), 6.73 (s, 1H), 2.82 – 2.76 (m, 1H), 1.82 – 1.76 (m, 2H), 1.48 – 1.27 (m, 20H).

^{13}C NMR (101 MHz, Chloroform-*d*) δ 164.2 (d, *J* = 239.2 Hz), 162.9 (d, *J* = 7.4 Hz), 147.3 (d, *J* = 15.4 Hz), 121.0 (d, *J* = 3.8 Hz), 108.4 (d, *J* = 36.6 Hz), 39.6 (d, *J* = 2.8 Hz), 30.7, 23.8, 23.7, 23.5, 23.3, 22.5.

^{19}F NMR (376 MHz, Chloroform-*d*) δ -69.21.

HRMS (ESI) m/z calcd. for $C_{17}H_{27}FN$ ($[M+H]^+$): 264.2122, found: 264.2115.

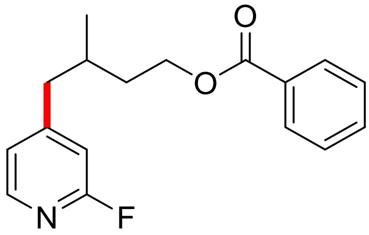

4-(2-Fluoropyridin-4-yl)-3-methylbutyl benzoate (5): Colorless oil was obtained with 87% isolated yield following the general procedure B (0.3 mmol scale, 75.0 mg, average yield of two times).

^{1}H NMR (600 MHz, Chloroform-*d*) δ 8.08 (d, *J* = 4.6 Hz, 1H), 8.01 (d, *J* = 7.2 Hz, 2H), 7.56 – 7.53 (m, 1H), 7.44 – 7.42 (m, 2H), 6.97 (d, *J* = 5.2 Hz, 1H), 6.72 (s, 1H), 4.43 – 4.39 (m, 1H), 4.37 – 4.33 (m, 1H), 2.74 (dd, *J* = 13.6, 6.0 Hz, 1H), 2.48 (dd, *J* = 13.6, 8.6 Hz, 1H), 2.03 – 1.97 (m, 1H), 1.85 – 1.80 (m, 1H), 1.68 – 1.60 (m, 1H), 0.95 (d, *J* = 6.6 Hz, 3H).

^{13}C NMR (151 MHz, Chloroform-*d*) δ 166.6, 164.1 (d, *J* = 238.4 Hz), 155.9 (d, *J* =

7.6 Hz), 147.4 (d, J = 15.2 Hz), 133.1, 130.2, 129.6, 128.5, 122.3 (d, J = 3.8 Hz), 109.9 (d, J = 36.4 Hz), 62.9, 42.6 (d, J = 2.7 Hz), 35.2, 31.4, 19.3.

^{19}F NMR (565 MHz, Chloroform-*d*) δ -69.12.

HRMS (ESI) m/z calcd. for $C_{17}H_{19}FNO_2$ ($[M+H]^+$): 288.1394, found: 288.1391.

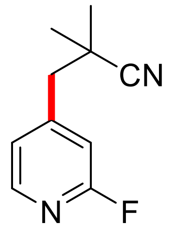

3-(2-Fluoropyridin-4-yl)-2,2-dimethylpropanenitrile (6): Yellow oil was obtained with 63% isolated yield following the general procedure B (0.3 mmol scale, 33.7 mg, average yield of two times).

^{1}H NMR (600 MHz, Chloroform-*d*) δ 8.17 (d, J = 5.0 Hz, 1H), 7.13 – 7.11 (m, 1H), 6.84 (s, 1H), 2.82 (s, 2H), 1.36 (s, 6H).

^{13}C NMR (151 MHz, Chloroform-*d*) δ 163.9 (d, J = 239.0 Hz), 150.4 (d, J = 7.8 Hz), 147.8 (d, J = 15.2 Hz), 123.8, 123.1 (d, J = 4.0 Hz), 111.0 (d, J = 37.0 Hz), 45.6 (d, J = 2.8 Hz), 33.1, 26.6.

^{19}F NMR (565 MHz, Chloroform-*d*) δ -67.92.

HRMS (ESI) m/z calcd. for $C_{10}H_{12}FN_2$ ($[M+H]^+$): 179.0979, found: 179.0975.

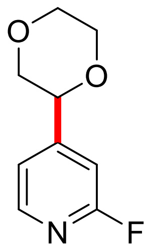

4-(1,4-Dioxan-2-yl)-2-fluoropyridine (7): Colorless oil was obtained with 63% isolated yield following the general procedure B (0.3 mmol scale, 34.6 mg, average yield of two times).

^{1}H NMR (400 MHz, Chloroform-*d*) δ 8.18 (d, J = 5.0 Hz, 1H), 7.11 (d, J = 5.0 Hz, 1H), 6.94 (s, 1H), 4.66 (dd, J = 10.0, 2.8 Hz, 1H), 3.97 – 3.85 (m, 3H), 3.81 (dd, J = 11.6, 2.6 Hz, 1H), 3.69 (td, J = 11.4, 3.2 Hz, 1H), 3.36 (t, J = 10.8 Hz, 1H).

^{13}C NMR (101 MHz, Chloroform-*d*) δ 164.1 (d, *J* = 240.2 Hz), 153.1 (d, *J* = 8.0 Hz), 147.8 (d, *J* = 15.0 Hz), 118.7, 106.9 (d, *J* = 38.6 Hz), 75.8 (d, *J* = 3.0 Hz), 71.7, 66.9, 66.4.

^{19}F NMR (376 MHz, Chloroform-*d*) δ -67.77.

HRMS (ESI) m/z calcd. for $C_9H_{11}FNO_2$ ([M+H]$^+$): 184.0768, found: 184.0766.

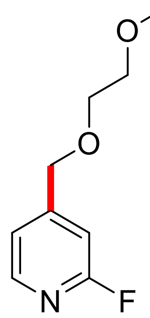

2-Fluoro-4-((2-methoxyethoxy)methyl)pyridine (8): Colorless oil was obtained with 57% isolated yield following the general procedure B (0.3 mmol scale, 31.7 mg, average yield of two times).

^{1}H NMR (400 MHz, Chloroform-*d*) δ 8.14 (d, *J* = 5.0 Hz, 1H), 7.12 (d, *J* = 5.2 Hz, 1H), 6.93 (s, 1H), 4.60 (s, 2H), 3.67 (t, *J* = 4.0 Hz, 2H), 3.58 (t, *J* = 4.0 Hz, 2H), 3.39 (s, 3H).

^{13}C NMR (101 MHz, Chloroform-*d*) δ 164.2 (d, *J* = 239.6 Hz), 153.9 (d, *J* = 7.8 Hz), 147.6 (d, *J* = 15.0 Hz), 119.4 (d, *J* = 4.0 Hz), 107.4 (d, *J* = 38.0 Hz), 72.0, 71.1 (d, *J* = 3.0 Hz), 70.4, 59.2.

^{19}F NMR (376 MHz, Chloroform-*d*) δ -68.12.

HRMS (APCI) m/z calcd. for $C_9H_{13}FNO_2$ ([M+H]$^+$): 186.0925, found: 186.0923.

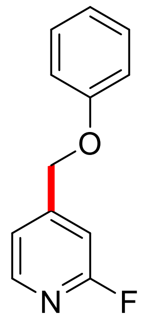

2-Fluoro-4-(phenoxymethyl)pyridine (9): Colorless oil was obtained with 66%

isolated yield following the general procedure B (0.3 mmol scale, 40.2 mg, average yield of two times).

^{1}H NMR (400 MHz, Chloroform-*d*) δ 8.21 (d, *J* = 5.0 Hz, 1H), 7.32 (t, *J* = 7.6 Hz, 2H), 7.23 (d, *J* = 5.0 Hz, 1H), 7.03 – 6.99 (m, 2H), 6.96 (d, *J* = 8.0 Hz, 2H), 5.11 (s, 2H).

^{13}C NMR (101 MHz, Chloroform-*d*) δ 164.2 (d, *J* = 240.0 Hz), 157.9, 152.5 (d, *J* = 8.0 Hz), 147.9 (d, *J* = 15.2 Hz), 129.8, 121.8, 119.1 (d, *J* = 4.0 Hz), 114.8, 107.3 (d, *J* = 38.6 Hz), 67.6 (d, *J* = 3.3 Hz).

^{19}F NMR (376 MHz, Chloroform-*d*) δ -67.43.

HRMS (ESI) m/z calcd. for $C_{12}H_{11}FNO$ ([M+H]$^{+}$): 204.0819, found: 204.0799.

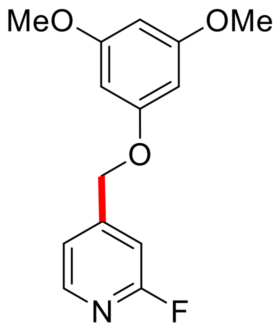

4-((3,5-Dimethoxyphenoxy)methyl)-2-fluoropyridine (10): White solid was obtained with 75% isolated yield following the general procedure B (0.3 mmol scale, 59.2 mg, average yield of two times).

^{1}H NMR (400 MHz, Chloroform-*d*) δ 8.21 (d, *J* = 5.2 Hz, 1H), 7.21 (d, *J* = 5.0 Hz, 1H), 7.01 (s, 1H), 6.12 (s, 3H), 5.06 (s, 2H), 3.77 (s, 6H).

^{13}C NMR (101 MHz, Chloroform-*d*) δ 164.2 (d, *J* = 239.9 Hz), 161.7, 159.8, 152.3 (d, *J* = 8.0 Hz), 147.9 (d, *J* = 15.4 Hz), 119.1 (d, *J* = 4.2 Hz), 107.3 (d, *J* = 38.6 Hz), 93.8, 93.7, 67.7 (d, *J* = 3.2 Hz), 55.5.

^{19}F NMR (376 MHz, Chloroform-*d*) δ -67.44.

HRMS (ESI) m/z calcd. for $C_{14}H_{15}FNO_3$ ([M+H]$^{+}$): 264.1030, found: 264.1025.

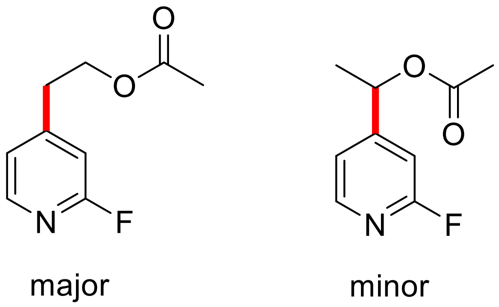

2-(2-Fluoropyridin-4-yl)ethyl acetate (11, major) and 1-(2-Fluoropyridin-4-yl)ethyl acetate (11`, minor): Prepared following general procedure B outlined above. Purification by column chromatography afforded major as a yellow oil (22.8 mg, 41.4% yield) and minor as a yellow oil (20.6 mg, 37.4% yield) (0.3 mmol scale, average yield of four times).

2-(2-Fluoropyridin-4-yl)ethyl acetate (major):

^{1}H NMR (600 MHz, $CDCl_3$) δ 8.11 (d, *J* = 5.2 Hz, 1H), 7.02 (d, *J* = 5.2 Hz, 1H), 6.77 (s, 1H), 4.29 (t, *J* = 6.5 Hz, 2H), 2.95 (t, *J* = 6.6 Hz, 2H), 2.01 (s, 3H).

^{13}C NMR (151 MHz, $CDCl_3$) δ 170.9, 164.1 (d, *J* = 238.4 Hz), 153.1 (d, *J* = 7.8 Hz), 147.7 (d, *J* = 15.2 Hz), 121.9 (d, *J* = 4.2 Hz), 109.7 (d, *J* = 37.2 Hz), 63.2, 34.2 (d, *J* = 3.0 Hz), 20.9.

^{19}F NMR (565 MHz, Chloroform-*d*) δ -68.60.

HRMS (ESI) m/z calcd. for $C_9H_{11}FNO_2$ ($[M+H]^+$): 184.0768, found: 184.0771.

1-(2-Fluoropyridin-4-yl)ethyl acetate (minor):

^{1}H NMR (600 MHz, $CDCl_3$) δ 8.16 (d, *J* = 5.2 Hz, 1H), 7.10 (d, *J* = 5.4 Hz, 1H), 6.86 (s, 1H), 5.81 (q, *J* = 6.8 Hz, 1H), 2.10 (s, 3H), 1.50 (d, *J* = 6.8 Hz, 3H).

^{13}C NMR (151 MHz, $CDCl_3$) δ 170.0, 164.1 (d, *J* = 238.6 Hz), 156.6 (d, *J* = 7.6 Hz), 148.1 (d, *J* = 15.2 Hz), 118.5 (d, *J* = 4.0 Hz), 106.5 (d, *J* = 38.6 Hz), 70.3 (d, *J* = 2.8 Hz), 22.0, 21.2.

^{19}F NMR (565 MHz, Chloroform-*d*) δ -67.45.

HRMS (ESI) m/z calcd. for $C_9H_{11}FNO_2$ ($[M+H]^+$): 184.0768, found: 184.0770.

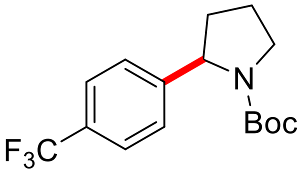

tert-Butyl-2-(4-(trifluoromethyl)phenyl)pyrrolidine-1-carboxylate (12)[7]: Colorless oil was obtained with 46% isolated yield following the general procedure B (0.3 mmol scale, 32.1 mg, average yield of two times).

^{1}H NMR (600 MHz, CDCl$_3$) δ 7.55 (d, *J* = 8.0 Hz, 2H), 7.28 (d, *J* = 8.0 Hz, 2H), 4.98 – 4.79 (m, 1H), 3.65 – 3.54 (m, 2H), 2.38 – 2.29 (m, 1H), 1.90 – 1.88 (m, 2H), 1.82 – 1.77 (m, 1H), 1.47 – 1.45 (m, 3H), 1.17 (s, 6H).

^{13}C NMR (151 MHz, CDCl$_3$) δ 154.5, 149.4, 148.4, 129.0 (q, *J* = 32.4 Hz), 125.9 (q, *J* = 15.6 Hz), 125.5, 125.3, 124.1 (q, *J* = 272.0 Hz), 79.7, 61.2 (60.6), 47.5 (47.3), 36.1 (34.9), 28.6 (28.2), 23.7 (23.3).

^{19}F NMR (565 MHz, CDCl$_3$): δ -62.29, -62.36.

HRMS (ESI) m/z calcd. for $C_{16}H_{21}F_3NO_2$ ([M+H]$^+$): 316.1519, found: 316.1529.

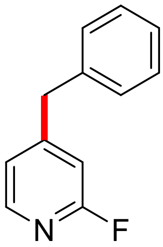

4-Benzyl-2-fluoropyridine (13): Yellow oil was obtained with 71% isolated yield following the general procedure B (0.3 mmol scale, 39.9 mg, average yield of two times).

^{1}H NMR (600 MHz, Chloroform-*d*) δ 7.98 (d, *J* = 5.2 Hz, 1H), 7.23 – 7.20 (m, 2H), 7.16 – 7.13 (m, 1H), 7.07 – 7.05 (m, 2H), 6.88 (dt, *J* = 5.2, 1.6 Hz, 1H), 6.60 (s, 1H), 3.87 (s, 2H).

^{13}C NMR (151 MHz, Chloroform-*d*) δ 164.1 (d, *J* = 238.6 Hz), 156.1 (d, *J* = 7.8 Hz), 147.4 (d, *J* = 15.2 Hz), 138.1, 129.1, 128.9, 127.0, 121.9 (d, *J* = 3.8 Hz), 109.5 (d, *J* = 37.2 Hz), 41.0 (d, *J* = 3.0 Hz).

^{19}F NMR (565 MHz, CDCl$_3$): δ -68.62.

HRMS (ESI) m/z calcd. for $C_{12}H_{11}FN$ ([M+H]$^+$): 188.0870, found: 188.0863.

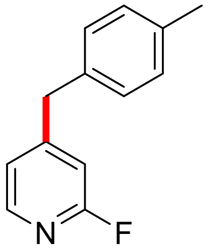

2-Fluoro-4-(4-methylbenzyl)pyridine (14): Colorless oil was obtained with 81% isolated yield following the general procedure B (0.3 mmol scale, 48.9 mg, average yield of two times).

^{1}H NMR (600 MHz, Chloroform-*d*) δ 8.09 (d, *J* = 5.2 Hz, 1H), 7.15 (d, *J* = 7.8 Hz, 2H), 7.07 (d, *J* = 8.0 Hz, 2H), 7.00 (dt, *J* = 5.2, 1.8 Hz, 1H), 6.72 (s, 1H), 3.95 (s, 2H), 2.34 (s, 3H).

^{13}C NMR (151 MHz, Chloroform-*d*) δ 164.2 (d, *J* = 238.4 Hz), 156.5 (d, *J* = 7.8 Hz), 147.4 (d, *J* = 15.2 Hz), 136.7, 135.1, 129.6, 129.0, 121.9 (d, *J* = 3.8 Hz), 109.5 (d, *J* = 37.2 Hz), 40.7 (d, *J* = 3.0 Hz), 21.1.

^{19}F NMR (565 MHz, $CDCl_3$): δ -68.74.

HRMS (ESI) m/z calcd. for $C_{13}H_{13}FN$ ([M+H]$^+$): 202.1027, found: 202.1020.

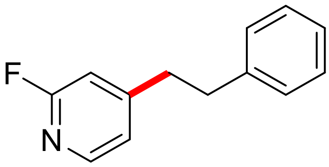

2-Fluoro-4-phenethylpyridine (15): White solid was obtained with 67% isolated yield following the general procedure B (0.3 mmol scale, 40.4 mg, average yield of two times).

^{1}H NMR (400 MHz, Chloroform-*d*) δ 8.09 (d, *J* = 5.2 Hz, 1H), 7.31 – 7.27 (m, 2H), 7.24 – 7.20 (m, 1H), 7.16 – 7.14 (m, 2H), 6.98 – 6.95 (m, 1H), 6.71 (s, 1H), 2.98 – 2.93 (m, 4H).

^{13}C NMR (101 MHz, Chloroform-*d*) δ 164.2 (d, *J* = 239.4 Hz), 156.6 (d, *J* = 7.8 Hz), 147.4 (d, *J* = 15.4 Hz), 140.3, 128.7, 128.5, 126.5, 121.8 (d, *J* = 3.8 Hz), 109.3 (d, *J* =

36.8 Hz), 37.0 (d, J = 2.8 Hz), 36.4.

^{19}F NMR (376 MHz, CDCl$_3$): δ -68.91.

HRMS (ESI) m/z calcd. for $C_{13}H_{13}FN$ ([M+H]$^+$): 202.1027, found: 202.1021.

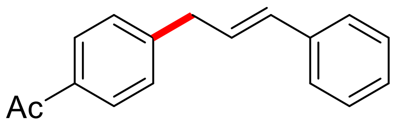

1-(4-Cinnamylphenyl)ethan-1-one (16)[8]: Yellow oil was obtained with 71% isolated yield following the general procedure B (0.3 mmol scale, 50.3 mg, average yield of three times).

^{1}H NMR (600 MHz, Chloroform-*d*) δ 7.92 (d, J = 8.2 Hz, 2H), 7.37 (d, J = 7.2 Hz, 2H), 7.35 (d, J = 8.0 Hz, 2H), 7.31 (t, J = 7.6 Hz, 2H), 7.23 (t, J = 7.2 Hz, 1H), 6.48 (d, J = 15.6 Hz, 1H), 6.34 (dt, J = 15.6, 6.8 Hz, 1H), 3.61 (d, J = 6.8 Hz, 2H), 2.60 (s, 3H).

^{13}C NMR (151 MHz, Chloroform-*d*) δ 198.0, 146.0, 137.3, 135.5, 132.0, 129.0, 128.8, 128.7, 128.0, 127.5, 126.3, 39.4, 26.7.

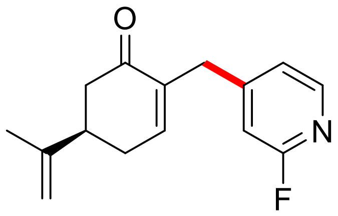

(*R*)-2-((2-fluoropyridin-4-yl)methyl)-5-(prop-1-en-2-yl)cyclohex-2-en-1-one (17): Yellow oil was obtained with 49% isolated yield following the general procedure B (0.3 mmol scale, 36.1 mg, average yield of two times).

^{1}H NMR (600 MHz, Chloroform-*d*) δ 8.08 (d, J = 5.2 Hz, 1H), 7.00 (d, J = 4.6 Hz, 1H), 6.76 (dd, J = 6.0, 2.4 Hz, 1H), 6.74 (s, 1H), 4.82 (s, 1H), 4.75 (s, 1H), 3.54 (s, 2H), 2.73 – 2.68 (m, 1H), 2.62 – 2.59 (m, 1H), 2.51 (dt, J = 18.6, 5.2 Hz, 1H), 2.41 – 2.33 (m, 2H), 1.75 (s, 3H).

^{13}C NMR (101 MHz, Chloroform-*d*) δ 198.4, 164.1 (d, J = 239.6 Hz), 155.0 (d, J = 8.0 Hz), 147.4 (d, J = 15.2 Hz), 147.2, 146.2, 137.1, 122.1 (d, J = 3.8 Hz), 111.0, 109.7 (d, J = 37.2 Hz), 43.1, 42.2, 35.0 (d, J = 3.0 Hz), 31.3, 20.6.

^{19}F NMR (376 MHz, CDCl$_3$): δ -68.80.

HRMS (ESI) m/z calcd. for $C_{15}H_{17}FNO$ ([M+H]$^+$): 246.1289, found: 246.1283.

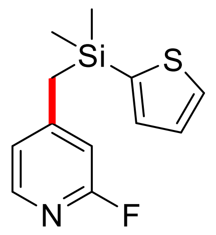

4-((Dimethyl(thiophen-2-yl)silyl)methyl)-2-fluoropyridine (18): Colorless oil was obtained with 31% isolated yield following the general procedure B (0.3 mmol scale, 23.4 mg, average yield of four times).

^{1}H NMR (400 MHz, Chloroform-*d*) δ 7.97 (d, *J* = 5.2 Hz, 1H), 7.64 – 7.62 (m, 1H), 7.20 – 7.17 (m, 2H), 6.70 (d, *J* = 5.2 Hz, 1H), 6.45 (s, 1H), 2.39 (s, 2H), 0.34 (s, 6H).

^{13}C NMR (101 MHz, Chloroform-*d*) δ 164.0 (d, *J* = 238.4 Hz), 155.2 (d, *J* = 8.2 Hz), 146.8 (d, *J* = 15.8 Hz), 135.9, 135.3, 131.5, 128.4, 121.6 (d, *J* = 3.8 Hz), 108.5 (d, *J* = 37.2 Hz), 27.9 (d, *J* = 3.0 Hz), -2.3.

^{19}F NMR (376 MHz, $CDCl_3$): δ -69.68.

HRMS (APCI) m/z calcd. for $C_{12}H_{15}FNSSi$ ([M+H]$^+$): 252.0673, found: 252.0673.

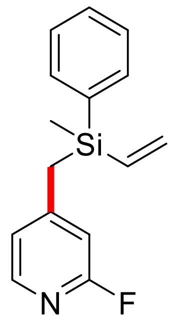

2-Fluoro-4-((methyl(phenyl)(vinyl)silyl)methyl)pyridine (19): Colorless oil was obtained with 66% isolated yield following the general procedure B (0.3 mmol scale, 51 mg, average yield of two times).

^{1}H NMR (400 MHz, Chloroform-*d*) δ 7.95 (d, *J* = 5.0 Hz, 1H), 7.45 – 7.34 (m, 5H), 6.70 (d, *J* = 4.6 Hz, 1H), 6.45 (s, 1H), 6.25 (dd, *J* = 19.7, 15.0 Hz, 1H), 6.15 (dd, J = 14.4, 3.7 Hz, 1H), 5.77 (dd, *J* = 19.6, 3.6 Hz, 1H), 2.45 (s, 2H), 0.35 (s, 3H).

^{13}C NMR (101 MHz, Chloroform-*d*) δ 164.1 (d, *J* = 238.2 Hz), 155.3 (d, *J* = 8.2 Hz), 146.8 (d, *J* = 15.8 Hz), 135.5, 134.9, 134.4, 134.2, 129.9, 128.1, 121.8 (d, *J* = 3.4 Hz),

108.7 (d, $J = 37.2$ Hz), 25.7 (d, $J = 3.0$ Hz), -5.5.

^{19}F NMR (376 MHz, CDCl$_3$): δ -69.82.

HRMS (APCI) m/z calcd. for $C_{15}H_{17}FNSi$ ([M+H]$^+$): 258.1109, found: 258.1106.

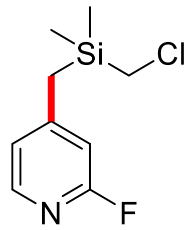

4-(((Chloromethyl)dimethylsilyl)methyl)-2-fluoropyridine (20): Colorless oil was obtained with 69% isolated yield following the general procedure B (0.3 mmol scale, 45.1 mg, average yield of two times).

^{1}H NMR (400 MHz, Chloroform-*d*) δ 8.03 (d, $J = 5.0$ Hz, 1H), 6.85 (d, $J = 5.2$ Hz, 1H), 6.60 (s, 1H), 2.74 (s, 2H), 2.31 (s, 2H), 0.13 (s, 6H).

^{13}C NMR (101 MHz, Chloroform-*d*) δ 164.3 (d, $J = 239.0$ Hz), 155.1 (d, $J = 8.4$ Hz), 147.3 (d, $J = 15.8$ Hz), 121.4 (d, $J = 3.6$ Hz), 108.4 (d, $J = 37.2$ Hz), 28.8, 24.2 (d, $J = 3.0$ Hz), -4.8.

^{19}F NMR (376 MHz, CDCl$_3$): δ -69.37.

HRMS (ESI) m/z calcd. for $C_9H_{14}FNClSi$ ([M+H]$^+$): 281.0562, found: 281.0554.

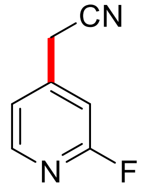

2-(2-Fluoropyridin-4-yl)acetonitrile (21): Yellow oil was obtained with 71% isolated yield following the general procedure B (0.3 mmol scale, 29.0 mg, average yield of three times).

^{1}H NMR (400 MHz, Chloroform-*d*) δ 8.26 (d, $J = 5.2$ Hz, 1H), 7.20 (d, $J = 5.0$ Hz, 1H), 6.97 (s, 1H), 3.83 (s, 2H).

^{13}C NMR (101 MHz, Chloroform-*d*) δ 164.3 (d, $J = 241.2$ Hz), 148.8 (d, $J = 15.6$ Hz), 144.5 (d, $J = 8.0$ Hz), 120.8 (d, $J = 4.4$ Hz), 115.8, 109.2 (d, $J = 39.0$ Hz), 23.2 (d, $J = 3.4$ Hz).

^{19}F NMR (376 MHz, Chloroform-*d*) δ -66.10.

HRMS (ESI) m/z calcd. for $C_7H_6FN_2$ ([M+H]$^+$): 137.0510, found: 137.0506.

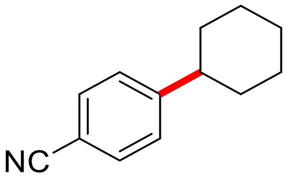

4-Cyclohexylbenzonitrile (22)[14]: Colorless oil was obtained with 74% isolated yield following the general procedure B (0.3 mmol scale, 41.1 mg, average yield of two times).

^{1}H NMR (400 MHz, Chloroform-*d*) δ 7.56 (d, *J* = 8.0 Hz, 2H), 7.29 (d, *J* = 8.0 Hz, 2H), 2.58 – 2.52 (m, 1H), 1.89 – 1.83 (m, 4H), 1.78 – 1.75 (m, 1H), 1.45 – 1.34 (m, 4H), 1.29 – 1.25 (m, 1H).

^{13}C NMR (101 MHz, Chloroform-*d*) δ 153.6, 132.3, 127.8, 119.3, 109.7, 44.9, 34.1, 26.7, 26.0.

HRMS (ESI) m/z calcd. for $C_{13}H_{16}N$ ([M+H]$^+$): 186.1277, found: 186.1270.

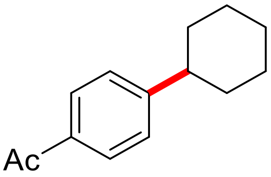

1-(4-Cyclohexylphenyl)ethan-1-one (23)[14]: White solid was obtained with 72% isolated yield following the general procedure B (0.3 mmol scale, 43.7 mg, average yield of two times).

^{1}H NMR (400 MHz, Chloroform-*d*) δ 7.89 (d, *J* = 7.6 Hz, 2H), 7.29 (d, *J* = 8.4 Hz, 2H), 2.59 – 2.53 (m, 4H), 1.90 – 1.82 (m, 4H), 1.78 – 1.73 (m, 1H), 1.48 – 1.35 (m, 4H), 1.31 – 1.21 (m, 1H).

^{13}C NMR (101 MHz, Chloroform-*d*) δ 198.0, 153.9, 135.1, 128.7, 127.2, 44.8, 34.2, 26.8, 26.7, 26.1.

HRMS (ESI) m/z calcd. for $C_{14}H_{19}O$ ([M+H]$^+$): 203.1430, found: 203.1424.

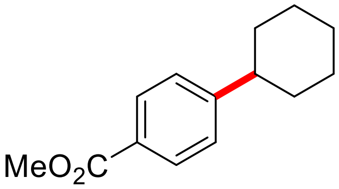

Methyl 4-cyclohexylbenzoate (24)[14]**:** White solid was obtained with 96% isolated yield following the general procedure B (0.3 mmol scale, 62.9 mg, average yield of two times).

^{1}H NMR (400 MHz, Chloroform-*d*) δ 7.10 (d, J = 8.4 Hz, 2H), 6.41 (d, J = 8.2 Hz, 2H), 3.04 (s, 3H), 1.73 – 1.67 (m, 1H), 1.03 – 0.98 (m, 4H), 0.93 – 0.88 (m, 1H), 0.62 – 0.49 (m, 4H), 0.45 – 0.40 (m, 1H).

^{13}C NMR (101 MHz, Chloroform-*d*) δ 167.3, 153.6, 129.8, 127.8, 127.0, 52.1, 44.8, 34.3, 26.9, 26.2.

HRMS (ESI) m/z calcd. for $C_{14}H_{19}O_2$ ([M+H]$^+$): 219.1380, found: 219.1371.

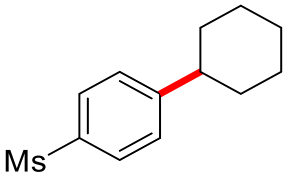

1-Cyclohexyl-4-(methylsulfonyl)benzene (25)[14]**:** White solid was obtained with 85% isolated yield following the general procedure B (0.3 mmol scale, 60.8 mg, average yield of two times).

^{1}H NMR (600 MHz, Chloroform-*d*) δ 7.84 (d, J = 8.2 Hz, 2H), 7.38 (d, J = 8.2 Hz, 2H), 3.03 (s, 3H), 2.61 – 2.57 (m, 1H), 1.88 – 1.83 (m, 4H), 1.78 – 1.75 (m, 1H), 1.45 – 1.36 (m, 4H), 1.28 – 1.24 (m, 1H).

^{13}C NMR (151 MHz, Chloroform-*d*) δ 154.6, 137.9, 127.9, 127.6, 44.7, 44.6, 34.2, 26.7, 26.0.

HRMS (ESI) m/z calcd. for $C_{13}H_{19}SO_2$ ([M+H]$^+$): 239.1100, found: 239.1094.

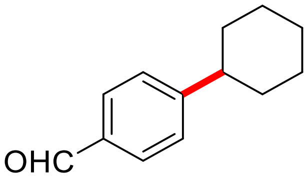

4-Cyclohexylbenzaldehyde (26)[10]**:** Colorless oil was obtained with 49% isolated yield following the general procedure B (0.3 mmol scale, 27.2 mg, average yield of two times).

^{1}H NMR (600 MHz, Chloroform-*d*) δ 9.96 (s, 1H), 7.80 (d, *J* = 8.2 Hz, 2H), 7.36 (d, *J* = 8.0 Hz, 2H), 2.61 – 2.56 (m, 1H), 1.90 – 1.84 (m, 4H), 1.79 – 1.75 (m, 1H), 1.45 – 1.38 (m, 4H), 1.280 – 1.25 (m, 1H).

^{13}C NMR (151 MHz, Chloroform-*d*) δ 192.3, 155.5, 134.6, 130.1, 127.7, 45.0, 34.2, 26.8, 26.1.

HRMS (ESI) m/z calcd. for $C_{13}H_{17}O$ ([M+H]$^+$): 189.1274, found: 189.1269.

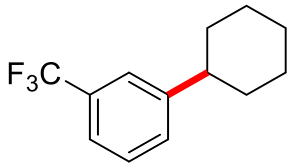

1-Cyclohexyl-3-(trifluoromethyl)benzene (27)[9]**:** Colorless oil was obtained with 73% isolated yield following the general procedure B (0.3 mmol scale, 49.9 mg, average yield of two times).

^{1}H NMR (400 MHz, Chloroform-*d*) δ 7.50 – 7.38 (m, 4H), 2.62 – 2.56 (m, 1H), 1.93 – 1.90 (m, 4H), 1.82 – 1.78 (m, 1H), 1.51 – 1.39 (m, 4H), 1.34 – 1.28 (m, 1H).

^{13}C NMR (101 MHz, Chloroform-*d*) δ 149.0, 130.7 (q, *J* = 31.8 Hz), 130.4, 128.8, 124.6 (q, *J* = 273.8 Hz), 123.7 (q, *J* = 3.8 Hz), 122.8 (q, *J* = 3.8 Hz), 44.6, 34.4, 26.9, 26.1.

^{19}F NMR (376 MHz, $CDCl_3$): δ -62.34.

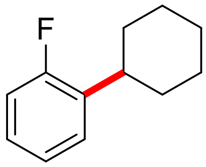

1-Cyclohexyl-2-fluorobenzene (28)[18]**:** Colorless oil was obtained with 79% isolated yield following the general procedure B (0.3 mmol scale, 42.3 mg, average yield of two times).

^{1}H NMR (400 MHz, Chloroform-*d*) δ 7.25 – 7.21 (m, 1H), 7.17 – 7.12 (m, 1H), 7.10 – 7.06 (m, 1H), 7.02 – 6.97 (m, 1H), 2.90 – 2.83 (m, 1H), 1.86 – 1.82 (m, 4H), 1.78 – 1.75 (m, 1H), 1.50 – 1.38 (m, 4H), 1.32 – 1.24 (m, 1H).

^{13}C NMR (101 MHz, Chloroform-*d*) δ 160.7 (d, *J* = 245.6 Hz), 134.6 (d, *J* = 14.7 Hz), 127.8 (d, *J* = 5.4 Hz), 127.1 (d, *J* = 8.4 Hz), 124.1 (d, *J* = 3.4 Hz), 115.3 (d, *J* = 23.2 Hz), 37.2 (d, *J* = 2.0 Hz), 33.2, 27.0, 26.3.

^{19}F NMR (376 MHz, $CDCl_3$): δ -119.49.

HRMS (ESI) m/z calcd. for $C_{12}H_{15}F$ ($[M]^-$): 178.1152, found: 178.1153.

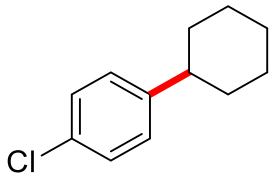

1-Chloro-4-cyclohexylbenzene (29)[14]**:** Colorless oil was obtained with 83% isolated yield following the general procedure B (0.3 mmol scale, 48.5 mg, average yield of two times).

^{1}H NMR (600 MHz, Chloroform-*d*) δ 7.16 (d, *J* = 8.4 Hz, 2H), 7.05 (d, *J* = 8.0 Hz, 2H), 2.41 – 2.37 (m, 1H), 1.77 – 1.75 (m, 4H), 1.68 – 1.65 (m, 1H), 1.32 – 1.28 (m, 4H), 1.18 – 1.17 (m, 1H).

^{13}C NMR (151 MHz, Chloroform-*d*) δ 146.6, 131.4, 128.5, 128.3, 44.1, 34.6, 26.9, 26.2.

HRMS (ESI) m/z calcd. for $C_{12}H_{15}Cl$ ($[M]^-$): 194.0857, found: 194.0859.

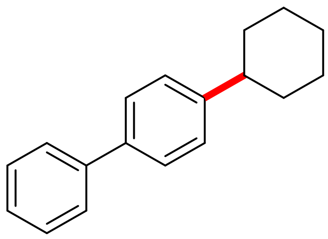

4-Cyclohexyl-1,1'-biphenyl (30)[15]**:** White solid was obtained with 82% isolated yield following the general procedure B (0.3 mmol scale, 58.2 mg, average yield of two times).

^{1}H NMR (600 MHz, Chloroform-*d*) δ 7.60 – 7.58 (m, 2H), 7.55 – 7.53 (m, 2H), 7.45 – 7.42 (m, 2H), 7.35 – 7.32 (m, 1H), 7.30 (d, *J* = 8.0 Hz, 2H), 2.58 – 2.54 (m, 1H), 1.94 – 1.87 (m, 4H), 1.80 – 1.77 (m, 1H), 1.49 – 1.41 (m, 4H), 1.30 – 1.27 (m, 1H).

^{13}C NMR (151 MHz, Chloroform-*d*) δ 147.4, 141.3, 138.9, 128.8, 127.4, 127.2, 127.1, 127.0, 44.4, 34.6, 27.1, 26.3.

HRMS (ESI) m/z calcd. for $C_{18}H_{20}$ ($[M]^-$): 236.1560, found: 236.1559.

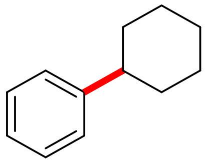

Cyclohexylbenzene (31)[14]: Colorless oil was obtained with 67% isolated yield following the general procedure B (0.3 mmol scale, 32.2 mg, average yield of two times).

^{1}H NMR (600 MHz, Chloroform-*d*) δ 7.41 – 7.37 (m, 2H), 7.32 (d, *J* = 8.4 Hz, 2H), 7.29 – 7.26 (m, 1H), 2.63 – 2.58 (m, 1H), 2.00 – 1.94 (m, 4H), 1.88 – 1.85 (m, 1H), 1.55 – 1.48 (m, 4H), 1.40 – 1.33 (m, 1H).

^{13}C NMR (151 MHz, Chloroform-*d*) δ 148.2, 128.4, 127.0, 125.9, 44.8, 34.6, 27.1, 26.3.

HRMS (ESI) m/z calcd. for $C_{12}H_{17}$ ($[M+H]^+$): 161.1325, found: 161.1319.

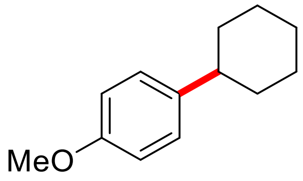

1-Cyclohexyl-4-methoxybenzene (32)[10]: White solid was obtained with 60% isolated yield following the general procedure B (0.3 mmol scale, 34.3 mg, average yield of two times).

^{1}H NMR (400 MHz, Chloroform-*d*) δ 7.15 (d, *J* = 8.4 Hz, 2H), 6.86 (d, *J* = 8.6 Hz, 2H), 3.81 (s, 3H), 2.50 – 2.44 (m, 1H), 1.89 – 1.84 (m, 4H), 1.78 – 1.74 (m, 1H), 1.46 – 1.35 (m, 4H), 1.31 – 1.24 (m, 1H).

^{13}C NMR (101 MHz, Chloroform-*d*) δ 157.8, 140.5, 127.8, 113.8, 55.3, 43.8, 34.9,

27.1, 26.3.

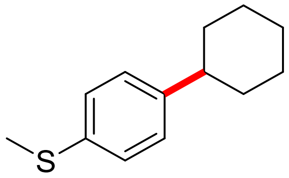

(4-Cyclohexylphenyl)(methyl)sulfane (33)[11]**:** Yellow oil was obtained with 52% isolated yield following the general procedure B (0.3 mmol scale, 32.4 mg, average yield of two times).

^{1}H NMR (400 MHz, Chloroform-*d*) δ 7.22 (d, *J* = 8.0 Hz, 2H), 7.14 (d, *J* = 8.4 Hz, 2H), 2.50 – 2.45 (m, 4H), 1.88 – 1.82 (m, 4H), 1.78 – 1.72 (m, 1H), 1.42 – 1.37 (m, 4H), 1.29 – 1.23 (m, 1H).

^{13}C NMR (101 MHz, Chloroform-*d*) δ 145.5, 135.2, 127.5, 127.3, 44.2, 34.6, 27.0, 26.2, 16.5.

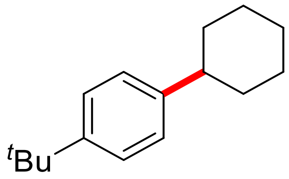

1-(tert-Butyl)-4-cyclohexylbenzene (34)[16]**:** Colorless oil was obtained with 57% isolated yield following the general procedure B (0.3 mmol scale, 37.0 mg, average yield of two times).

^{1}H NMR (600 MHz, Chloroform-*d*) δ 7.31 (d, *J* = 8.4 Hz, 2H), 7.15 (d, *J* = 8.2 Hz, 2H), 2.49 – 2.46 (m, 1H), 1.89 – 1.82 (m, 4H), 1.75 – 1.73 (m, 1H), 1.43 – 1.37 (m, 4H), 1.31 (s, 9H), 1.28 – 1.24 (m, 1H).

^{13}C NMR (151 MHz, Chloroform-*d*) δ 148.6, 145.2, 126.5, 125.3, 44.1, 34.6, 34.5, 31.6, 27.1, 26.4.

HRMS (ESI) m/z calcd. for $C_{16}H_{24}$ ([M]$^-$): 216.1873, found: 216.1872.

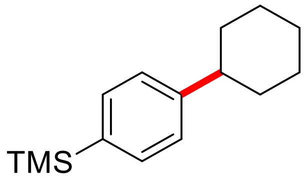

(4-Cyclohexylphenyl)trimethylsilane (35): Colorless oil was obtained with 66% isolated yield following the general procedure B (0.3 mmol scale, 46.0 mg, average yield of two times).

^{1}H NMR (400 MHz, Chloroform-*d*) δ 7.59 (d, *J* = 8.0 Hz, 2H), 7.34 (d, *J* = 7.4 Hz, 2H), 2.64 – 2.58 (m, 1H), 2.02 – 1.95 (m, 4H), 1.90 – 1.85 (m, 1H), 1.58 – 1.50 (m, 3H), 1.42 – 1.37 (m, 1H), 1.03 – 0.94 (m, 1H), 0.38 (s, 9H).

^{13}C NMR (101 MHz, Chloroform-*d*) δ 148.9, 137.5, 133.6, 126.5, 76.8, 44.7, 34.5, 27.1, 26.3.

HRMS (ESI) m/z calcd. for $C_{15}H_{25}Si$ ($[M+H]^+$): 233.1720, found: 233.1719.

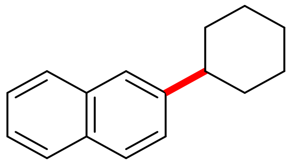

2-Cyclohexylnaphthalene (36)[17]**:** Colorless oil was obtained with 77% isolated yield following the general procedure B (0.3 mmol scale, 48.6 mg, average yield of two times).

^{1}H NMR (400 MHz, Chloroform-*d*) δ 7.86 – 7.82 (m, 3H), 7.69 (s, 1H), 7.52 – 7.42 (m, 3H), 2.75 – 2.69 (m, 1H), 2.05 – 1.93 (m, 4H), 1.87 – 1.83 (m, 1H), 1.64 – 1.49 (m, 4H), 1.40 – 1.34 (m, 1H).

^{13}C NMR (101 MHz, Chloroform-*d*) δ 145.7, 133.8, 132.2, 127.8, 127.7, 127.6, 126.3, 125.9, 125.1, 124.7, 44.8, 34.5, 27.1, 26.4.

HRMS (ESI) m/z calcd. for $C_{16}H_{18}$ ($[M]^-$): 210.1403, found: 210.1400.

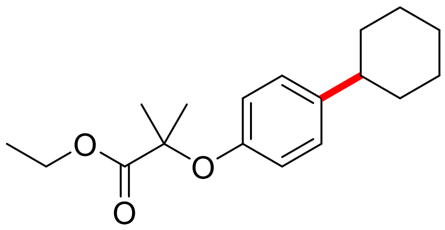

Ethyl 2-(4-cyclohexylphenoxy)-2-methylpropanoate (37): Colorless oil was obtained with 51% isolated yield following the general procedure B (0.3 mmol scale, 44.4 mg, average yield of two times).

^{1}H NMR (400 MHz, Chloroform-*d*) δ 7.05 (d, *J* = 8.4 Hz, 2H), 6.76 (d, *J* = 8.6 Hz, 2H), 4.23 (q, *J* = 7.2 Hz, 2H), 2.45 – 2.37 (m, 1H), 1.88 – 1.77 (m, 4H), 1.74 – 1.71 (m, 1H), 1.57 (s, 6H), 1.41 – 1.31 (m, 4H), 1.27 – 1.23 (m, 4H).

^{13}C NMR (101 MHz, Chloroform-*d*) δ 174.6, 153.4, 142.0, 127.4, 119.2, 79.1, 61.4, 43.8, 34.7, 27.0, 26.3, 25.5, 14.2.

HRMS (ESI) m/z calcd. for $C_{18}H_{27}O_3$ ($[M]^-$): 291.1955, found: 291.1957.

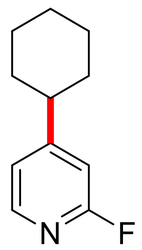

4-Cyclohexyl-2-fluoropyridine (38): Colorless oil was obtained with 93% isolated yield following the general procedure B (0.3 mmol scale, 50.0 mg, average yield of two times).

^{1}H NMR (400 MHz, Chloroform-*d*) δ 8.07 (d, *J* = 5.2 Hz, 1H), 6.99 (d, *J* = 5.2 Hz, 1H), 6.73 (s, 1H), 2.57 – 2.49 (m, 1H), 1.87 – 1.83 (m, 4H), 1.76 – 1.73 (m, 1H), 1.40 – 1.35 (m, 4H), 1.28 – 1.21 (m, 1H).

^{13}C NMR (101 MHz, Chloroform-*d*) δ 164.3 (d, *J* = 238.8 Hz), 162.9 (d, *J* = 7.6 Hz), 147.4 (d, *J* = 15.2 Hz), 120.3 (d, *J* = 3.8 Hz), 107.6 (d, *J* = 36.8 Hz), 43.8 (d, *J* = 2.8 Hz), 33.5, 26.5, 25.9.

^{19}F NMR (376 MHz, Chloroform-*d*) δ -69.04.

HRMS (ESI) m/z calcd. for $C_{11}H_{15}FN$ ($[M]^-$): 180.1183, found: 180.1179.

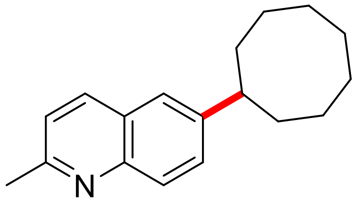

6-Cyclooctyl-2-methylquinoline (39): Colorless oil was obtained with 56% isolated yield following the general procedure B (0.3 mmol scale, 42.6 mg, average yield of two times).

^{1}H NMR (400 MHz, Chloroform-*d*) δ 7.95 (d, *J* = 8.4 Hz, 1H), 7.82 (s, 1H), 7.65 (d,

J = 8.4 Hz, 1H), 7.33 (d, J = 8.6 Hz, 1H), 7.18 (d, J = 8.4 Hz, 1H), 2.98 – 2.91 (m, 1H), 2.71 (s, 3H), 1.96 – 1.76 (m, 6H), 1.69 – 1.59 (m, 8H).

^{13}C NMR (101 MHz, Chloroform-*d*) δ 158.8, 151.9, 148.2, 135.9, 127.3, 126.5, 125.4, 124.7, 121.2, 44.8, 34.1, 27.1, 26.3, 25.9, 25.4.

HRMS (ESI) m/z calcd. for $C_{18}H_{24}N$ ([M+H]$^+$): 254.1903, found: 254.1891.

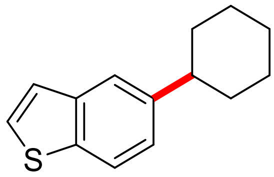

5-Cyclohexylbenzo[*b*]thiophene (40): Colorless oil was obtained with 61% isolated yield following the general procedure B (0.3 mmol scale, 39.6 mg, average yield of two times).

^{1}H NMR (400 MHz, Chloroform-*d*) δ 7.80 (d, J = 8.4 Hz, 1H), 7.67 (s, 1H), 7.41 (d, J = 5.4 Hz, 1H), 7.29 (d, J = 5.4 Hz, 1H), 7.25 – 7.22 (m, 1H), 2.67 – 2.60 (m, 1H), 1.96 – 1.87 (m, 4H), 1.80 – 1.76 (m, 1H), 1.55 – 1.40 (m, 4H), 1.35 – 1.28 (m, 1H).

^{13}C NMR (101 MHz, Chloroform-*d*) δ 144.5, 140.0, 137.4, 126.5, 124.3, 123.9, 122.3, 121.4, 44.7, 35.0, 27.1, 26.3.

HRMS (ESI) m/z calcd. for $C_{14}H_{17}S$ ([M+H]$^+$): 217.1045, found: 217.1051.

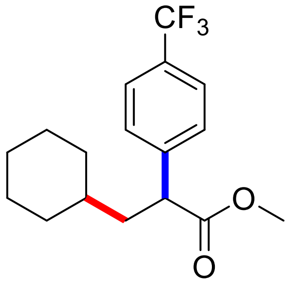

Methyl 3-cyclohexyl-2-(4-(trifluoromethyl)phenyl)propanoate (41)[12]: Colorless oil was obtained with 89% isolated yield following the general procedure C (0.3 mmol scale, 84.2 mg, average yield of two times).

^{1}H NMR (600 MHz, Chloroform-*d*) δ 7.57 (d, J = 8.0 Hz, 2H), 7.43 (d, J = 8.0 Hz, 2H), 3.77 (t, J = 7.8 Hz, 1H), 3.66 (s, 3H), 2.02 – 1.97 (m, 1H), 1.74 – 1.62 (m, 6H), 1.18 – 1.09 (m, 4H), 0.94 – 0.88 (m, 2H).

^{13}C NMR (151 MHz, Chloroform-*d*) δ 174.3, 143.6, 129.6 (q, J = 32.4 Hz), 128.6, 125.8 (q, J = 3.8 Hz), 124.4 (q, J = 272.2 Hz), 52.4, 48.8, 41.3, 35.4, 33.5, 33.1, 26.6, 26.3, 26.2.

^{19}F NMR (565 MHz, Chloroform-*d*) δ -62.51.

HRMS (ESI) m/z calcd. for $C_{17}H_{22}F_3O_2$ ($[M+H]^+$): 315.1566, found: 315.1576.

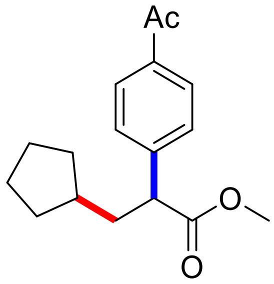

Methyl 2-(4-acetylphenyl)-3-cyclopentylpropanoate (42): Yellow oil was obtained with 81% isolated yield following the general procedure C (0.3 mmol scale, 66.7 mg, average yield of two times).

^{1}H NMR (600 MHz, Chloroform-*d*) δ 7.90 (d, J = 8.0 Hz, 2H), 7.41 (d, J = 8.2 Hz, 2H), 3.67 (t, J = 8.0 Hz, 1H), 3.65 (s, 3H), 2.58 (s, 3H), 2.10 – 2.05 (m, 1H), 1.85 – 1.81 (m, 1H), 1.76 – 1.68 (m, 2H), 1.61 – 1.55 (m, 3H), 1.49 – 1.41 (m, 2H), 1.12 – 1.06 (m, 2H).

^{13}C NMR (101 MHz, Chloroform-*d*) δ 197.8, 174.1, 144.8, 136.2, 128.8, 128.4, 52.2, 51.0, 39.9, 37.9, 32.8, 32.3, 26.7, 25.2 *One peak is missing due to overlap.*

HRMS (ESI) m/z calcd. for $C_{17}H_{23}O_3$ ($[M+H]^+$): 275.1642, found: 275.1637.

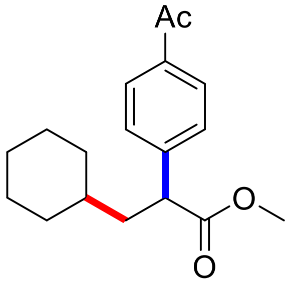

Methyl 2-(4-acetylphenyl)-3-cyclohexylpropanoate (43): Colorless oil was obtained with 85% isolated yield following the general procedure C (0.3 mmol scale, 73.6 mg, average yield of two times).

^{1}H NMR (400 MHz, Chloroform-*d*) δ 7.91 (d, *J* = 8.4 Hz, 2H), 7.40 (d, *J* = 8.4 Hz, 2H), 3.76 (t, *J* = 7.8 Hz, 1H), 3.65 (s, 3H), 2.59 (s, 3H), 2.02 – 1.95 (m, 1H), 1.74 – 1.62 (m, 6H), 1.18 – 1.06 (m, 4H), 0.95 – 0.86 (m, 2H).

^{13}C NMR (101 MHz, Chloroform-*d*) δ 197.9, 174.2, 145.0, 136.3, 128.8, 128.4, 52.3, 48.9, 41.1, 35.4, 33.4, 33.0, 26.7, 26.6, 26.2, 26.1.

HRMS (ESI) m/z calcd. for $C_{18}H_{25}O_3$ ([M+H]$^+$): 289.1798, found: 289.1793.

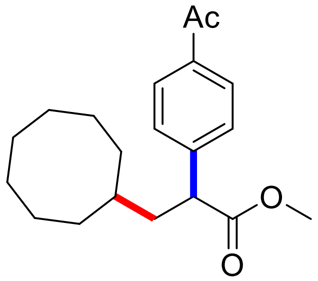

Methyl 2-(4-acetylphenyl)-3-cyclooctylpropanoate (44): Yellow oil was obtained with 72% isolated yield following the general procedure C (0.3 mmol scale, 68.3 mg, average yield of two times).

^{1}H NMR (400 MHz, Chloroform-*d*) δ 7.87 (d, *J* = 7.8 Hz, 2H), 7.37 (d, *J* = 7.8 Hz, 2H), 3.71 (t, *J* = 7.8 Hz, 1H), 3.61 – 3.60 (m, 3H), 2.54 – 2.53 (m, 3H), 1.97 (dt, *J* = 14.4, 7.2 Hz, 1H), 1.67 – 1.23 (m, 16H).

^{13}C NMR (101 MHz, Chloroform-*d*) δ 197.5, 174.0, 144.8, 136.1, 128.7, 128.3, 52.1, 49.3, 41.3, 34.7, 32.1, 31.6, 27.3, 27.2, 26.5, 26.2, 25.2, 25.1.

HRMS (ESI) m/z calcd. for $C_{20}H_{29}O_3$ ([M+H]$^+$): 317.2111, found: 317.2110.

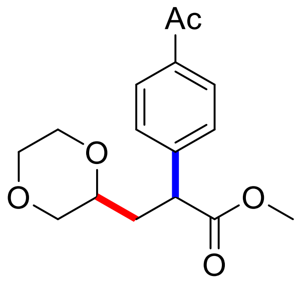

Methyl 2-(4-acetylphenyl)-3-cyclooctylpropanoate (45): White solid was obtained with 49% isolated yield following the general procedure C (0.3 mmol scale, 43.0 mg, average yield of two times, d.r. = 1:1).

^{1}H NMR (400 MHz, Chloroform-*d, mixture of two diastereomers*) δ 7.92 – 7.87 (m, 2H), 7.39 – 7.35 (m, 2H), 3.97 – 3.89 (m, 2H), 3.76 – 3.68 (m, 2H), 3.67 – 3.64 (m, 2.51H), 3.63 (s, 1.53H), 3.58 – 3.51 (m, 2.52H), 3.29 – 3.15 (m, 1.58H), 2.57 (s, 1.53H), 2.56 (s, 1.46H), 2.22 – 2.08 (m, 1H), 1.83 – 1.76 (m, 0.53H), 1.69 – 1.62 (m, 0.49H).

^{13}C NMR (101 MHz, Chloroform-*d, mixture of two diastereomers*) δ 197.7, 173.8, 173.4, 144.5, 143.7, 136.4, 136.3, 128.9, 128.8, 128.5, 128.1, 73.2, 72.4, 71.1, 66.8, 66.7, 66.5(3), 66.4(8), 52.4, 52.3, 46.9, 46.8, 35.3, 34.7, 26.8 *Three peaks are missing due to overlap.*

HRMS (ESI) m/z calcd. for $C_{16}H_{21}O_5$ ($[M+H]^+$): 293.1384, found: 293.1382.

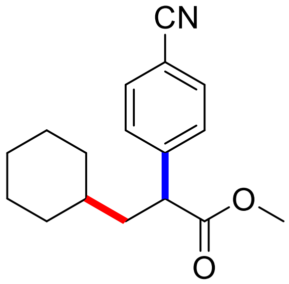

Methyl 2-(4-cyanophenyl)-3-cyclohexylpropanoate (46)[12]: Colorless oil was obtained with 69% isolated yield following the general procedure C (0.3 mmol scale, 56.2 mg, average yield of two times).

^{1}H NMR (600 MHz, Chloroform-*d*) δ 7.58 (d, *J* = 6.8 Hz, 2H), 7.40 (d, *J* = 8.2 Hz, 2H), 3.73 (t, *J* = 7.8 Hz, 1H), 3.63 (s, 3H), 1.98 – 1.93 (m, 1H), 1.69 – 1.59 (m, 6H), 1.14 – 1.04 (m, 4H), 0.90 – 0.85 (m, 2H).

^{13}C NMR (101 MHz, Chloroform-*d*) δ 173.8, 144.9, 132.5, 129.0, 118.8, 111.3, 52.4, 49.0, 41.0, 35.4, 33.3, 32.9, 26.5, 26.1, 26.0.

HRMS (ESI) m/z calcd. for $C_{17}H_{22}NO_2$ ($[M+H]^+$): 272.1645, found: 272.1640.

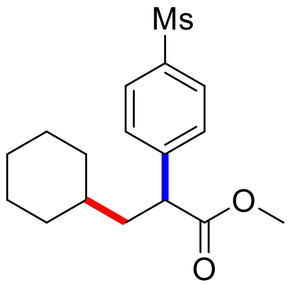

Methyl 3-cyclohexyl-2-(4-(methylsulfonyl)phenyl)propanoate (47): White solid was obtained with 81% isolated yield following the general procedure C (0.3 mmol scale, 78.8 mg, average yield of two times).

^{1}H NMR (400 MHz, Chloroform-*d*) δ 7.87 (d, *J* = 8.4 Hz, 2H), 7.50 (d, *J* = 8.4 Hz, 2H), 3.78 (t, *J* = 7.8 Hz, 1H), 3.65 (s, 3H), 3.03 (s, 3H), 2.02 – 1.94 (m, 1H), 1.71 – 1.59 (m, 6H), 1.16 – 1.04 (m, 4H), 0.94 – 0.85 (m, 2H).

^{13}C NMR (101 MHz, Chloroform-*d*) δ 173.8, 145.8, 139.4, 129.1, 127.8, 52.3, 48.8, 44.6, 41.1, 35.3, 33.3, 32.9, 26.4, 26.1, 26.0.

HRMS (ESI) m/z calcd. for $C_{17}H_{25}O_4S$ ([M+H]$^+$): 325.1468, found: 325.1458.

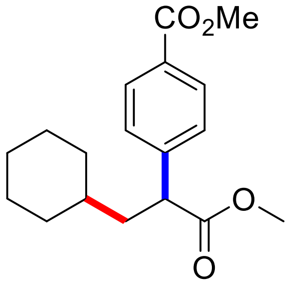

Methyl 4-(3-cyclohexyl-1-methoxy-1-oxopropan-2-yl)benzoate (48): Yellow oil was obtained with 74% isolated yield following the general procedure C (0.3 mmol scale, 67.6 mg, average yield of two times).

^{1}H NMR (600 MHz, Chloroform-*d*) δ 7.98 (d, *J* = 8.4 Hz, 2H), 7.37 (d, *J* = 8.2 Hz, 2H), 3.90 (s, 3H), 3.75 (t, *J* = 7.8 Hz, 1H), 3.65 (s, 3H), 2.00 – 1.95 (m, 1H), 1.73 – 1.60 (m, 6H), 1.15 – 1.07 (m, 4H), 0.92 – 0.87 (m, 2H).

^{13}C NMR (151 MHz, Chloroform-*d*) δ 174.3, 167.0, 144.7, 130.0, 129.2, 128.2, 52.3, 52.2, 48.9, 41.1, 35.3, 33.4, 33.0, 26.5, 26.2, 26.1.

HRMS (APCI) m/z calcd. for $C_{18}H_{25}O_4$ ([M+H]$^+$): 305.1747, found: 305.1763.

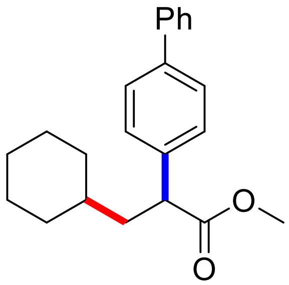

Methyl 2-([1,1'-biphenyl]-4-yl)-3-cyclohexylpropanoate (49)[12]**:** Colorless oil was obtained with 83% isolated yield following the general procedure C (0.3 mmol scale, 80.3 mg, average yield of two times).

^{1}H NMR (600 MHz, Chloroform-*d*) δ 7.59 (dd, *J* = 8.2, 1.4 Hz, 2H), 7.56 (d, *J* = 8.2 Hz, 2H), 7.45 – 7.43 (m, 2H), 7.39 (d, *J* = 8.2 Hz, 2H), 7.36 – 7.33 (m, 1H), 3.76 (t, *J* = 7.8 Hz, 1H), 3.68 (s, 3H), 2.05 – 2.00 (m, 1H), 1.79 – 1.63 (m, 6H), 1.22 – 1.13 (m, 4H), 0.97 – 0.91 (m, 2H).

^{13}C NMR (151 MHz, Chloroform-*d*) δ 175.1, 141.0, 140.3, 138.7, 129.0, 128.6, 127.6, 127.5, 127.3, 52.3, 48.6, 41.3, 35.5, 33.5, 33.2, 26.7, 26.4, 26.3.

HRMS (ESI) m/z calcd. for $C_{22}H_{27}O_2$ ($[M+H]^+$): 323.2006, found: 323.1999.

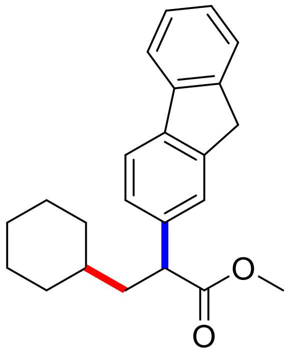

Methyl 3-cyclohexyl-2-(9H-fluoren-2-yl)propanoate (50): White solid was obtained with 71% isolated yield following the general procedure C (0.3 mmol scale, 71.3 mg, average yield of two times).

^{1}H NMR (400 MHz, Chloroform-*d*) δ 7.76 (d, *J* = 7.4 Hz, 1H), 7.73 (d, *J* = 7.8 Hz, 1H), 7.54 (d, *J* = 7.4 Hz, 1H), 7.51 (s, 1H), 7.38 – 7.26 (m, 3H), 3.89 (s, 2H), 3.78 (t, *J* = 7.8 Hz, 1H), 3.68 (s, 3H), 2.07 – 2.00 (m, 1H). 1.80 – 1.63 (m, 6H), 1.23 – 1.12 (m, 4H). 0.98 – 0.90 (m, 2H).

^{13}C NMR (101 MHz, Chloroform-*d*) δ 175.1, 143.8, 143.4, 141.5, 141.0, 138.2, 126.9, 126.8, 126.7, 125.1, 124.6, 120.0, 119.9, 52.1, 49.0, 41.4, 37.0, 35.4, 33.5, 33.1, 26.6, 26.3, 26.2.

HRMS (APCI) m/z calcd. for $C_{23}H_{27}O_2$ ($[M+H]^+$): 335.2006, found:335.2027.

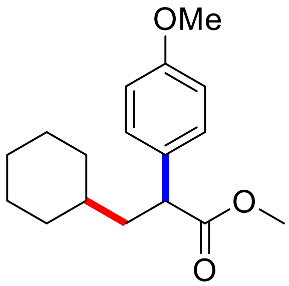

Methyl 3-cyclohexyl-2-(4-methoxyphenyl)propanoate (51)[12]: White solid was obtained with 58% isolated yield following the general procedure C (0.3 mmol scale, 48.1 mg, average yield of two times).

^{1}H NMR (600 MHz, Chloroform-*d*) δ 7.23 (d, *J* = 8.6 Hz, 2H), 6.85 (d, *J* = 8.6 Hz, 2H), 3.79 (s, 3H), 3.66 – 3.64 (m, 4H), 1.96 – 1.91 (m, 1H), 1.75 – 1.60 (m, 6H), 1.18 – 1.11 (m, 4H), 0.94 – 0.89 (m, 2H).

^{13}C NMR (151 MHz, Chloroform-*d*) δ 175.2, 158.8, 131.6, 129.0, 114.1, 55.3, 52.0, 47.9, 41.2, 35.3, 33.4, 33.0, 26.6, 26.2(4), 26.1(9).

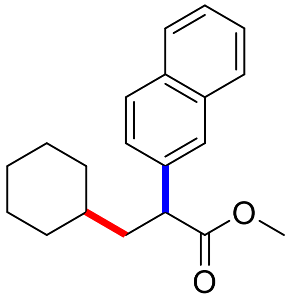

Methyl 3-cyclohexyl-2-(naphthalen-2-yl)propanoate (52)[12]: White solid was obtained with 77% isolated yield following the general procedure C (0.3 mmol scale, 88.9 mg, average yield of two times).

^{1}H NMR (600 MHz, Chloroform-*d*) δ 7.84 – 7.82 (m, 3H), 7.76 (s, 1H), 7.50 – 7.45 (m, 3H), 3.89 (t, *J* = 7.8 Hz, 1H), 3.67 (s, 3H), 2.10 – 2.05 (m, 1H), 1.83 – 1.63 (m, 6H), 1.21 – 1.13 (m, 4H), 0.99 – 0.92 (m, 2H).

^{13}C NMR (151 MHz, Chloroform-*d*) δ 174.9, 137.0, 133.5, 132.7, 128.4, 127.9, 127.7, 126.9, 126.2, 126.1, 125.9, 52.2, 48.9, 41.1, 35.3, 33.5, 33.0, 26.6, 26.2, 26.1.

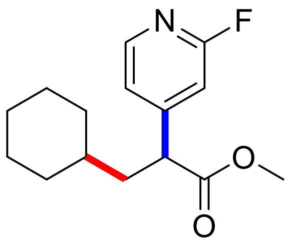

Methyl 3-cyclohexyl-2-(2-fluoropyridin-4-yl)propanoate (53): Colorless oil was obtained with 79% isolated yield following the general procedure C (0.3 mmol scale, 62.9 mg, average yield of two times).

^{1}H NMR (400 MHz, Chloroform-*d*) δ 8.06 (d, *J* = 5.0 Hz, 1H), 7.07 – 7.05 (m, 1H), 6.82 (s, 1H), 3.66 (t, *J* = 7.8 Hz, 1H), 3.60 – 3.57 (m, 3H), 1.94 – 1.86 (m, 1H), 1.64 – 1.53 (m, 6H), 1.09 – 1.00 (m, 4H), 0.87 – 0.79 (m, 2H).

^{13}C NMR (101 MHz, Chloroform-*d*) δ 173.0, 164.1 (d, *J* = 240.0 Hz), 154.0 (d, *J* = 7.8 Hz), 147.8 (d, *J* = 15.4 Hz), 121.1 (d, *J* = 4.2 Hz), 109.0 (d, *J* = 37.8 Hz), 52.4, 48.2 (d, *J* = 2.8 Hz), 40.7, 35.3, 33.2, 32.9, 26.4, 26.1, 26.0.

^{19}F NMR (565 MHz, Chloroform-*d*) δ -67.90.

HRMS (ESI) m/z calcd. for $C_{15}H_{21}FNO_2$ ($[M+H]^+$): 266.1551, found: 266.1546.

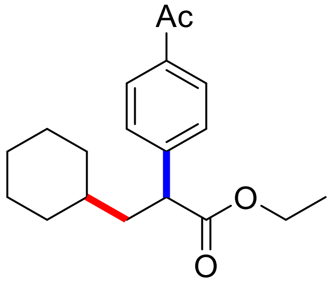

Ethyl 2-(4-acetylphenyl)-3-cyclohexylpropanoate (54): Colorless oil was obtained with 85% isolated yield following the general procedure C (0.3 mmol scale, 77.1 mg, average yield of two times).

^{1}H NMR (400 MHz, Chloroform-*d*) δ 7.90 (d, *J* = 8.2 Hz, 2H), 7.40 (d, *J* = 8.2 Hz, 2H), 4.18 – 4.03 (m, 2H), 3.73 (t, *J* = 7.8 Hz, 1H), 2.58 (s, 3H), 2.01 – 1.94 (m, 1H), 1.74 – 1.61 (m, 6H), 1.19 (t, *J* = 7.2 Hz, 3H), 1.16 – 1.03 (m, 4H), 0.94 – 0.85 (m, 2H).

^{13}C NMR (101 MHz, Chloroform-*d*) δ 197.9, 173.7, 145.1, 136.1, 128.8, 128.3, 61.0, 49.1, 41.1, 35.4, 33.4, 33.0, 26.7, 26.5, 26.2, 26.1, 14.2.

HRMS (ESI) m/z calcd. for $C_{19}H_{27}O_3$ ($[M+H]^+$): 303.1955, found: 303.1950.

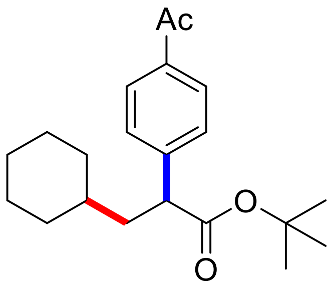

***tert*-Butyl 2-(4-acetylphenyl)-3-cyclohexylpropanoate (55):** Yellow solid was obtained with 53% isolated yield following the general procedure C (0.3 mmol scale, 51.2 mg, average yield of two times).

^{1}H NMR (600 MHz, Chloroform-*d*) δ 7.89 (d, *J* = 8.4 Hz, 2H), 7.38 (d, *J* = 8.0 Hz, 2H), 3.63 (t, *J* = 7.8 Hz, 1H), 2.58 (s, 3H), 1.95 – 1.90 (m, 1H), 1.73 – 1.57 (m, 6H), 1.37 (s, 9H), 1.16 – 1.09 (m, 4H), 0.92 – 0.86 (m, 2H).

^{13}C NMR (151 MHz, Chloroform-*d*) δ 198.0, 172.9, 145.7, 135.9, 128.7, 128.2, 81.0, 50.0, 41.1, 35.5, 33.3, 33.1, 28.0, 26.7, 26.5, 26.3, 26.2.

HRMS (APCI) m/z calcd. for $C_{21}H_{31}O_3$ ($[M+H]^+$): 331.2268, found: 331.2287.

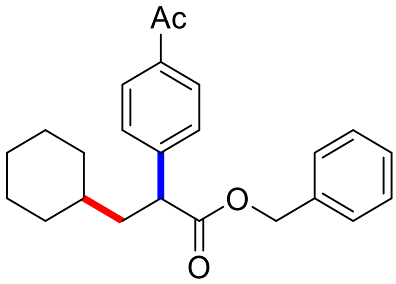

Benzyl 2-(4-acetylphenyl)-3-cyclohexylpropanoate (56): White solid was obtained with 67% isolated yield following the general procedure C (0.3 mmol scale, 73.3 mg, average yield of two times).

^{1}H NMR (400 MHz, Chloroform-*d*) δ 7.89 (d, *J* = 8.4 Hz, 2H), 7.40 (d, *J* = 8.4 Hz, 2H), 7.33 – 7.28 (m, 3H), 7.25 – 7.22 (m, 2H), 5.13 (d, *J* = 12.4 Hz, 1H), 5.04 (d, *J* = 12.4 Hz, 1H), 3.81 (t, *J* = 7.8 Hz, 1H), 2.58 (s, 3H), 2.04 – 1.97 (m, 1H), 1.71 – 1.59 (m, 6H), 1.16 – 1.05 (m, 4H), 0.93 – 0.84 (m, 2H).

^{13}C NMR (101 MHz, Chloroform-*d*) δ 197.8, 173.5, 144.8, 136.2, 135.9, 128.8, 128.6, 128.4, 128.3, 128.1, 66.8, 49.0, 41.0, 35.4, 33.3, 33.0, 26.7, 26.5, 26.1 *One peak is missing due to overlap.*

HRMS (APCI) m/z calcd. for $C_{24}H_{29}O_3$ ([M+H]$^+$): 365.2111, found:365.2126.

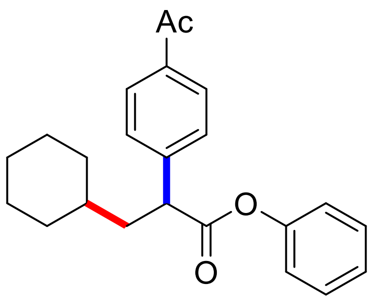

Phenyl 2-(4-acetylphenyl)-3-cyclohexylpropanoate (57): White solid was obtained with 71% isolated yield following the general procedure C (0.3 mmol scale, 74.7 mg, average yield of two times).

^{1}H NMR (400 MHz, Chloroform-*d*) δ 7.97 (d, *J* = 8.4 Hz, 2H), 7.51 (d, *J* = 8.4 Hz, 2H), 7.37 – 7.31 (m, 2H), 7.23 – 7.18 (m, 1H), 6.97 (d, *J* = 7.4 Hz, 2H), 4.01 (t, *J* = 7.8 Hz, 1H), 2.61 (s, 3H), 2.17 – 2.10 (m, 1H), 1.80 – 1.63 (m, 6H), 1.28 – 1.14 (m, 4H), 1.04 – 0.94 (m, 2H).

^{13}C NMR (101 MHz, Chloroform-*d*) δ 197.8, 172.3, 150.8, 144.4, 136.4, 129.5, 129.0, 128.4, 126.0, 121.4, 49.1, 41.0, 35.5, 33.4, 33.1, 26.7, 26.5, 26.2, 26.1.

HRMS (APCI) m/z calcd. for $C_{23}H_{27}O_3$ ([M+H]$^+$): 351.1955, found:351.1959.

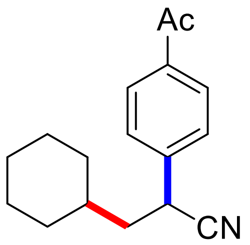

2-(4-Acetylphenyl)-3-cyclohexylpropanenitrile (58): Colorless oil was obtained with 79% isolated yield following the general procedure C (0.3 mmol scale, 60.6 mg, average yield of two times).

^{1}H NMR (600 MHz, Chloroform-*d*) δ 7.96 (d, *J* = 8.4 Hz, 2H), 7.42 (d, *J* = 8.2 Hz, 2H), 3.92 – 3.89 (m, 1H), 2.60 (s, 3H), 1.90 – 1.81 (m, 2H), 1.73 – 1.63 (m, 5H), 1.53 – 1.47 (m, 1H), 1.27 – 1.21 (m, 2H), 1.18 – 1.10 (m, 1H), 0.99 – 0.90 (m, 2H).

^{13}C NMR (151 MHz, Chloroform-*d*) δ 197.5, 141.7, 136.8, 129.2, 127.6, 120.5, 43.5,

35.4, 34.9, 33.3, 32.4, 26.8, 26.3, 26.0, 25.9.

HRMS (APCI) m/z calcd. for $C_{17}H_{22}NO$ ([M+H]$^+$): 256.1696, found: 256.1714.

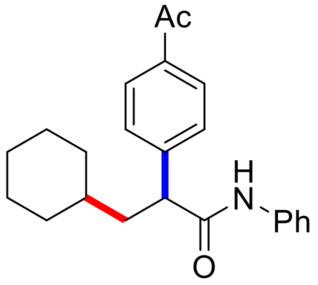

2-(4-Acetylphenyl)-3-cyclohexyl-N-phenylpropanamide (59): White solid was obtained with 53% isolated yield following the general procedure C (0.3 mmol scale, 55.7 mg, average yield of two times).

^{1}H NMR (400 MHz, Chloroform-*d*) δ 7.84 (br, 1H), 7.81 (d, *J* = 8.0 Hz, 2H), 7.38 (d, *J* = 8.2 Hz, 4H), 7.16 (t, *J* = 7.8 Hz, 2H), 6.97 (t, *J* = 7.4 Hz, 1H), 3.68 (t, *J* = 7.6 Hz, 1H), 2.50 (s, 3H), 2.06 – 1.99 (m, 1H), 1.69 – 1.51 (m, 6H), 1.12 – 0.99 (m, 4H), 0.87 – 0.78 (m, 2H).

^{13}C NMR (101 MHz, Chloroform-*d*) δ 198.2, 171.4, 145.8, 138.0, 136.1, 129.0, 128.2, 124.5, 120.1, 51.3, 41.1, 35.3, 33.6, 33.0, 26.7, 26.5, 26.2, 26.1 *One peak is missing due to overlap.*

HRMS (APCI) m/z calcd. for $C_{23}H_{28}NO_2$ ([M+H]$^+$): 350.2115, found: 350.2115.

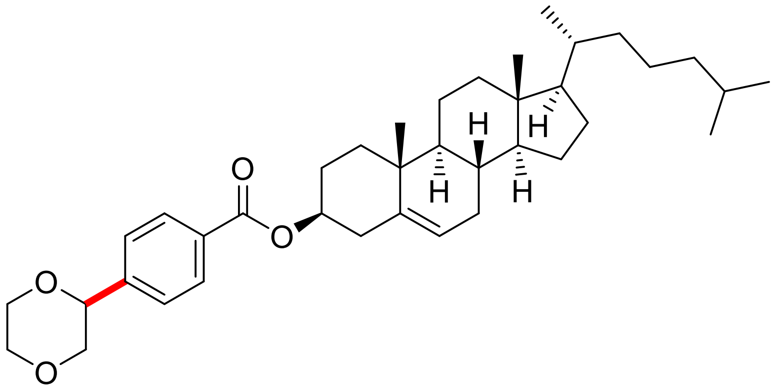

(*3S,8S,9S,10R,13R,14S,17R*)-10,13-Dimethyl-17-((*R*)-6-methylheptan-2-yl)-2,3,4,7,8,9,10,11,12,13,14,15,16,17-tetradecahydro-*1H*-cyclopenta[a]phenanthren-3-yl 4-(1,4-dioxan-2-yl)benzoate (60): White solid was obtained with 67% isolated

yield following the general procedure B (0.3 mmol scale, 116.0 mg).

^{1}H NMR (400 MHz, Chloroform-*d*) δ 8.02 (d, *J* = 8.6 Hz, 2H), 7.41 (d, *J* = 8.4 Hz, 2H), 5.42 – 5.40 (m, 1H), 4.89 – 4.81 (m, 1H), 4.68 (dd, *J* = 10.2, 2.8 Hz, 1H), 3.98 – 3.69 (m, 5H), 3.40 (dd, *J* = 11.8, 10.0 Hz, 1H), 2.45 (d, *J* = 7.6 Hz, 2H), 2.04 – 1.68 (m, 7H), 1.60 – 1.44 (m, 6H), 1.35 – 1.11 (m, 9H), 1.06 (s, 3H), 1.04 – 0.95 (m, 4H), 0.92 (d, *J* = 6.4 Hz, 3H), 0.86 (dd, *J* = 6.6, 2.0 Hz, 6H), 0.68 (s, 3H).

^{13}C NMR (101 MHz, Chloroform-*d*) δ 165.8, 143.2, 139.7, 130.6, 129.8, 126.1, 122.9, 77.6, 74.7, 72.4, 67.1, 66.5, 56.8, 56.2, 50.1, 42.4, 39.8, 39.6, 38.3, 37.1, 36.8, 36.3, 35.9, 32.0, 31.9, 28.4, 28.1, 28.0, 24.4, 23.9, 23.0, 22.7, 21.2, 19.5, 18.8, 11.9.

HRMS (ESI) m/z calcd. for $C_{38}H_{57}O_4$ ($[M+H]^+$): 577.4251, found: 577.4251.

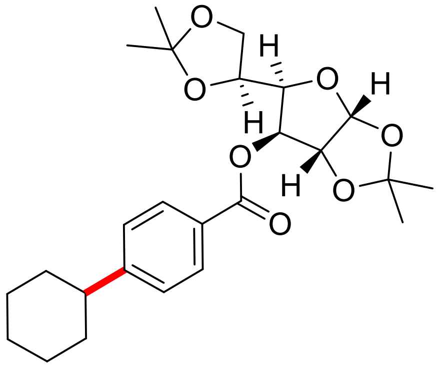

(*3aR,5R,6S,6aR*)-5-((*S*)-2,2-dimethyl-1,3-dioxolan-4-yl)-2,2-dimethyltetrahydrofuro[2,3-*d*][1,3]dioxol-6-yl 4-cyclohexylbenzoate (61): White solid was obtained with 68% isolated yield following the general procedure B (0.3 mmol scale, 90.6 mg).

^{1}H NMR (400 MHz, Chloroform-*d*) δ 7.94 (d, *J* = 8.2 Hz, 2H), 7.28 (d, *J* = 8.2 Hz, 2H), 5.93 (d, *J* = 3.8 Hz, 1H), 5.48 (d, *J* = 2.8 Hz, 1H), 4.60 (d, *J* = 3.8 Hz, 1H), 4.39 – 4.32 (m, 2H), 4.13 – 4.07 (m, 2H), 2.60 – 2.53 (m, 1H), 1.89 – 1.85 (m, 4H), 1.79 – 1.74 (m, 1H), 1.55 (s, 3H), 1.47 – 1.34 (m, 8H), 1.31 (s, 3H), 1.27 (s, 3H).

^{13}C NMR (101 MHz, Chloroform-*d*) δ 165.4, 154.3, 130.0, 127.2, 112.5, 109.5, 105.3, 83.5, 80.1, 76.5, 72.7, 67.3, 44.9, 34.2, 27.0, 26.9, 26.8, 26.3, 26.1, 25.4.

HRMS (ESI) m/z calcd. for $C_{26}H_{37}O_6$ ($[M+H]^+$): 445.2585, found: 445.2583.

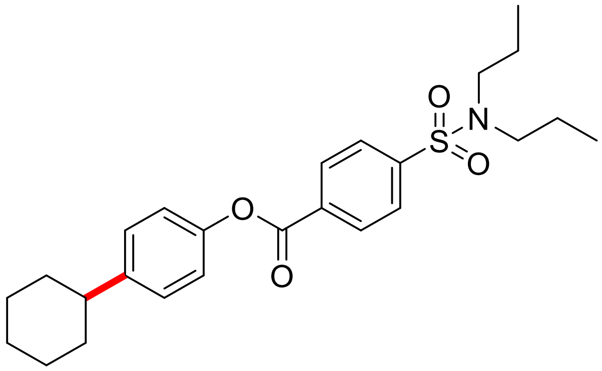

4-Cyclohexylphenyl 4-(*N*,*N*-dipropylsulfamoyl)benzoate (62): White solid was obtained with 41% isolated yield following the general procedure B (0.3 mmol scale, 54.7 mg, average yield of two times).

^{1}H NMR (400 MHz, Chloroform-*d*) δ 8.30 (d, *J* = 7.8 Hz, 2H), 7.93 (d, *J* = 7.6 Hz, 2H), 7.26 (d, *J* = 7.8 Hz, 2H), 7.12 (d, *J* = 7.2 Hz, 2H), 3.12 (t, *J* = 7.4 Hz, 4H), 2.55 – 2.49 (m, 1H), 1.90 – 1.73 (m, 4H), 1.75 (d, *J* = 13.0 Hz, 1H), 1.60 – 1.51 (m, 4H), 1.46 – 1.34 (m, 4H), 1.29 – 1.23 (m, 1H), 0.87 (t, *J* = 7.2 Hz, 6H).

^{13}C NMR (101 MHz, Chloroform-*d*) δ 164.1, 148.6, 146.2, 144.9, 133.1, 130.9, 128.0, 127.2, 121.2, 50.0, 44.1, 34.6, 26.9, 26.2, 22.0, 11.3.

HRMS (ESI) m/z calcd. for $C_{25}H_{34}NSO_4$ ($[M+H]^+$): 444.2203, found: 444.2195.

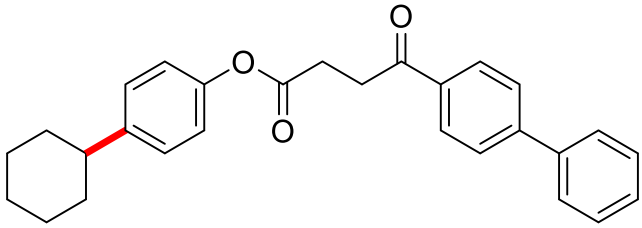

4-Cyclohexylphenyl 4-([1,1'-biphenyl]-4-yl)-4-oxobutanoate (63): White solid was obtained with 49% isolated yield following the general procedure B (0.3 mmol scale, 60.7 mg, average yield of two times).

^{1}H NMR (400 MHz, Chloroform-*d*) δ 8.09 (d, *J* = 8.4 Hz, 2H), 7.70 (d, *J* = 8.4 Hz, 2H), 7.65 – 7.63 (m, 2H), 7.50 – 7.46 (m, 2H), 7.43 – 7.39 (m, 1H), 7.21 (d, *J* = 8.6 Hz, 2H), 7.04 (d, *J* = 8.6 Hz, 2H), 3.46 (t, *J* = 6.6 Hz, 2H), 3.03 (t, *J* = 6.5 Hz, 2H), 2.54 – 2.46 (m, 1H), 1.88 – 1.82 (m, 4H), 1.78 – 1.72 (m, 1H), 1.44 – 1.34 (m, 4H), 1.29 – 1.23 (m, 1H).

^{13}C NMR (101 MHz, Chloroform-*d*) δ 197.6, 171.9, 148.8, 146.1, 145.7, 140.0, 135.3, 129.1, 128.8, 128.4, 127.8, 127.4, 121.3, 44.1, 34.6, 33.6, 28.7, 27.0, 26.2 *One peak is missing due to overlap.*

HRMS (ESI) m/z calcd. for $C_{28}H_{29}O_3$ ([M+H]$^+$): 413.2111, found: 413.2102.

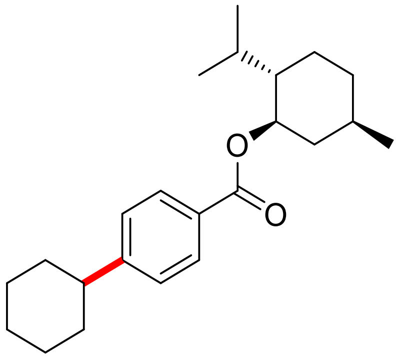

(*1R,2S,5R*)-2-Isopropyl-5-methylcyclohexyl 4-cyclohexylbenzoate (64): White solid was obtained with 69% isolated yield following the general procedure B (0.3 mmol scale, 70.9 mg, average yield of two times).

^{1}H NMR (400 MHz, Chloroform-*d*) δ 7.89 (d, *J* = 8.4 Hz, 2H), 7.19 (d, *J* = 8.4 Hz, 2H), 4.84 (td, *J* = 10.9, 4.3 Hz, 1H), 2.52 – 2.45 (m, 1H), 2.06 – 2.01 (m, 1H), 1.93 – 1.85 (m, 1H), 1.82 – 1.75 (m, 4H), 1.70 – 1.62 (m, 3H), 1.50 – 1.43 (m, 2H), 1.38 – 1.30 (m, 4H), 1.27 – 1.10 (m, 2H), 1.08 – 0.96 (m, 2H), 0.85 – 0.83 (m, 6H), 0.71 (d, *J* = 7.0 Hz, 3H).

^{13}C NMR (101 MHz, Chloroform-*d*) δ 166.3, 153.4, 129.8, 128.6, 126.9, 74.7, 47.4, 44.8, 41.1, 34.5, 34.4, 34.3, 31.6, 26.8, 26.6, 26.2, 23.8, 22.2, 20.9, 16.6.

HRMS (ESI) m/z calcd. for $C_{23}H_{35}O_2$ ([M+H]$^+$): 343.2632, found: 343.2621.

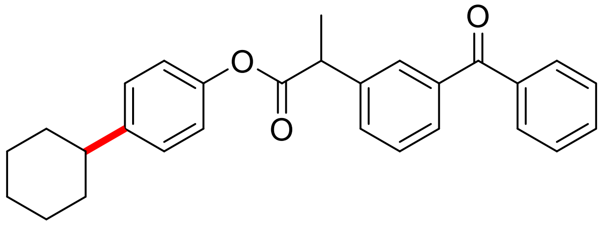

4-Cyclohexylphenyl 2-(3-benzoylphenyl)propanoate (65): Colorless oil was obtained with 47% isolated yield following the general procedure B (0.3 mmol scale,

58.2 mg, average yield of two times).

¹H NMR (400 MHz, Chloroform-*d*) δ 7.85 (s, 1H), 7.83 – 7.80 (m, 2H), 7.73 (dt, *J* = 7.8, 1.6 Hz, 1H), 7.65 (dt, *J* = 7.8, 1.6 Hz, 1H), 7.62 – 7.57 (m, 1H), 7.51 – 7.46 (m, 3H), 7.17 (d, *J* = 8.6 Hz, 2H), 6.92 (d, *J* = 8.6 Hz, 2H), 4.04 (q, *J* = 7.0 Hz, 1H), 2.52 – 2.44 (m, 1H), 1.87 – 1.80 (m, 4H), 1.76 – 1.71 (m, 1H), 1.65 (d, *J* = 7.2 Hz, 3H), 1.40 – 1.35 (m, 4H), 1.29 – 1.25 (m, 1H).

¹³C NMR (101 MHz, Chloroform-*d*) δ 196.5, 172.8, 148.7, 145.8, 140.6, 138.2, 137.6, 132.6, 131.6, 130.2, 129.4, 129.3, 128.8, 128.4, 127.8, 121.0, 45.6, 44.1, 34.6, 26.9, 26.2, 18.6.

HRMS (ESI) m/z calcd. for $C_{28}H_{29}O_3$ ($[M+H]^+$): 413.2111, found: 413.2106.

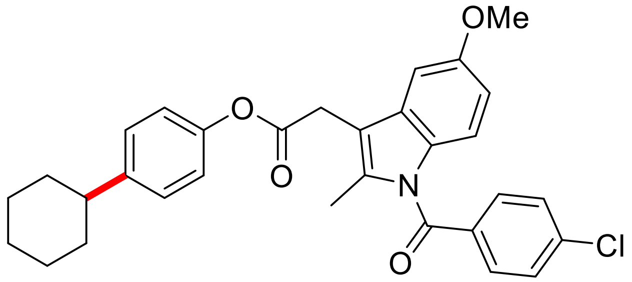

4-Cyclohexylphenyl 2-(1-(4-chlorobenzoyl)-5-methoxy-2-methyl-1H-indol-3-yl)acetate (66): Yellow solid was obtained with 51% isolated yield following the general procedure B (0.3 mmol scale, 78.9 mg).

¹H NMR (400 MHz, Chloroform-*d*) δ 7.69 – 7.66 (m, 2H), 7.49 – 7.46 (m, 2H), 7.18 (d, *J* = 8.6 Hz, 2H), 7.06 (d, *J* = 2.6 Hz, 1H), 6.98 – 6.95 (m, 2H), 6.91 (d, *J* = 9.0 Hz, 1H), 6.70 (dd, *J* = 9.0, 2.6 Hz, 1H), 3.89 (s, 2H), 3.84 (s, 3H), 2.52 – 2.46 (m, 1H), 2.45 (s, 3H), 1.87 – 1.80 (m, 4H), 1.76 – 1.71 (m, 1H), 1.40 – 1.34 (m, 4H), 1.28 – 1.25 (m, 1H).

¹³C NMR (101 MHz, Chloroform-*d*) δ 169.7, 168.5, 156.3, 148.7, 145.9, 139.5, 136.3, 134.0, 131.4, 131.0, 130.7, 129.3, 127.8, 121.1, 115.2, 112.3, 112.0, 101.3, 55.9, 44.1, 34.6, 30.7, 27.0, 26.2, 13.6.

HRMS (ESI) m/z calcd. for $C_{31}H_{31}ClNO_4$ ($[M+H]^+$): 516.1936, found: 516.1929.

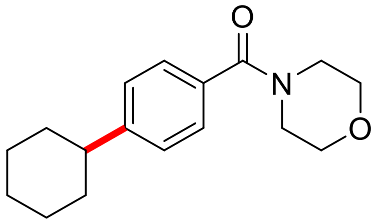

(4-Cyclohexylphenyl)(morpholino)methanone (67)[13]**:** White solid was obtained with 58% isolated yield following the general procedure B (0.3 mmol scale, 47.6 mg, average yield of two times).

^{1}H NMR (400 MHz, Chloroform-*d*) δ 7.31 (d, *J* = 8.2 Hz, 2H), 7.21 (d, *J* = 8.2 Hz, 2H), 3.67 – 3.51 (m, 8H), 2.54 – 2.46 (m, 1H), 1.86 – 1.80 (m, 4H), 1.76 – 1.71 (m, 1H), 1.44 – 1.32 (m, 4H), 1.28 – 1.20 (m, 1H).

^{13}C NMR (101 MHz, Chloroform-*d*) δ 170.7, 150.2, 132.7, 127.3, 127.0, 67.0, 44.5, 34.3, 26.8, 26.1 *One peak is missing due to overlap.*

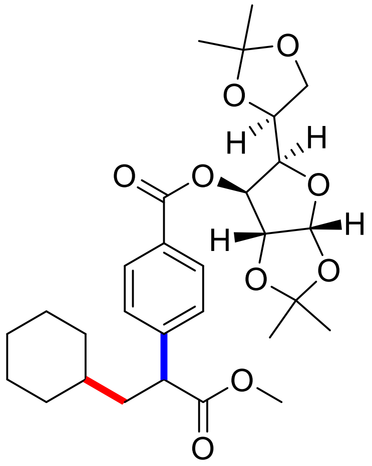

(*3aR,5R,6S,6aR*)-5-((*S*)-2,2-Dimethyl-1,3-dioxolan-4-yl)-2,2-dimethyltetrahydrofuro[2,3-d][1,3]dioxol-6-yl-4-(3-cyclohexyl-1-methoxy-1-oxopropan-2-yl)benzoate (68): Yellow oil was obtained with 81% isolated yield following the general procedure C (0.3 mmol scale, 129.4 mg, average yield of three times).

^{1}H NMR (400 MHz, Chloroform-*d*) δ 7.94 – 7.91 (m, 2H), 7.37 – 7.34 (m, 2H), 5.89 (d, *J* = 3.6 Hz, 1H), 5.44 – 5.43 (m, 1H), 4.57 (d, *J* = 3.8 Hz, 1H), 4.34 – 4.27 (m, 2H), 4.08 – 4.02 (m, 2H), 3.72 (t, *J* = 9.0 Hz, 1H), 3.60 (s, 3H), 1.98 – 1.91 (m, 1H), 1.70 –

1.57 (m, 6H), 1.50 (s, 3H), 1.37 (s, 3H), 1.27 (s, 3H), 1.23 (s, 3H), 1.11 – 1.03 (m, 4H), 0.90 – 0.80 (m, 2H).

^{13}C NMR (101 MHz, Chloroform-*d*) δ 173.9, 164.9, 145.3, 130.0, 128.5, 128.2, 112.3, 109.3, 105.1, 83.4, 79.9, 76.6, 72.6, 67.2, 52.1, 48.8, 40.9, 35.2, 33.3, 32.8, 26.8, 26.7, 26.4, 26.2, 26.1, 26.0, 25.2.

HRMS (ESI) m/z calcd. for $C_{29}H_{41}O_9$ ([M+H]$^+$): 533.2745, found: 533.2745.

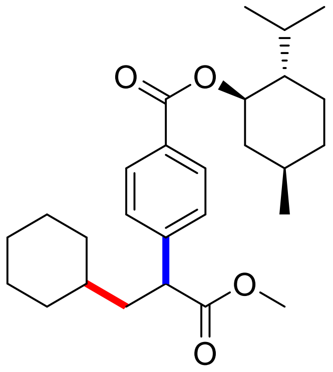

(*1R,2S,5R*)-2-Isopropyl-5-methylcyclohexyl 4-(3-cyclohexyl-1-methoxy-1-oxopropan-2-yl)benzoate (69): Colorless oil was obtained with 73% isolated yield following the general procedure C (0.3 mmol scale, 93.9 mg, average yield of three times).

^{1}H NMR (400 MHz, Chloroform-*d*) δ 7.98 (d, *J* = 8.2 Hz, 2H), 7.37 (d, *J* = 8.2 Hz, 2H), 4.91 (td, *J* = 11.0, 4.4 Hz, 1H), 3.75 (t, *J* = 7.8 Hz, 1H), 3.63 (s, 3H), 2.13 – 2.07 (m, 1H), 2.01 – 1.91 (m, 2H), 1.74 – 1.51 (m, 10H), 1.18 – 1.06 (m, 6H), 0.94 – 0.86 (m, 9H), 0.78 (d, *J* = 7.0 Hz, 3H).

^{13}C NMR (101 MHz, Chloroform-*d*) δ 174.2, 165.9, 144.4, 130.0, 129.9, 128.1, 74.8, 52.1, 48.8, 47.3, 41.1, 41.0, 35.3, 34.4, 33.4, 32.9, 31.5, 26.6, 26.5, 26.2, 26.1, 23.7, 22.1, 20.9, 16.6.

HRMS (ESI) m/z calcd. for $C_{27}H_{41}O_4$ ([M+H]$^+$): 429.2999, found: 429.2991.

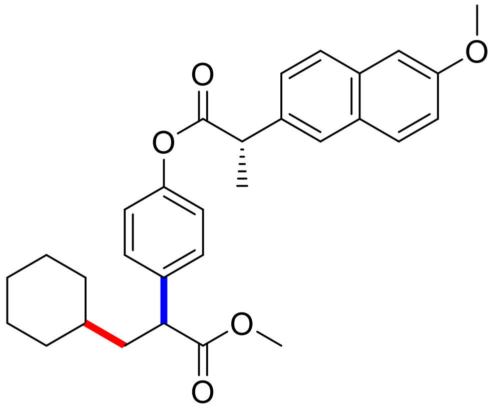

Methyl 3-cyclohexyl-2-(4-(((*S*)-2-(6-methoxynaphthalen-2-yl)propanoyl)oxy)phenyl)propanoate (70): Yellow solid was obtained with 61% isolated yield following the general procedure C (0.3 mmol scale, 86.9 mg, average yield of two times).

^{1}H NMR (400 MHz, Chloroform-*d*) δ 7.76 – 7.72 (m, 3H), 7.49 (dd, *J* = 8.6, 2.0 Hz, 1H), 7.26 (d, *J* = 8.6 Hz, 2H), 7.18 – 7.13 (m, 2H), 6.94 (d, *J* = 8.6 Hz, 2H), 4.08 (q, *J* = 7.2 Hz, 1H), 3.91 (s, 3H), 3.69 – 3.66 (m, 1H), 3.62 (s, 3H), 1.96 – 1.88 (m, 1H), 1.69 – 1.58 (m, 9H), 1.17 – 1.05 (s, 4H), 0.93 – 0.83 (m, 2H).

^{13}C NMR (101 MHz, Chloroform-*d*) δ 174.6, 173.2, 157.9, 150.0, 136.9, 135.2, 133.9, 129.4, 129.1, 128.9, 127.5, 126.2, 126.1, 121.5, 119.2, 105.7, 55.4, 52.0, 48.2, 45.6, 41.2, 35.2, 33.3, 33.0, 26.5, 26.1, 18.6. *One peak is missing due to overlap.*

HRMS (ESI) m/z calcd. for $C_{30}H_{35}O_5$ ([M+H]$^+$): 475.2479, found: 475.2471.

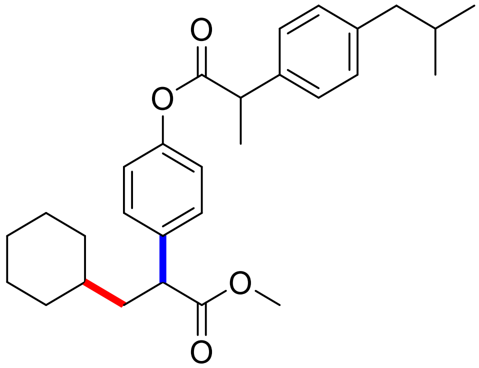

Methyl 3-cyclohexyl-2-(4-((2-(4-isobutylphenyl)propanoyl)oxy)phenyl)propanoate (71): Colorless oil was obtained with 67% isolated yield following the general procedure C (0.3 mmol scale, 90.6 mg, average yield of two times).

^{1}H NMR (400 MHz, Chloroform-*d*) δ 7.30 – 7.26 (m, 4H), 7.14 (d, *J* = 7.6 Hz, 2H), 6.95 (d, *J* = 8.0 Hz, 2H), 3.93 (q, *J* = 7.0 Hz, 1H), 3.67 (t, *J* = 8.0 Hz, 1H), 3.62 (s, 3H), 2.47 (d, *J* = 7.2 Hz, 2H), 1.97 – 1.84 (m, 2H), 1.73 – 1.59 (m, 9H), 1.20 – 1.07 (m, 4H), 0.92 – 0.88 (m, 8H).

^{13}C NMR (101 MHz, Chloroform-*d*) δ 174.7, 173.3, 150.0, 140.9, 137.3, 136.9, 129.6, 128.9, 127.3, 121.5, 52.1, 48.2, 45.4, 45.1, 41.2, 35.2, 33.4, 33.0, 30.3, 26.6, 26.2, 26.1, 22.5, 18.6.

HRMS (ESI) m/z calcd. for $C_{29}H_{39}O_4$ ($[M+H]^+$): 451.2843, found: 451.2841.

3. NMR Spectra

Compound 1

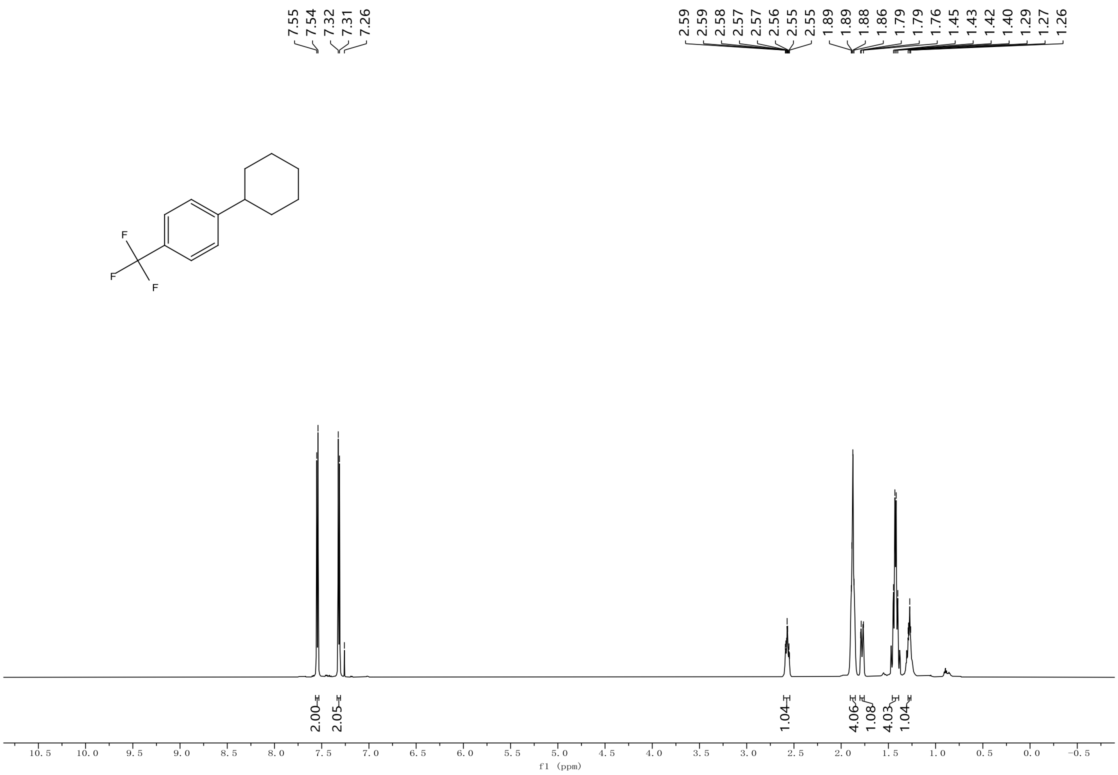

Supplementary Figure 33. ^{1}H NMR spectra of compound **1** (600 MHz, r.t., $CDCl_3$).

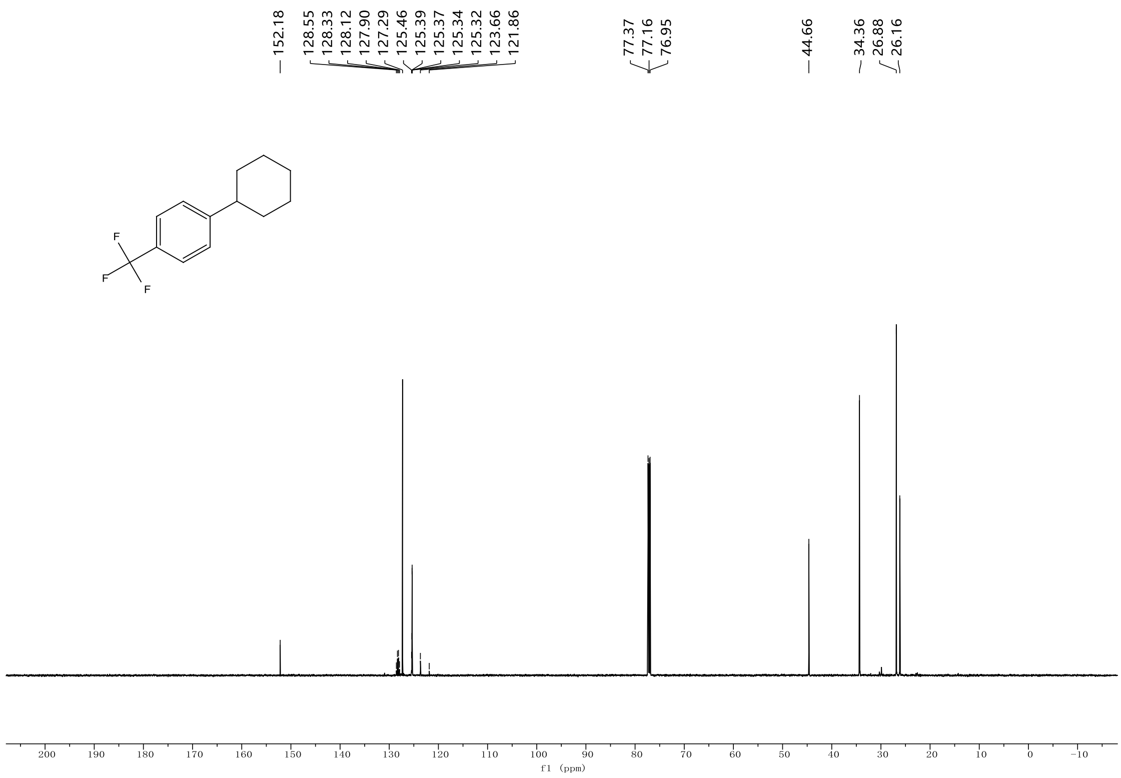

Supplementary Figure 34. ^{13}C NMR spectra of compound **1** (151 MHz, r.t., $CDCl_3$).

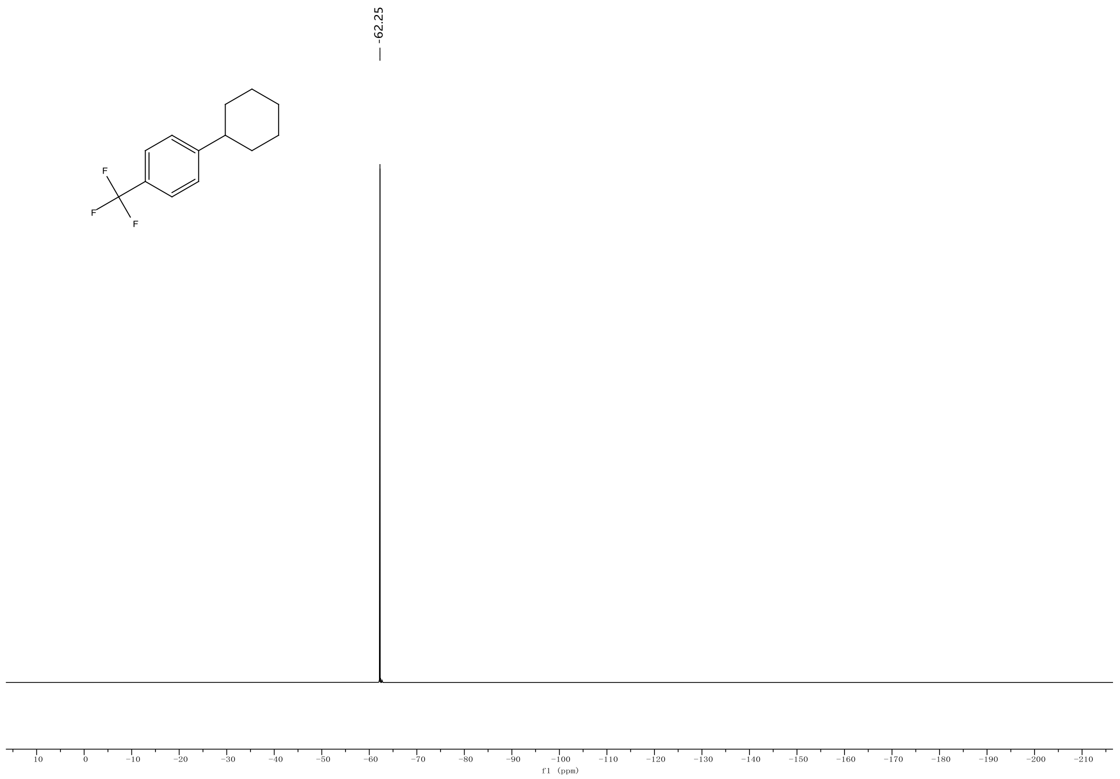

Supplementary Figure 35. ^{19}F NMR spectra of compound **1** (565 MHz, r.t., $CDCl_3$).

Compound 2

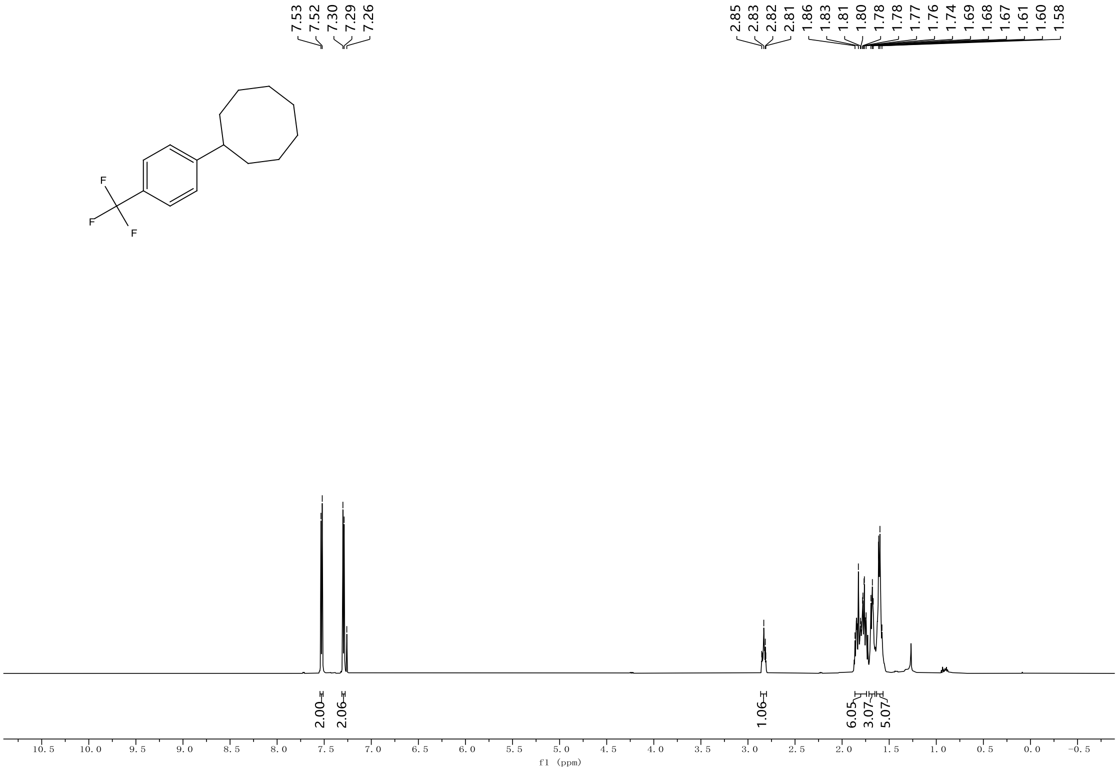

Supplementary Figure 36. ^{1}H NMR spectra of compound **2** (600 MHz, r.t., $CDCl_3$).

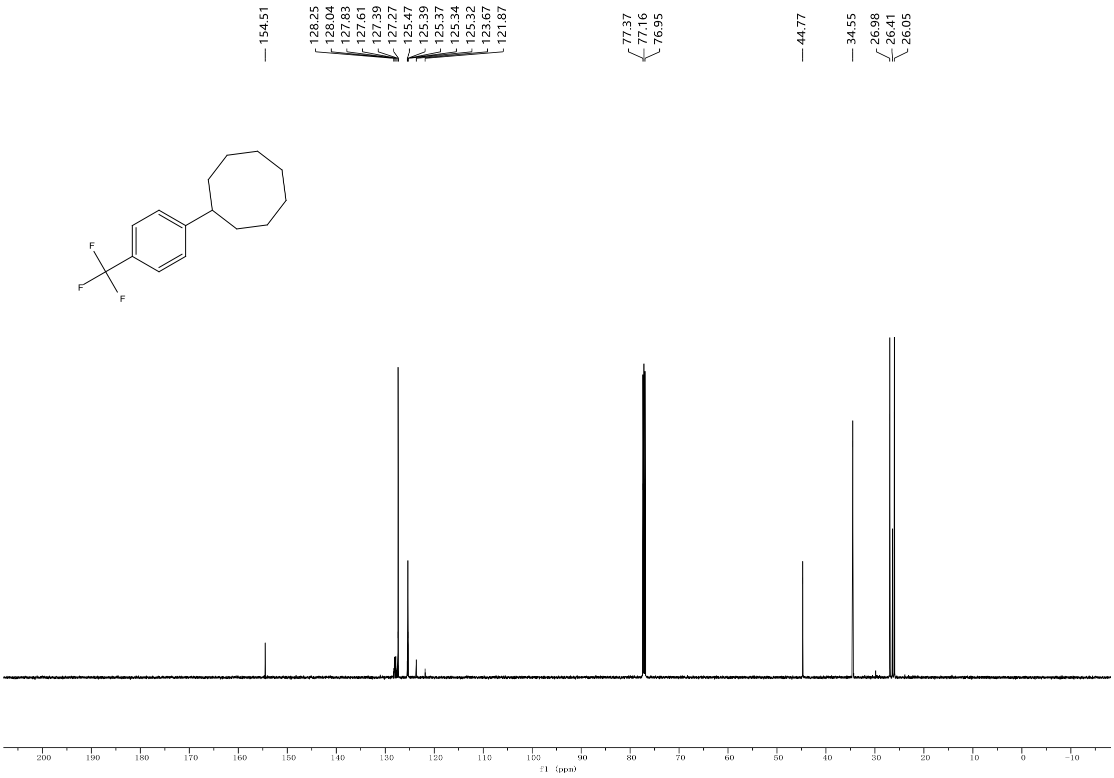

Supplementary Figure 37. ^{13}C NMR spectra of compound **2** (151 MHz, r.t., $CDCl_3$).

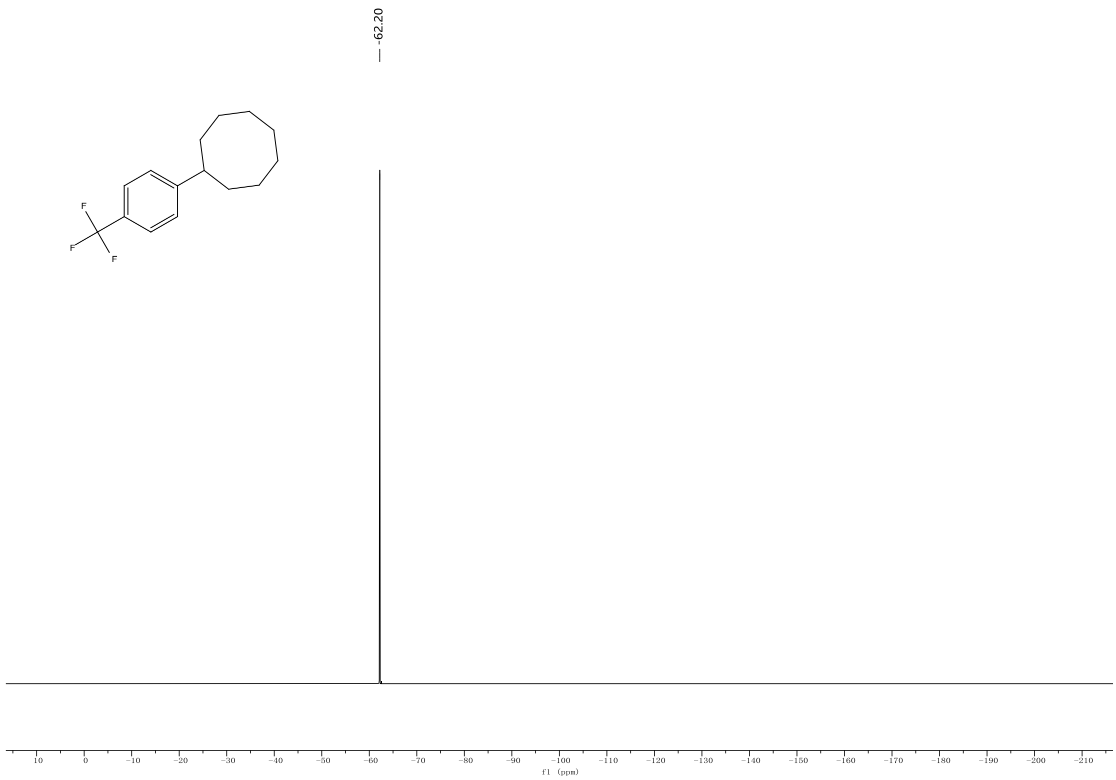

Supplementary Figure 38. ^{19}F NMR spectra of compound **2** (565 MHz, r.t., $CDCl_3$).

Compound 3

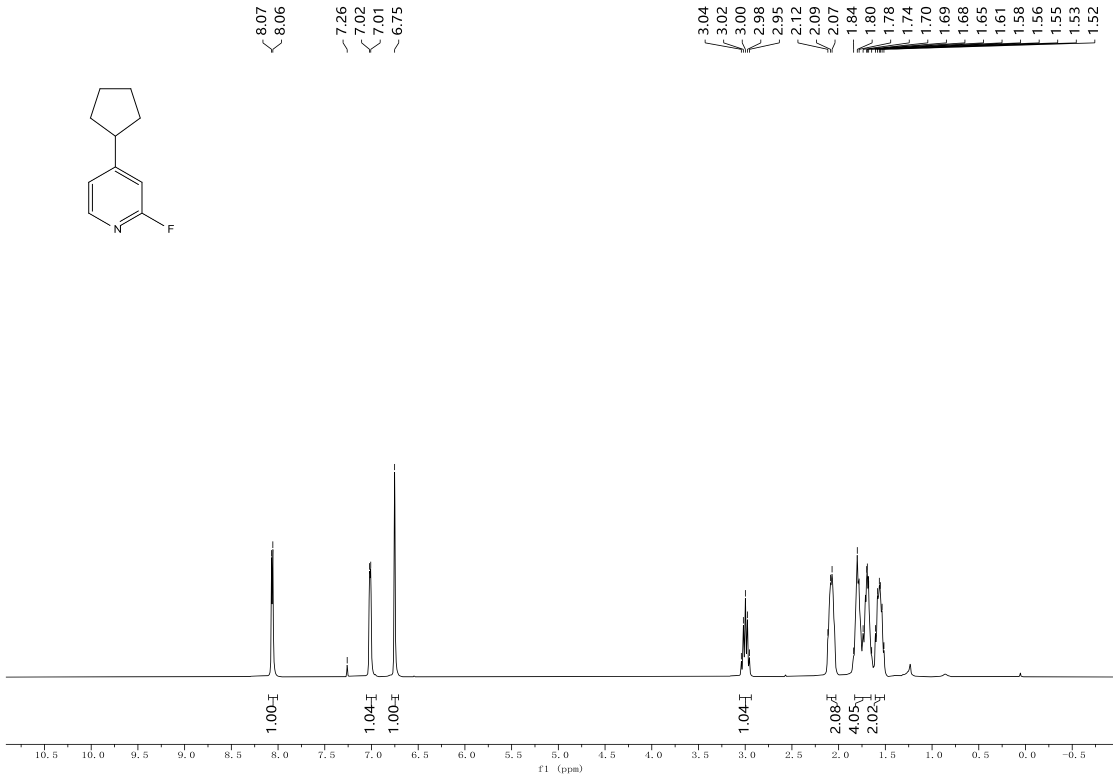

Supplementary Figure 39. ^{1}H NMR spectra of compound **3** (400 MHz, r.t., $CDCl_3$).

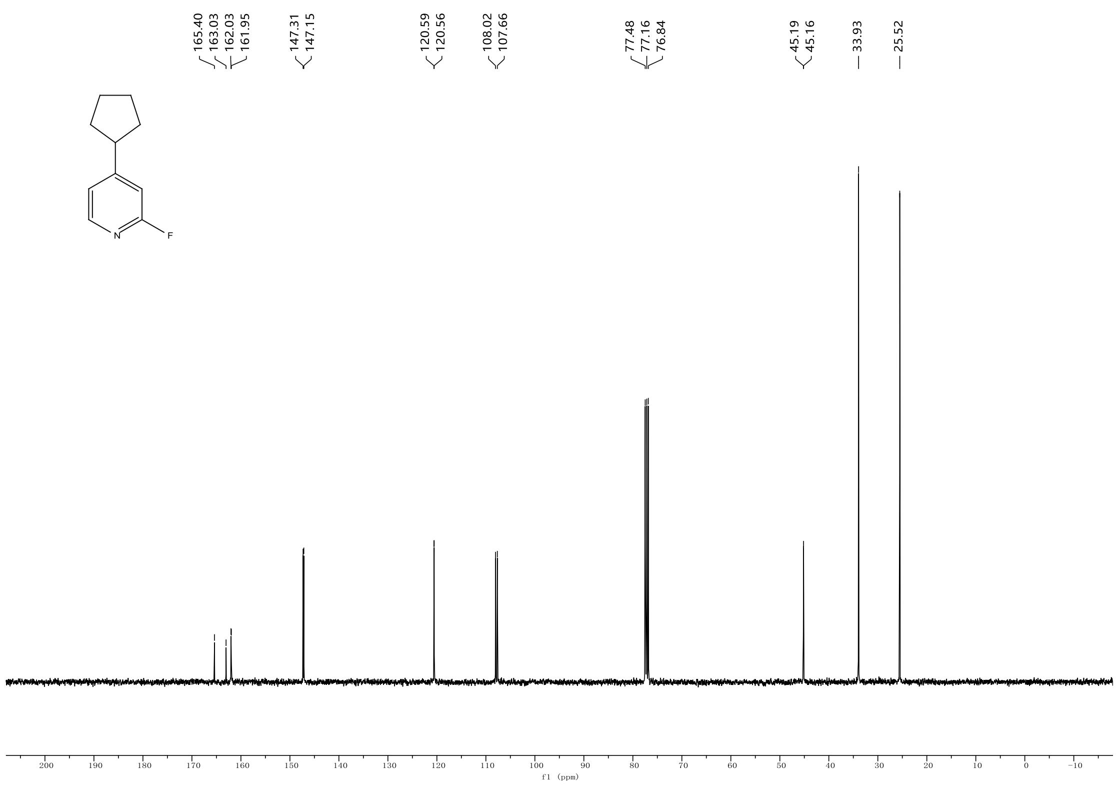

Supplementary Figure 40. ^{13}C NMR spectra of compound **3** (101 MHz, r.t., $CDCl_3$).

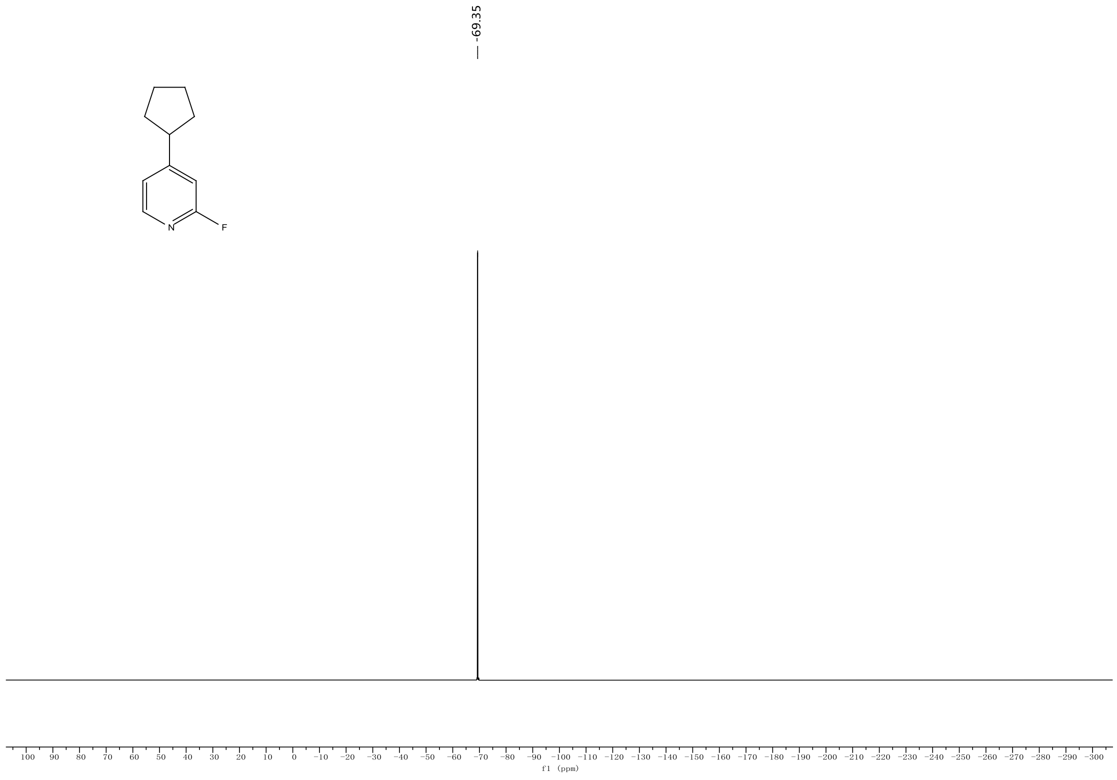

Supplementary Figure 41. ^{19}F NMR spectra of compound **3** (376 MHz, r.t., $CDCl_3$).

Compound 4

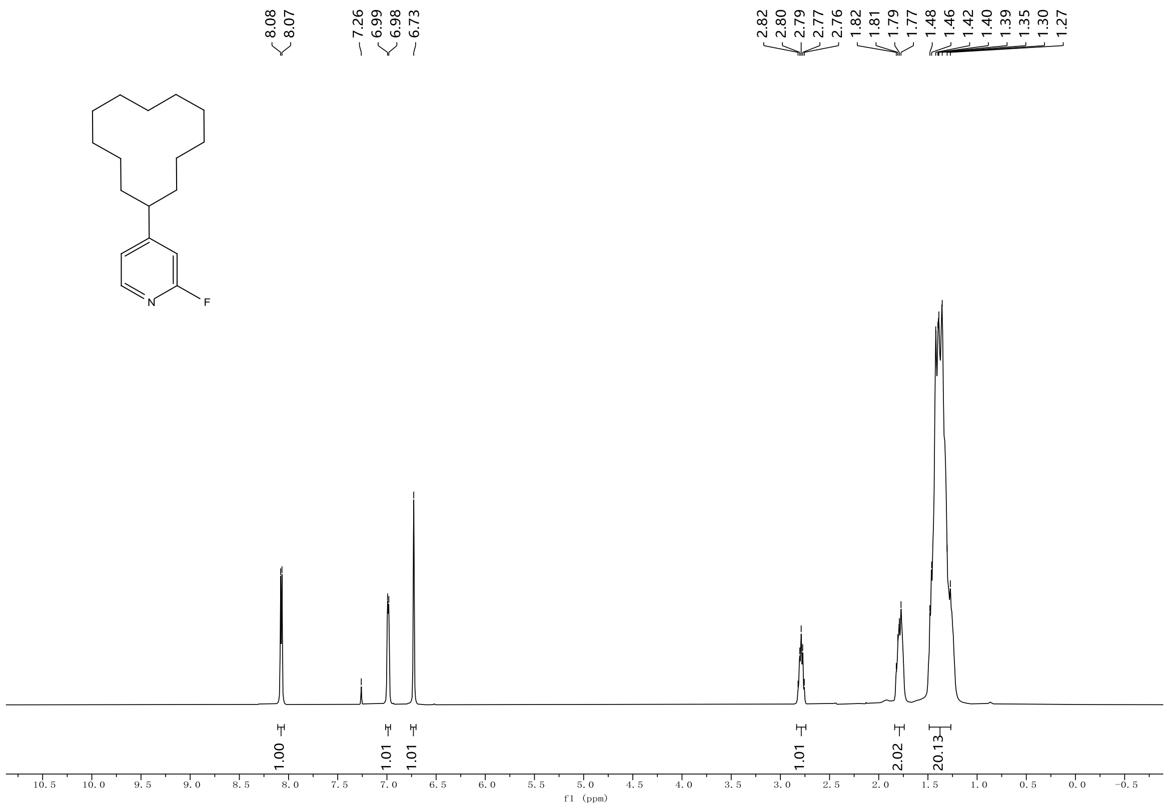

Supplementary Figure 42. ^{1}H NMR spectra of compound **4** (400 MHz, r.t., $CDCl_3$).

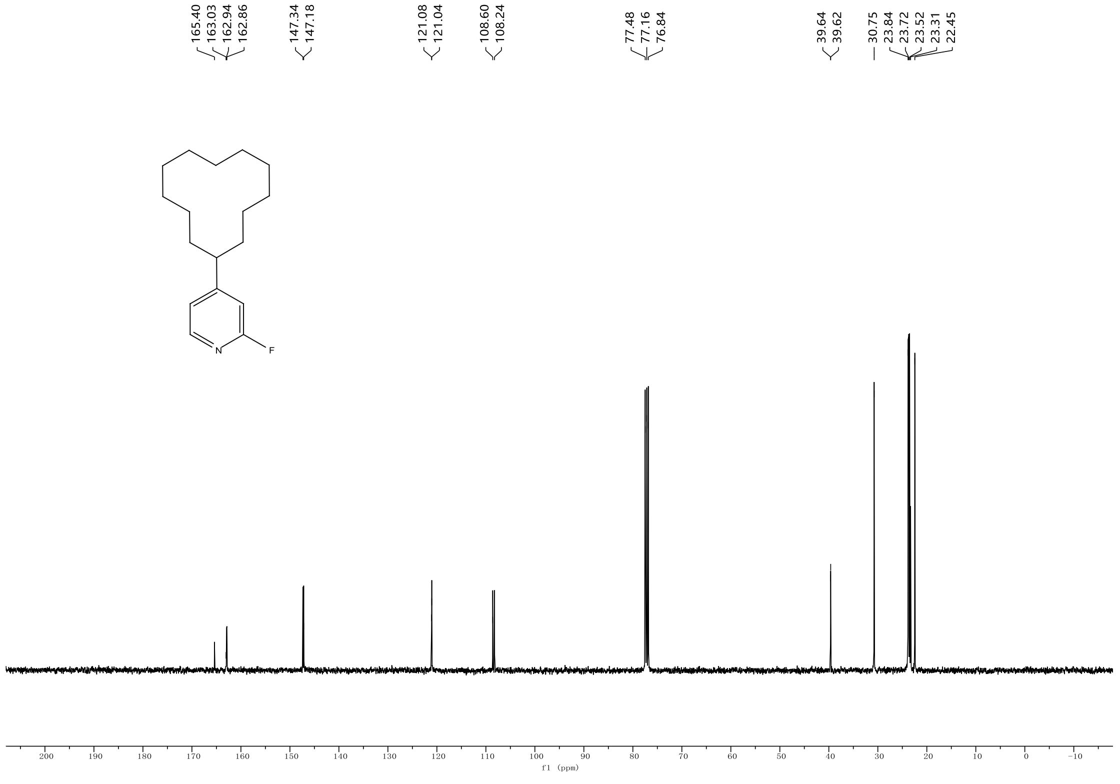

Supplementary Figure 43. ^{13}C NMR spectra of compound **4** (101 MHz, r.t., $CDCl_3$).

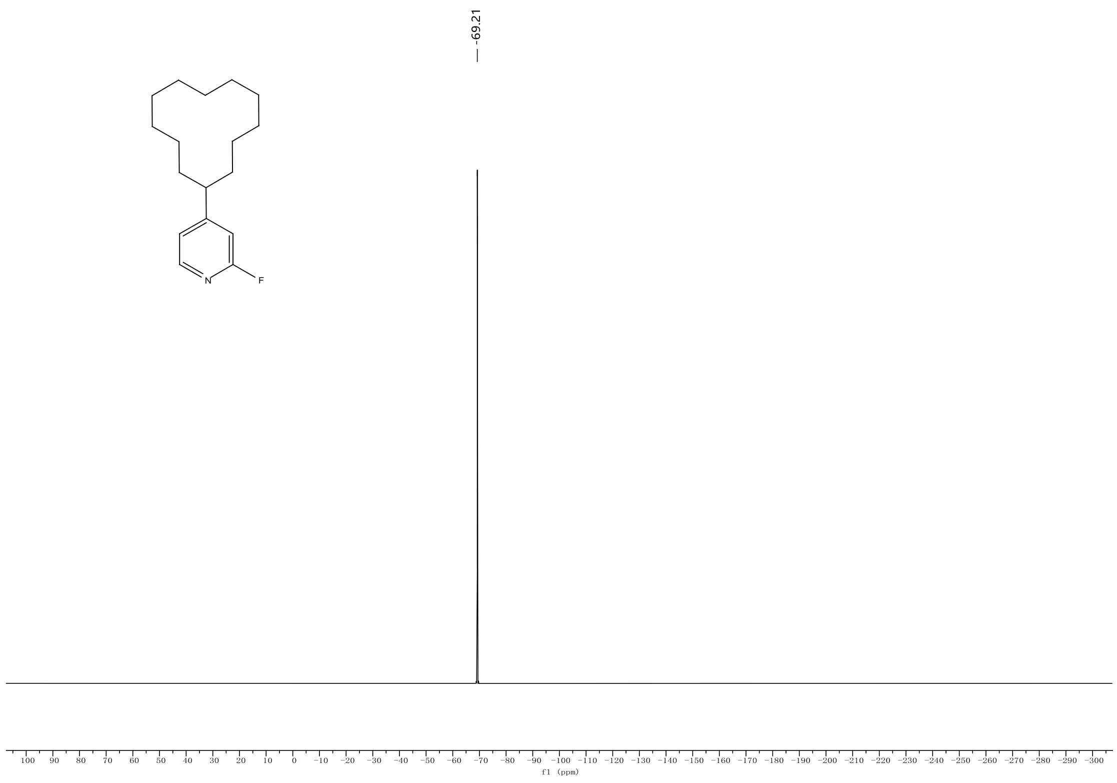

Supplementary Figure 44. ^{19}F NMR spectra of compound **4** (376 MHz, r.t., $CDCl_3$).

Compound 5

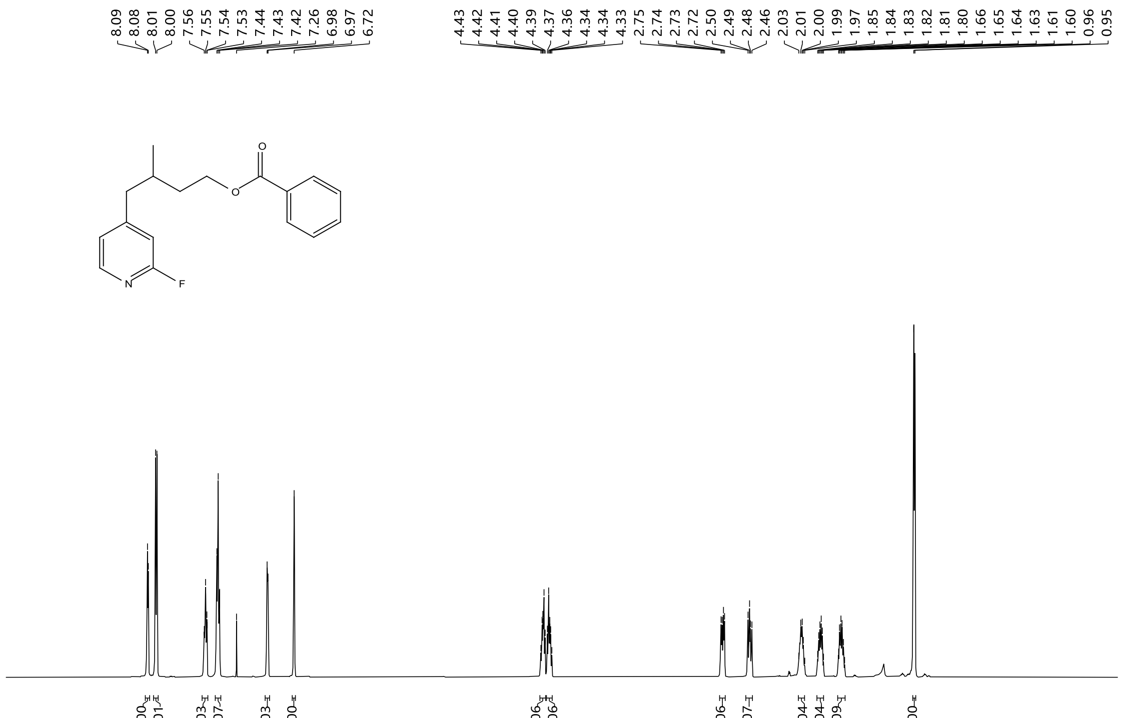

Supplementary Figure 45. ^{1}H NMR spectra of compound **5** (600 MHz, r.t., $CDCl_3$).

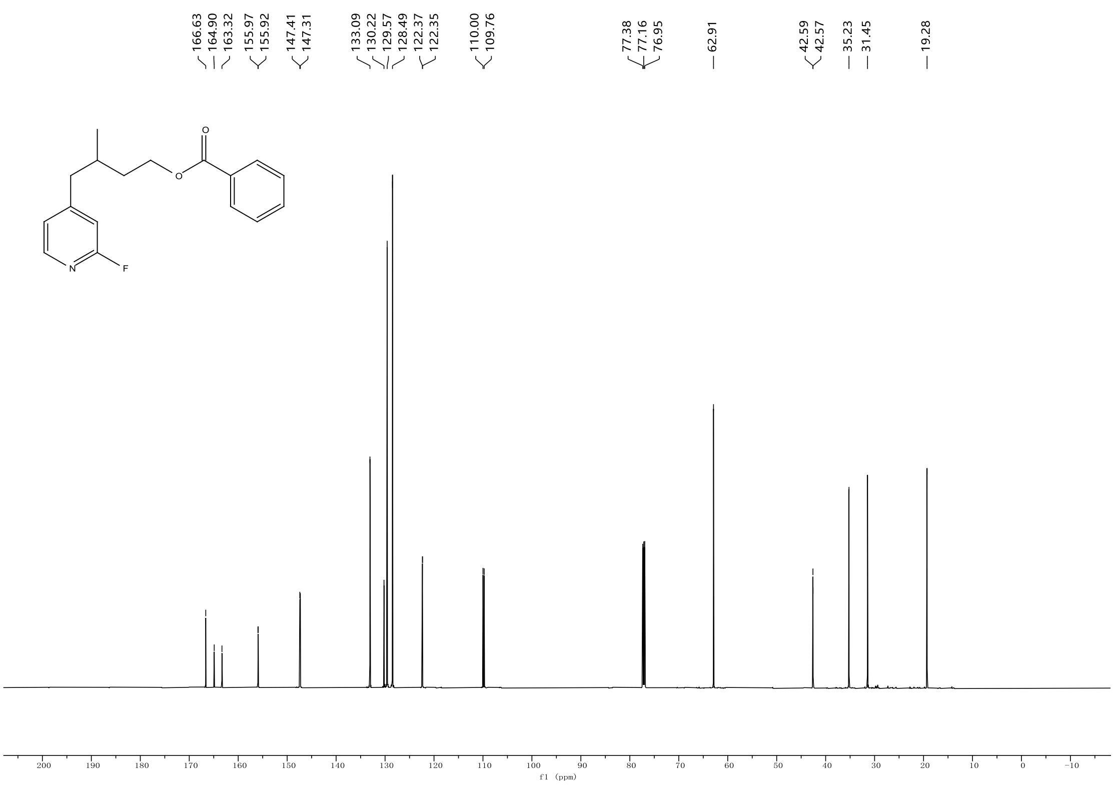

Supplementary Figure 46. ^{13}C NMR spectra of compound **5** (151 MHz, r.t., $CDCl_3$).

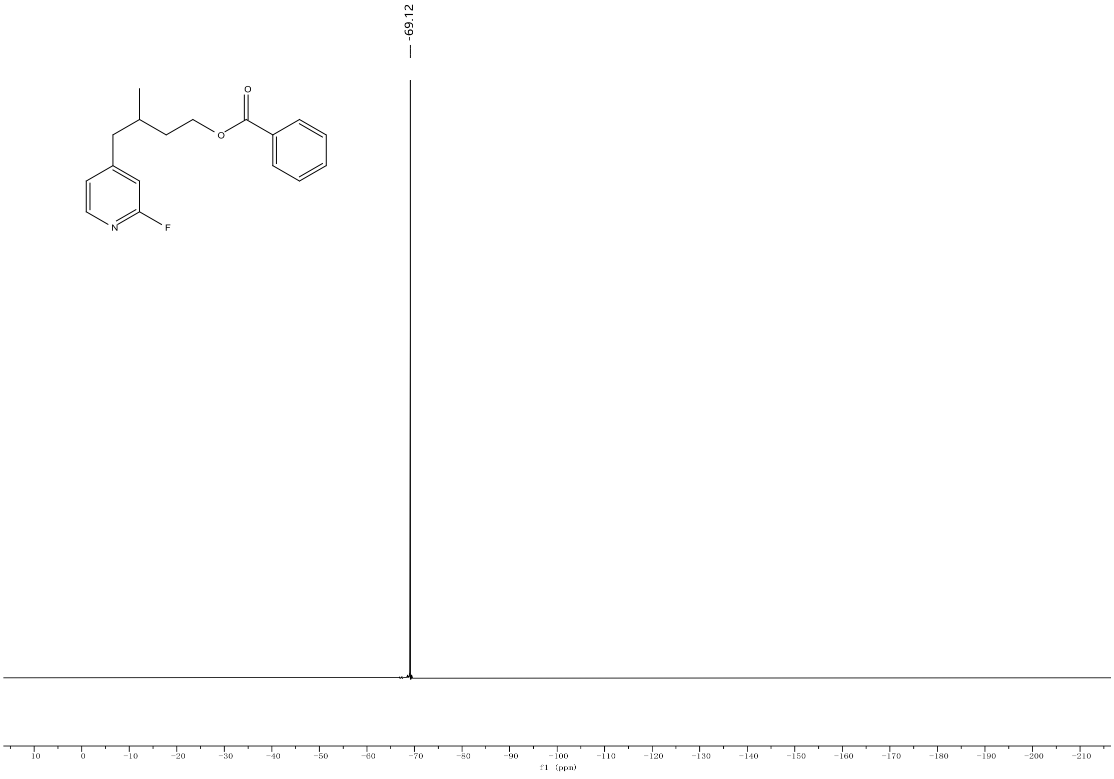

Supplementary Figure 47. ^{19}F NMR spectra of compound **5** (565 MHz, r.t., $CDCl_3$).

Compound 6

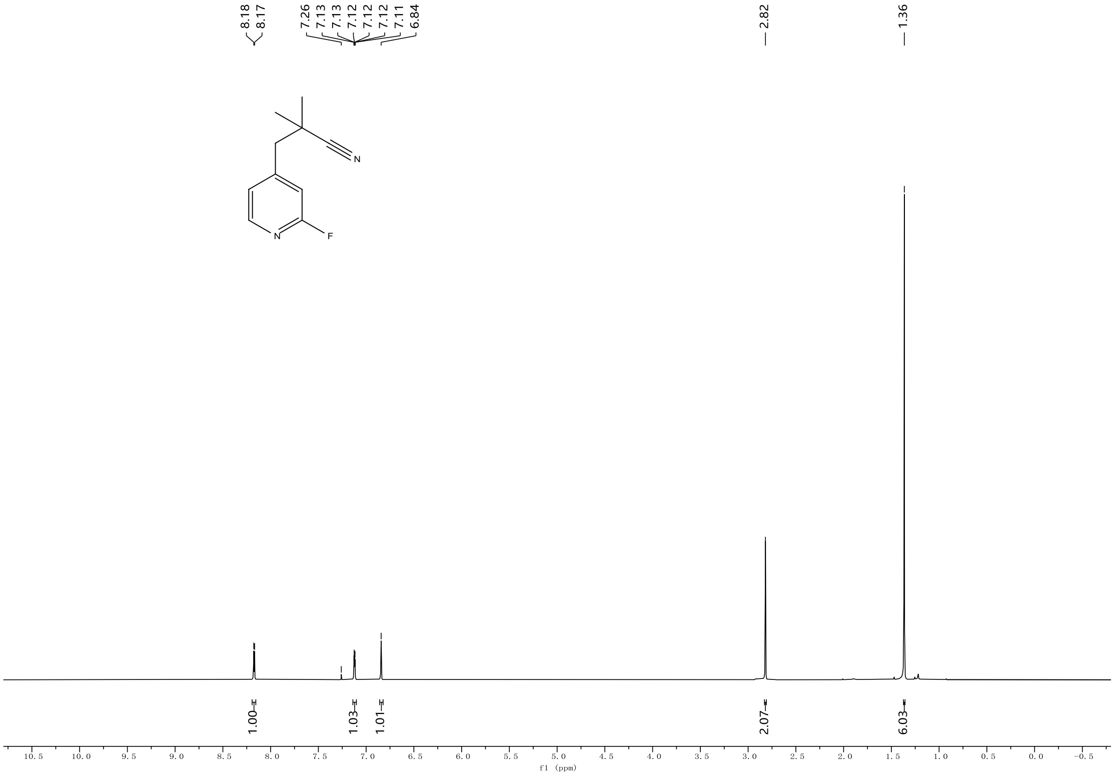

Supplementary Figure 48. ^{1}H NMR spectra of compound **6** (600 MHz, r.t., $CDCl_3$).

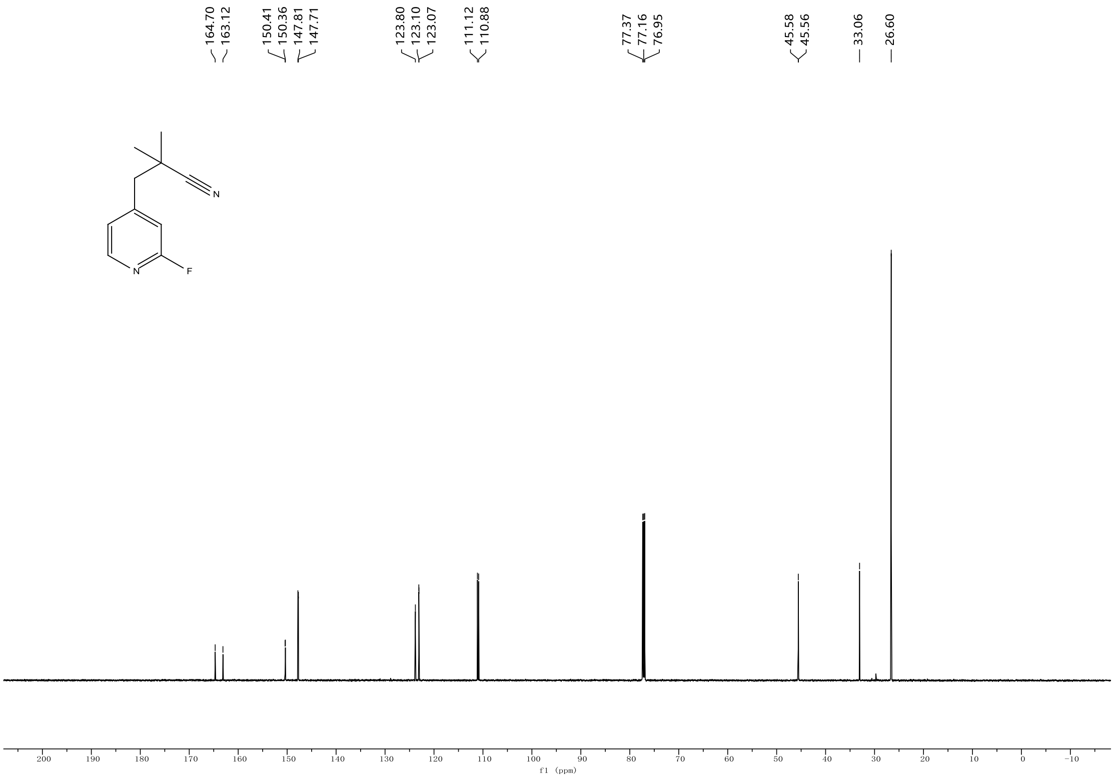

Supplementary Figure 49. ^{13}C NMR spectra of compound **6** (151 MHz, r.t., $CDCl_3$).

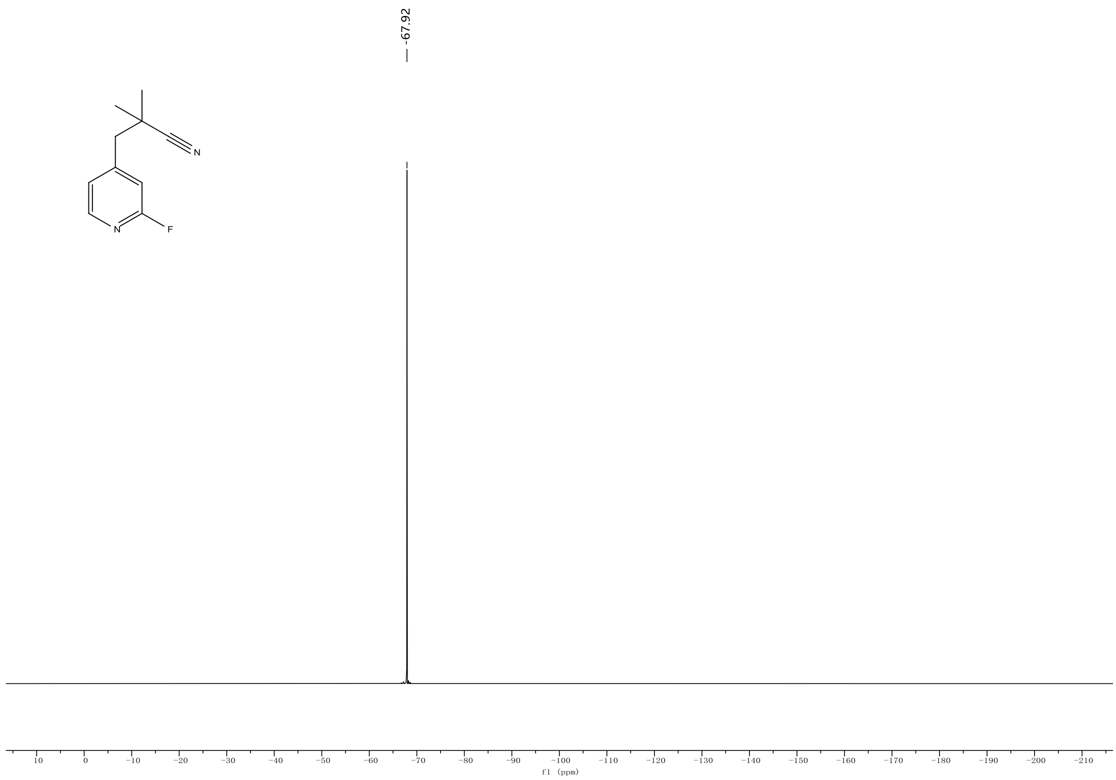

Supplementary Figure 50. ^{19}F NMR spectra of compound **6** (565 MHz, r.t., $CDCl_3$).

Compound 7

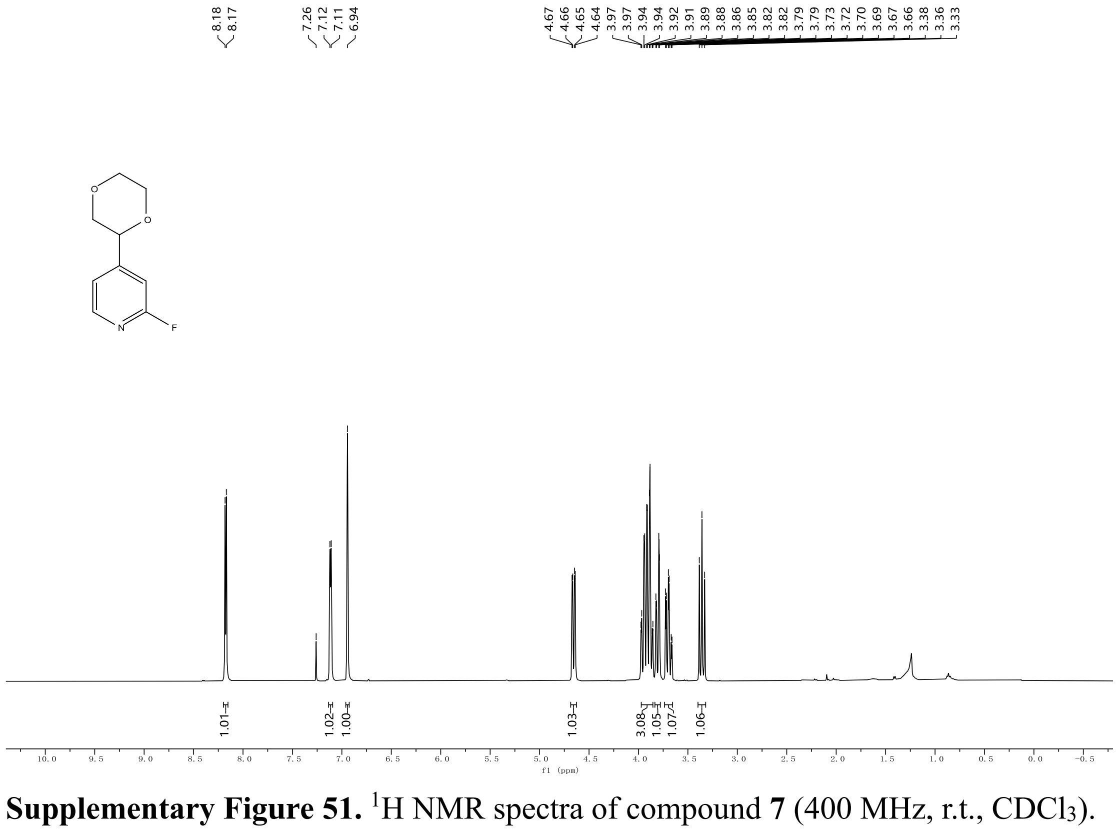

Supplementary Figure 51. ^{1}H NMR spectra of compound **7** (400 MHz, r.t., $CDCl_3$).

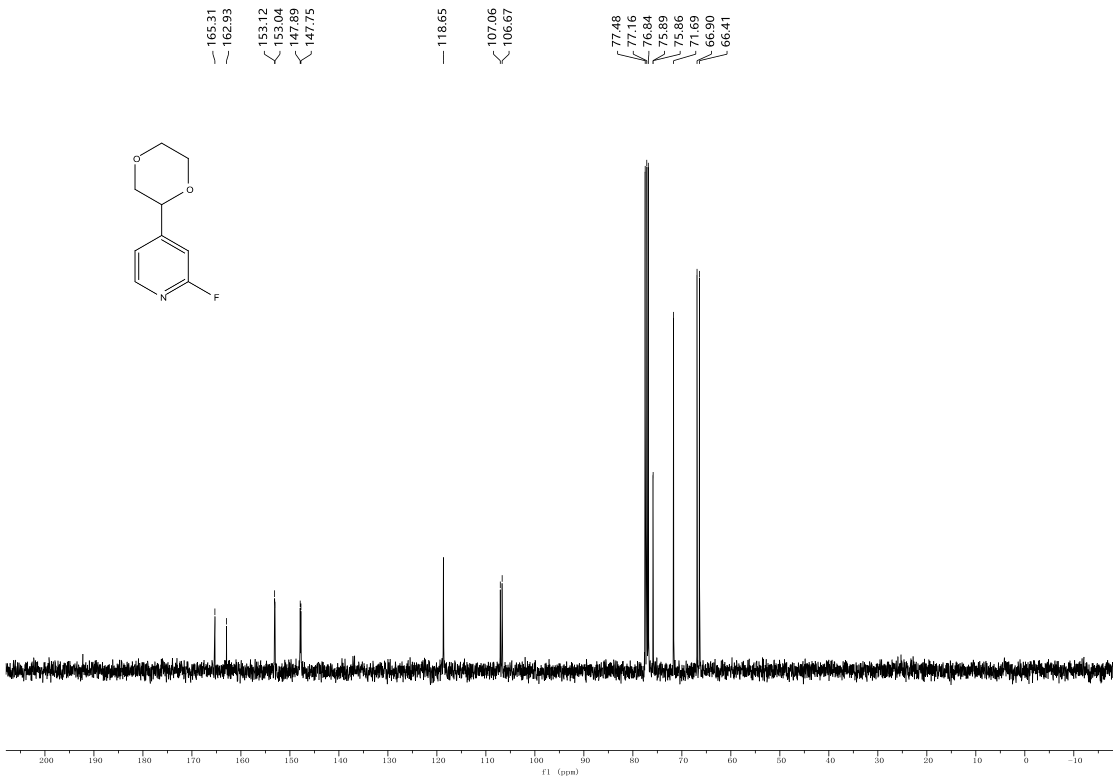

Supplementary Figure 52. ^{13}C NMR spectra of compound **7** (101 MHz, r.t., $CDCl_3$).

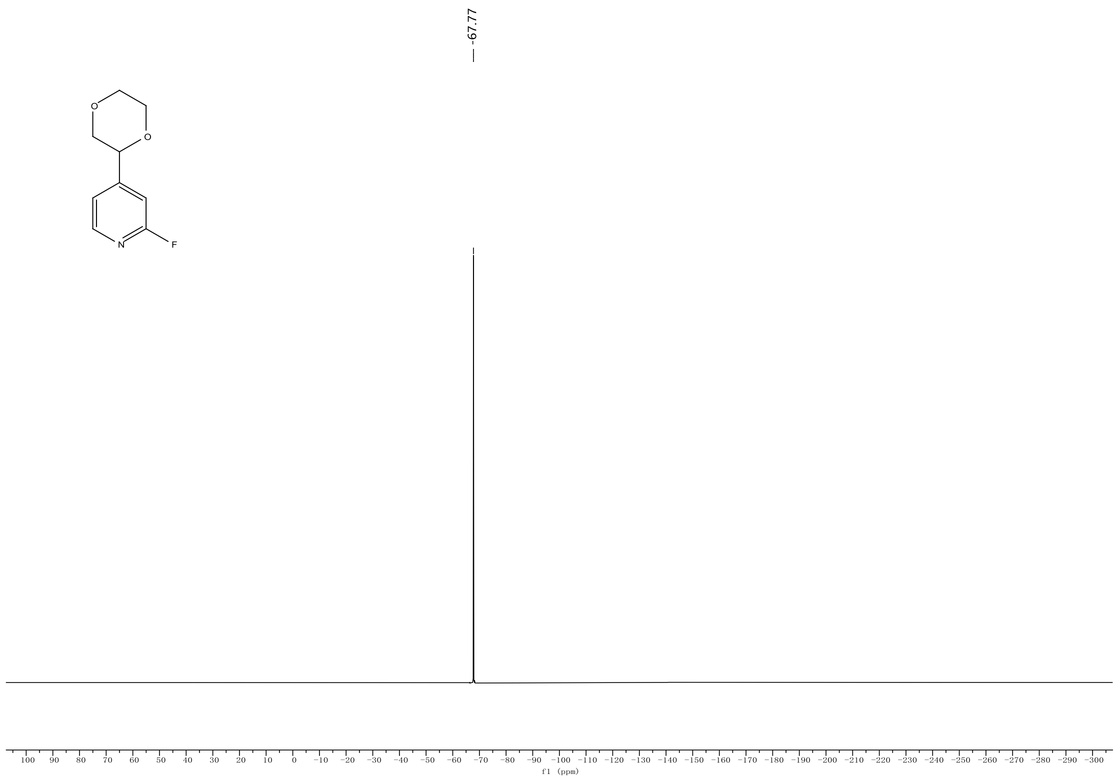

Supplementary Figure 53. ^{19}F NMR spectra of compound **7** (376 MHz, r.t., $CDCl_3$).

Compound 8

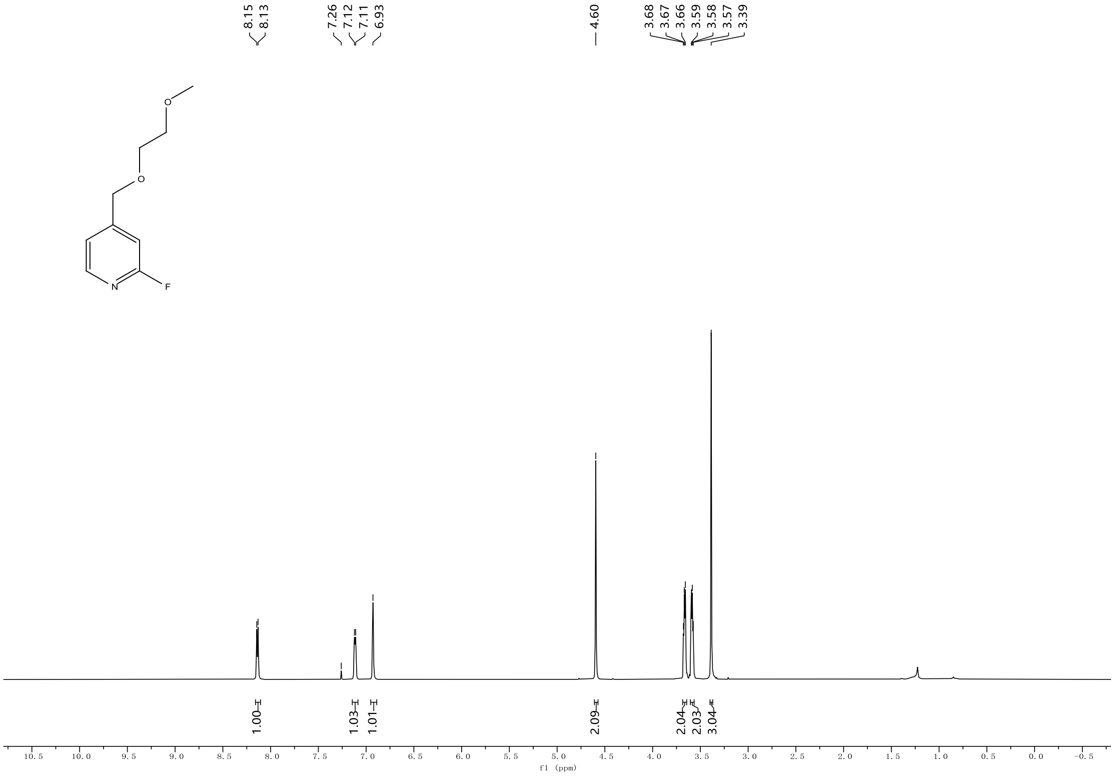

Supplementary Figure 54. ^{1}H NMR spectra of compound **8** (400 MHz, r.t., $CDCl_3$).

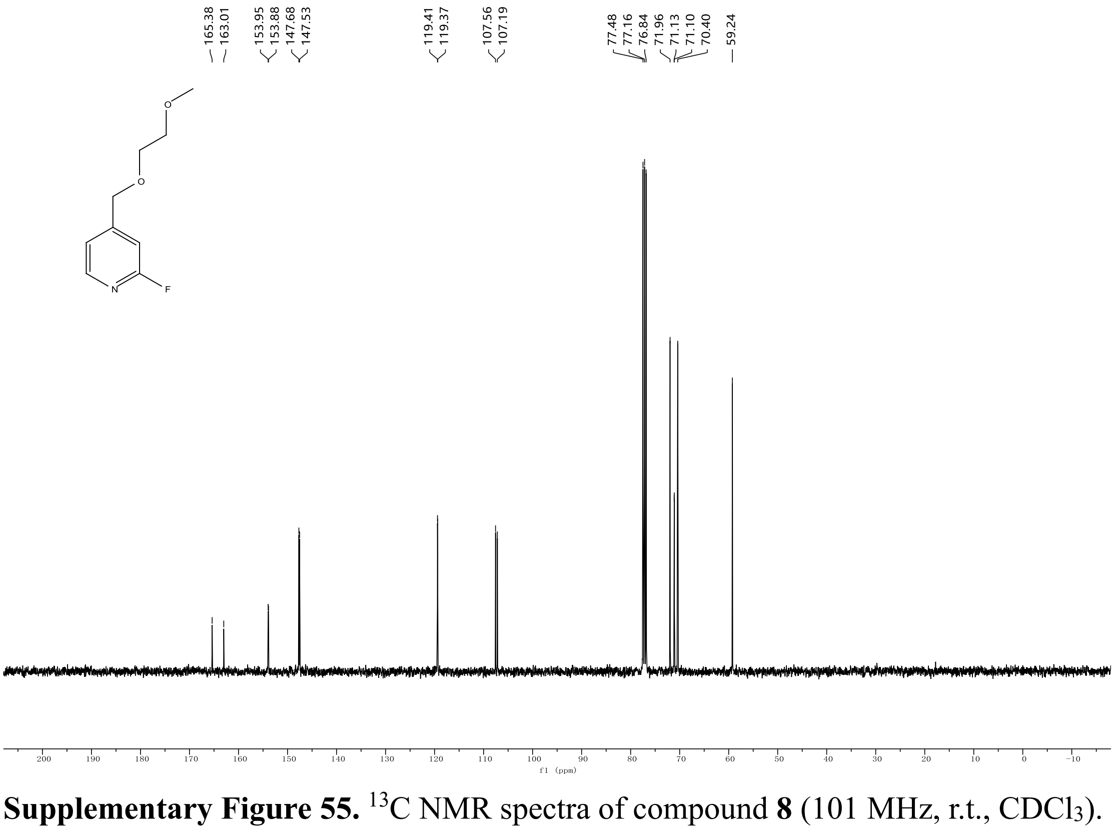

Supplementary Figure 55. ^{13}C NMR spectra of compound **8** (101 MHz, r.t., $CDCl_3$).

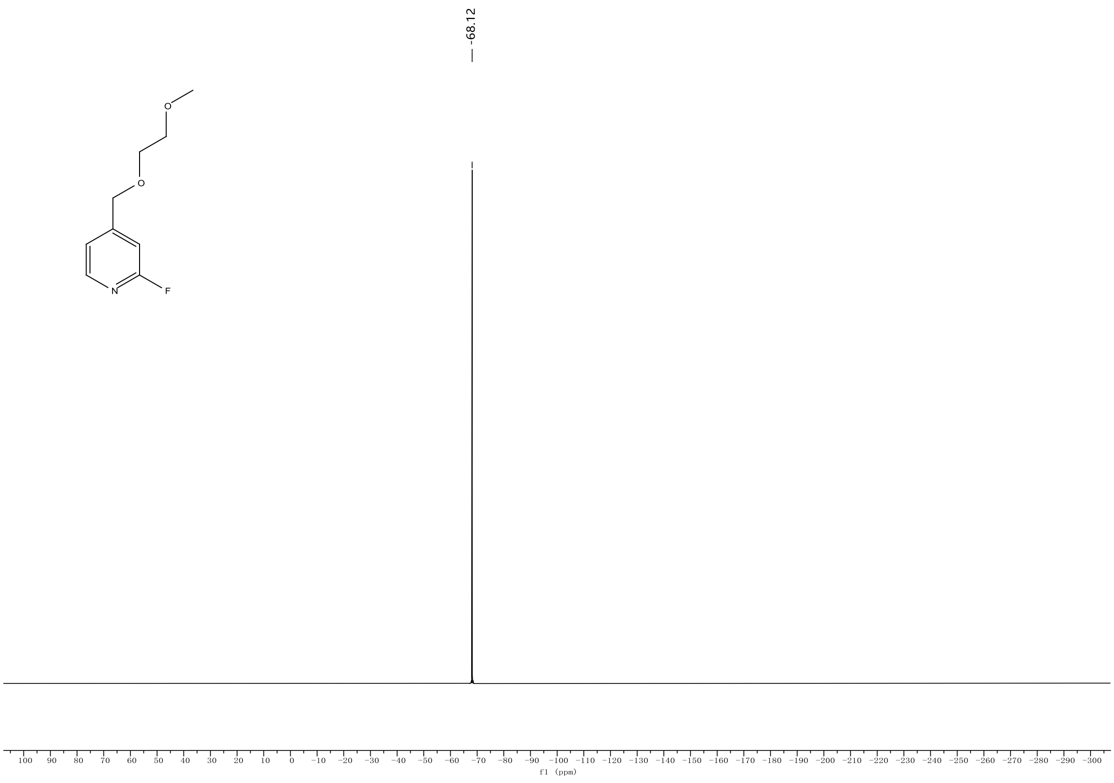

Supplementary Figure 56. ^{19}F NMR spectra of compound **8** (376 MHz, r.t., $CDCl_3$).

Compound 9

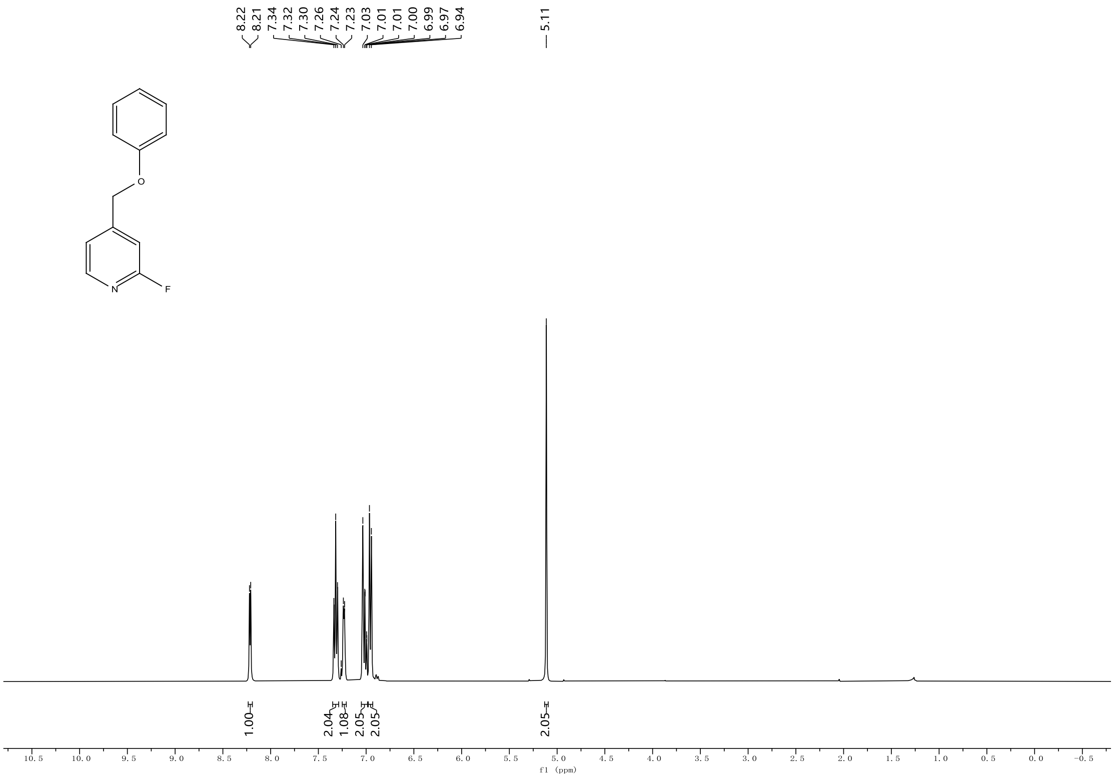

Supplementary Figure 57. ^{1}H NMR spectra of compound **9** (400 MHz, r.t., $CDCl_3$).

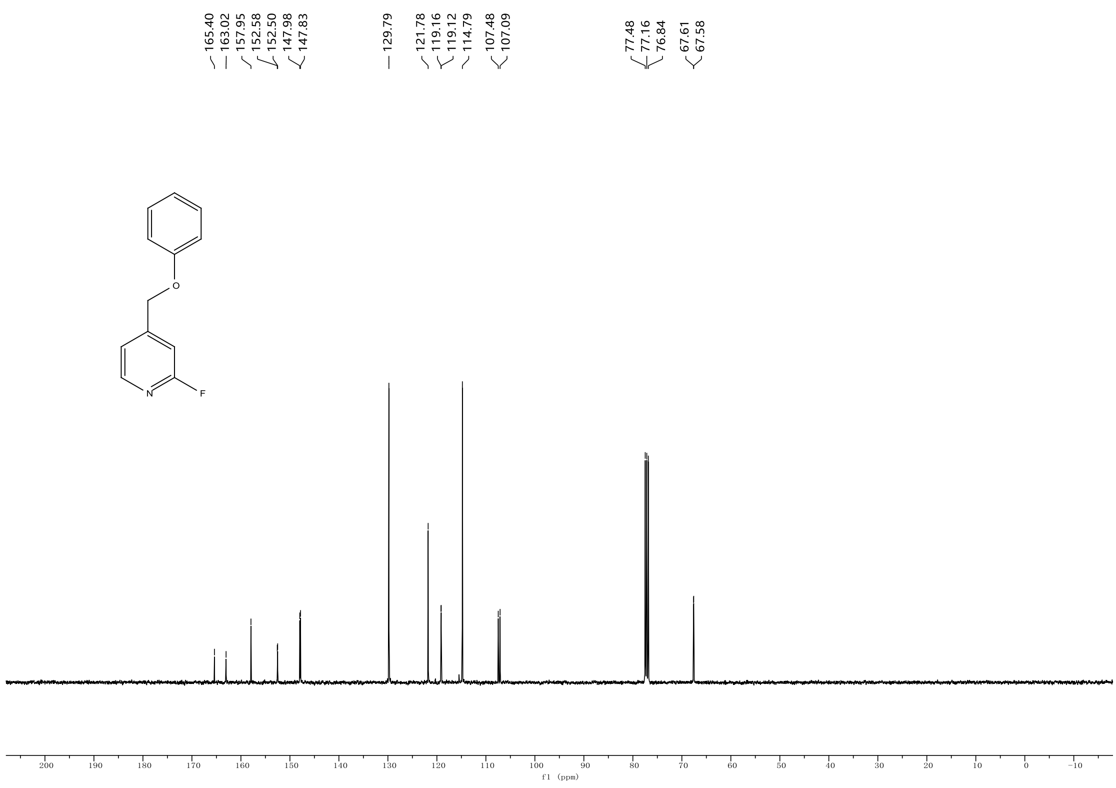

Supplementary Figure 58. ^{13}C NMR spectra of compound **9** (101 MHz, r.t., $CDCl_3$).

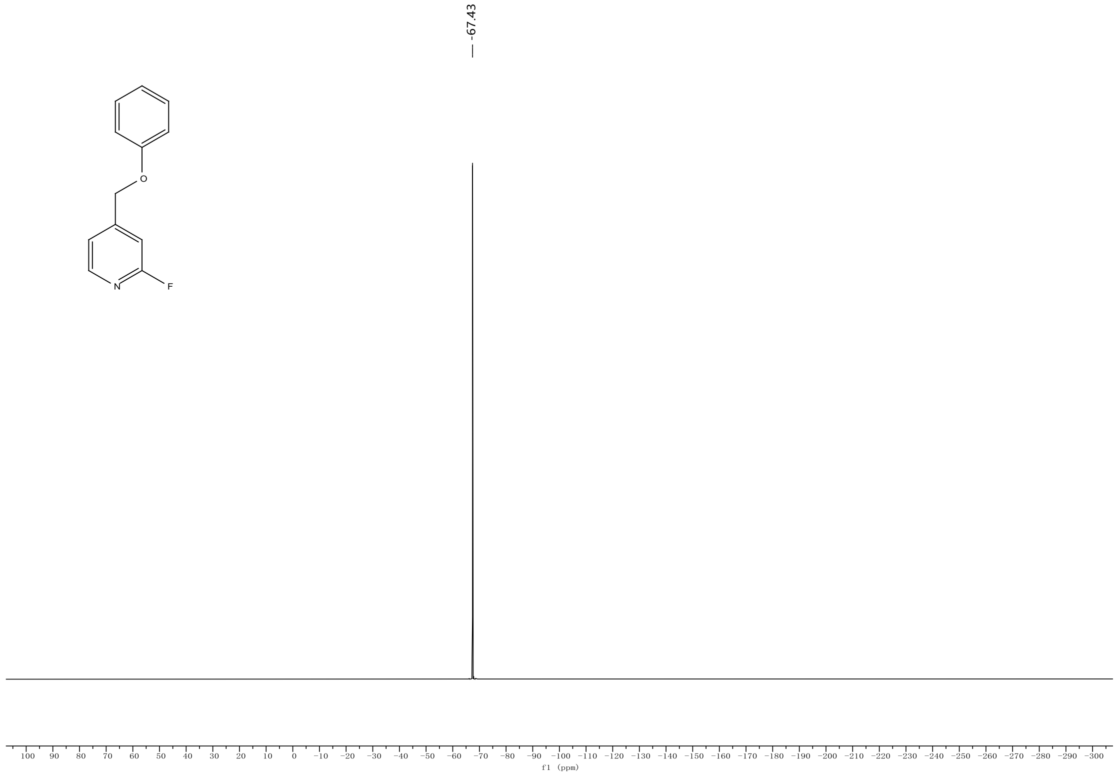

Supplementary Figure 59. ^{19}F NMR spectra of compound **9** (376 MHz, r.t., $CDCl_3$).

Compound 10

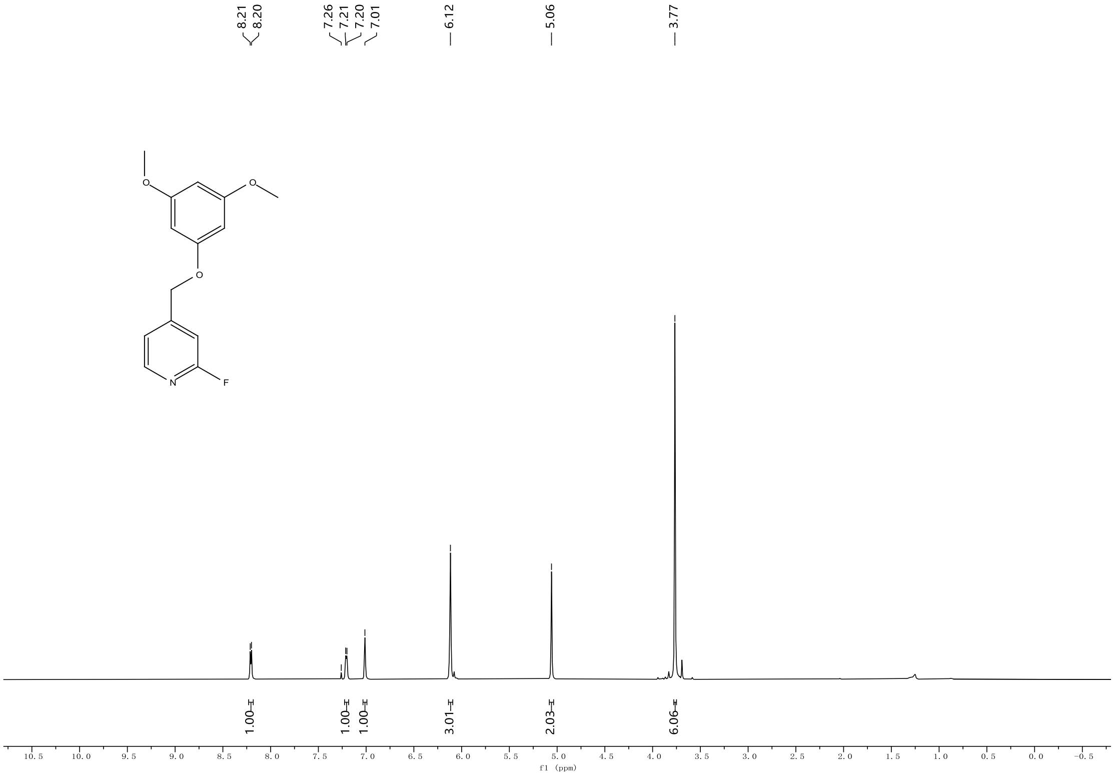

Supplementary Figure 60. ^{1}H NMR spectra of compound **10** (400 MHz, r.t., $CDCl_3$).

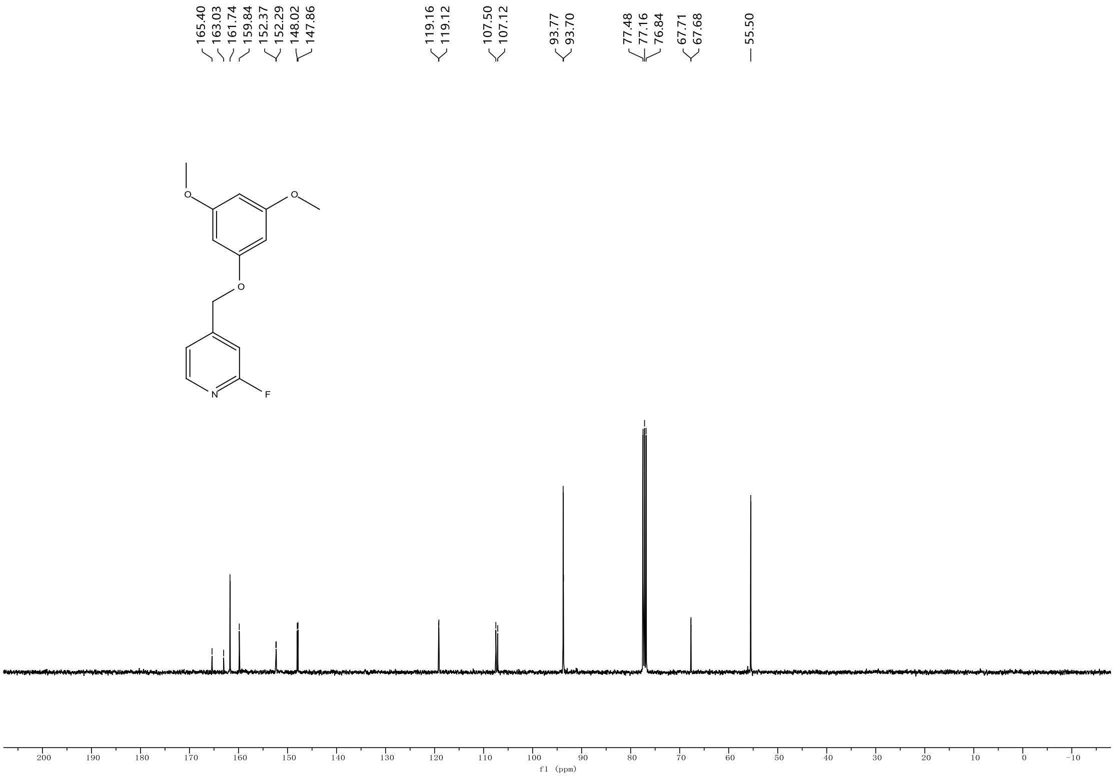

Supplementary Figure 61. ^{13}C NMR spectra of compound **10** (101 MHz, r.t., $CDCl_3$).

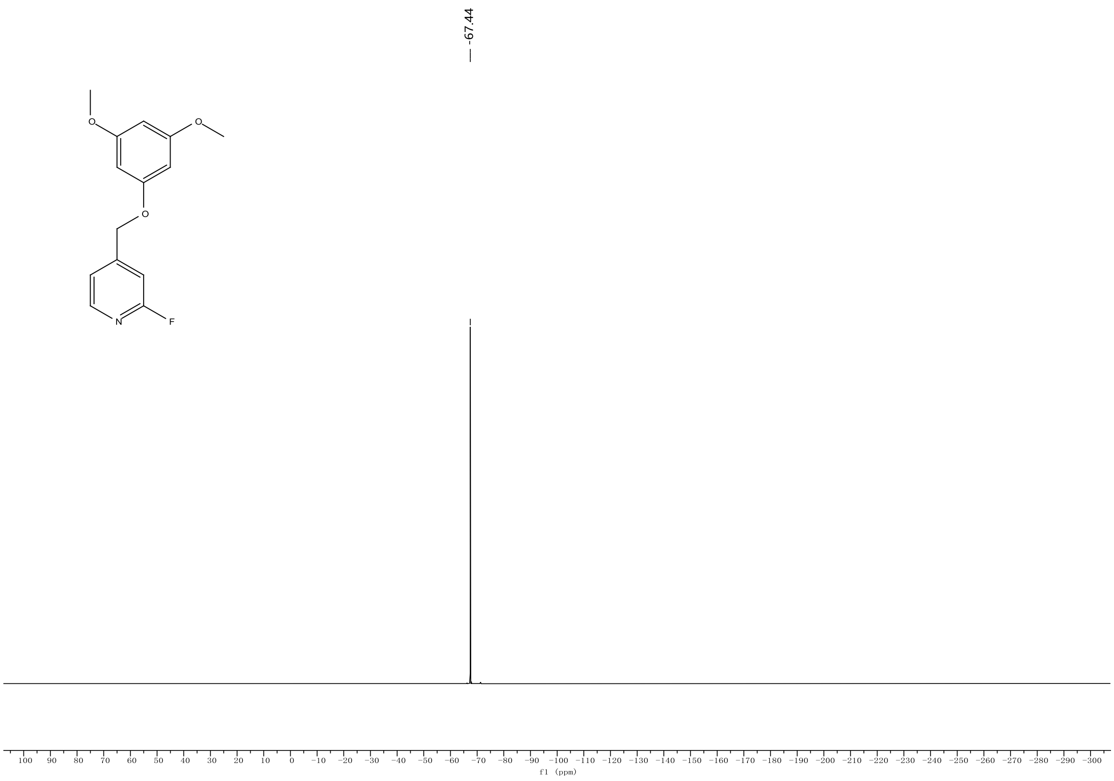

Supplementary Figure 62. ^{19}F NMR spectra of compound **10** (376 MHz, r.t., $CDCl_3$).

Compound 11 (major)

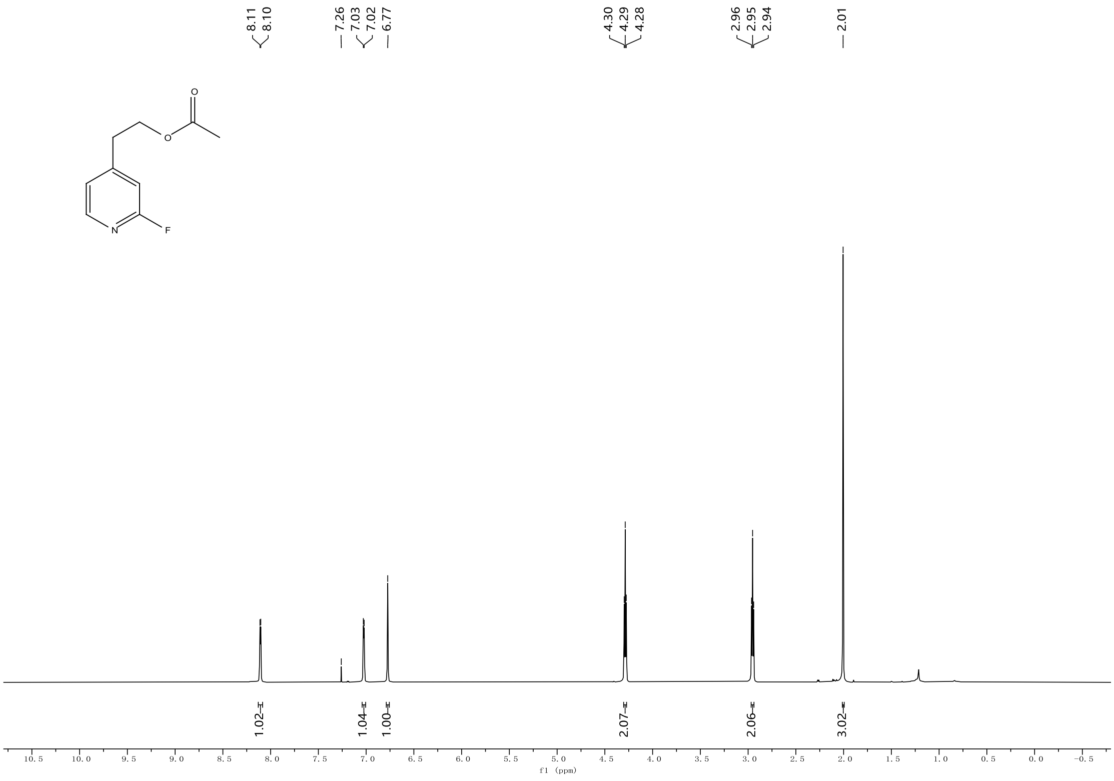

Supplementary Figure 63. ^{1}H NMR spectra of compound **11** (600 MHz, r.t., $CDCl_3$).

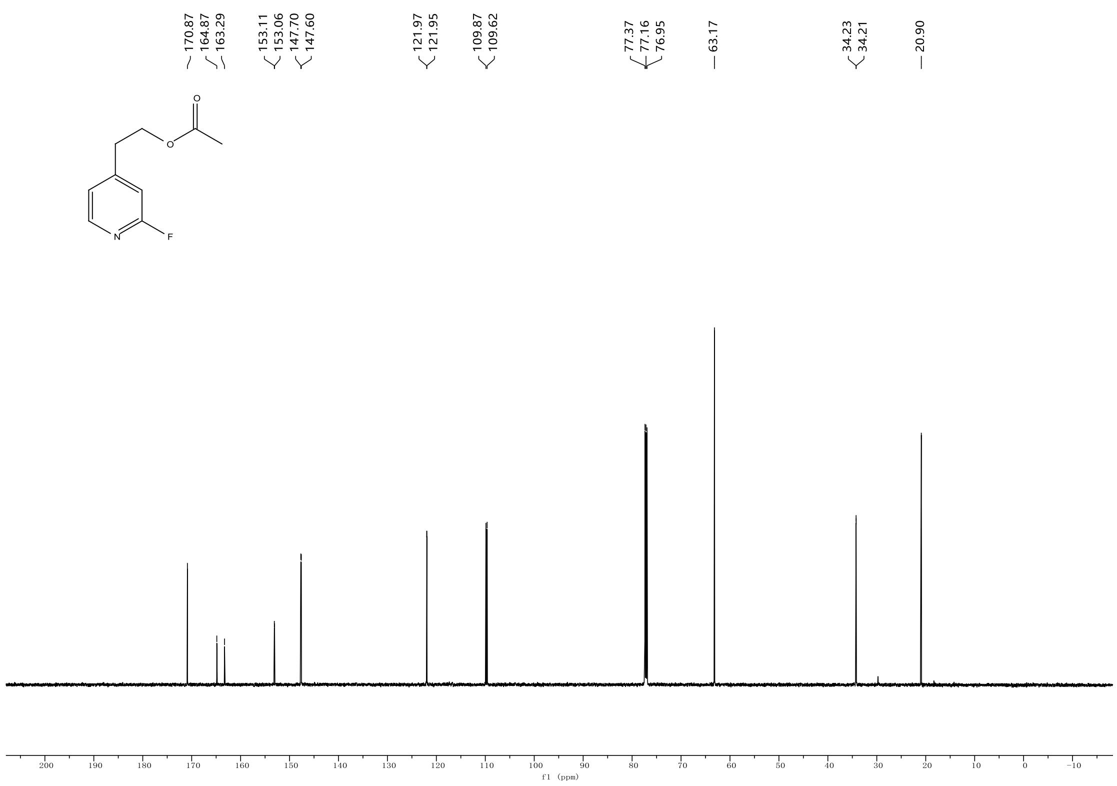

Supplementary Figure 64. ^{13}C NMR spectra of compound **11** (151 MHz, r.t., $CDCl_3$).

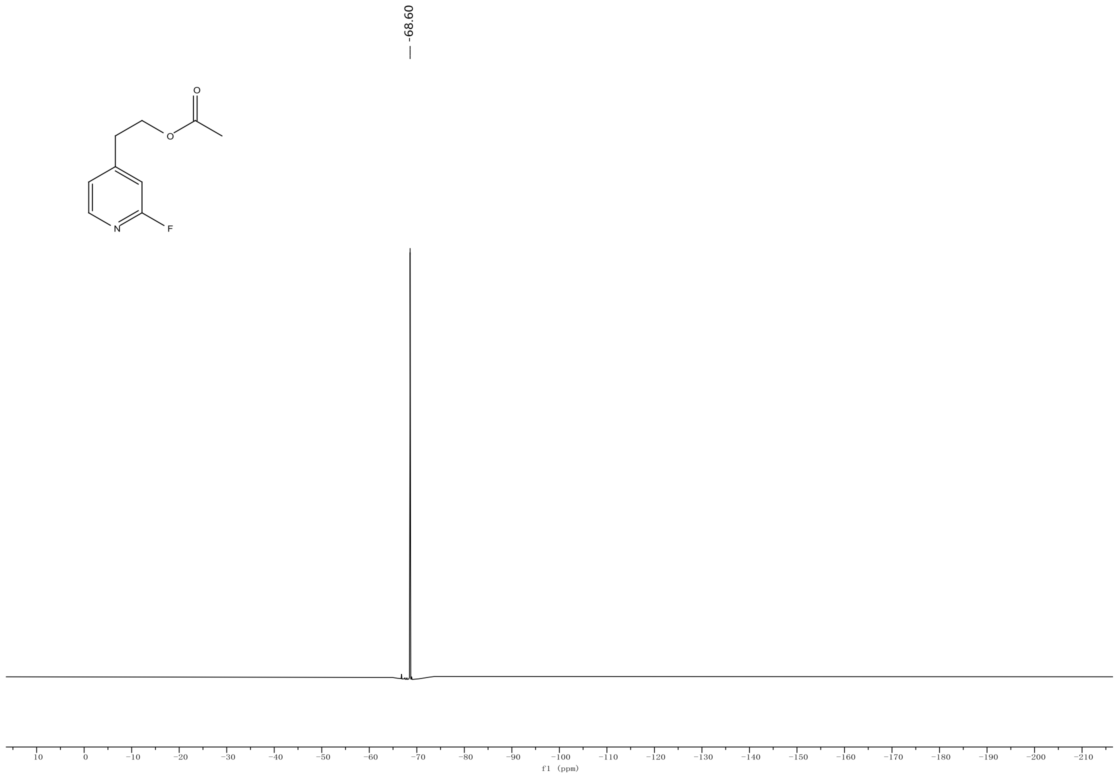

Supplementary Figure 65. ^{19}F NMR spectra of compound **11** (565 MHz, r.t., $CDCl_3$).

Compound 11` (minor)

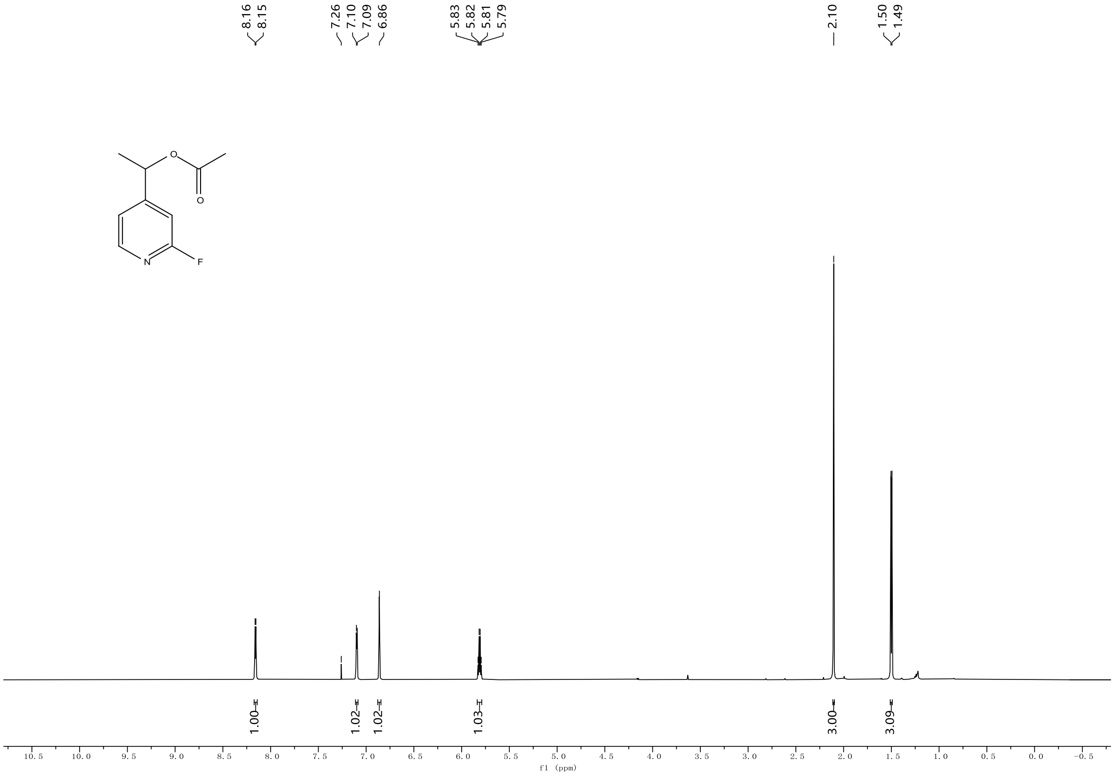

Supplementary Figure 66. ^{1}H NMR spectra of compound **11`** (600 MHz, r.t., $CDCl_3$).

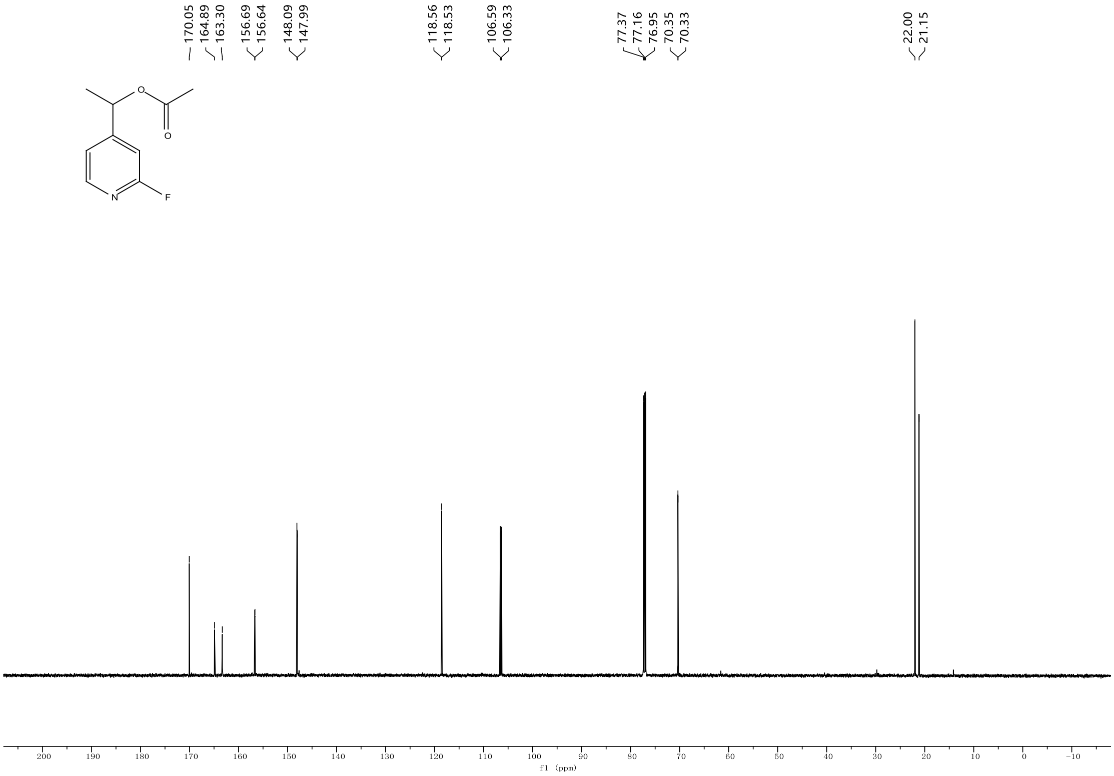

Supplementary Figure 67. ^{13}C NMR spectra of compound **11`** (151 MHz, r.t., $CDCl_3$).

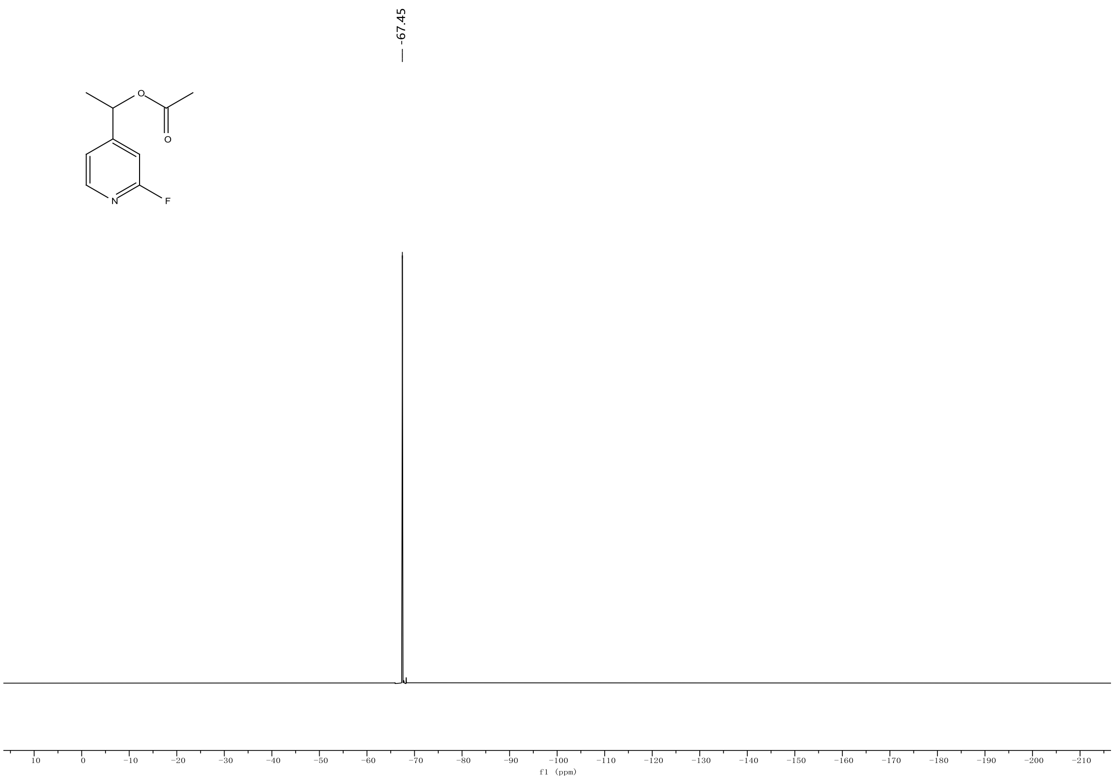

Supplementary Figure 68. ^{19}F NMR spectra of compound **11`** (565 MHz, r.t., $CDCl_3$).

Compound 12

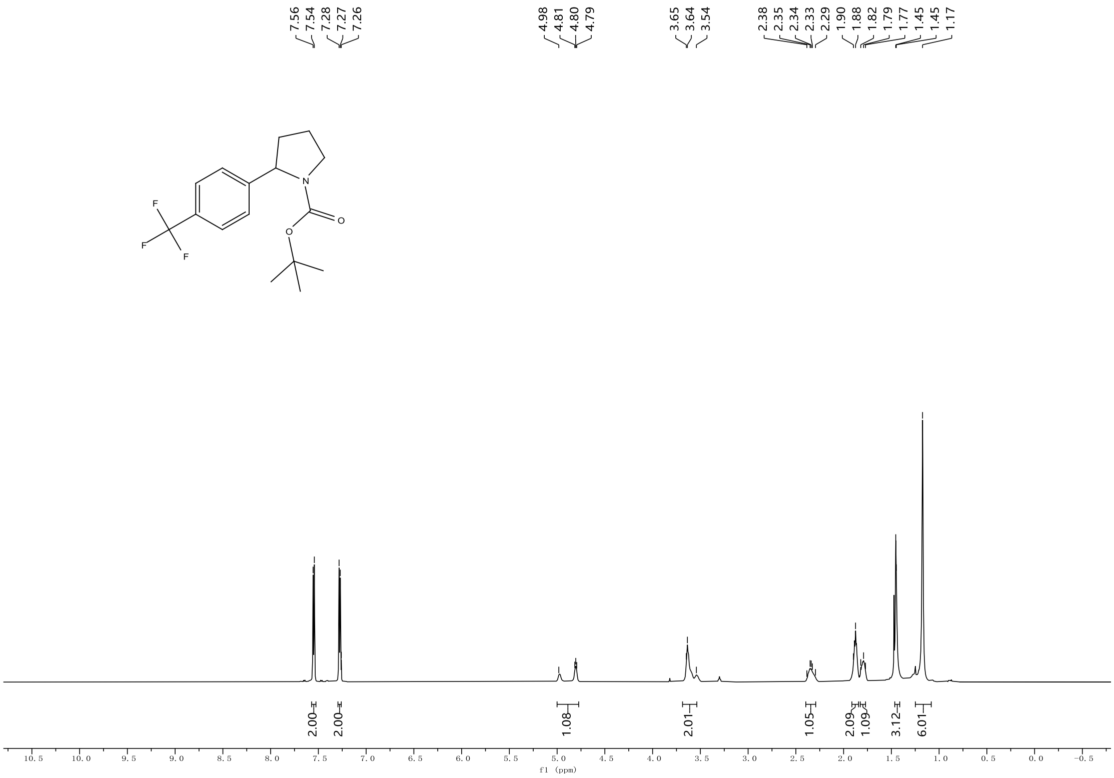

Supplementary Figure 69. ^{1}H NMR spectra of compound **12** (600 MHz, r.t., $CDCl_3$).

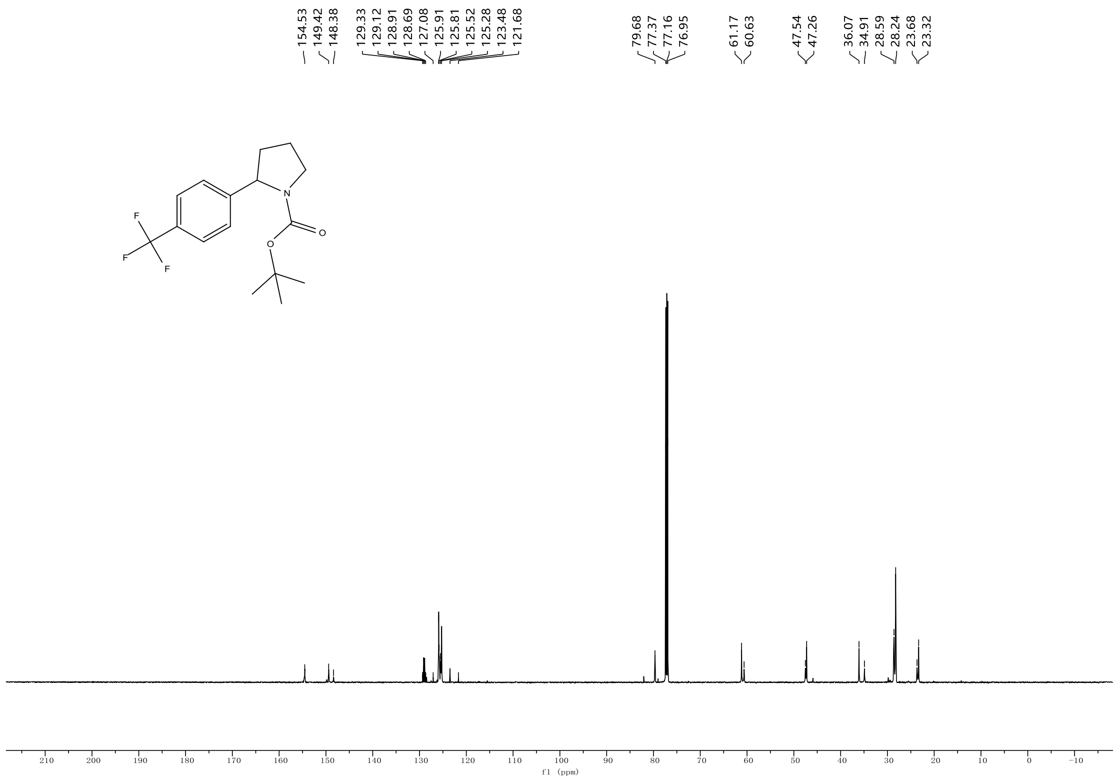

Supplementary Figure 70. ^{13}C NMR spectra of compound **12** (151 MHz, r.t., $CDCl_3$).

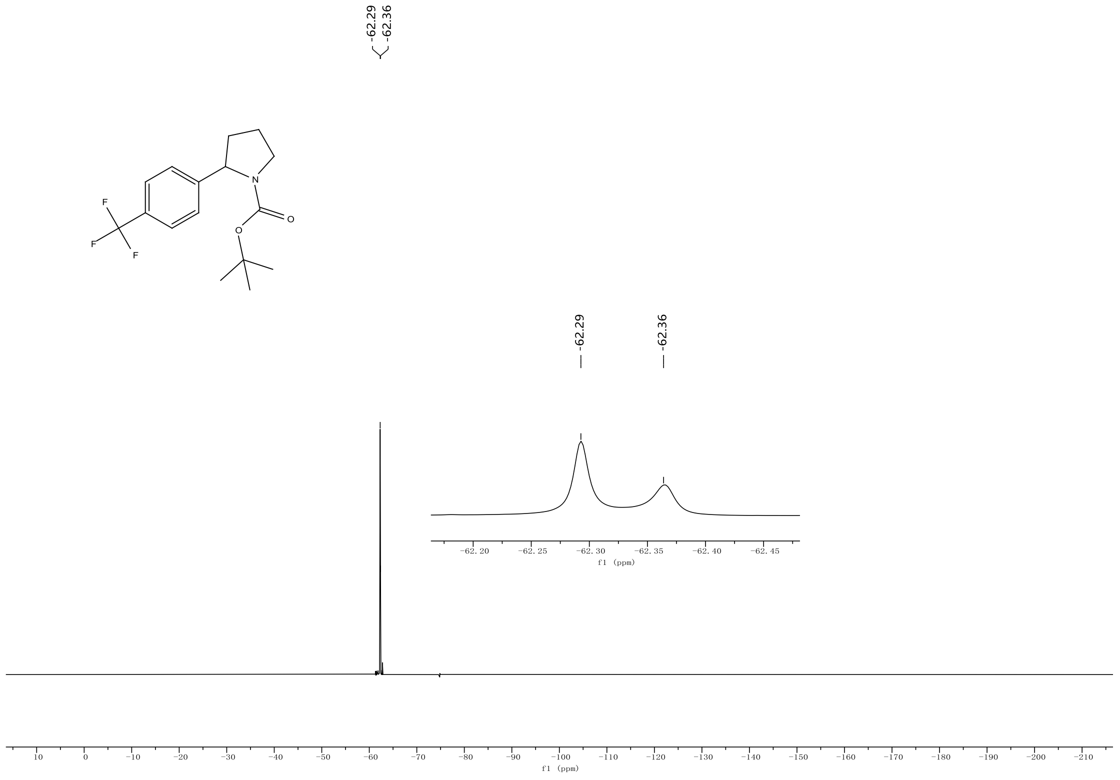

Supplementary Figure 71. ^{19}F NMR spectra of compound **12** (565 MHz, r.t., $CDCl_3$).

Compound 13

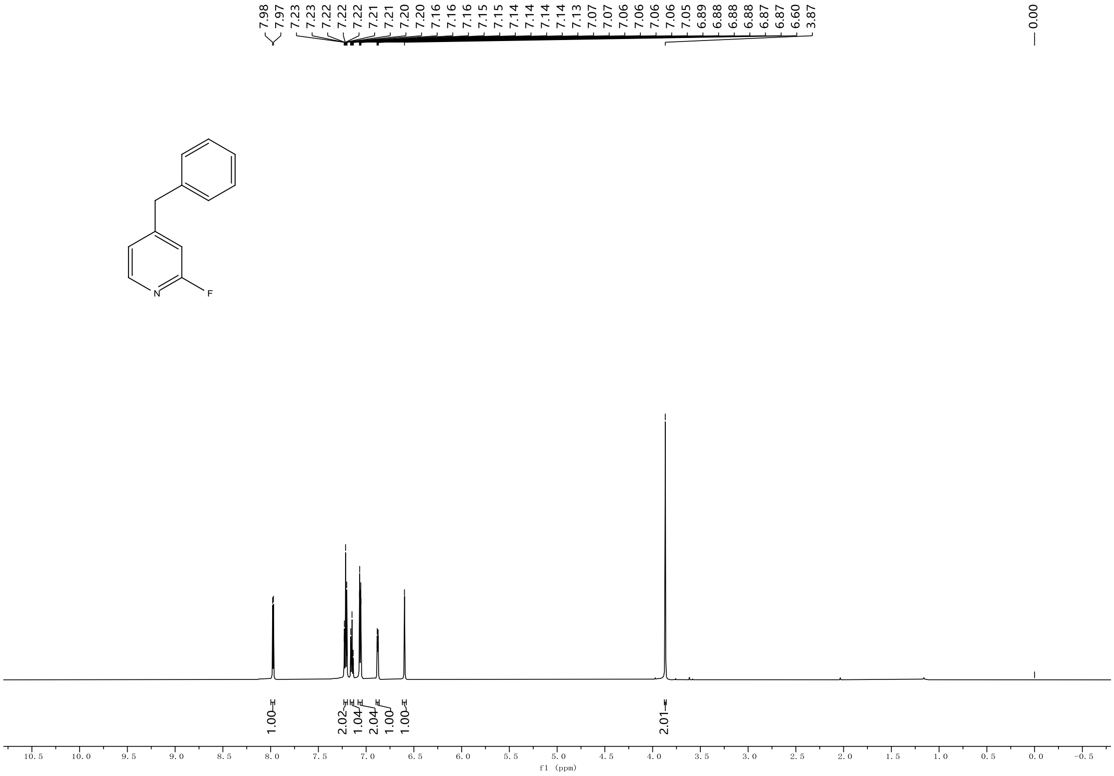

Supplementary Figure 72. ^{1}H NMR spectra of compound **13** (600 MHz, r.t., $CDCl_3$).

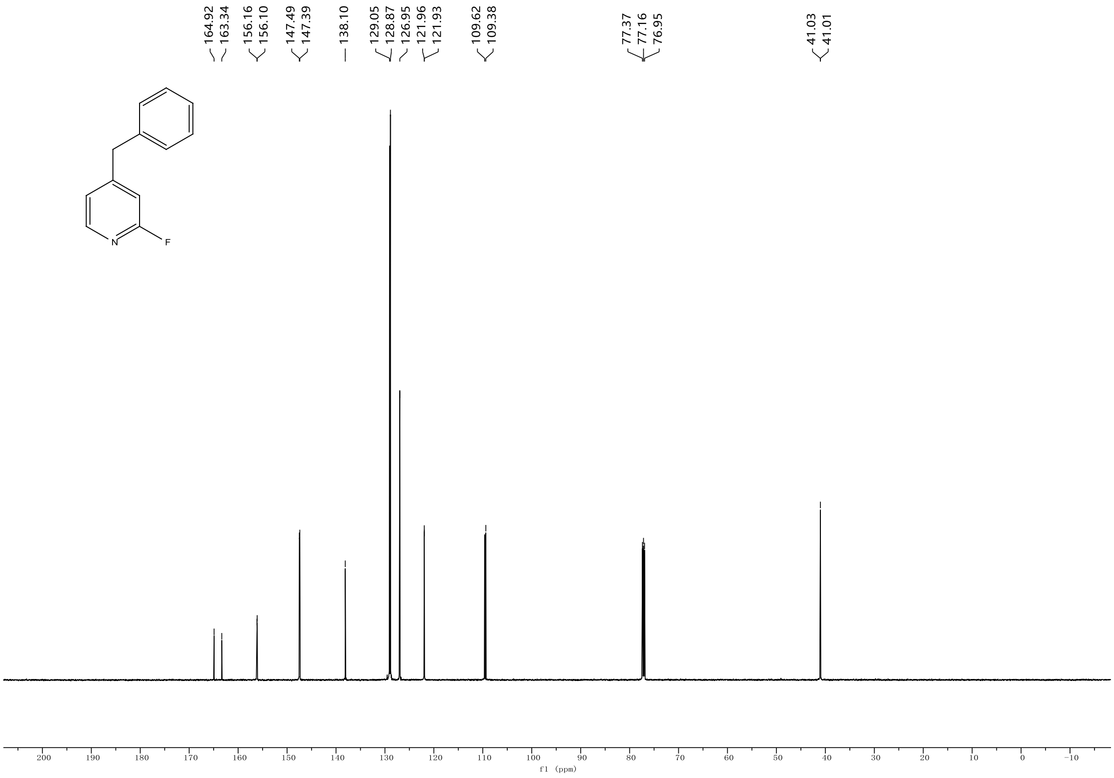

Supplementary Figure 73. ^{13}C NMR spectra of compound **13** (151 MHz, r.t., $CDCl_3$).

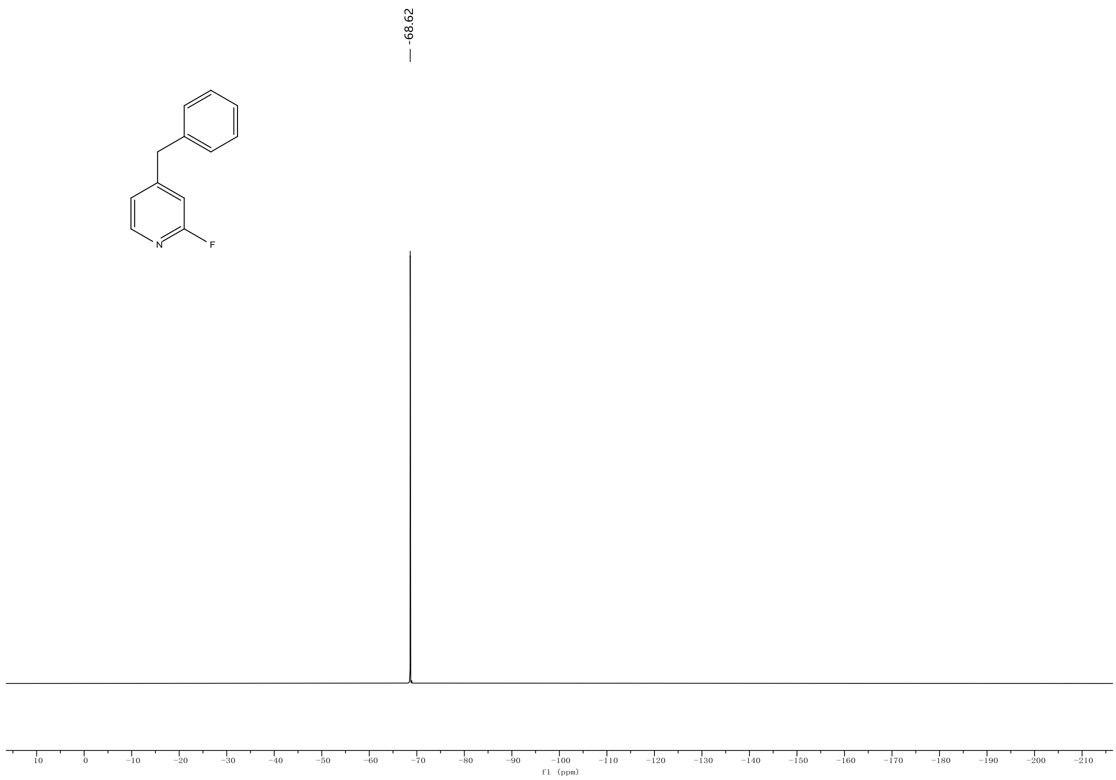

Supplementary Figure 74. ^{19}F NMR spectra of compound **13** (565 MHz, r.t., $CDCl_3$).

Compound 14

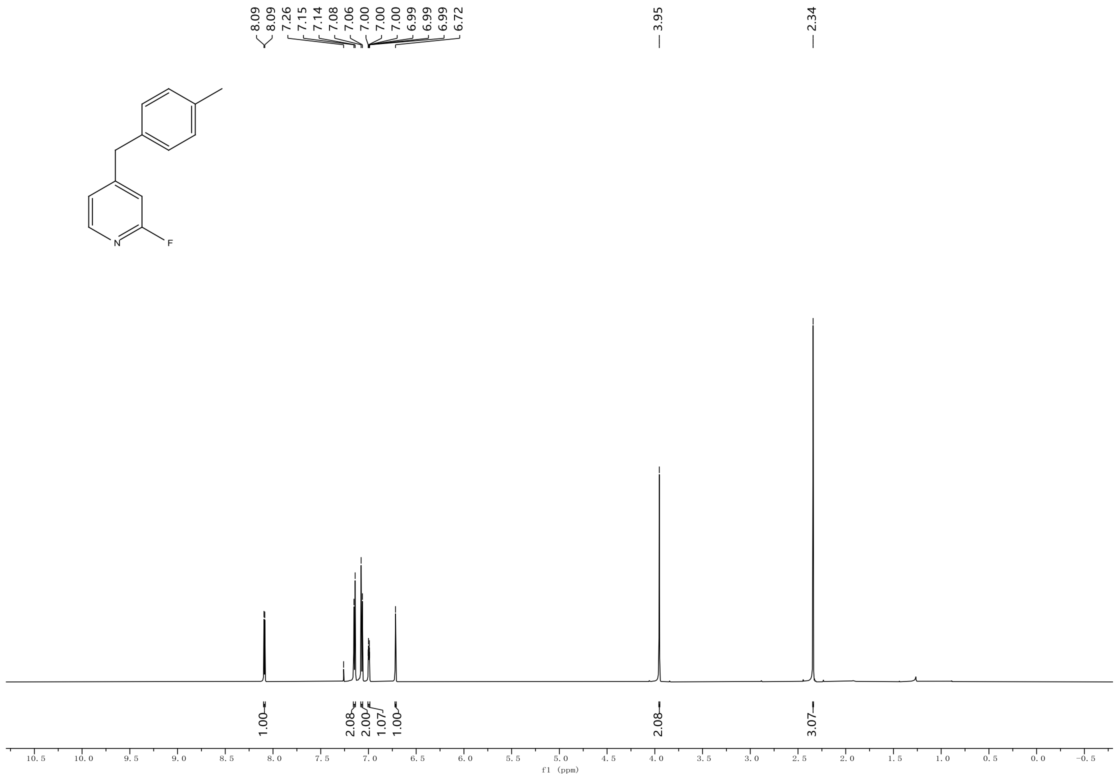

Supplementary Figure 75. ^{1}H NMR spectra of compound **14** (600 MHz, r.t., $CDCl_3$).

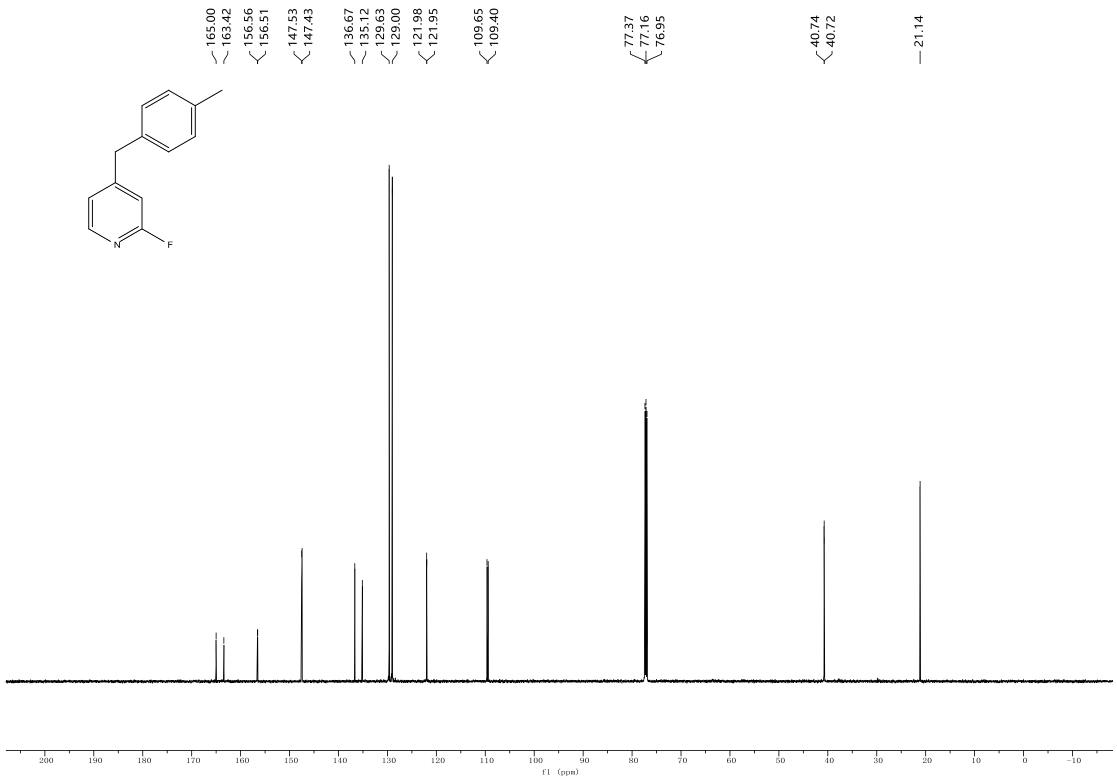

Supplementary Figure 76. ^{13}C NMR spectra of compound **14** (151 MHz, r.t., $CDCl_3$).

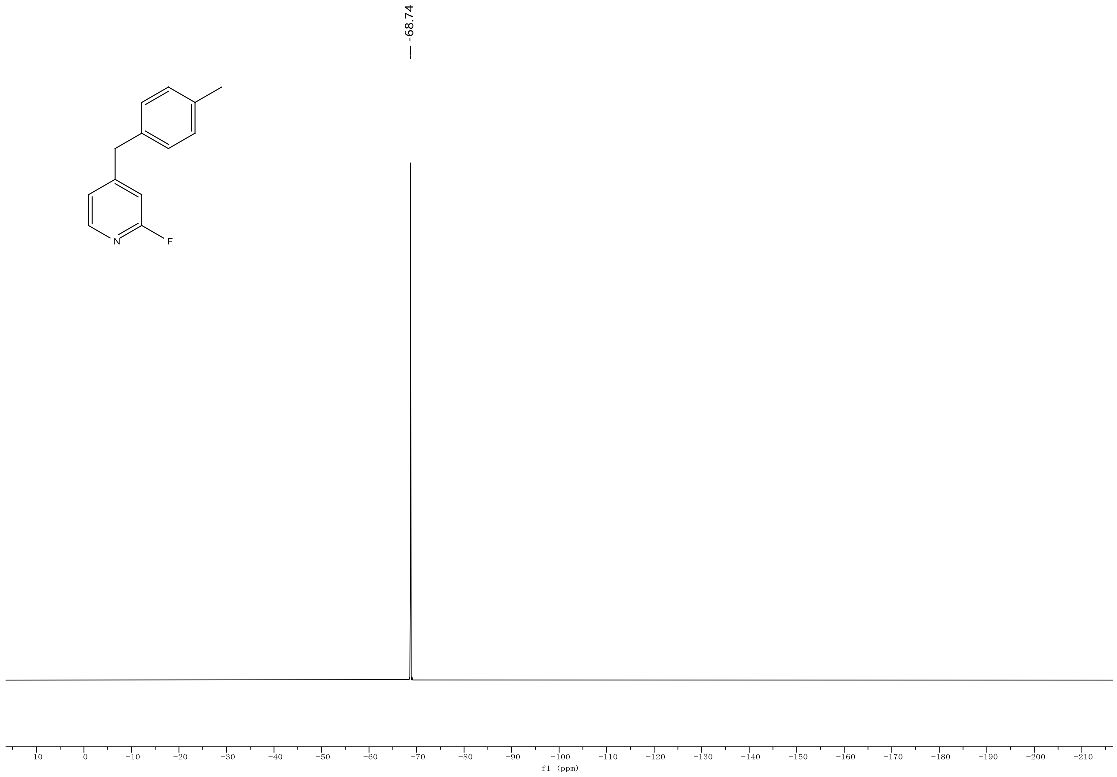

Supplementary Figure 77. ^{19}F NMR spectra of compound **14** (565 MHz, r.t., $CDCl_3$).

Compound 15

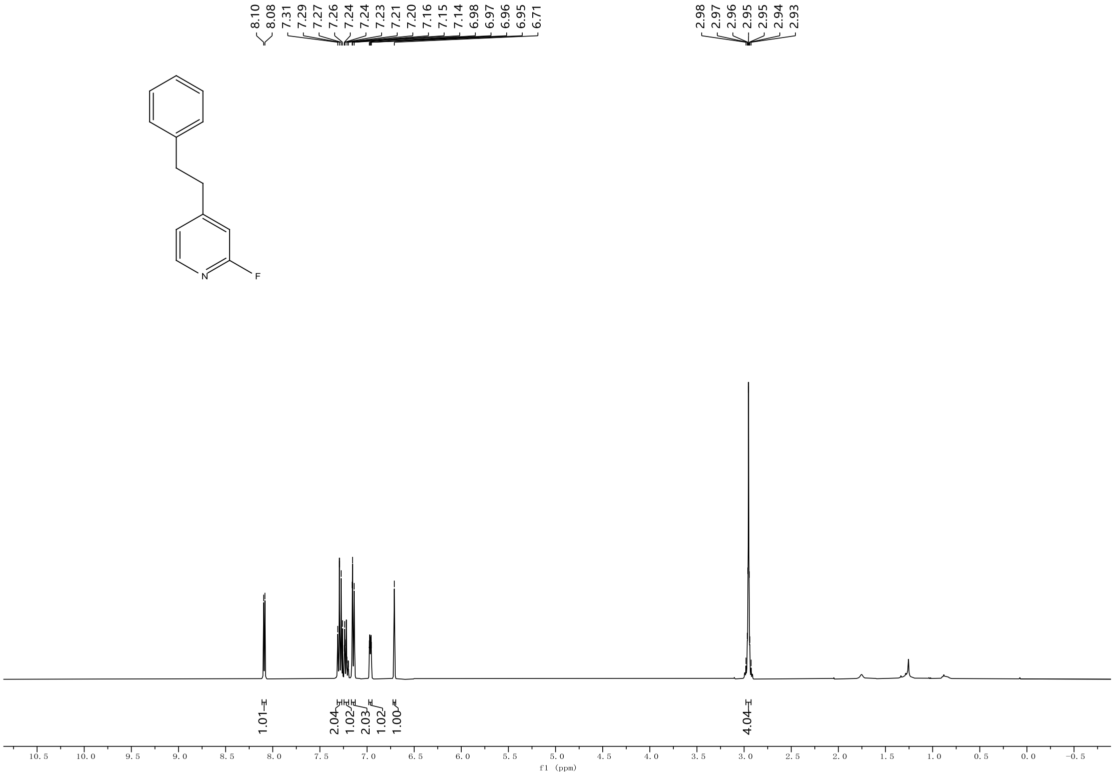

Supplementary Figure 78. ^{1}H NMR spectra of compound **15** (400 MHz, r.t., $CDCl_3$).

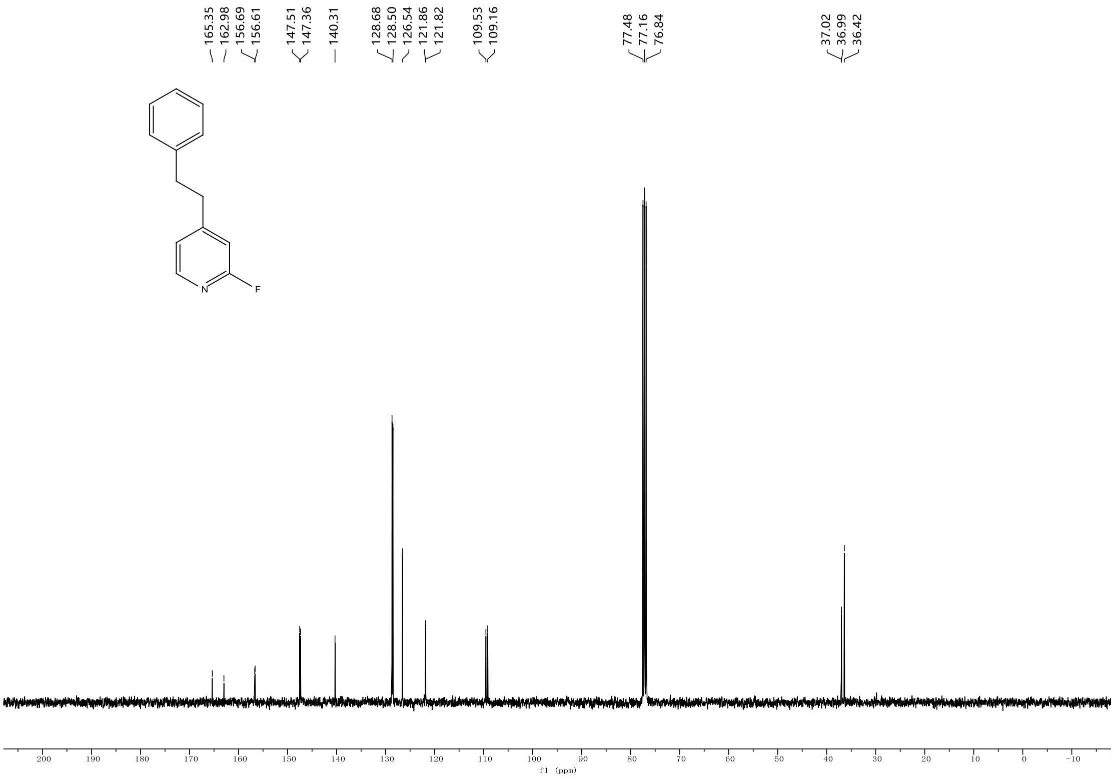

Supplementary Figure 79. ^{13}C NMR spectra of compound **15** (101 MHz, r.t., $CDCl_3$).

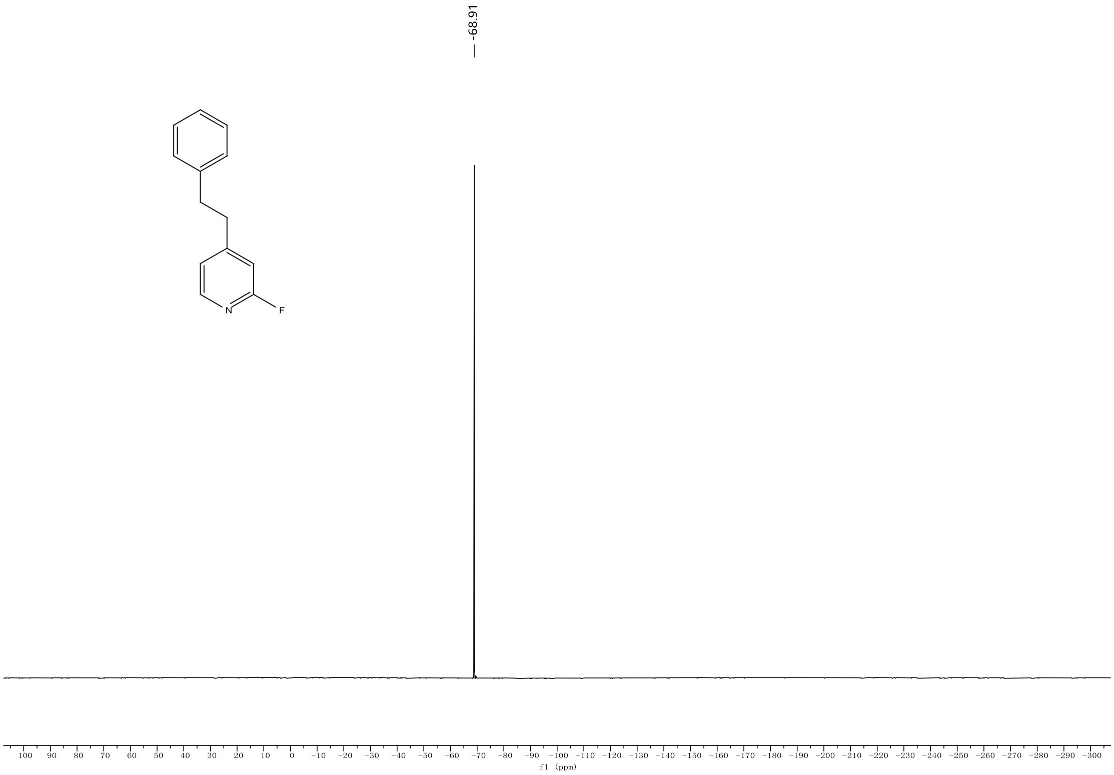

Supplementary Figure 80. ^{19}F NMR spectra of compound **15** (376 MHz, r.t., $CDCl_3$).

Compound 16

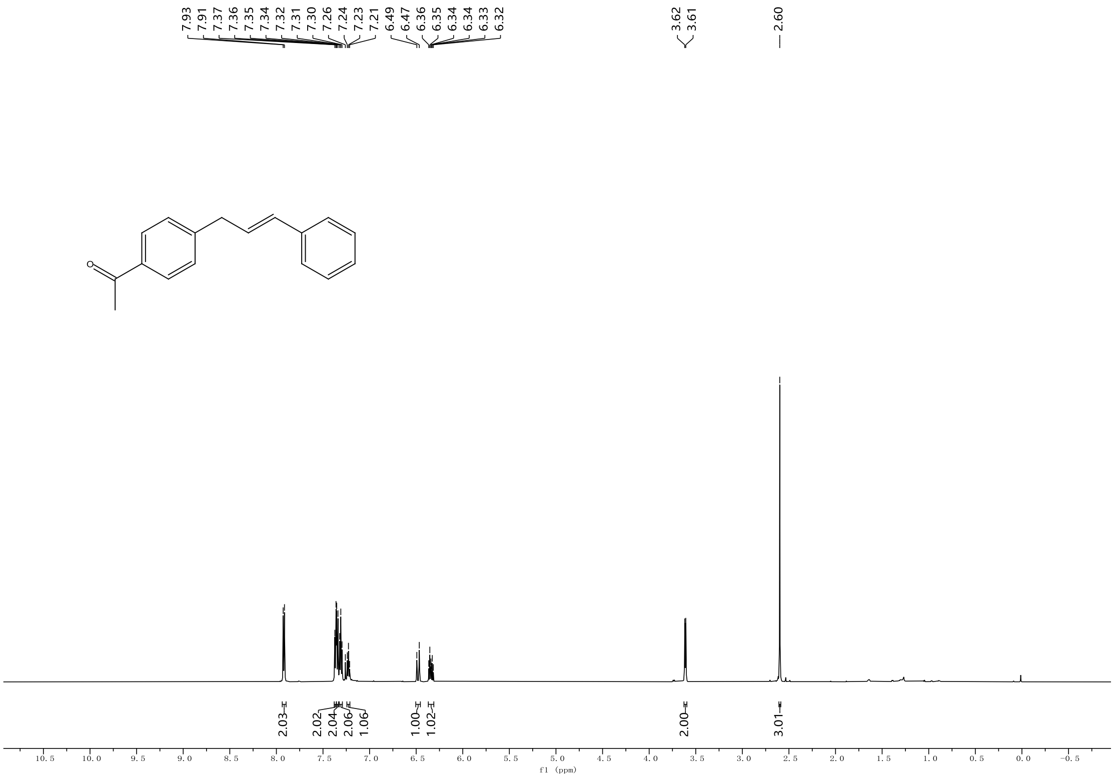

Supplementary Figure 81. ^{1}H NMR spectra of compound **16** (600 MHz, r.t., $CDCl_3$).

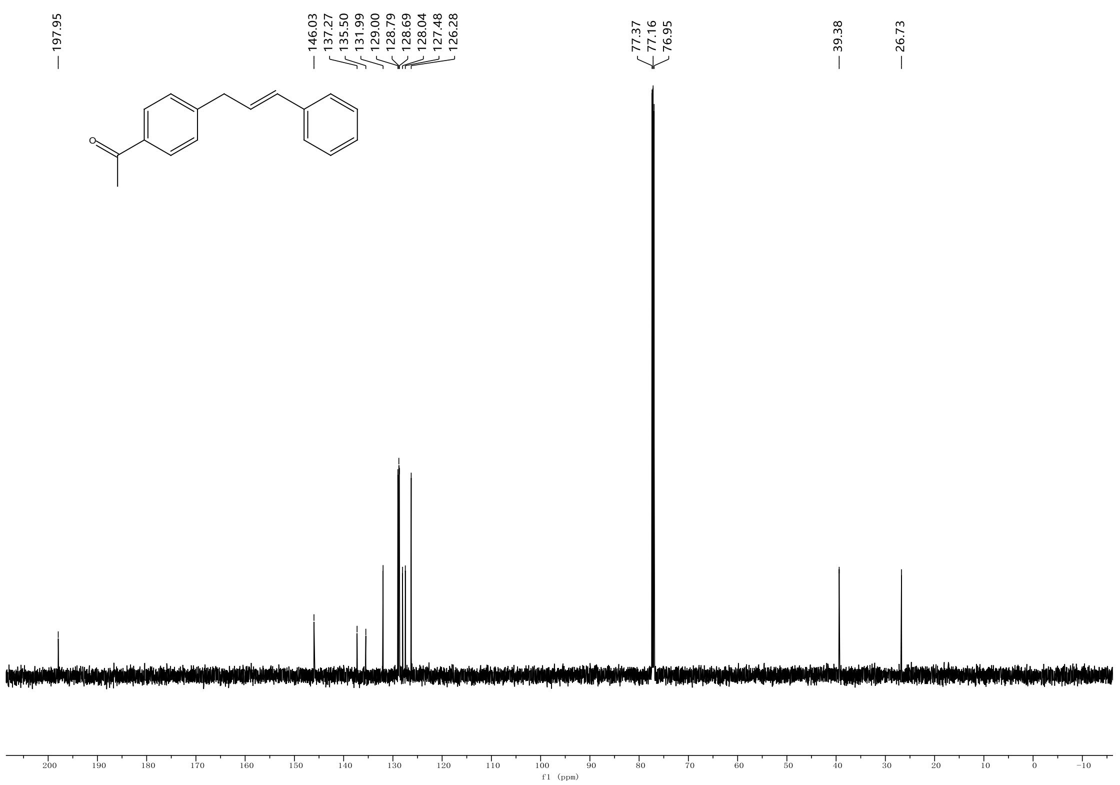

Supplementary Figure 82. ^{13}C NMR spectra of compound **16** (151 MHz, r.t., $CDCl_3$).

Compound 17

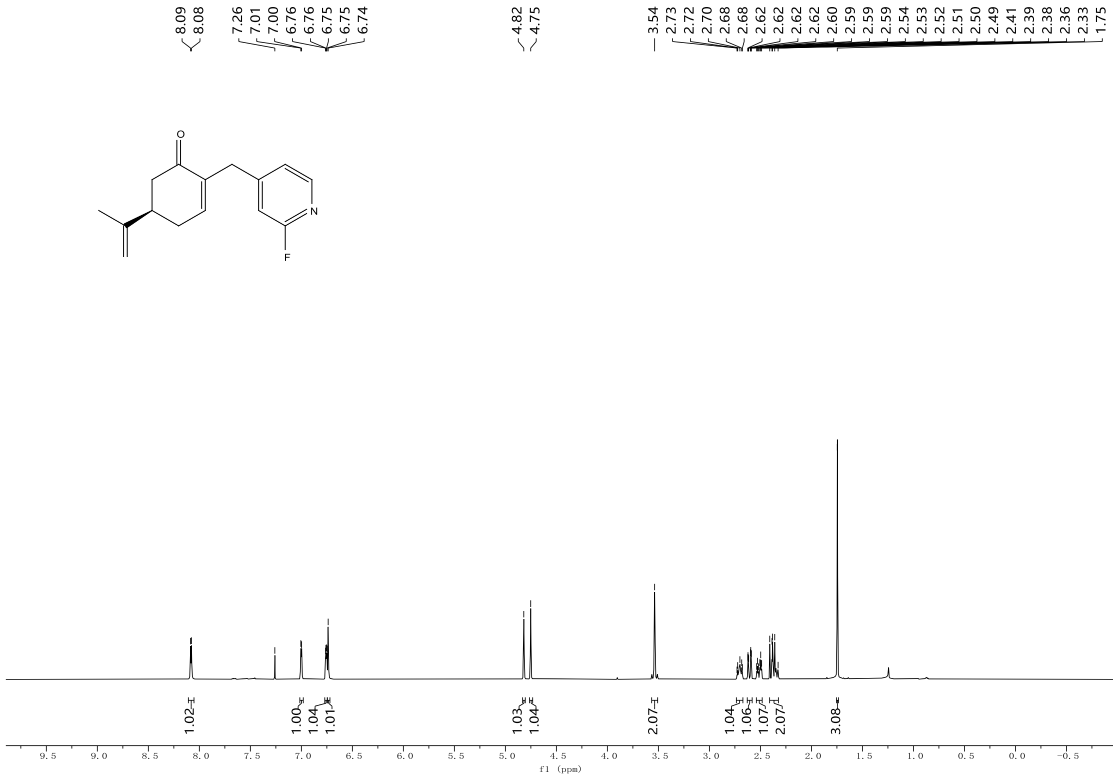

Supplementary Figure 83. ^{1}H NMR spectra of compound **17** (600 MHz, r.t., $CDCl_3$).

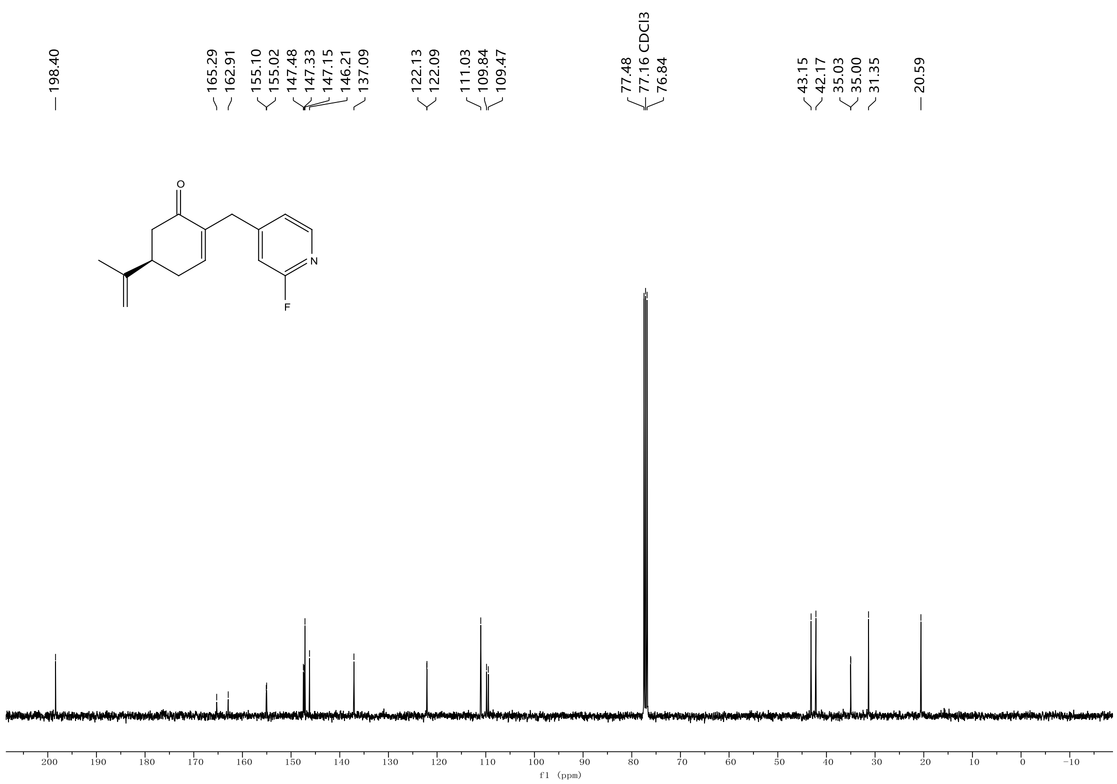

Supplementary Figure 84. ^{13}C NMR spectra of compound **17** (101 MHz, r.t., $CDCl_3$).

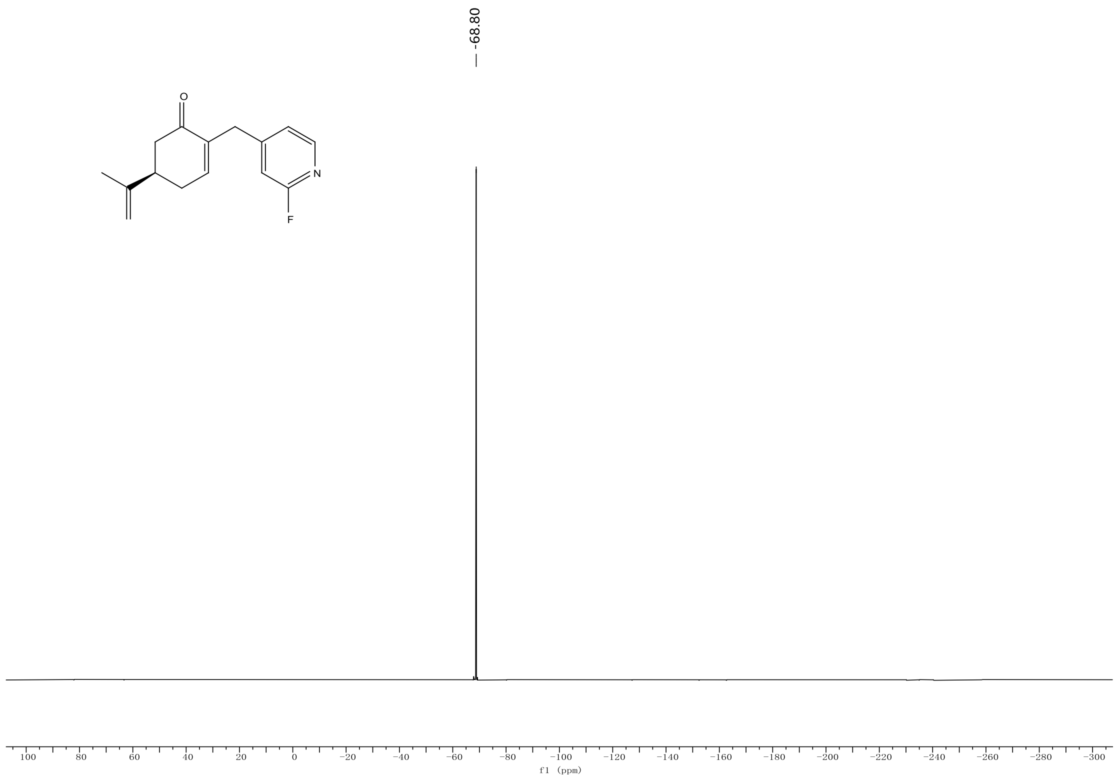

Supplementary Figure 85. ^{19}F NMR spectra of compound **17** (376 MHz, r.t., $CDCl_3$).

Compound 17-HMBC

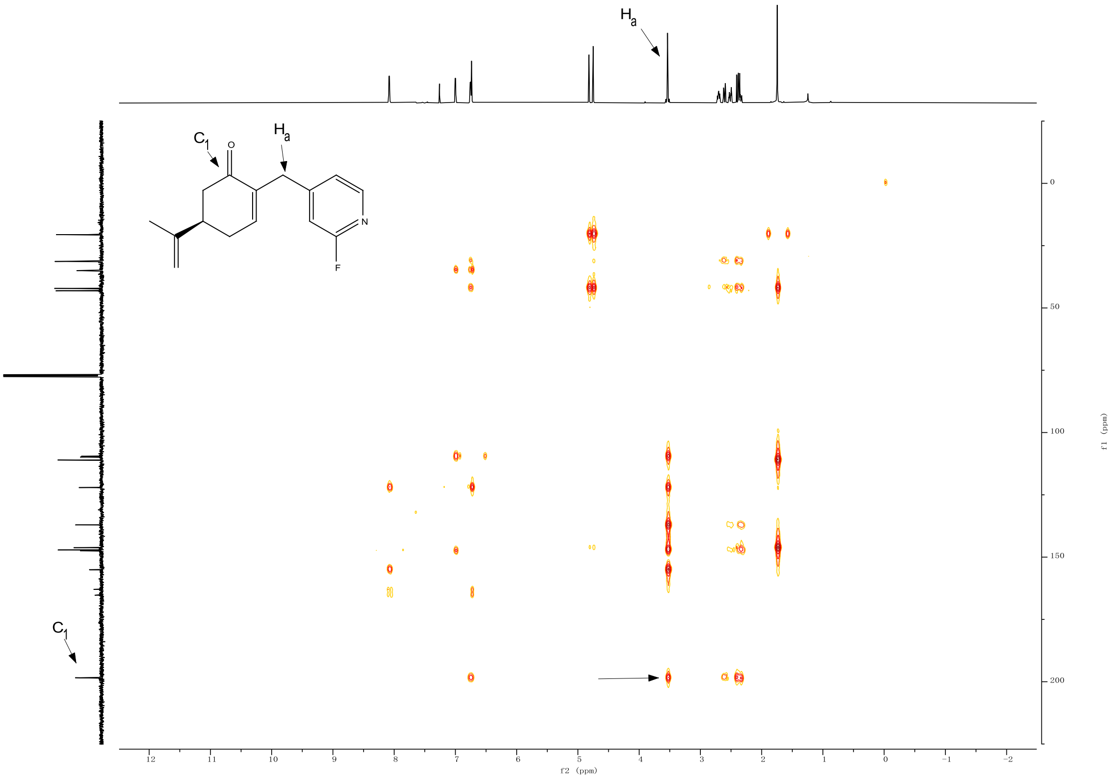

Supplementary Figure 86. HMBC spectra of compound **17** (400 MHz, r.t., $CDCl_3$).

Compound 18

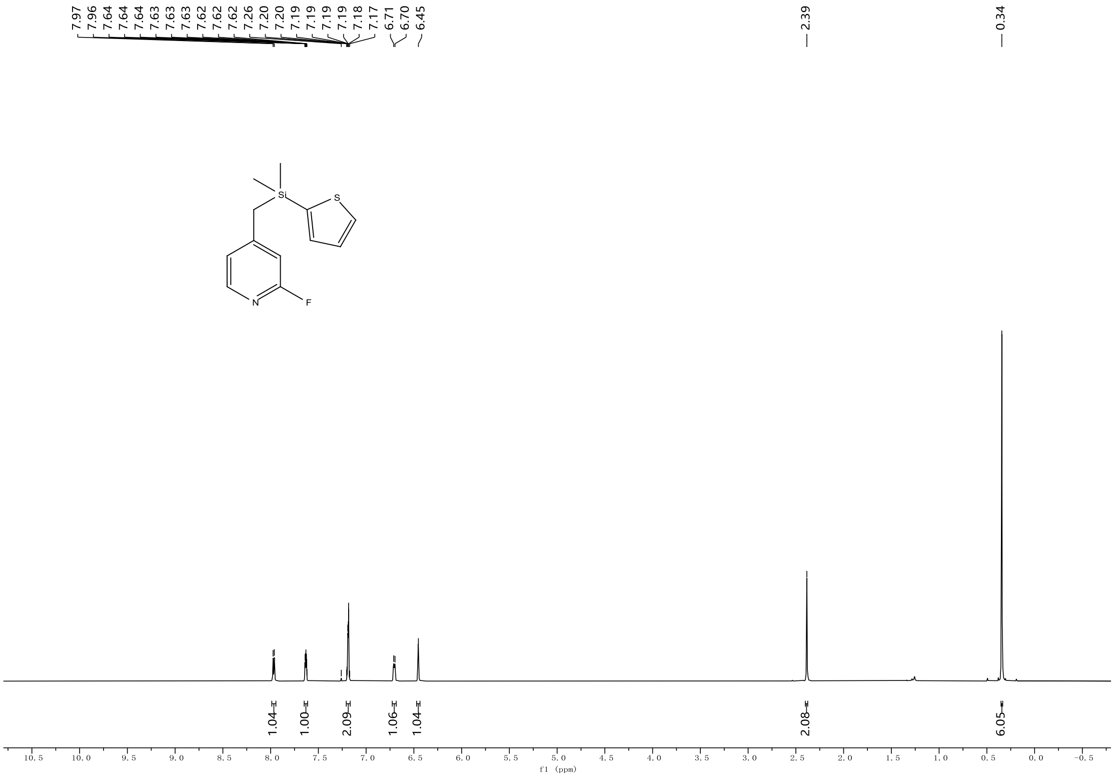

Supplementary Figure 87. ^{1}H NMR spectra of compound **18** (400 MHz, r.t., $CDCl_3$).

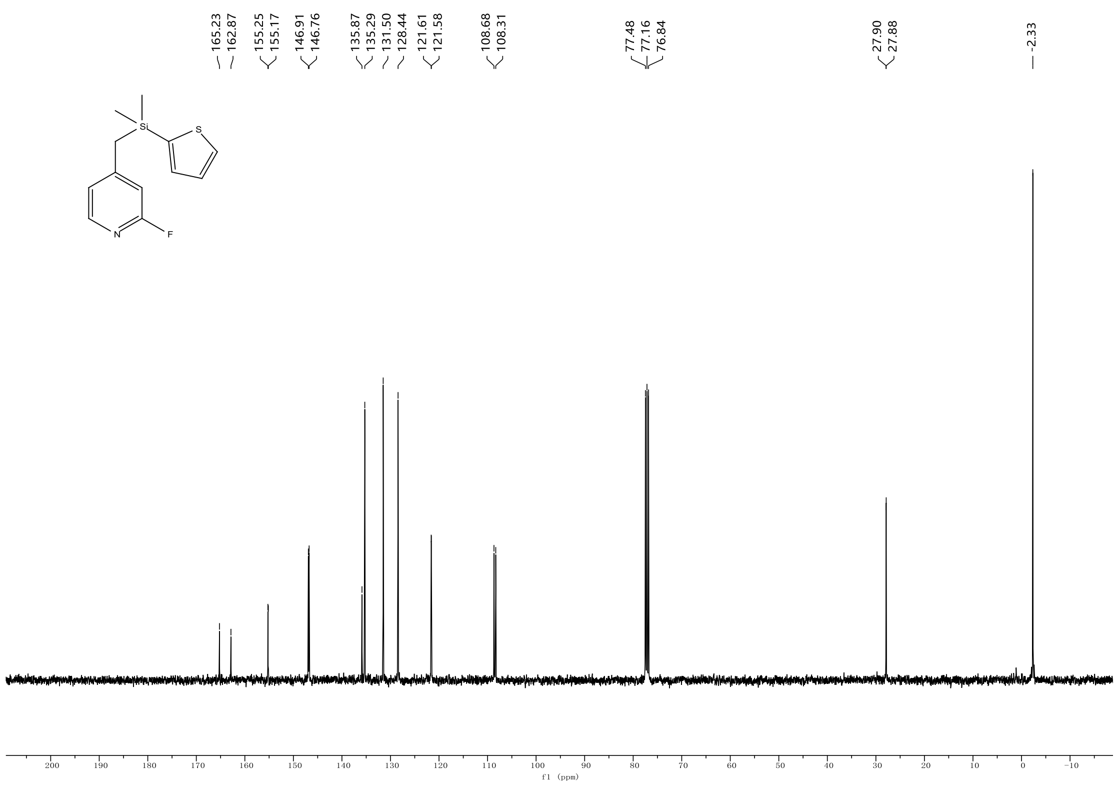

Supplementary Figure 88. ^{13}C NMR spectra of compound **18** (101 MHz, r.t., $CDCl_3$).

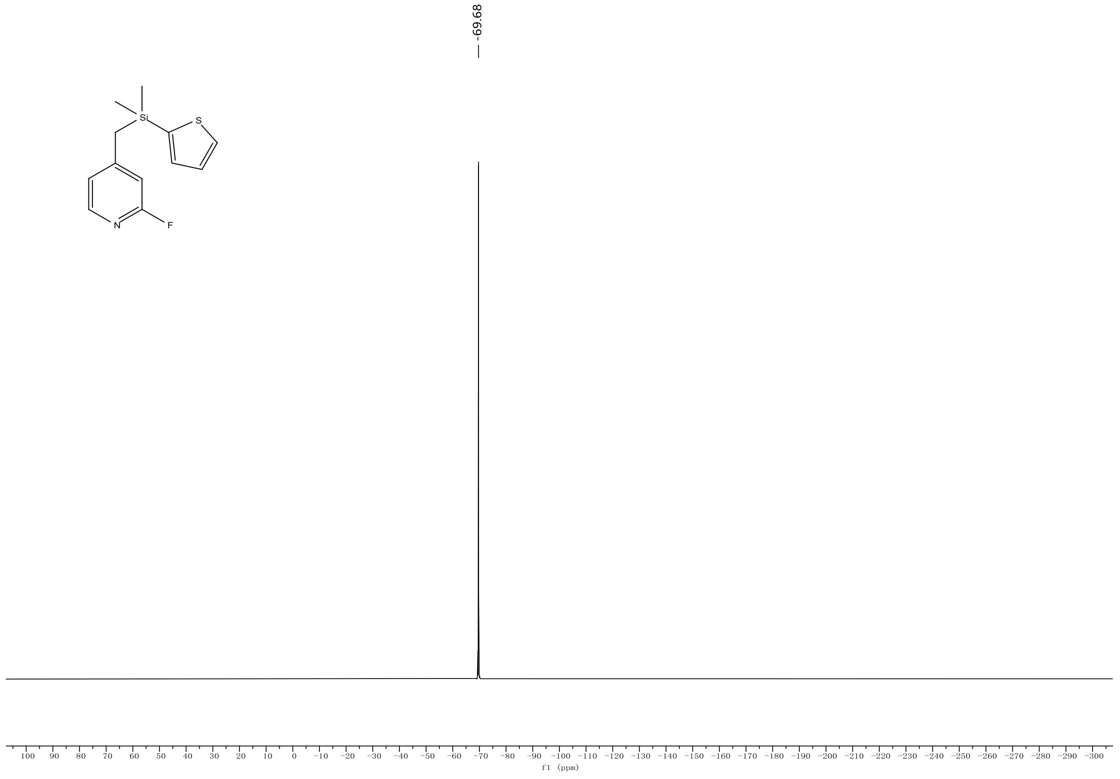

Supplementary Figure 89. ^{19}F NMR spectra of compound **18** (376 MHz, r.t., $CDCl_3$).

Compound 19

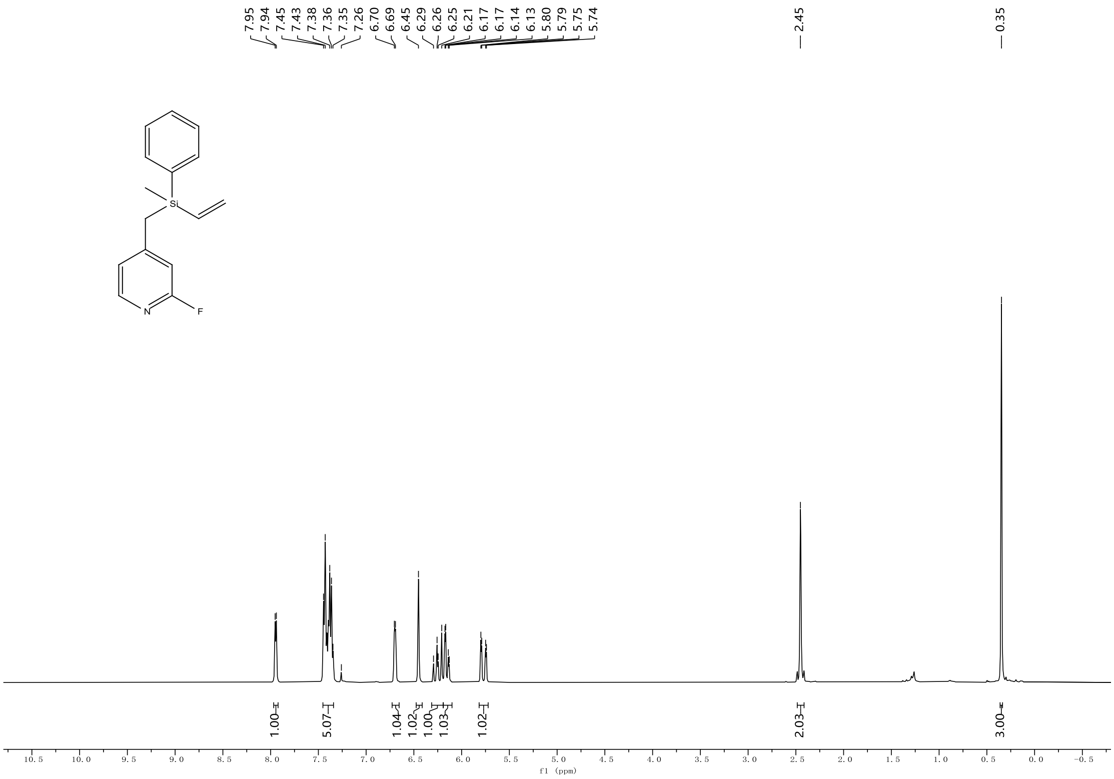

Supplementary Figure 90. ^{1}H NMR spectra of compound **19** (400 MHz, r.t., $CDCl_3$).

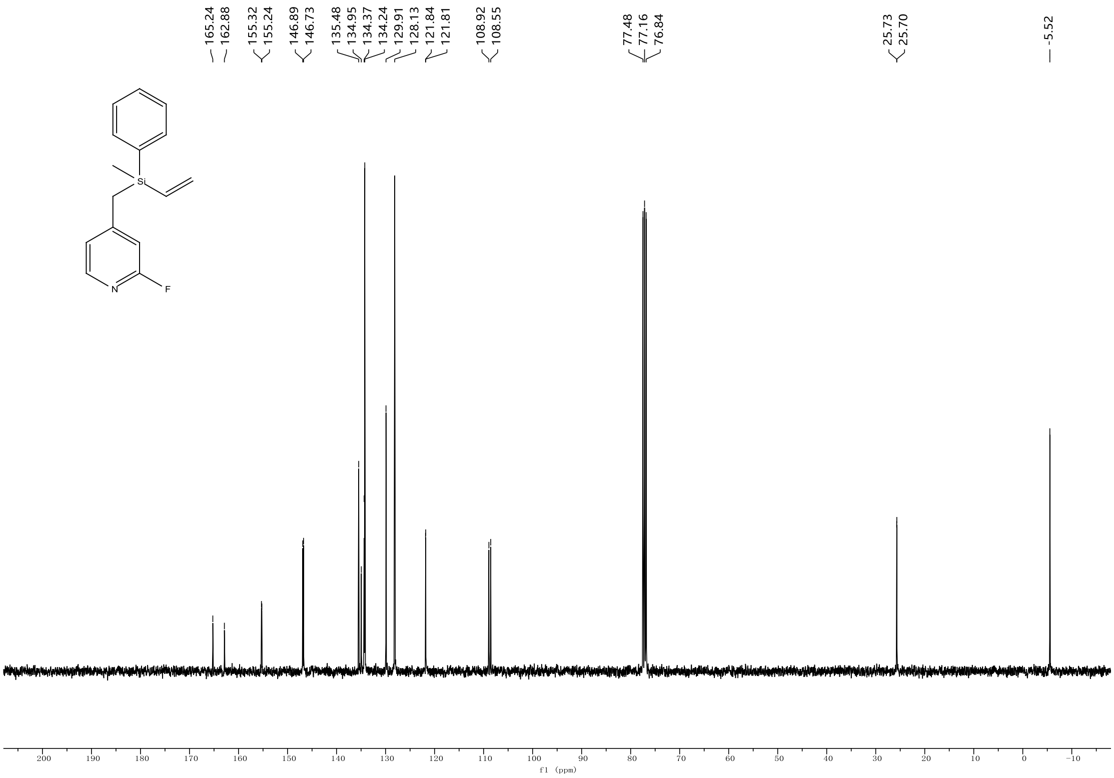

Supplementary Figure 91. ^{13}C NMR spectra of compound **19** (101 MHz, r.t., $CDCl_3$).

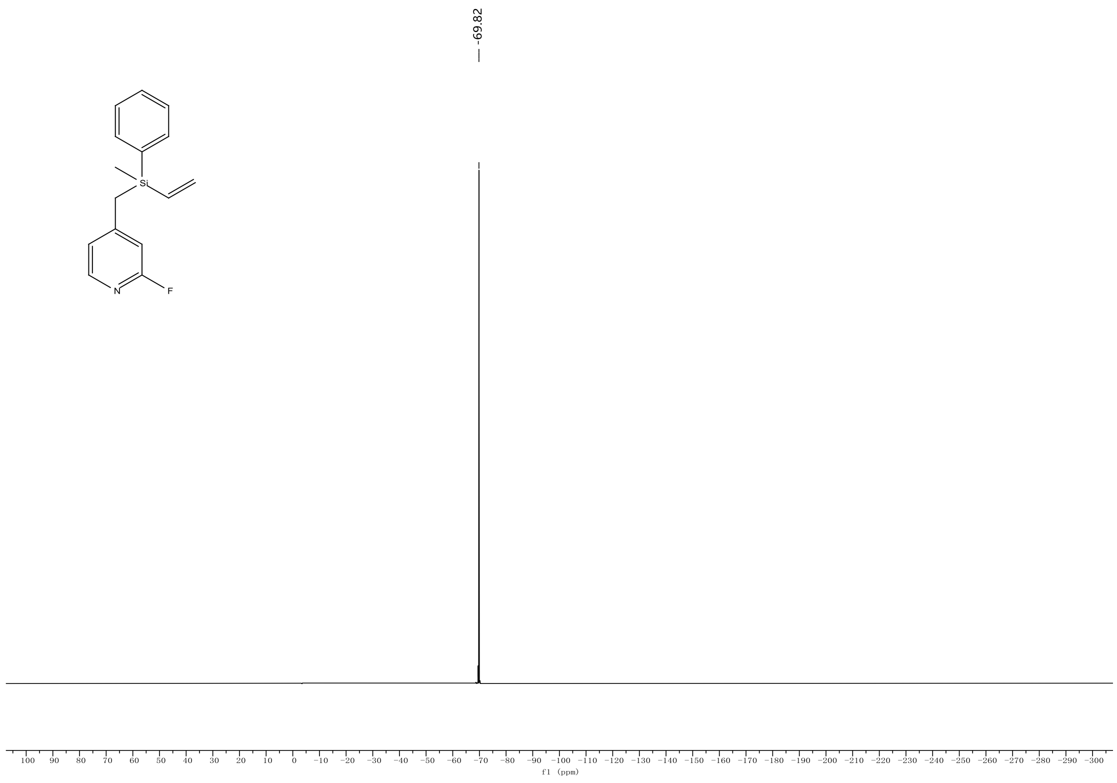

Supplementary Figure 92. ^{19}F NMR spectra of compound **19** (376 MHz, r.t., $CDCl_3$).

Compound 20

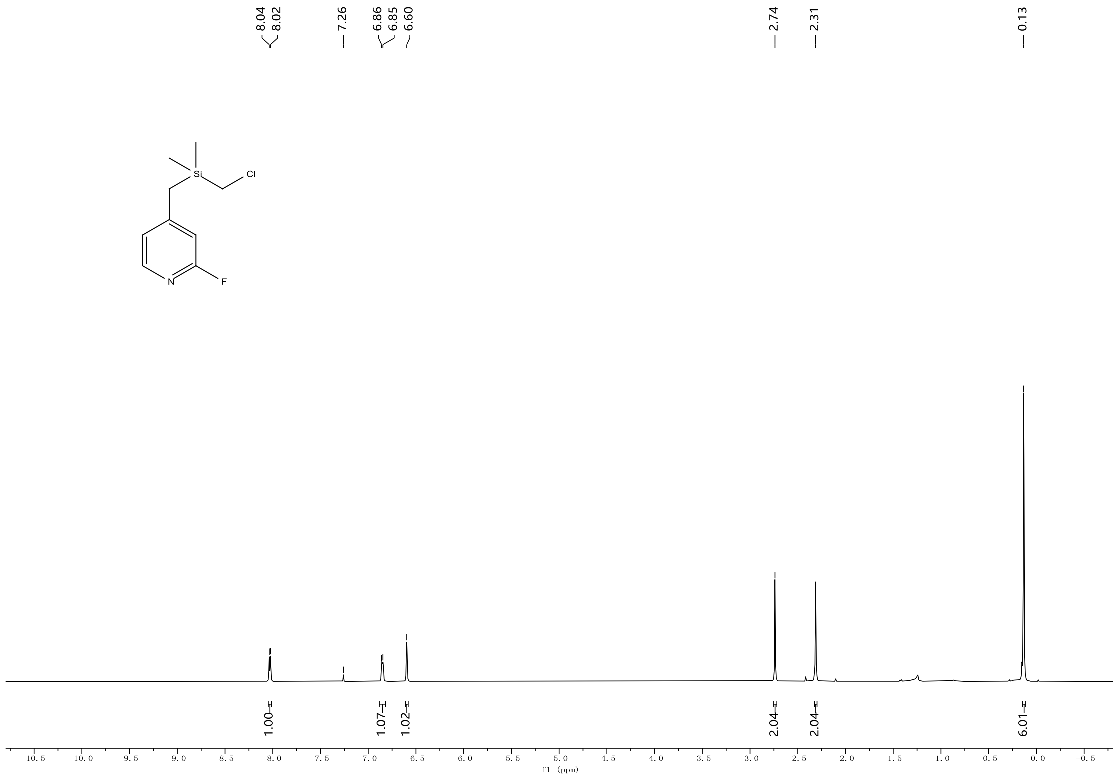

Supplementary Figure 93. ^{1}H NMR spectra of compound **20** (400 MHz, r.t., $CDCl_3$).

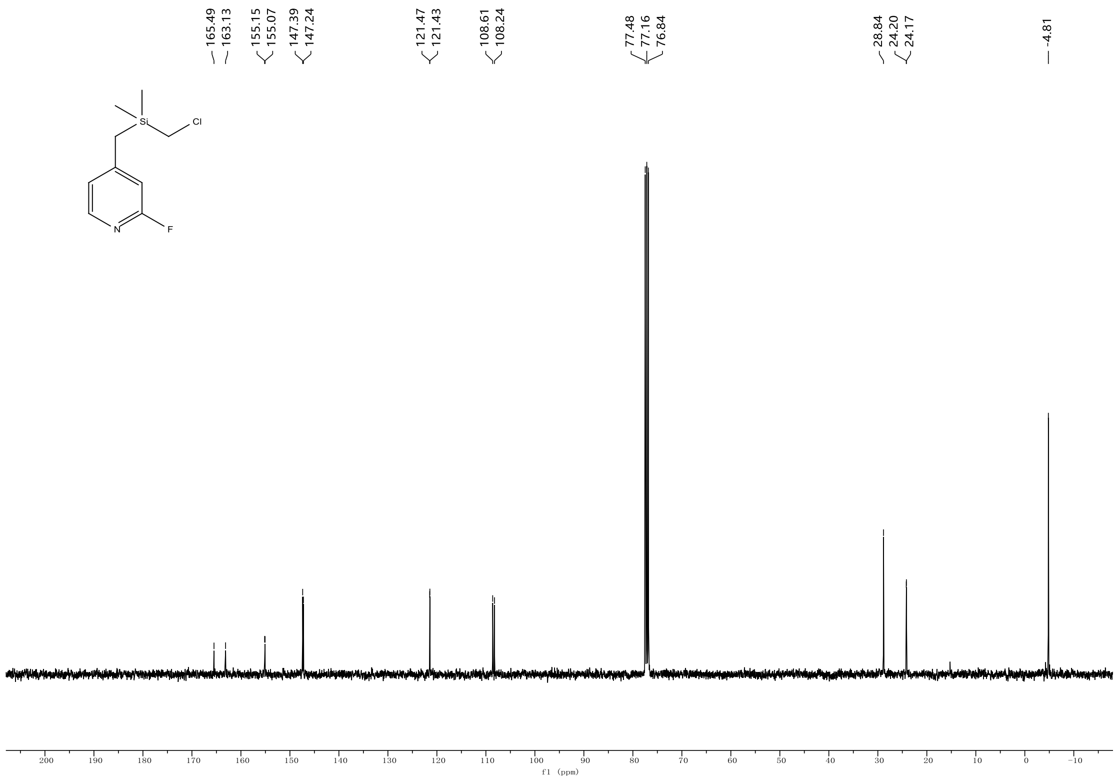

Supplementary Figure 94. ^{13}C NMR spectra of compound **20** (101 MHz, r.t., $CDCl_3$).

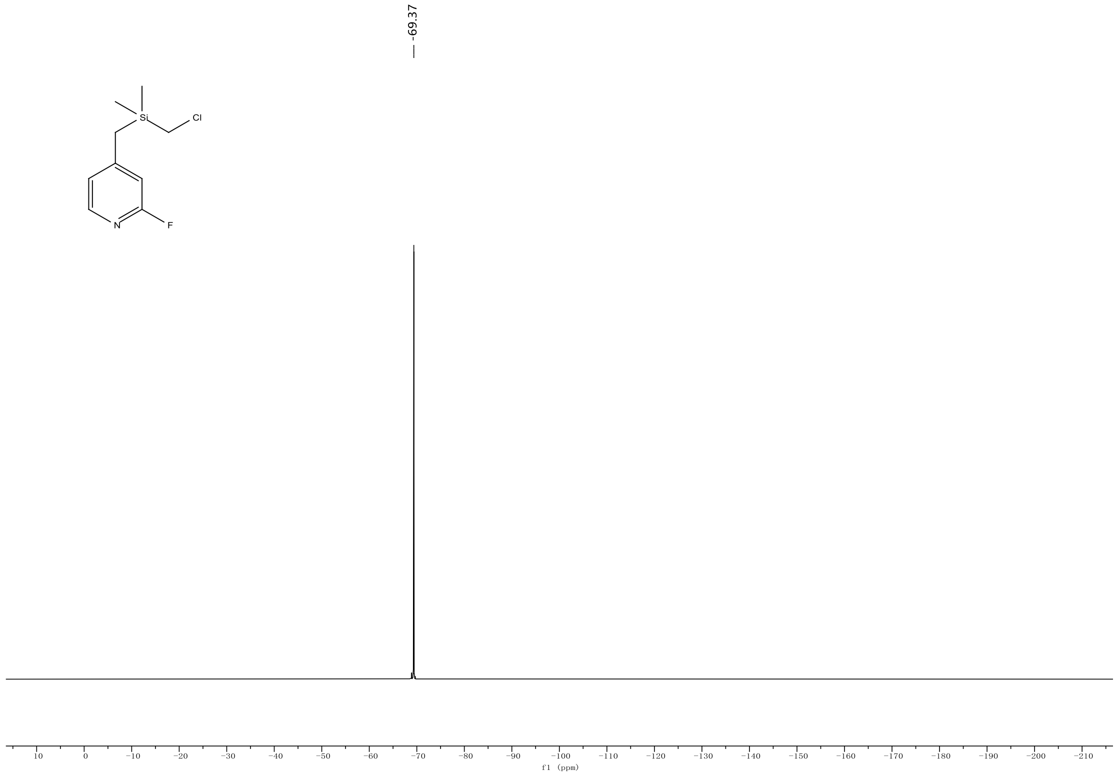

Supplementary Figure 95. ^{19}F NMR spectra of compound **20** (376 MHz, r.t., $CDCl_3$).

Compound 21

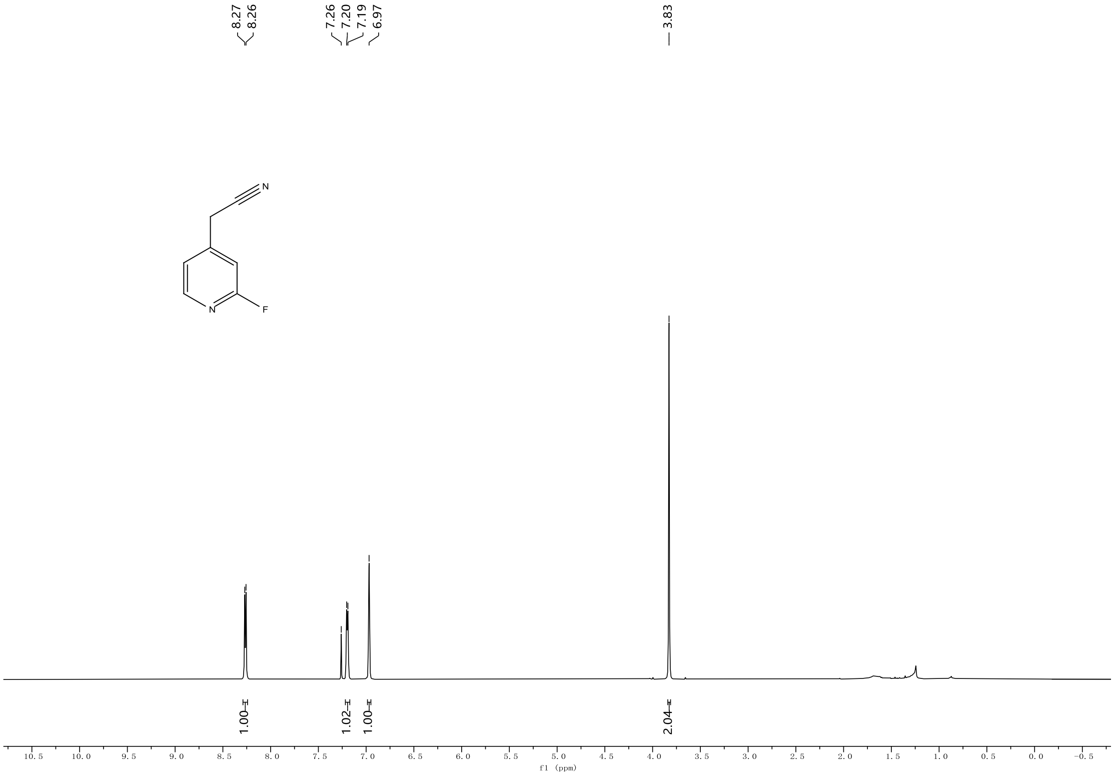

Supplementary Figure 96. ^{1}H NMR spectra of compound **21** (400 MHz, r.t., $CDCl_3$).

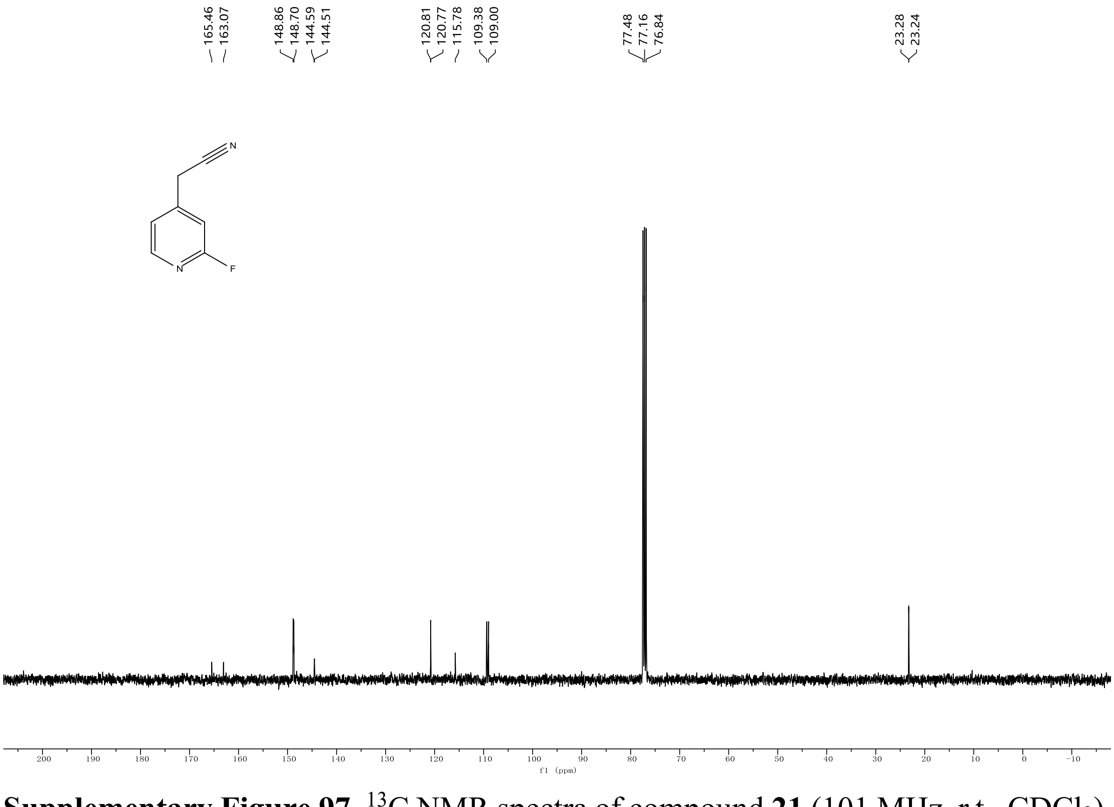

Supplementary Figure 97. ^{13}C NMR spectra of compound **21** (101 MHz, r.t., $CDCl_3$).

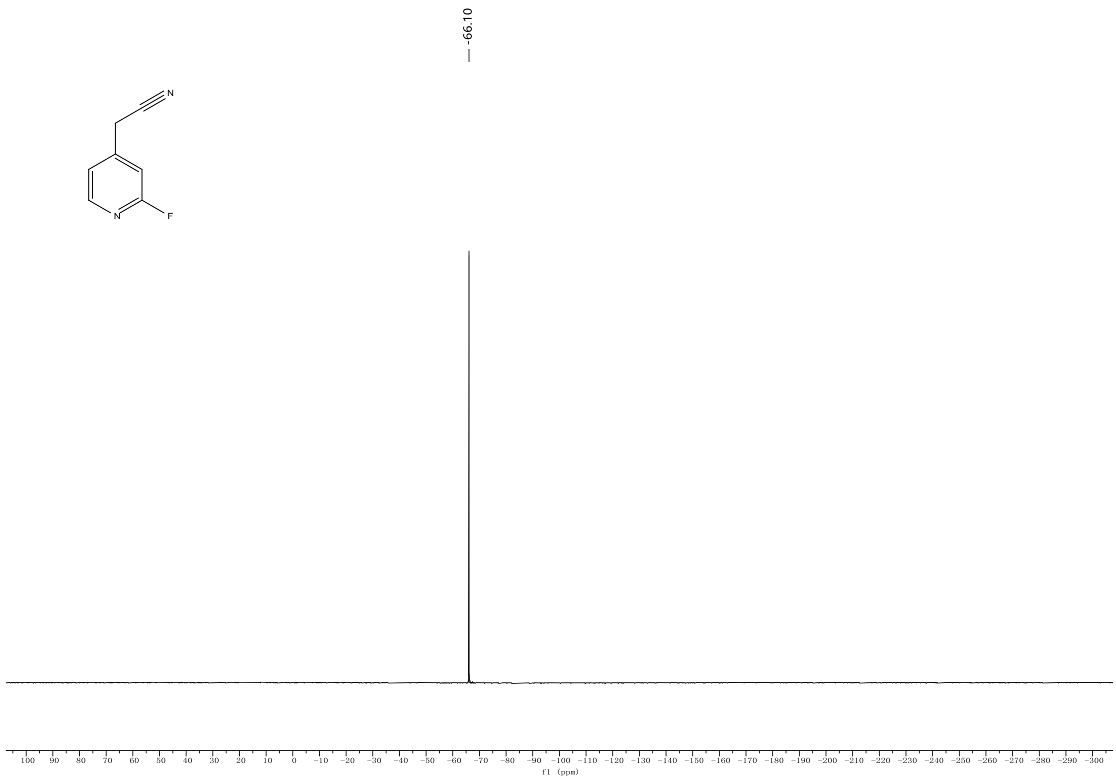

Supplementary Figure 98. ^{19}F NMR spectra of compound **21** (376 MHz, r.t., $CDCl_3$).

Compound 22

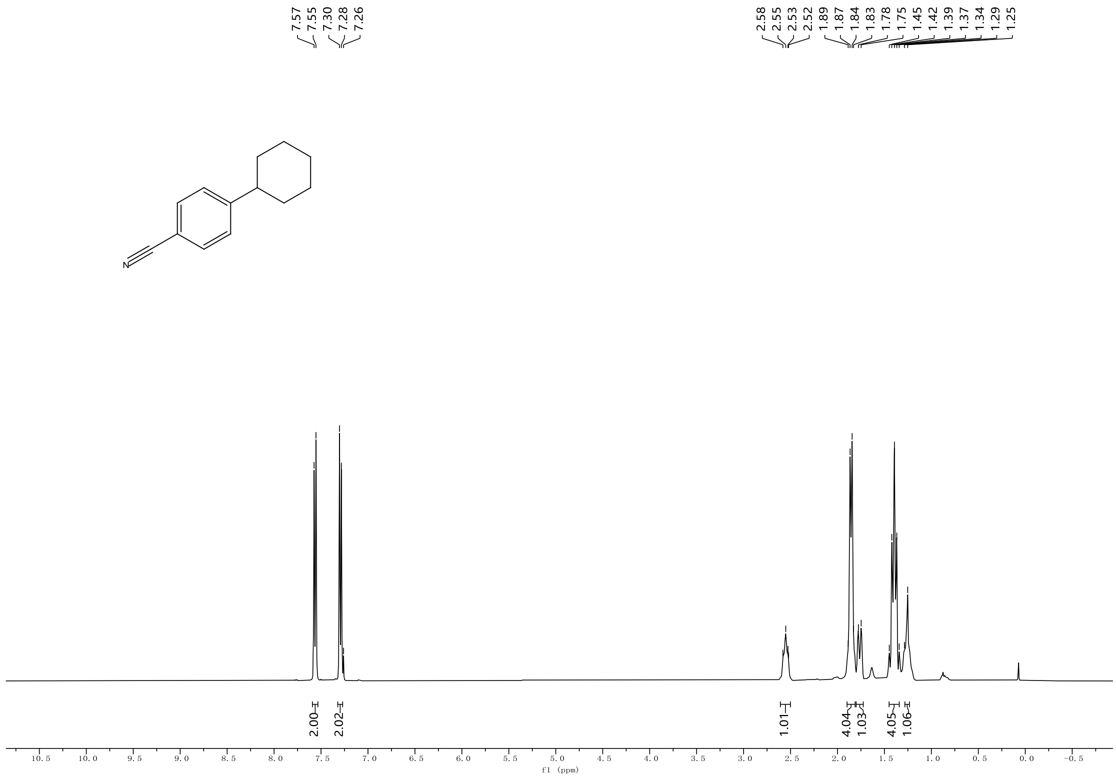

Supplementary Figure 99. ^{1}H NMR spectra of compound **22** (400 MHz, r.t., $CDCl_3$).

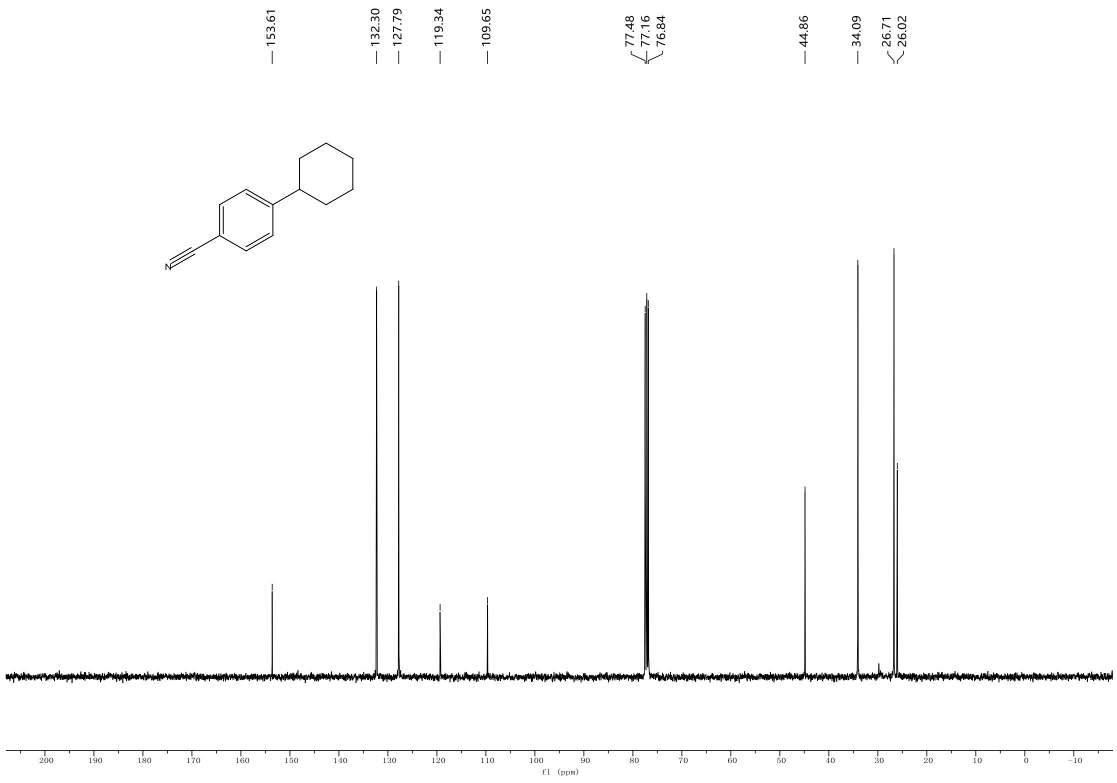

Supplementary Figure 100. ^{13}C NMR spectra of compound **22** (101 MHz, r.t., $CDCl_3$).

Compound 23

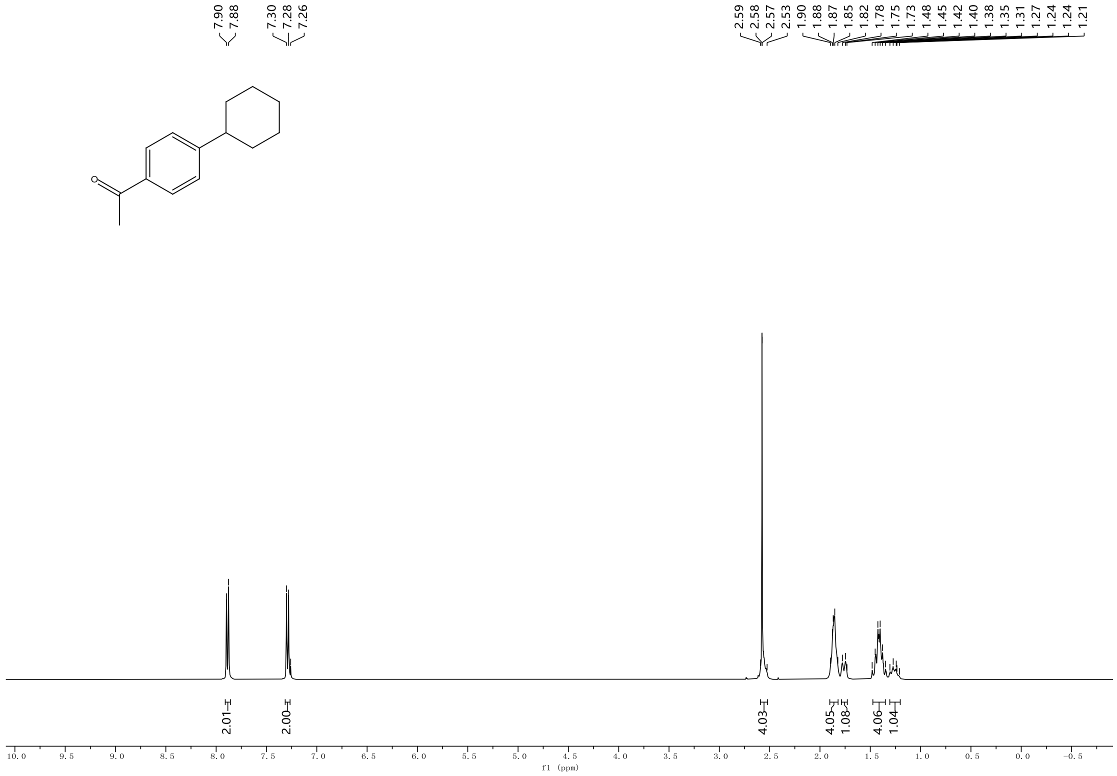

Supplementary Figure 101. ^{1}H NMR spectra of compound **23** (400 MHz, r.t., $CDCl_3$).

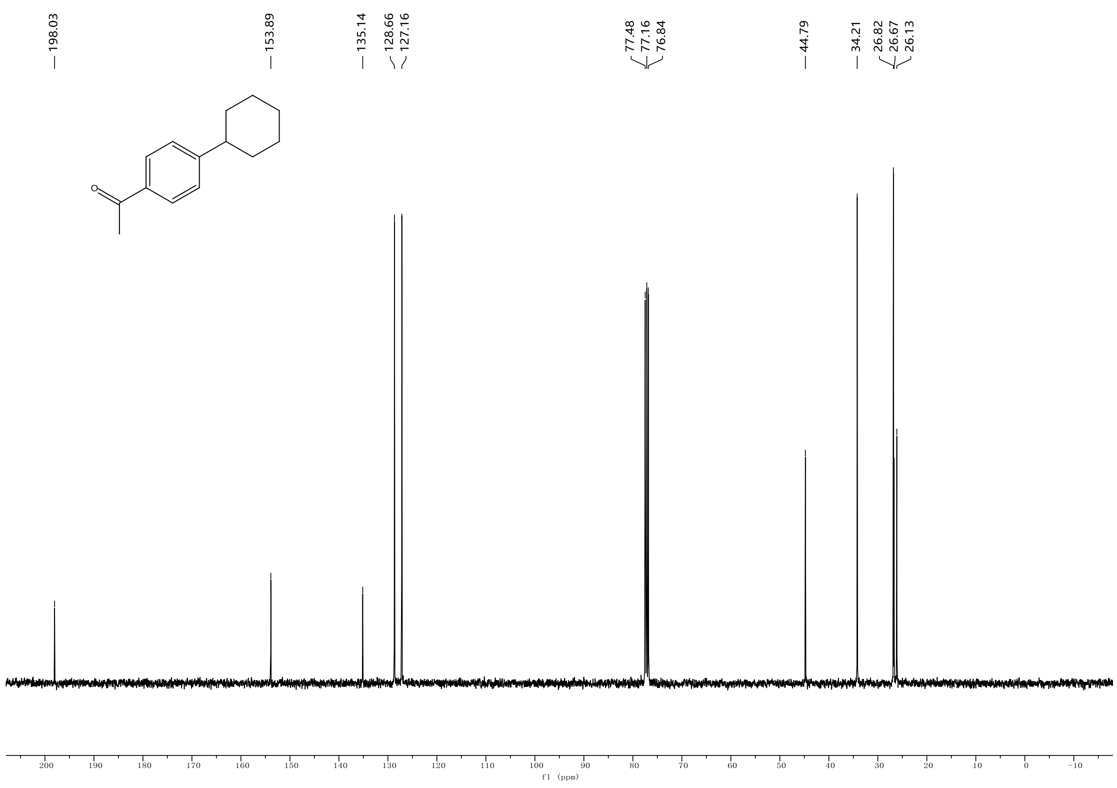

Supplementary Figure 102. ^{13}C NMR spectra of compound **22** (151 MHz, r.t., $CDCl_3$).

Compound 24

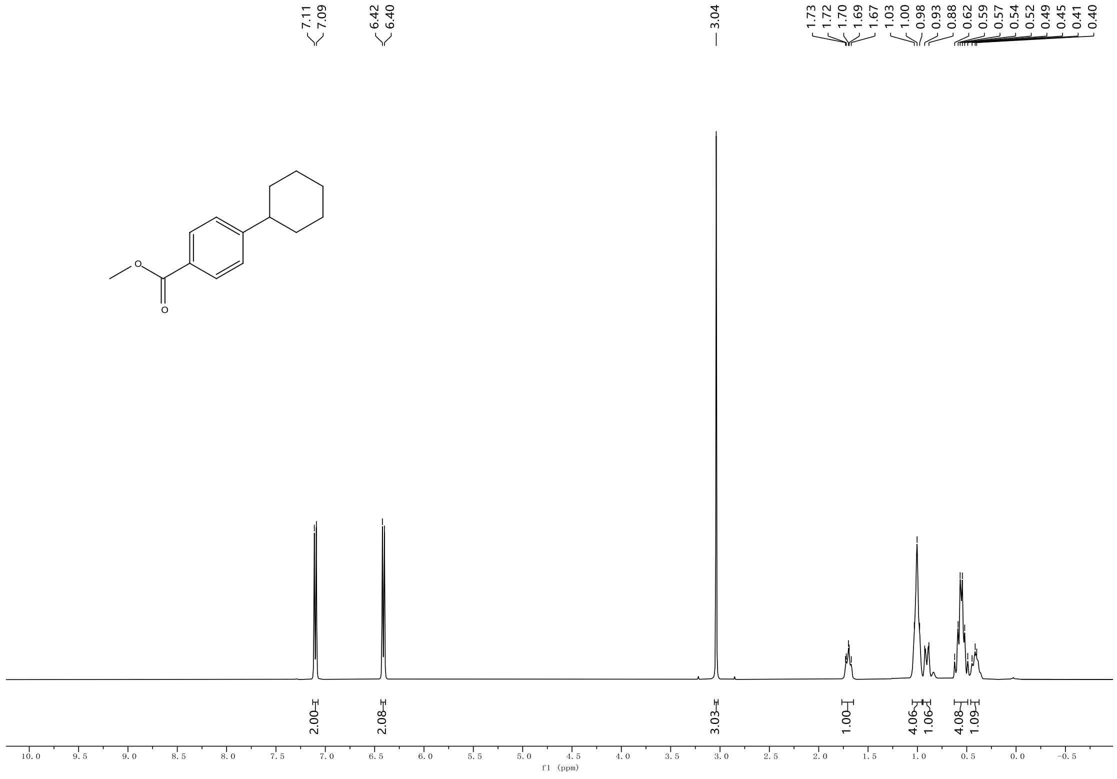

Supplementary Figure 103. ^{1}H NMR spectra of compound **24** (400 MHz, r.t., $CDCl_3$).

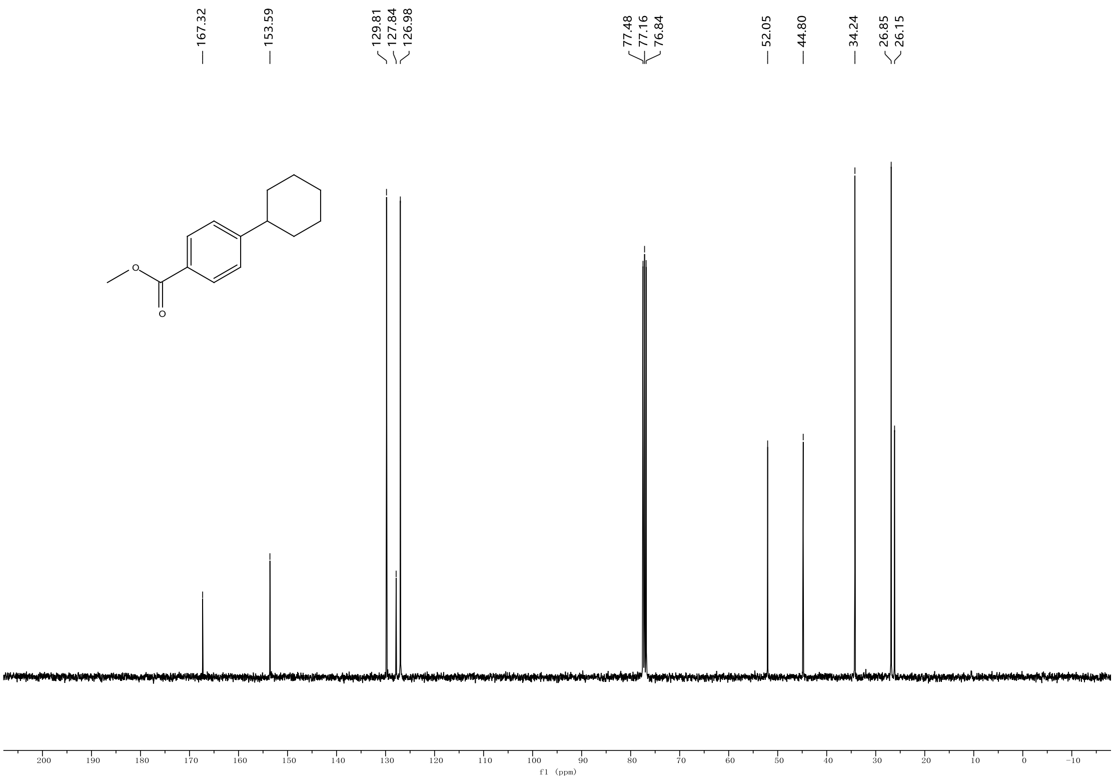

Supplementary Figure 104. ^{13}H NMR spectra of compound **24** (151 MHz, r.t., $CDCl_3$).

Compound 25

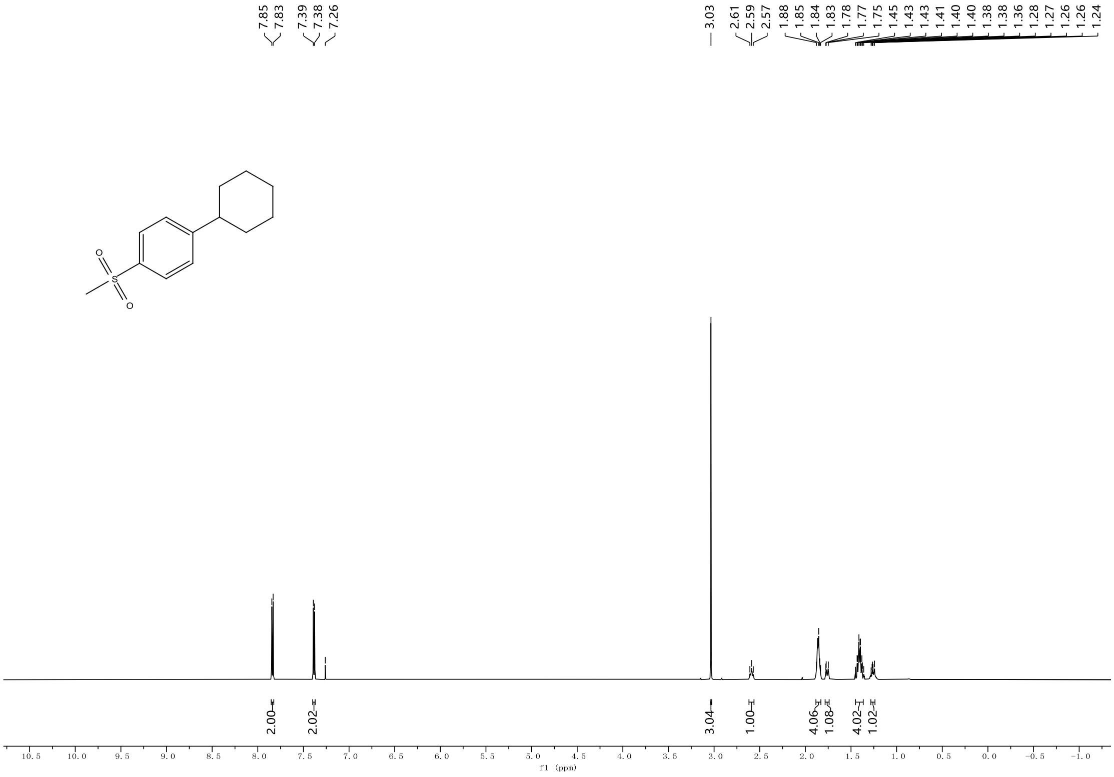

Supplementary Figure 105. ^{1}H NMR spectra of compound **25** (600 MHz, r.t., $CDCl_3$).

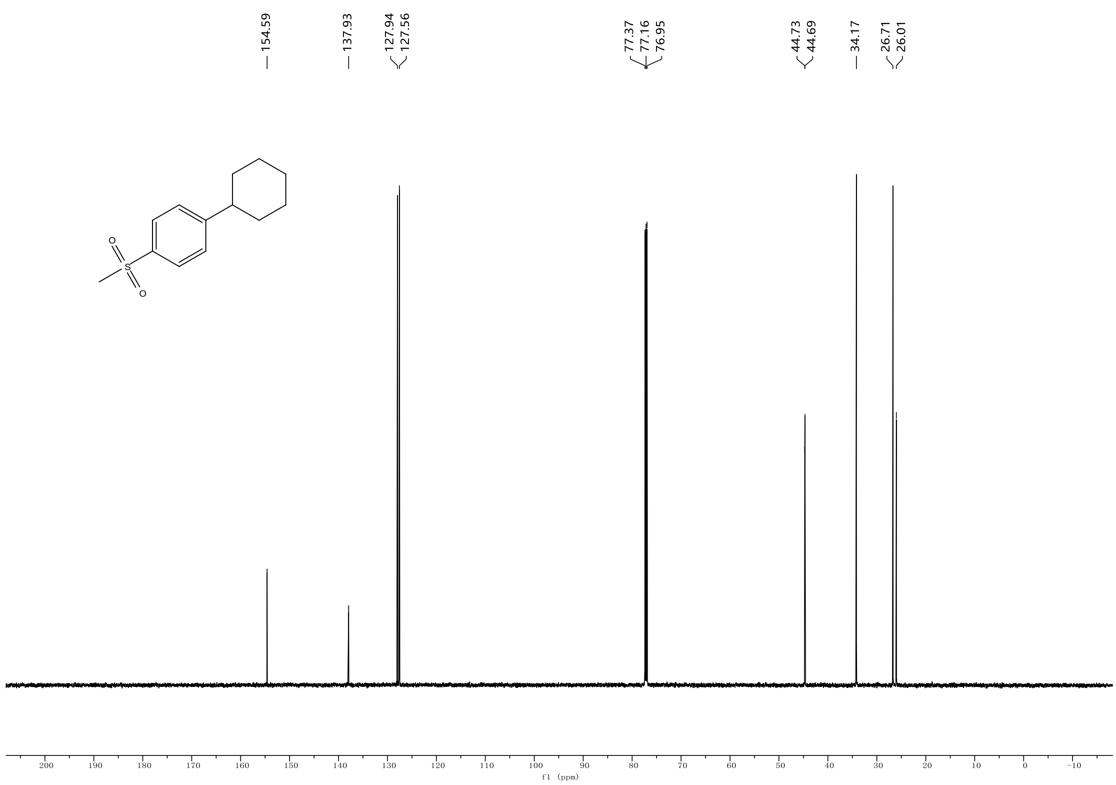

Supplementary Figure 106. ^{13}C NMR spectra of compound **25** (151 MHz, r.t., $CDCl_3$).

Compound 26

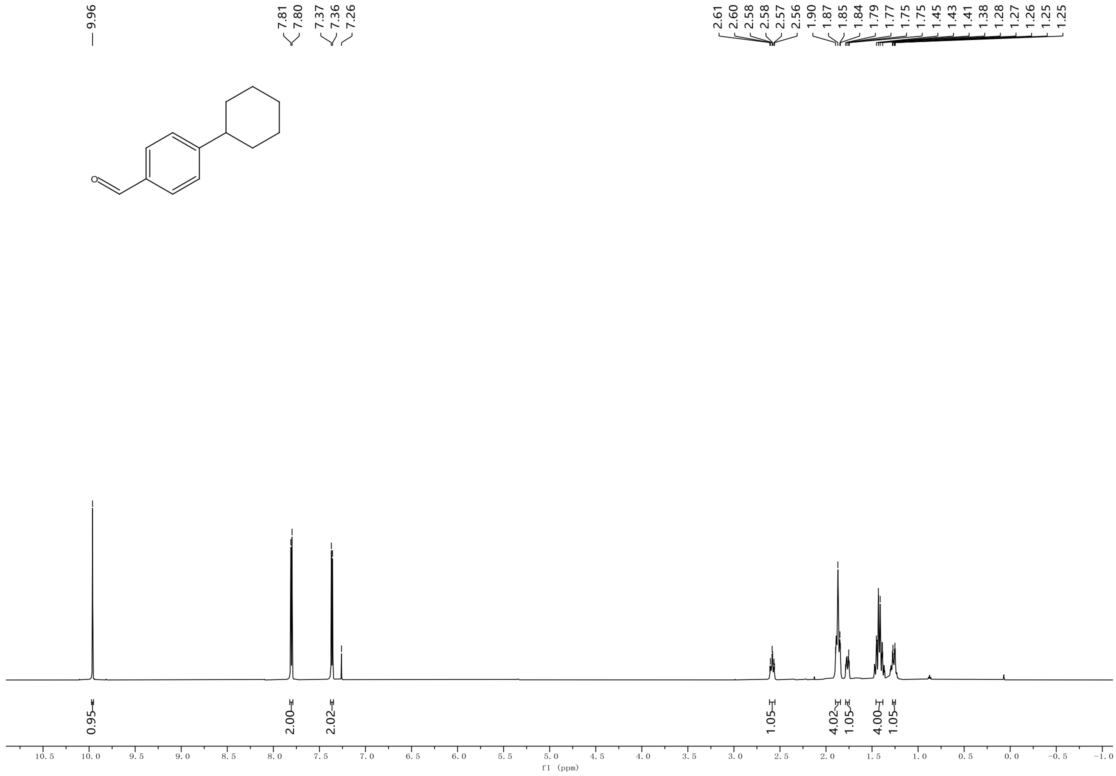

Supplementary Figure 107. ^{1}H NMR spectra of compound **26** (600 MHz, r.t., $CDCl_3$).

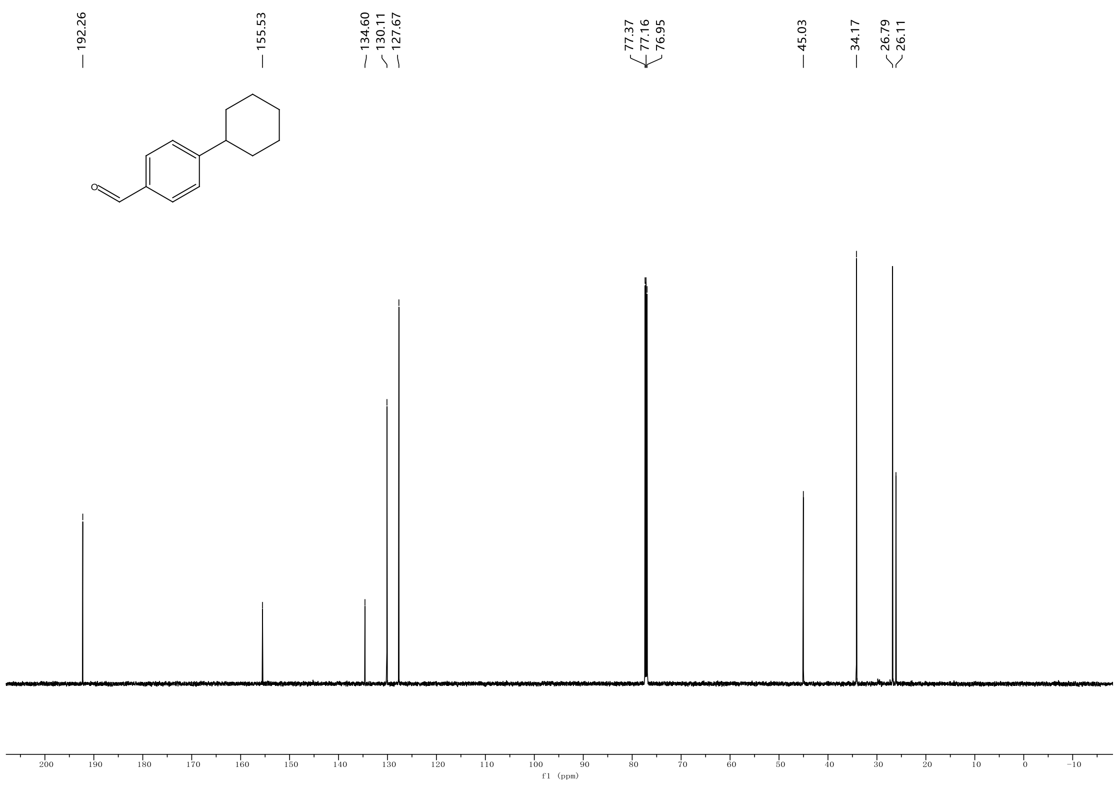

Supplementary Figure 108. ^{13}C NMR spectra of compound **26** (151 MHz, r.t., $CDCl_3$).

Compound 27

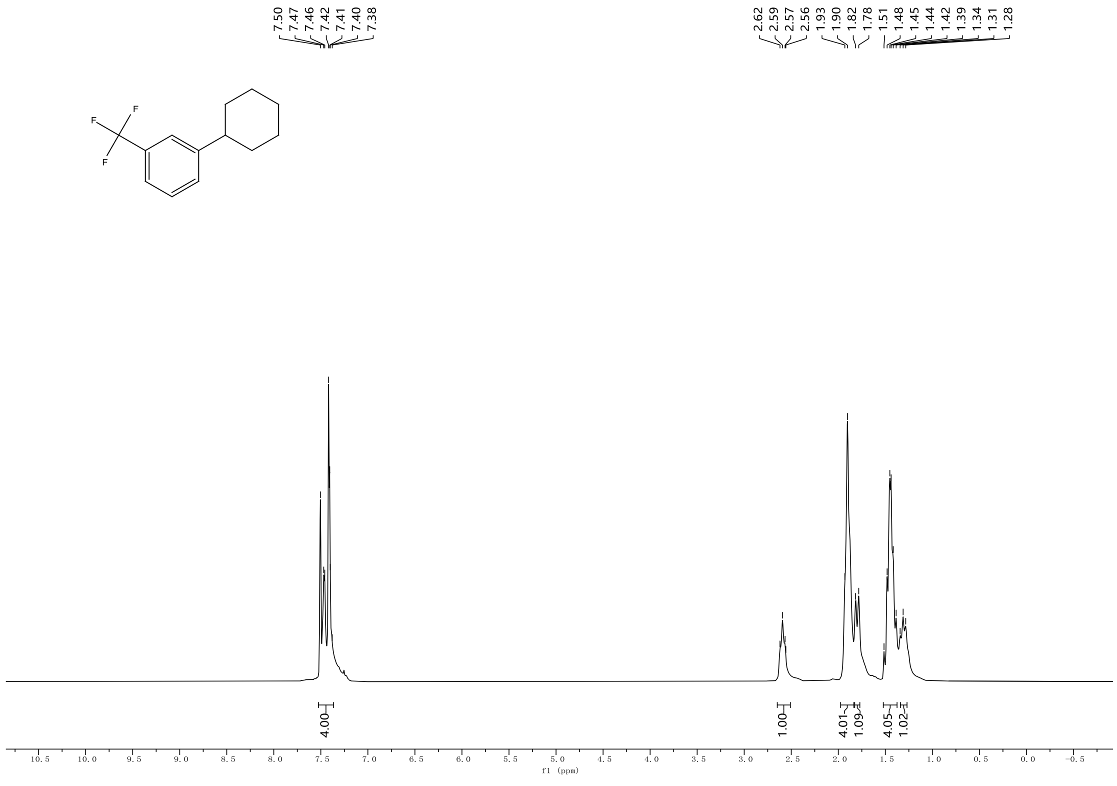

Supplementary Figure 109. ^{1}H NMR spectra of compound **27** (400 MHz, r.t., $CDCl_3$).

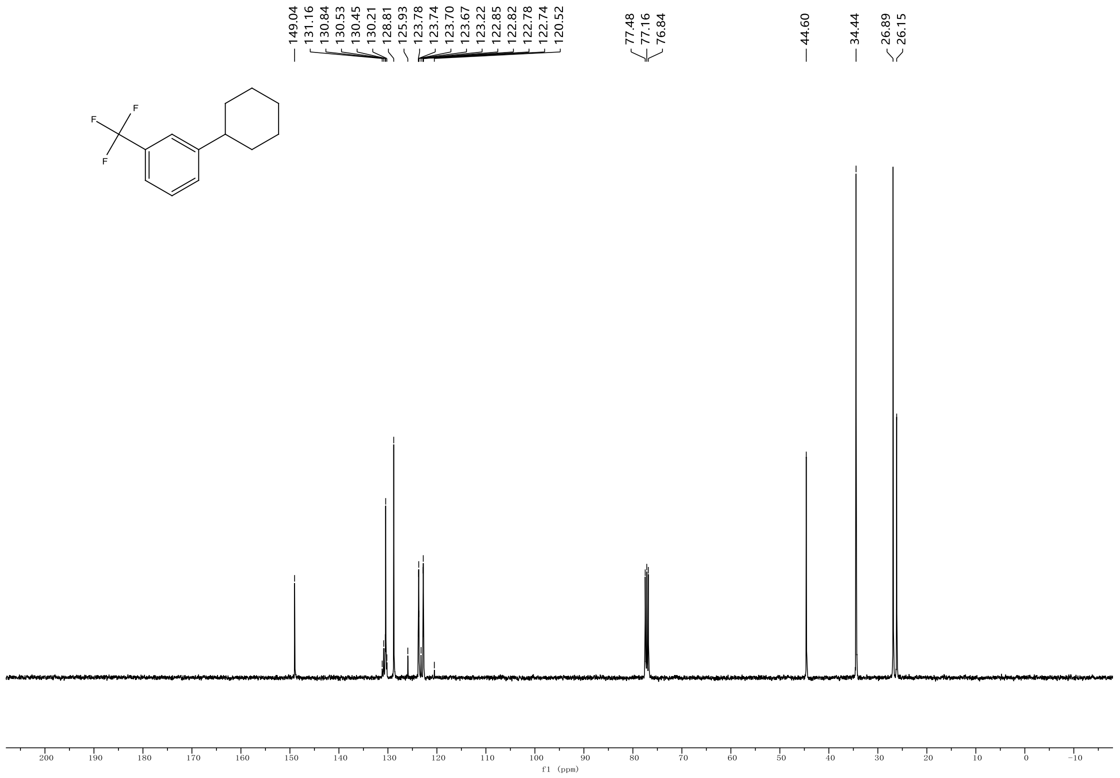

Supplementary Figure 110. ^{13}C NMR spectra of compound **27** (101 MHz, r.t., $CDCl_3$).

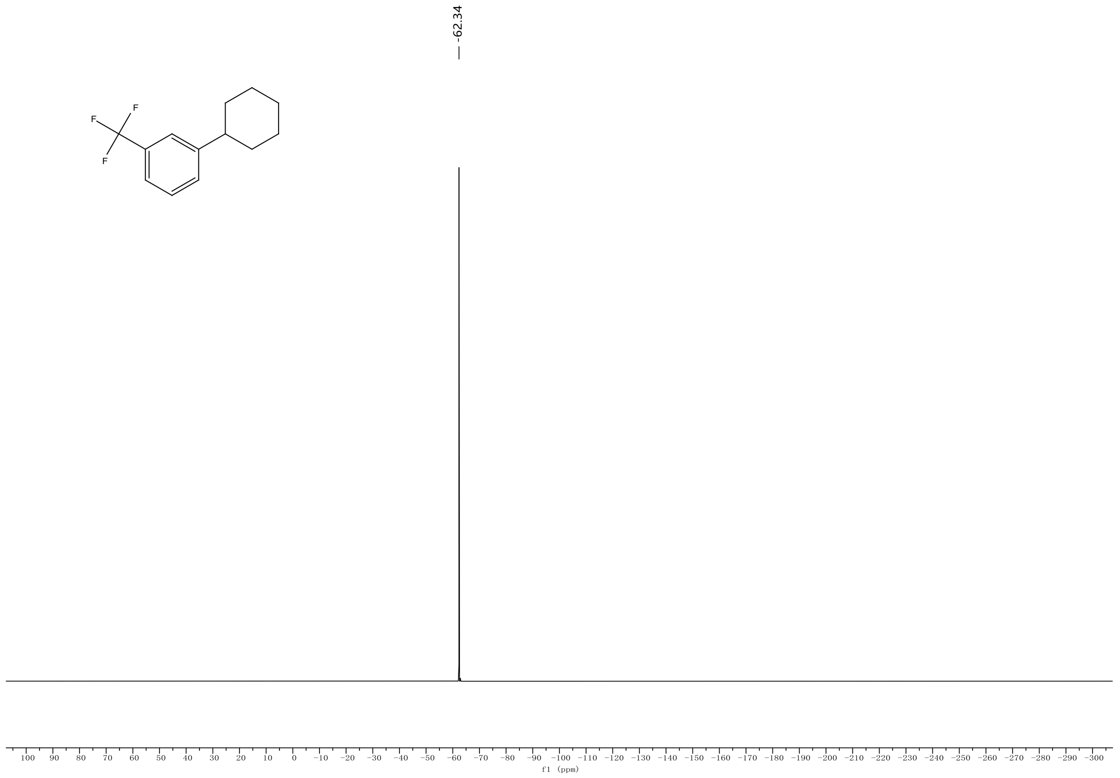

Supplementary Figure 111. ^{19}F NMR spectra of compound **27** (376 MHz, r.t., $CDCl_3$).

Compound 28

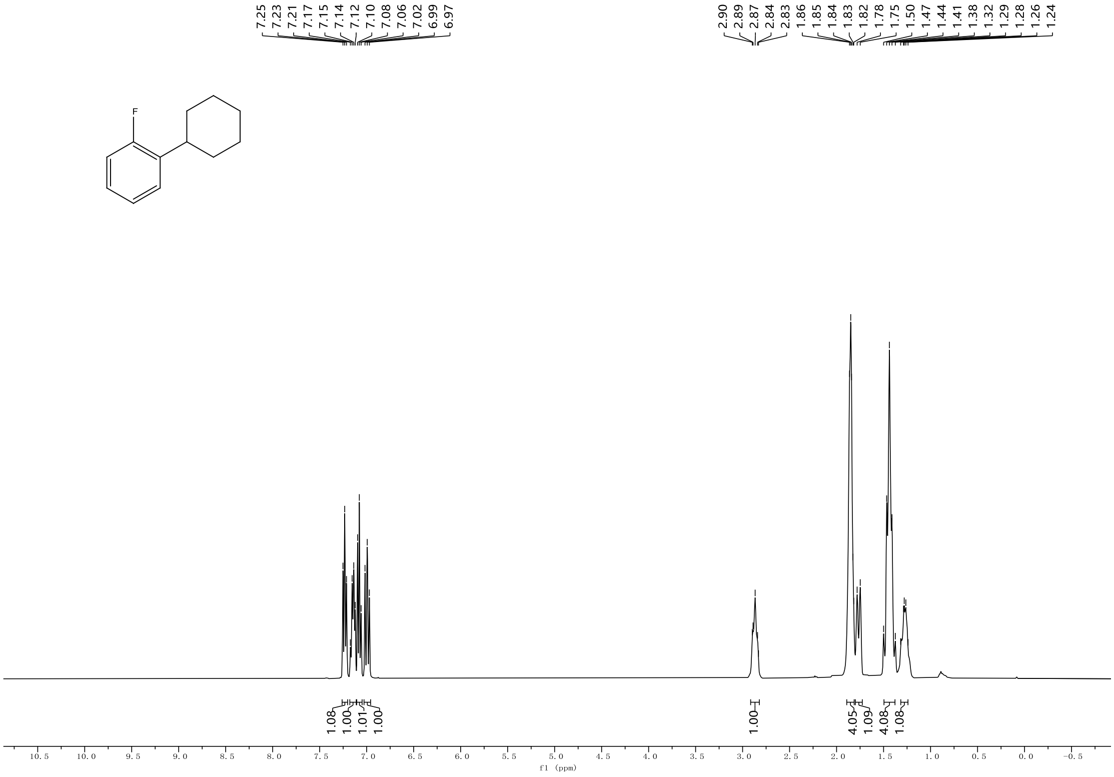

Supplementary Figure 112. ^{1}H NMR spectra of compound **28** (400 MHz, r.t., $CDCl_3$).

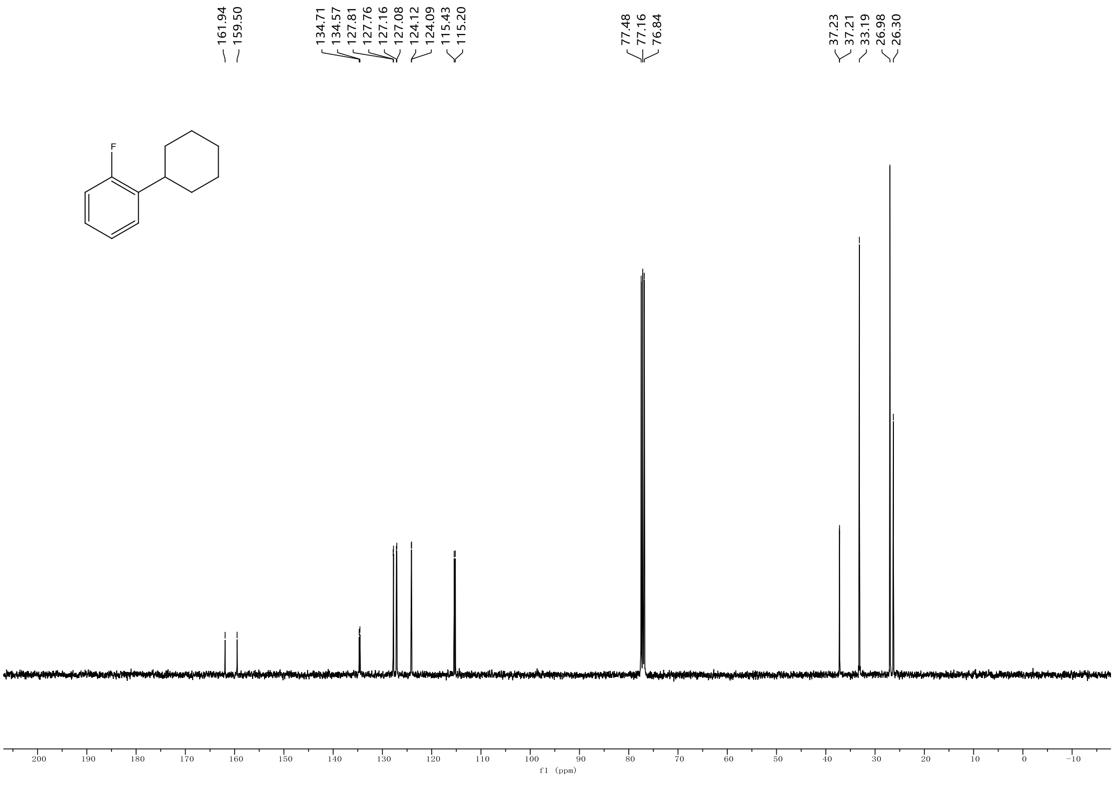

Supplementary Figure 113. ^{13}C NMR spectra of compound **28** (101 MHz, r.t., $CDCl_3$).

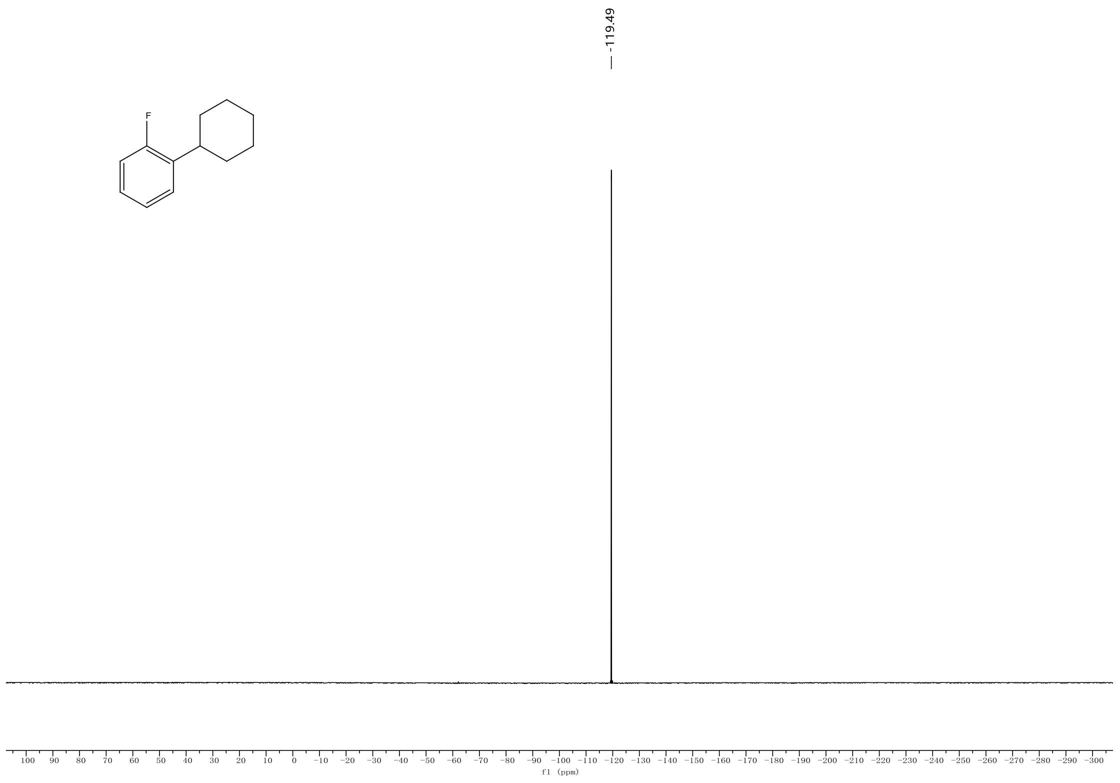

Supplementary Figure 114. ^{19}F NMR spectra of compound **28** (376 MHz, r.t., $CDCl_3$).

Compound 29

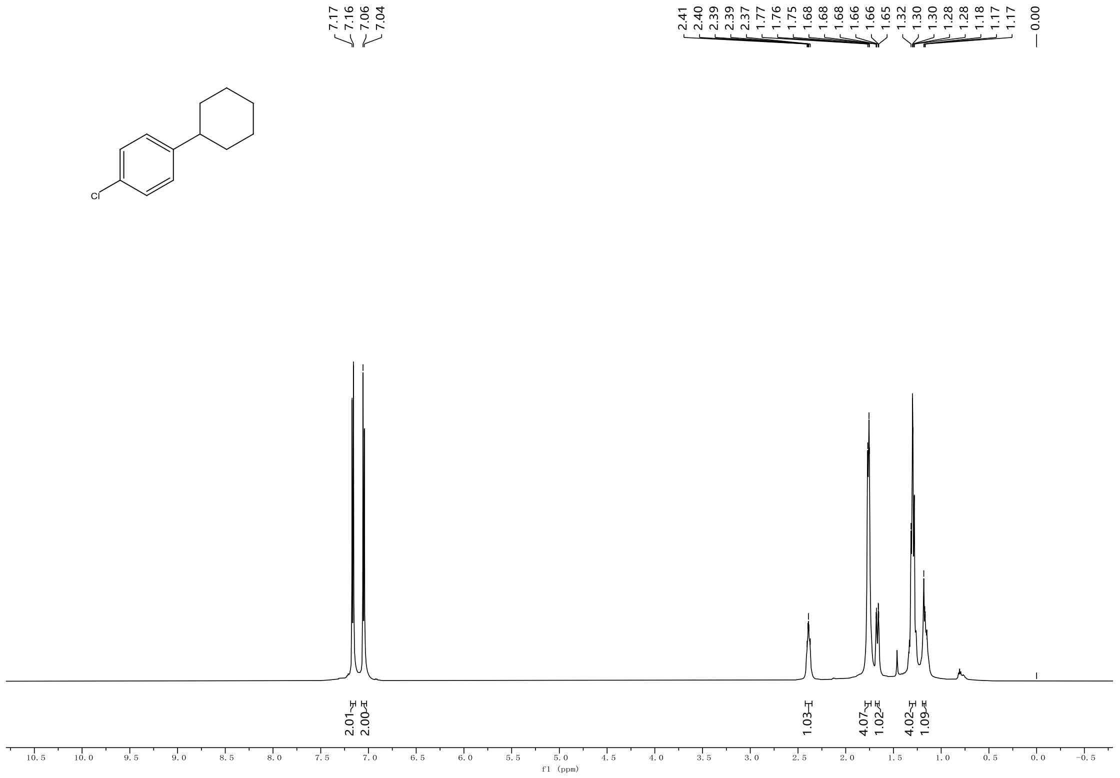

Supplementary Figure 115. ^{1}H NMR spectra of compound **29** (600 MHz, r.t., $CDCl_3$).

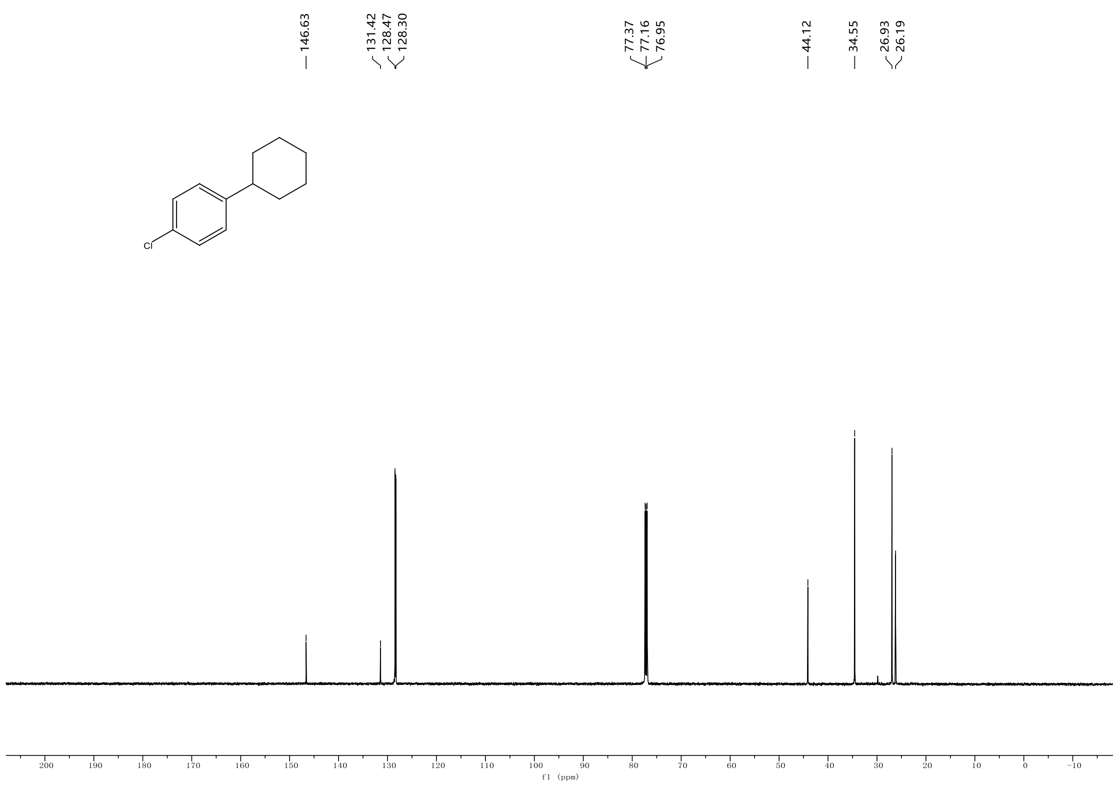

Supplementary Figure 116. ^{13}C NMR spectra of compound **29** (151 MHz, r.t., $CDCl_3$).

Compound 30

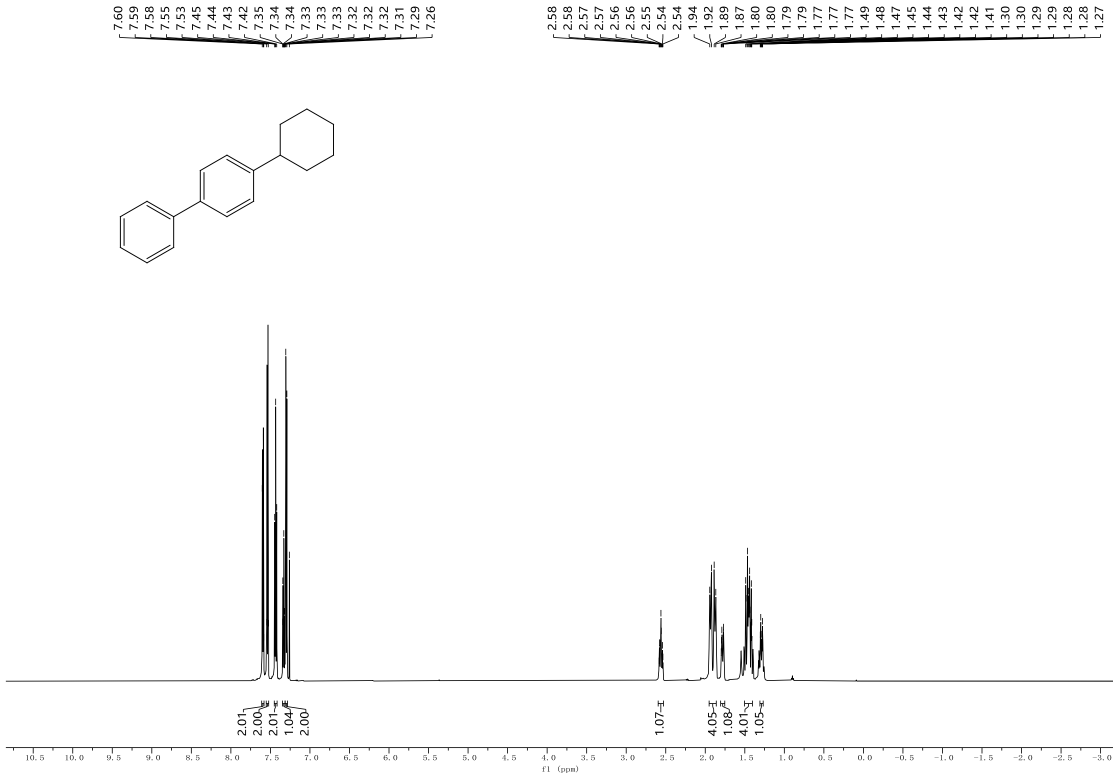

Supplementary Figure 117. ^{1}H NMR spectra of compound **30** (600 MHz, r.t., $CDCl_3$).

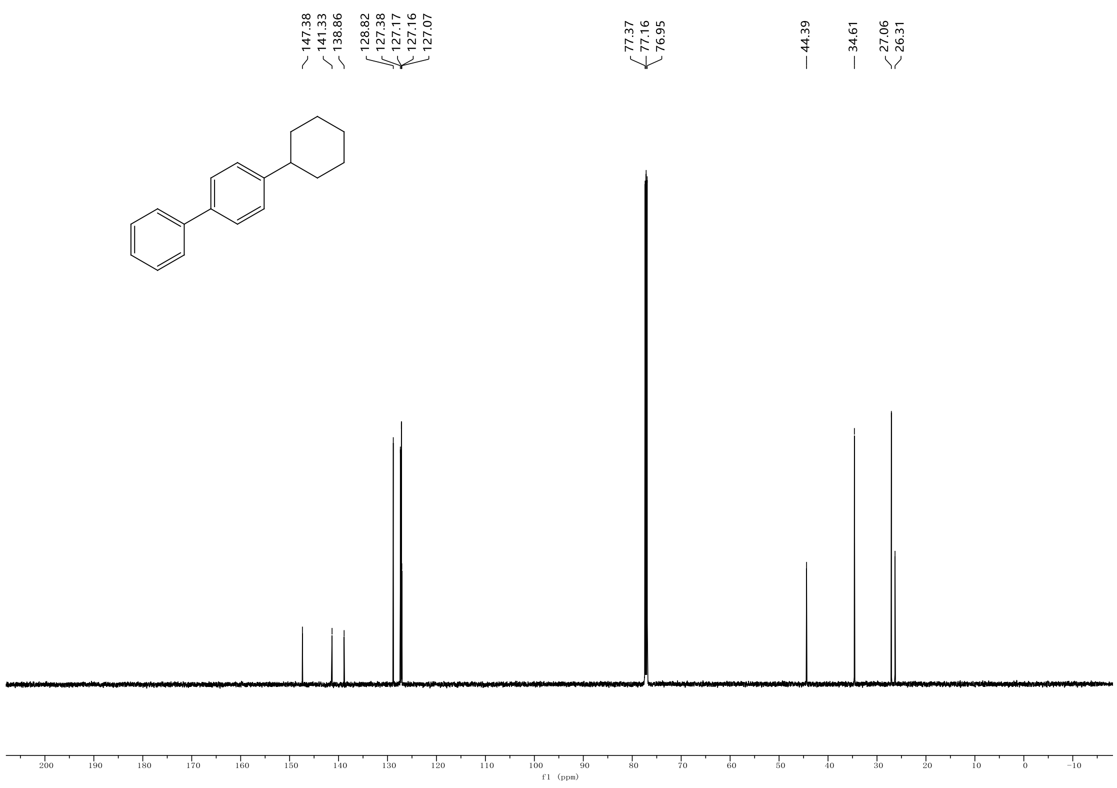

Supplementary Figure 118. ^{13}C NMR spectra of compound **30** (151 MHz, r.t., $CDCl_3$).

Compound 31

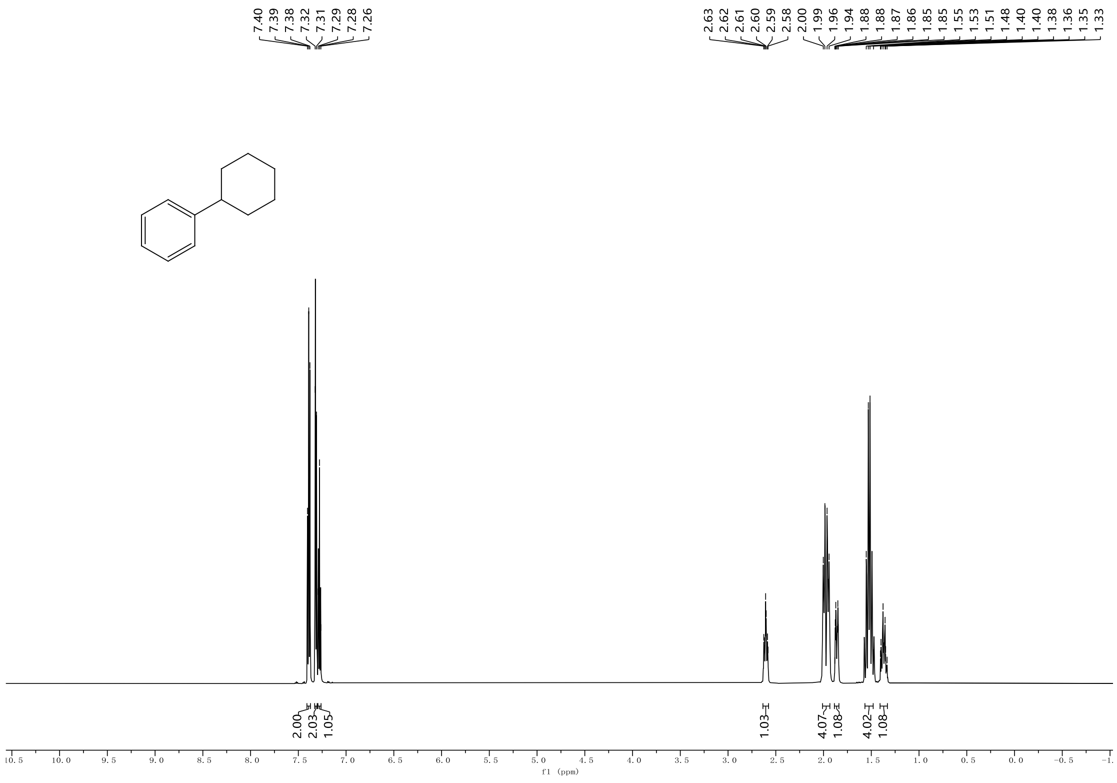

Supplementary Figure 119. ^{1}H NMR spectra of compound **31** (600 MHz, r.t., $CDCl_3$).

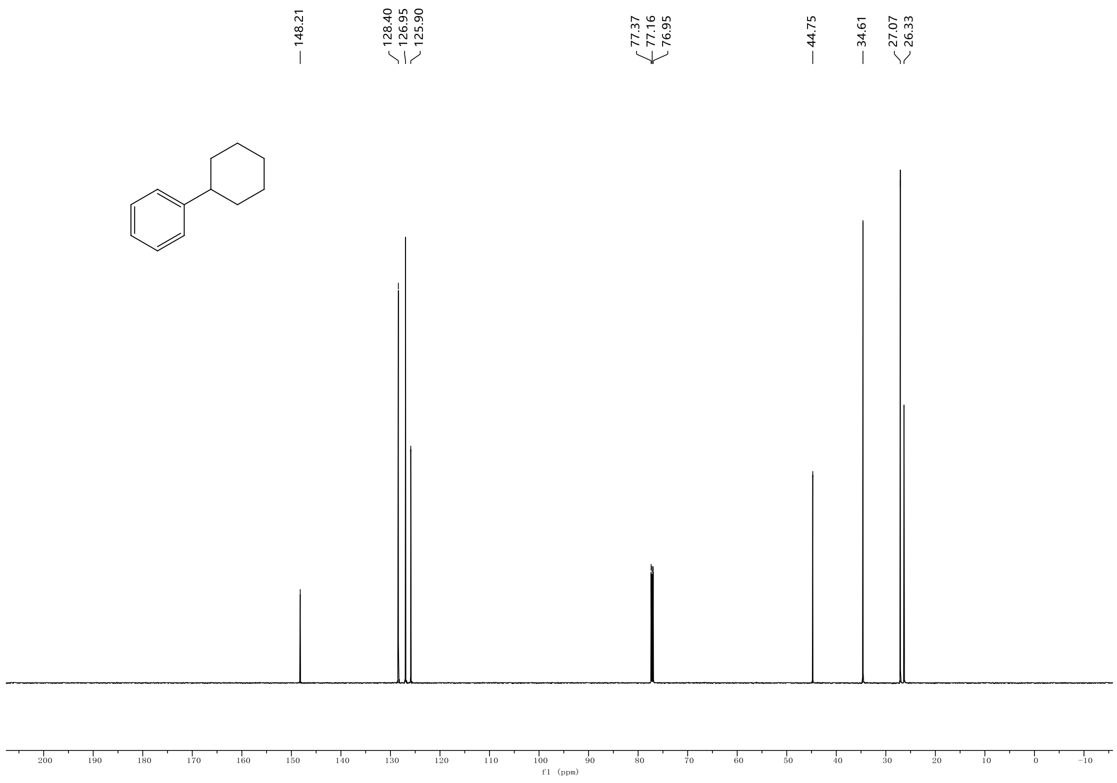

Supplementary Figure 120. ^{13}C NMR spectra of compound **31** (151 MHz, r.t., $CDCl_3$).

Compound 32

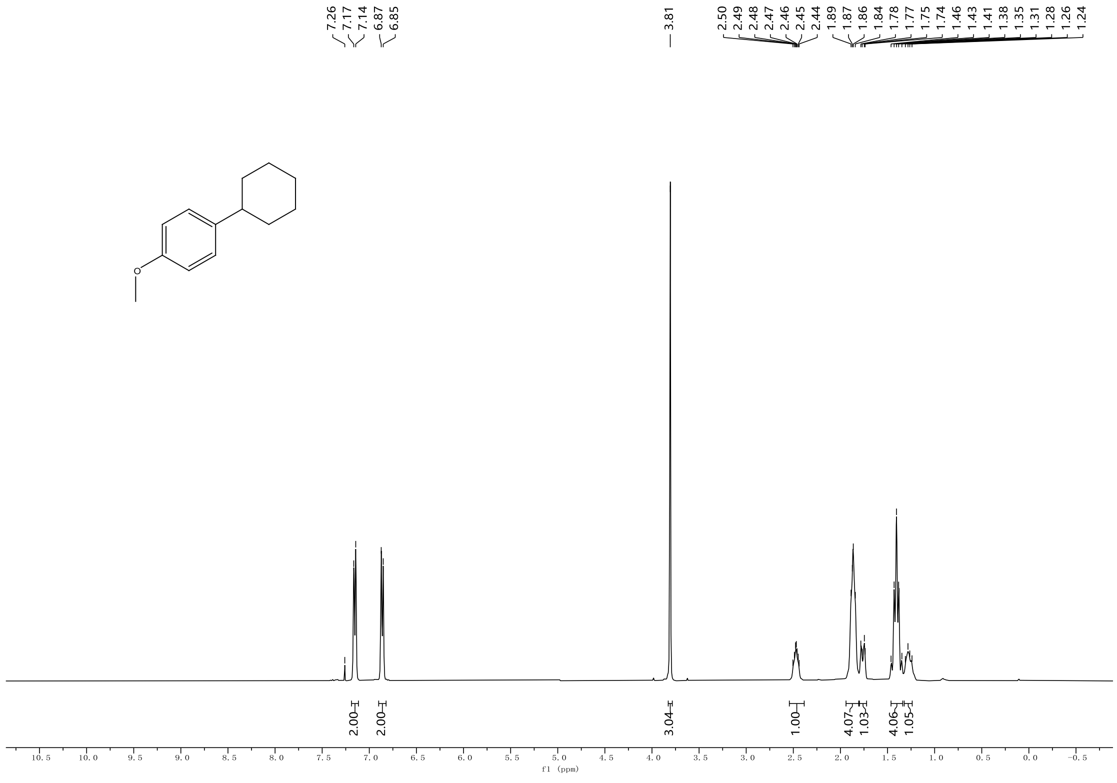

Supplementary Figure 121. ^{1}H NMR spectra of compound **32** (400 MHz, r.t., $CDCl_3$).

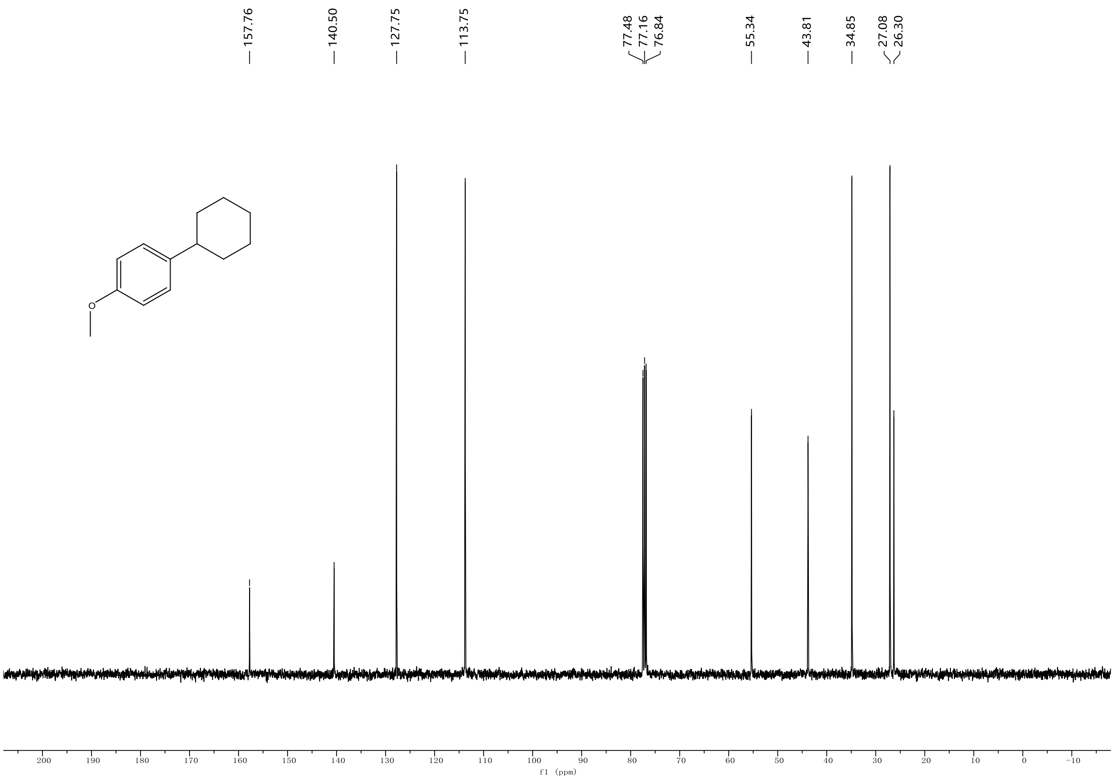

Supplementary Figure 122. ^{13}C NMR spectra of compound **32** (101 MHz, r.t., $CDCl_3$).

Compound 33

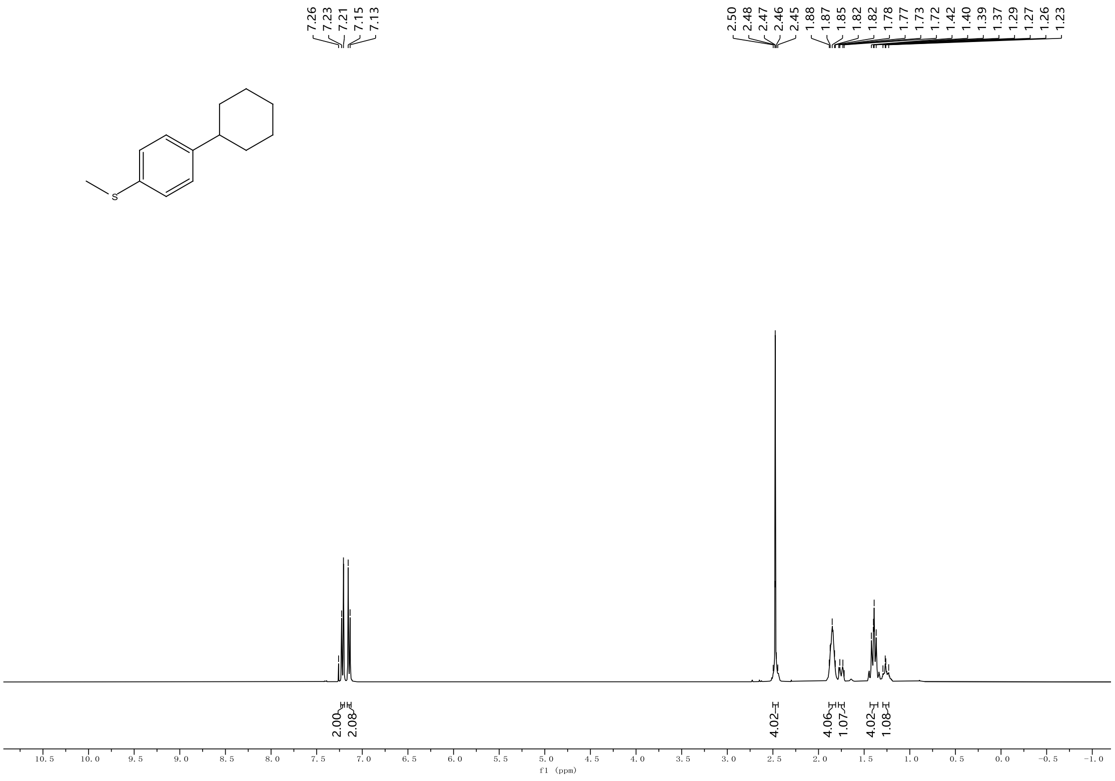

Supplementary Figure 123. ^{1}H NMR spectra of compound **33** (400 MHz, r.t., $CDCl_3$).

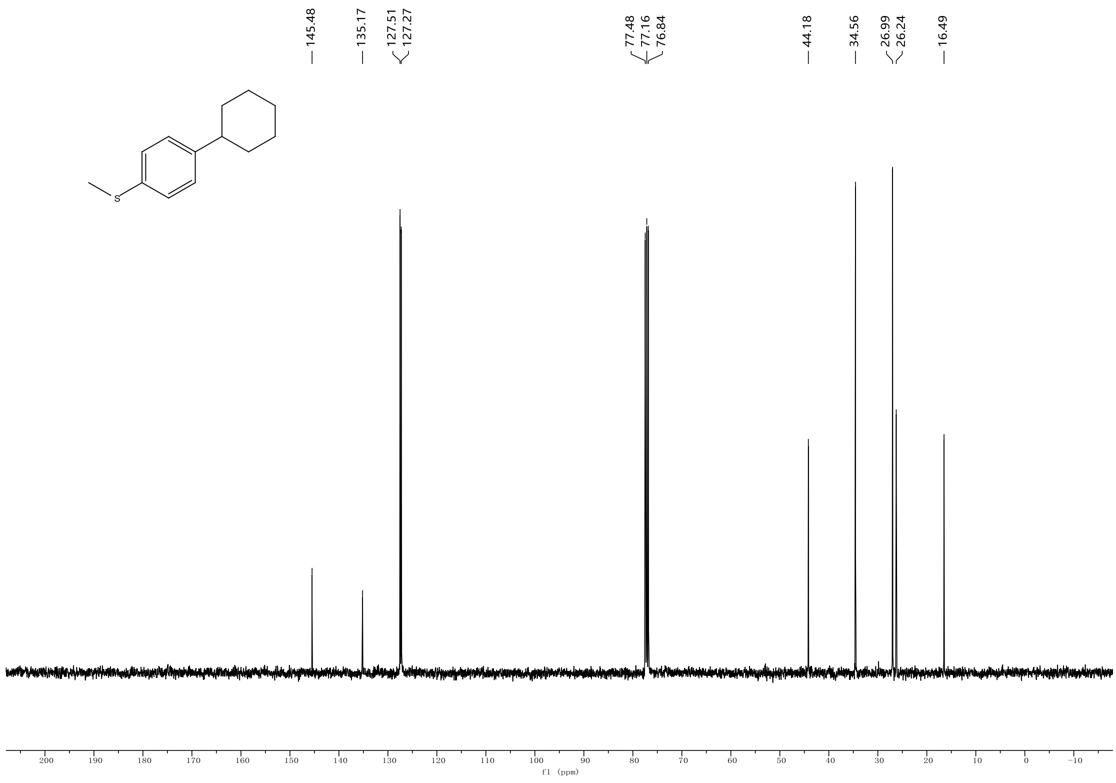

Supplementary Figure 124. ^{13}C NMR spectra of compound **33** (101 MHz, r.t., $CDCl_3$).

Compound 34

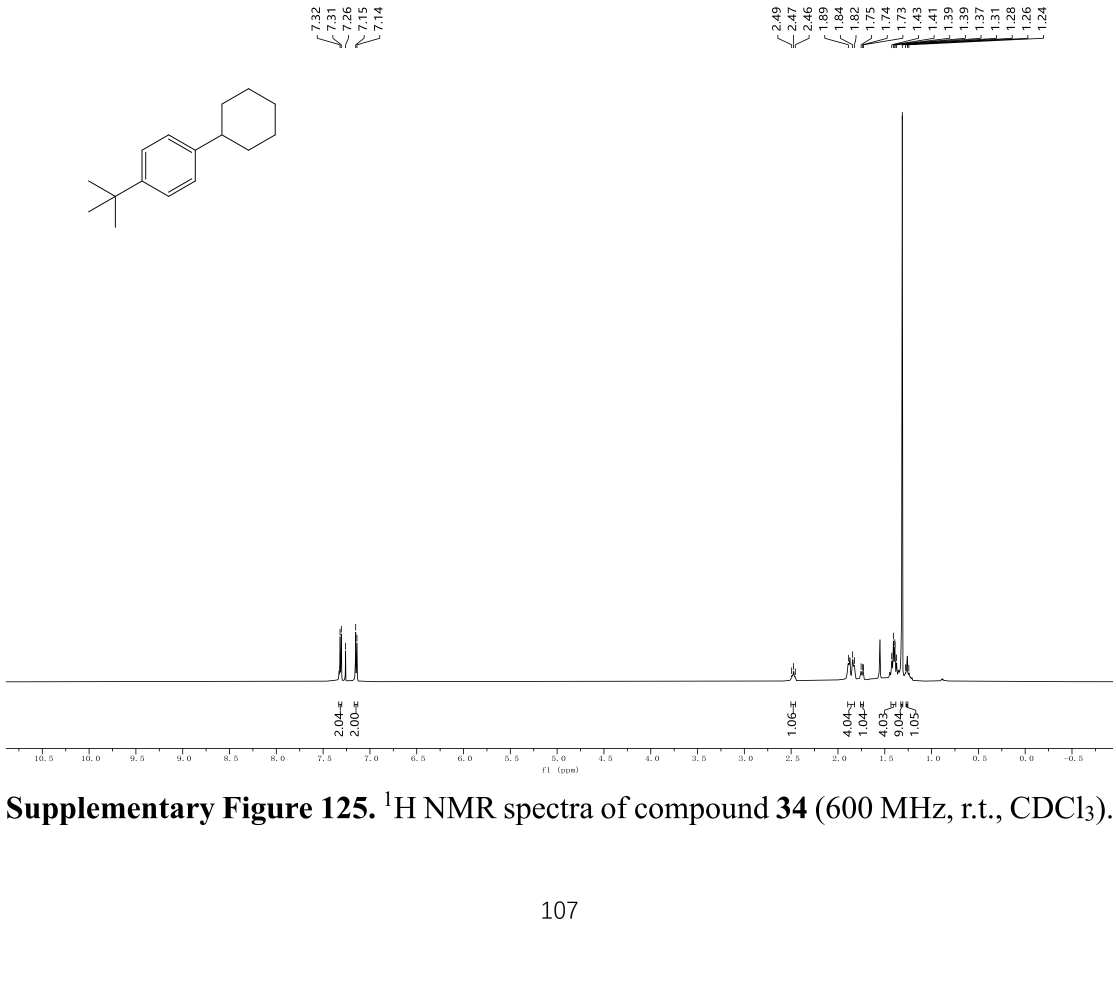

Supplementary Figure 125. ^{1}H NMR spectra of compound **34** (600 MHz, r.t., $CDCl_3$).

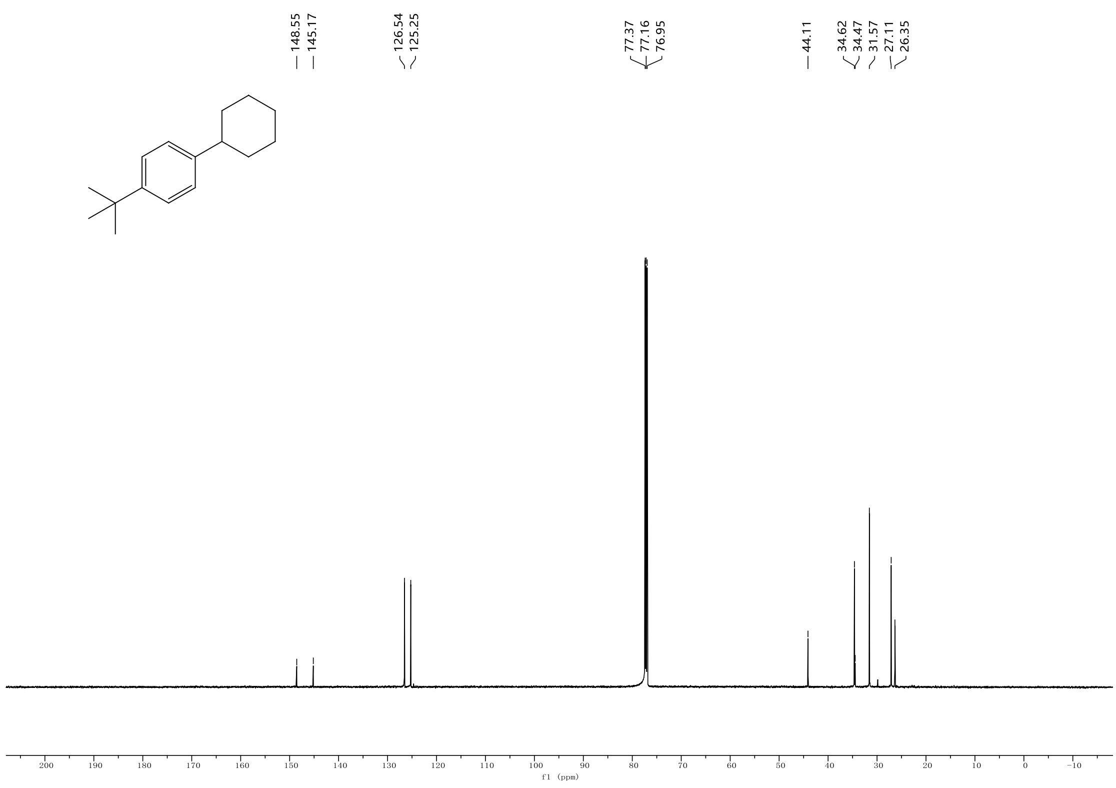

Supplementary Figure 126. ^{13}C NMR spectra of compound **34** (151 MHz, r.t., $CDCl_3$).

Compound 35

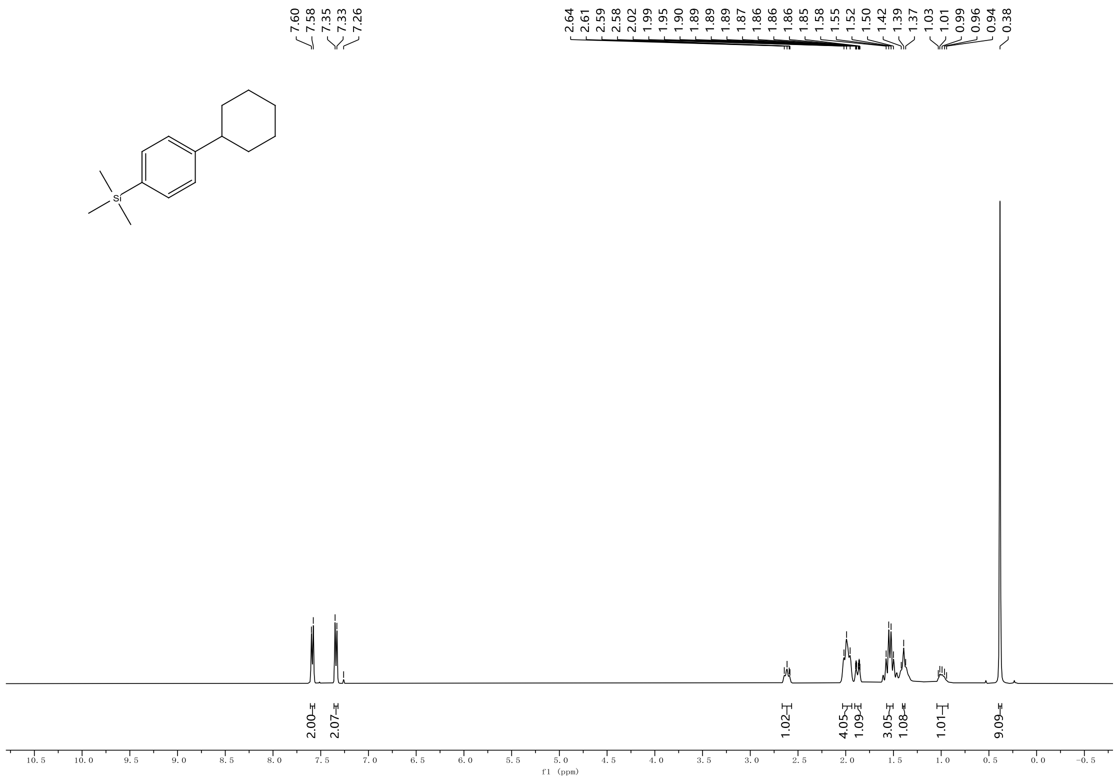

Supplementary Figure 127. ^{1}H NMR spectra of compound **35** (400 MHz, r.t., $CDCl_3$).

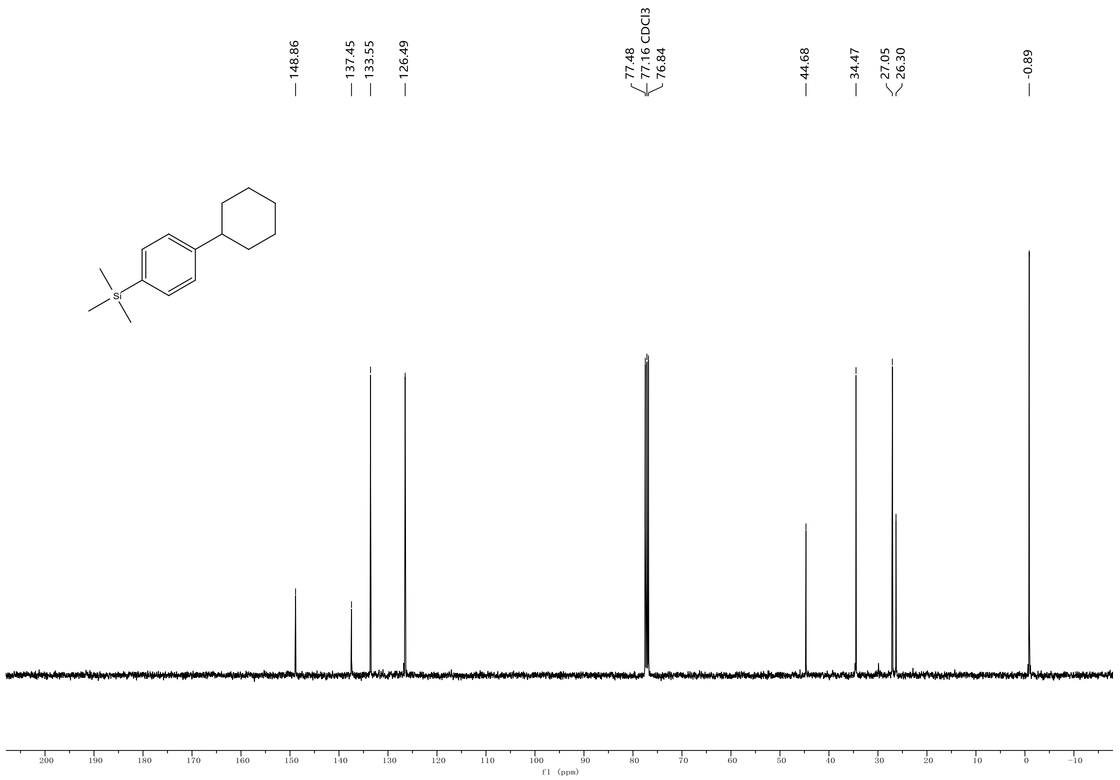

Supplementary Figure 128. ^{13}C NMR spectra of compound **35** (101 MHz, r.t., $CDCl_3$).

Compound 36

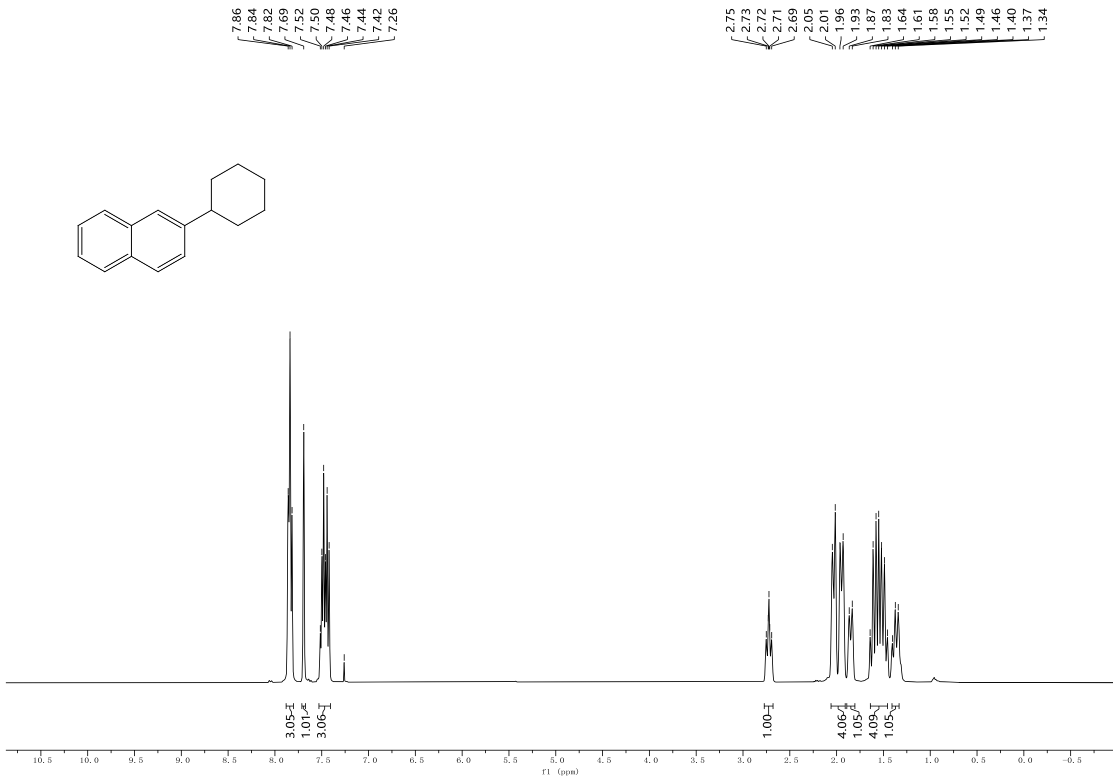

Supplementary Figure 129. ^{1}H NMR spectra of compound **36** (400 MHz, r.t., $CDCl_3$).

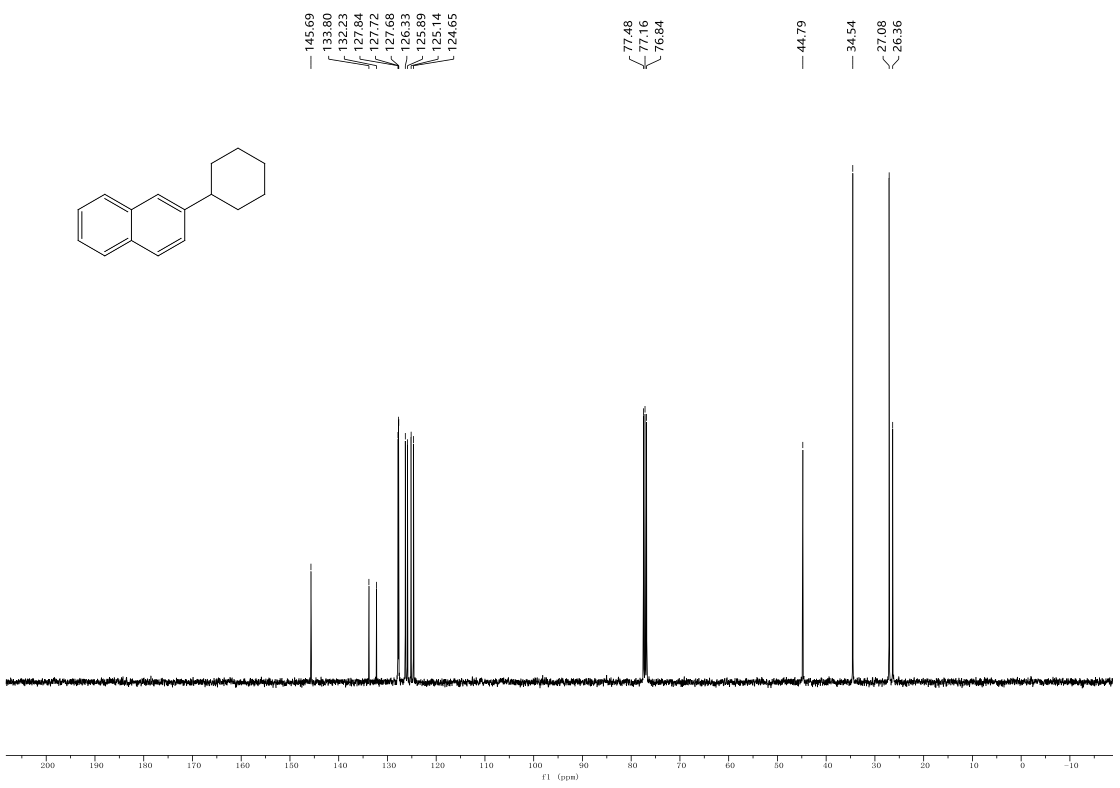

Supplementary Figure 130. ^{13}C NMR spectra of compound **36** (101 MHz, r.t., $CDCl_3$).

Compound 37

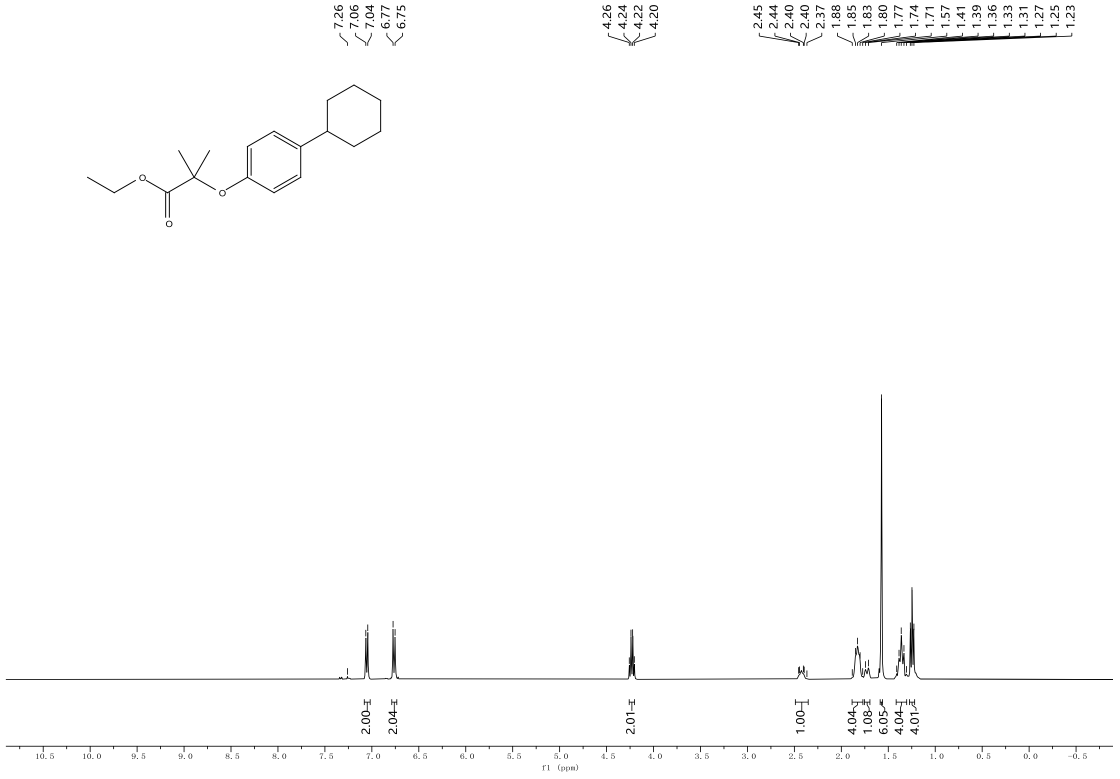

Supplementary Figure 131. ^{1}H NMR spectra of compound **37** (400 MHz, r.t., $CDCl_3$).

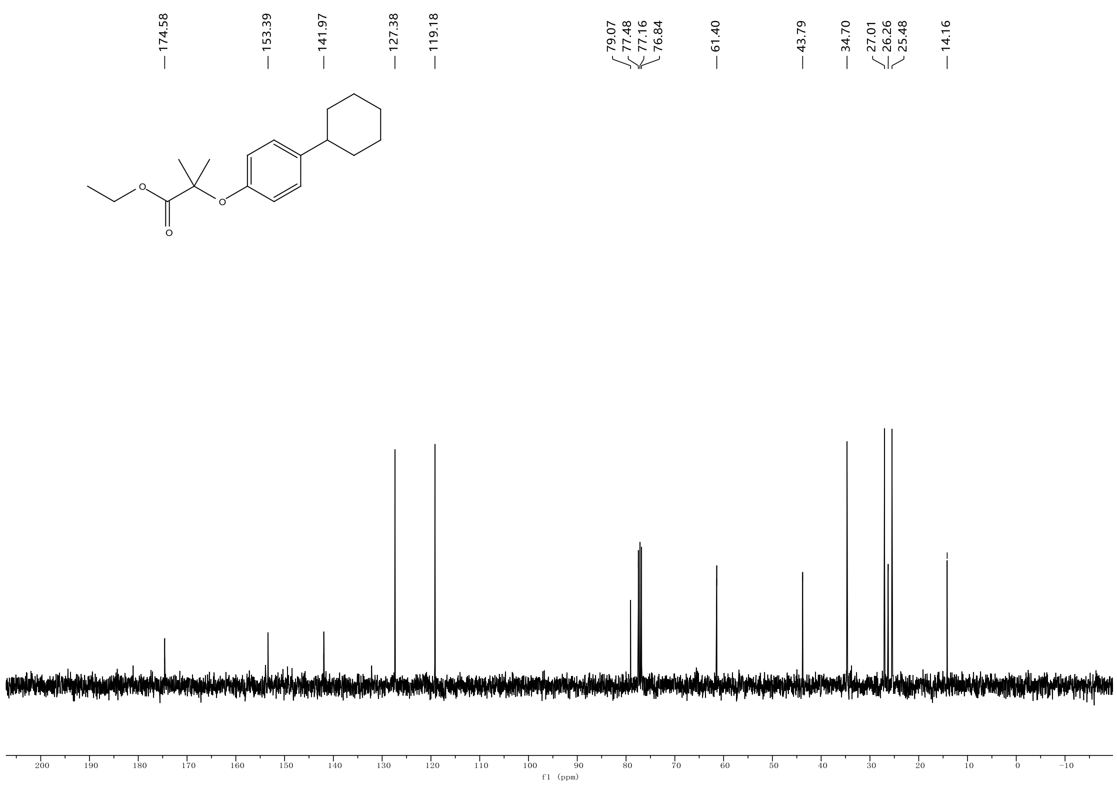

Supplementary Figure 132. ^{13}C NMR spectra of compound **37** (101 MHz, r.t., $CDCl_3$).

Compound 38

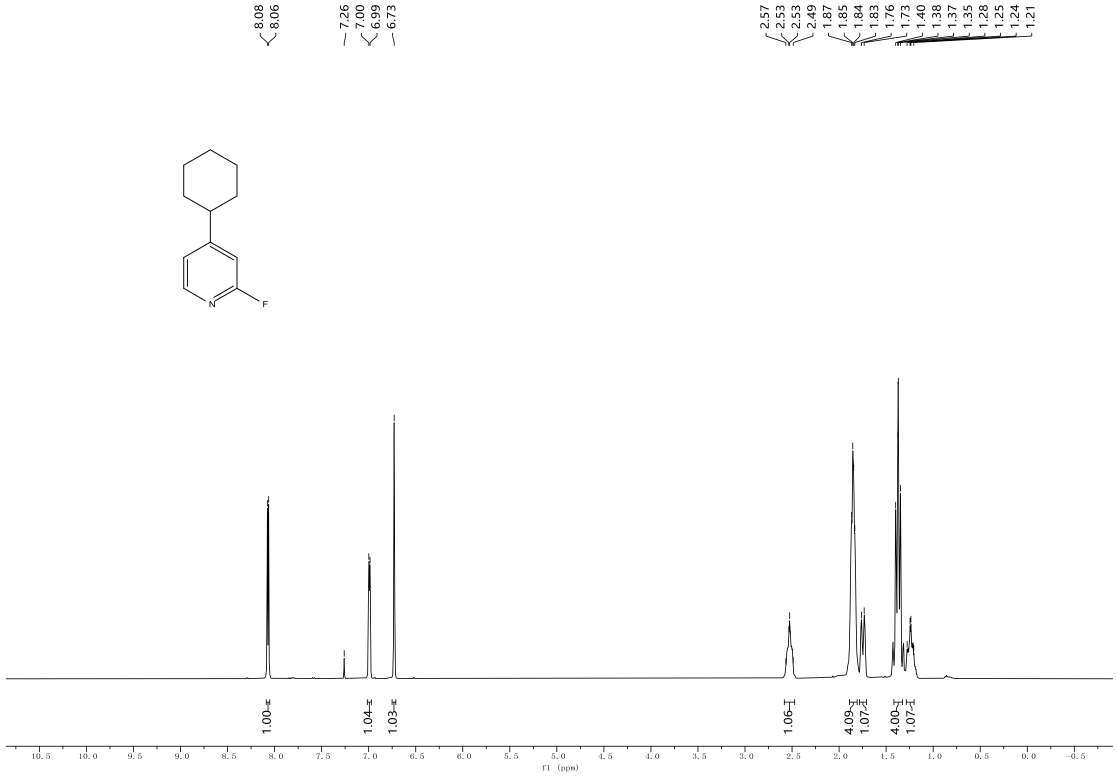

Supplementary Figure 133. ^{1}H NMR spectra of compound **38** (400 MHz, r.t., $CDCl_3$).

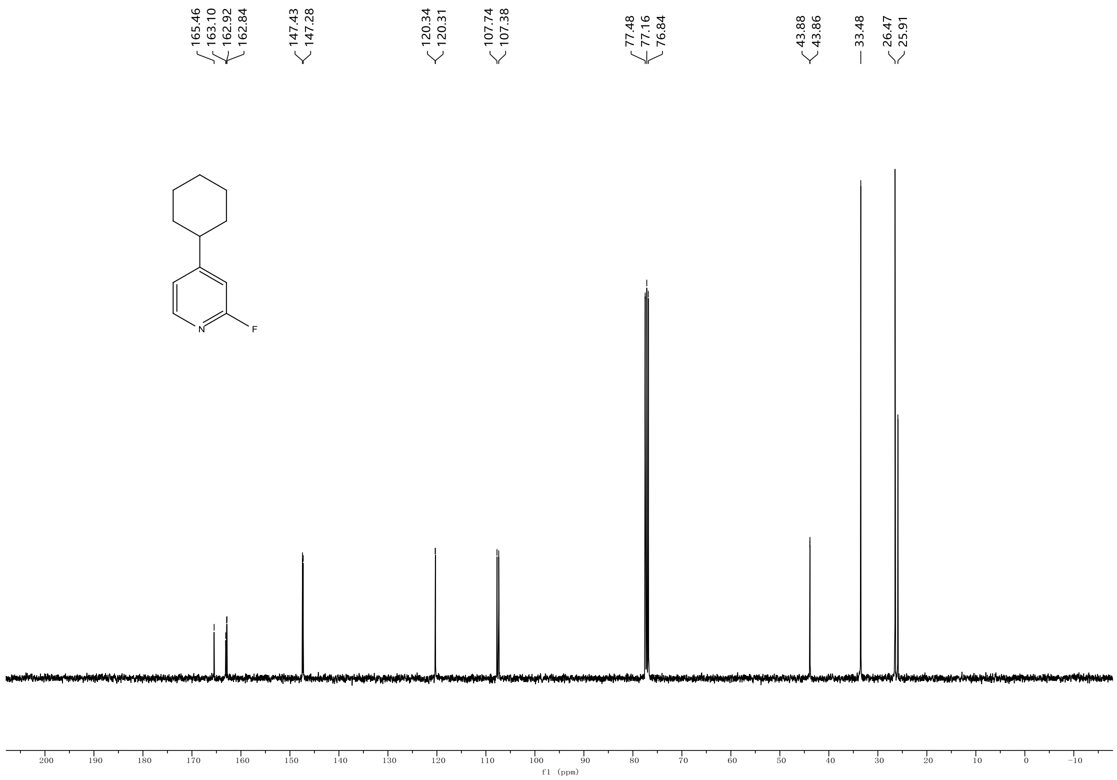

Supplementary Figure 134. ^{13}C NMR spectra of compound **38** (101 MHz, r.t., $CDCl_3$).

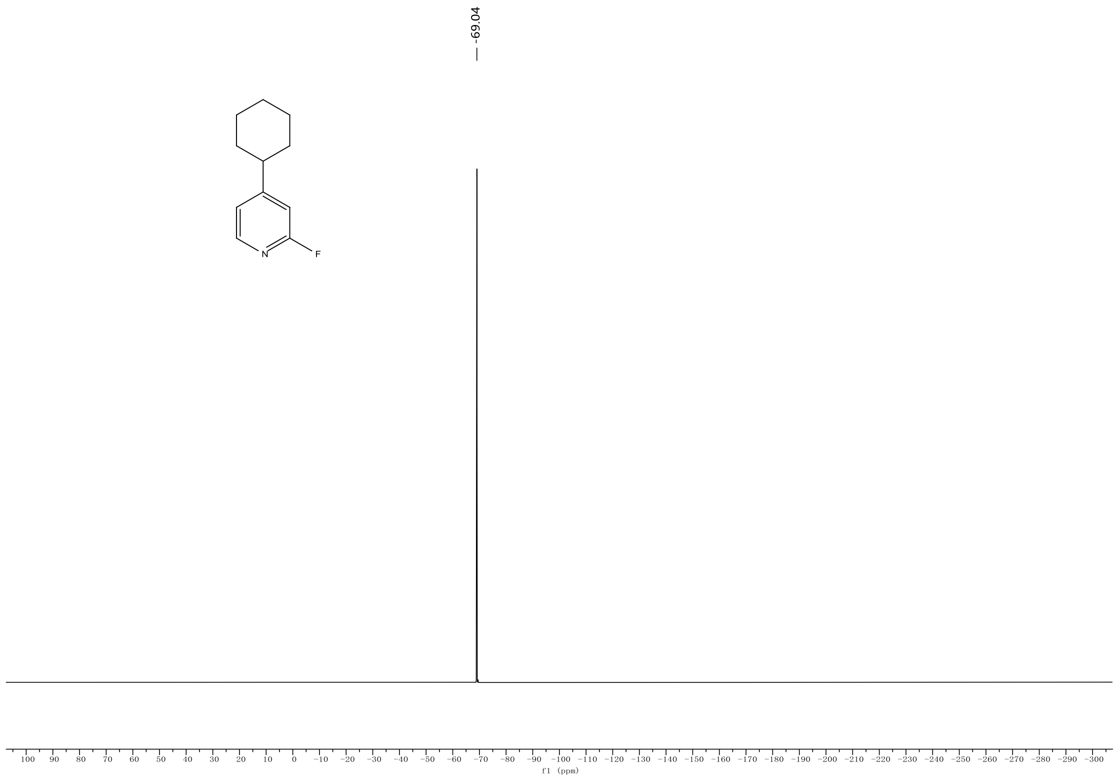

Supplementary Figure 135. ^{19}F NMR spectra of compound **38** (376 MHz, r.t., $CDCl_3$).

Compound 39

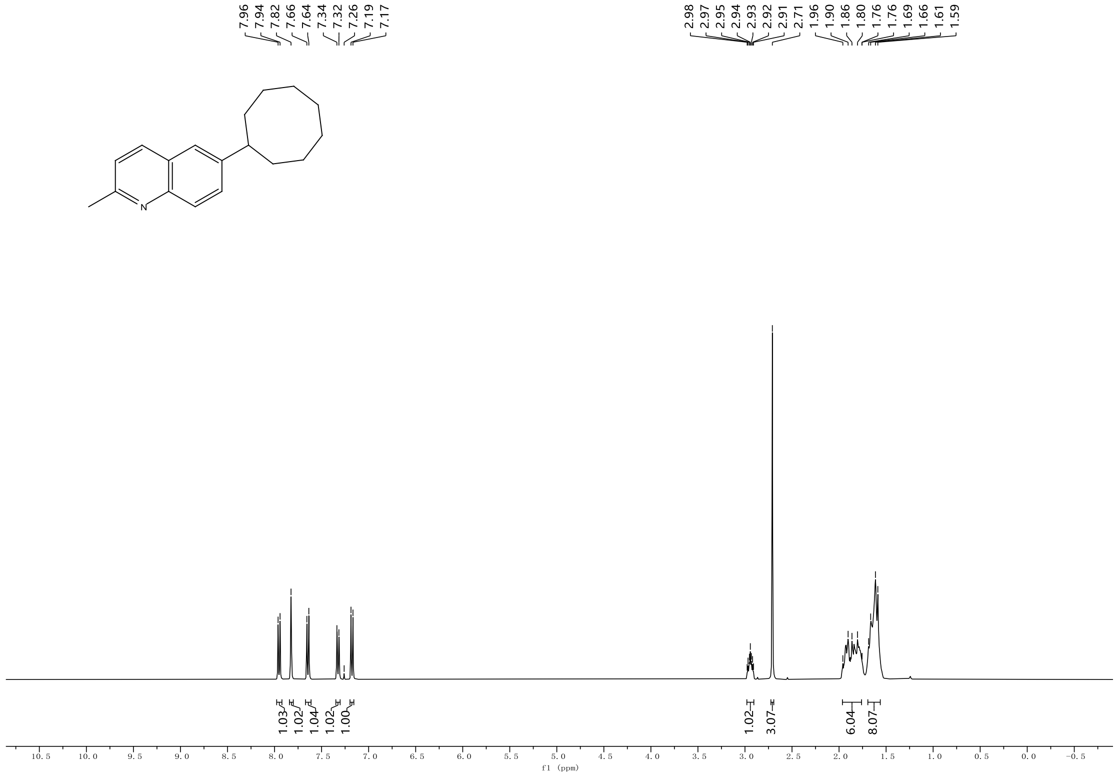

Supplementary Figure 136. ^{1}H NMR spectra of compound **39** (400 MHz, r.t., $CDCl_3$).

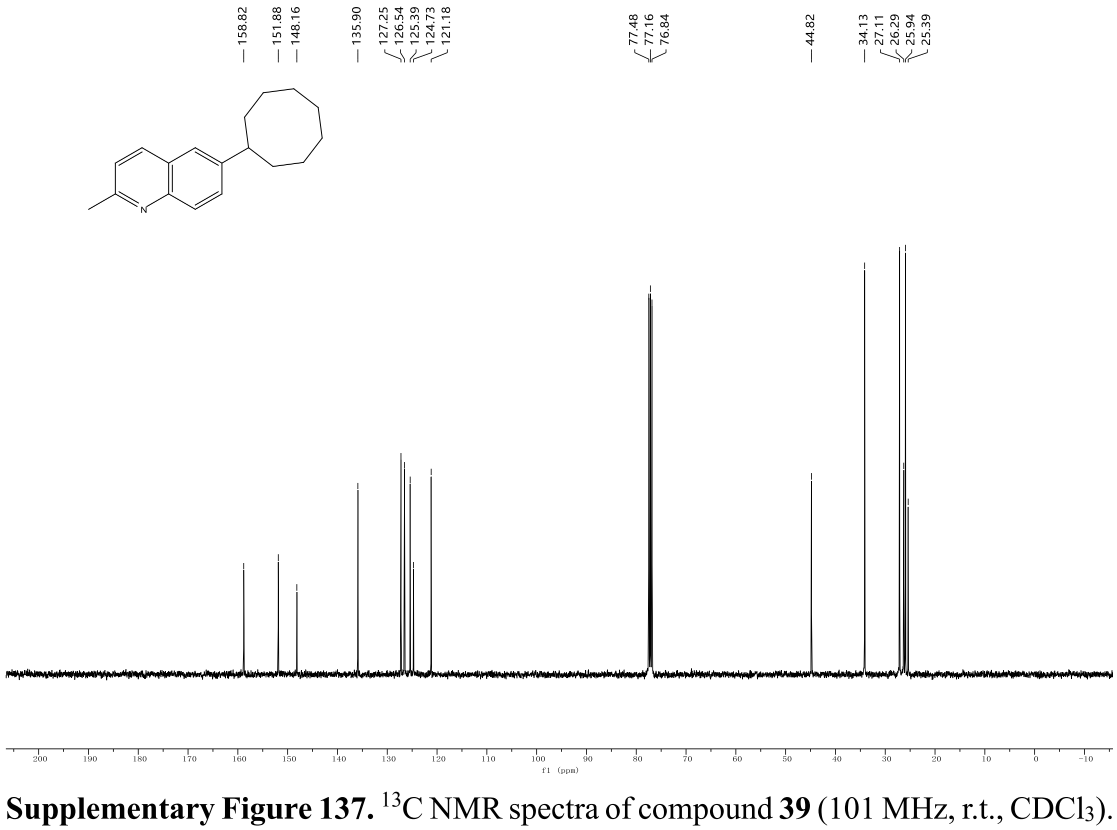

Supplementary Figure 137. ^{13}C NMR spectra of compound **39** (101 MHz, r.t., $CDCl_3$).

Compound 40

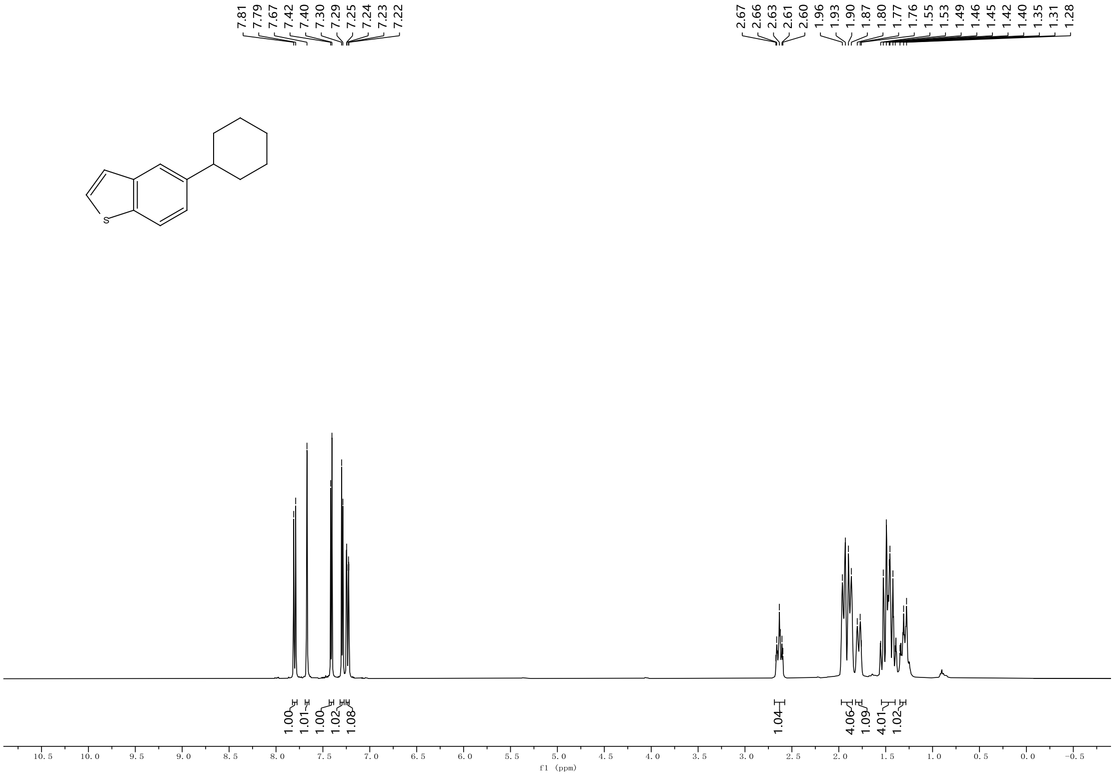

Supplementary Figure 138. ^{1}H NMR spectra of compound **40** (400 MHz, r.t., $CDCl_3$).

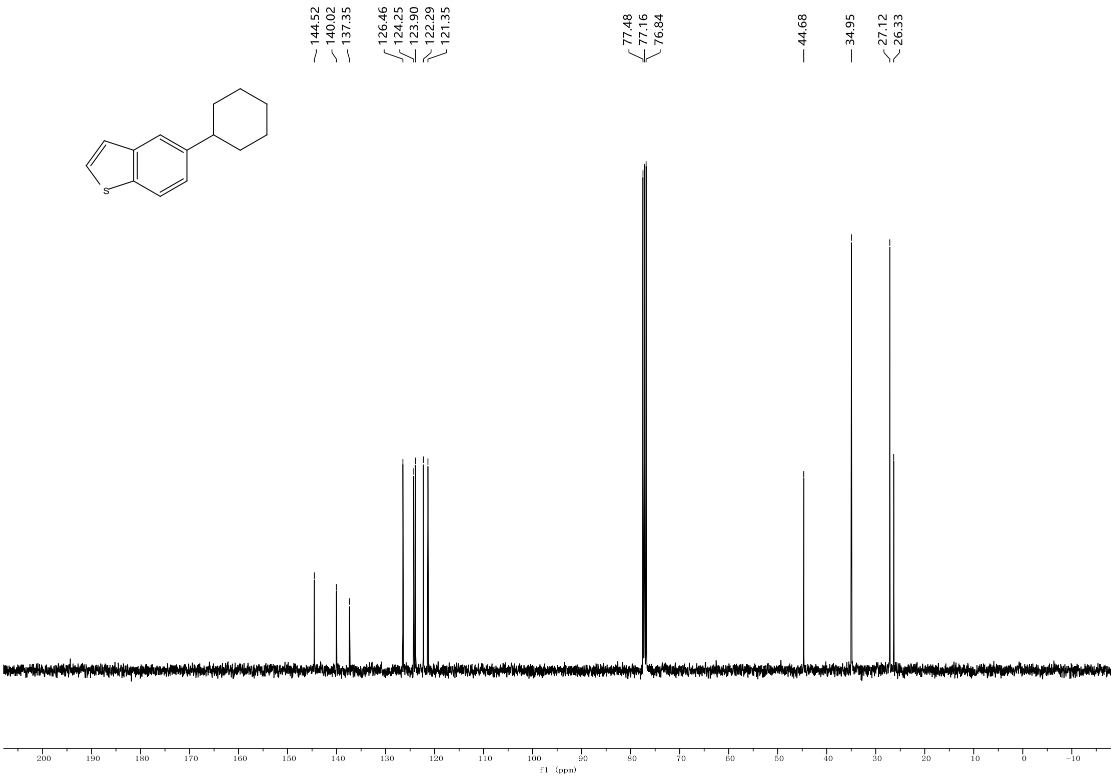

Supplementary Figure 139. ^{13}C NMR spectra of compound **40** (101 MHz, r.t., $CDCl_3$).

Compound 41

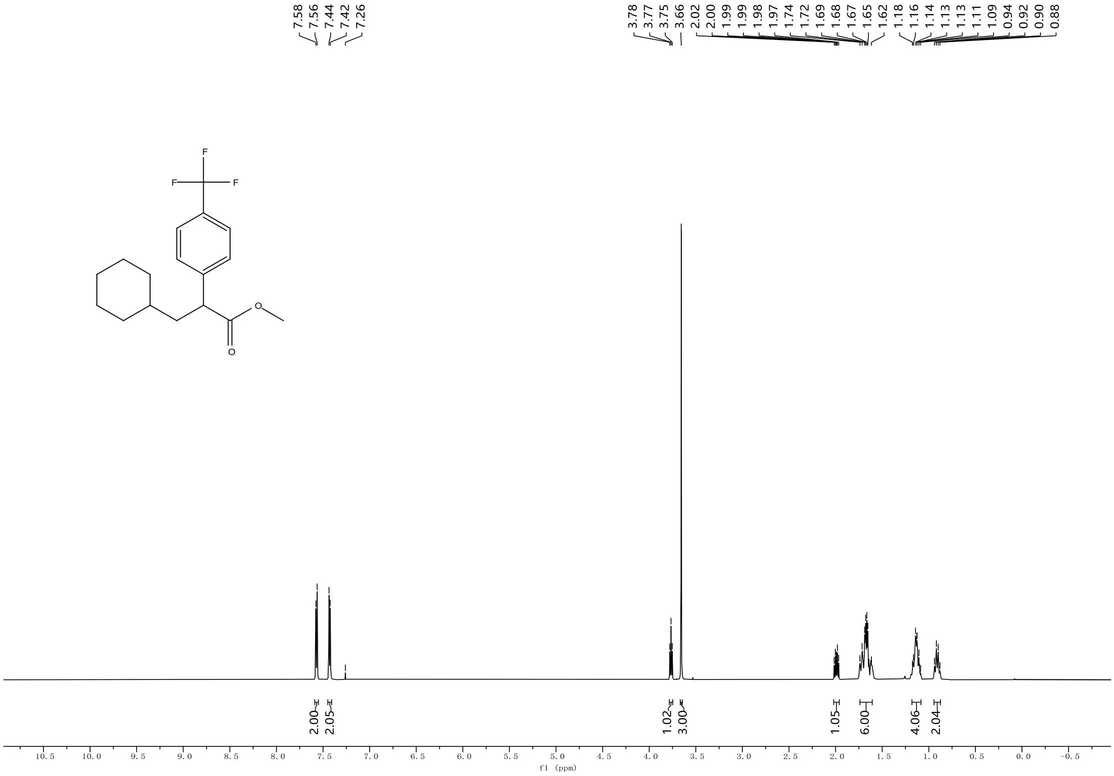

Supplementary Figure 140. ^{1}H NMR spectra of compound **41** (600 MHz, r.t., $CDCl_3$).

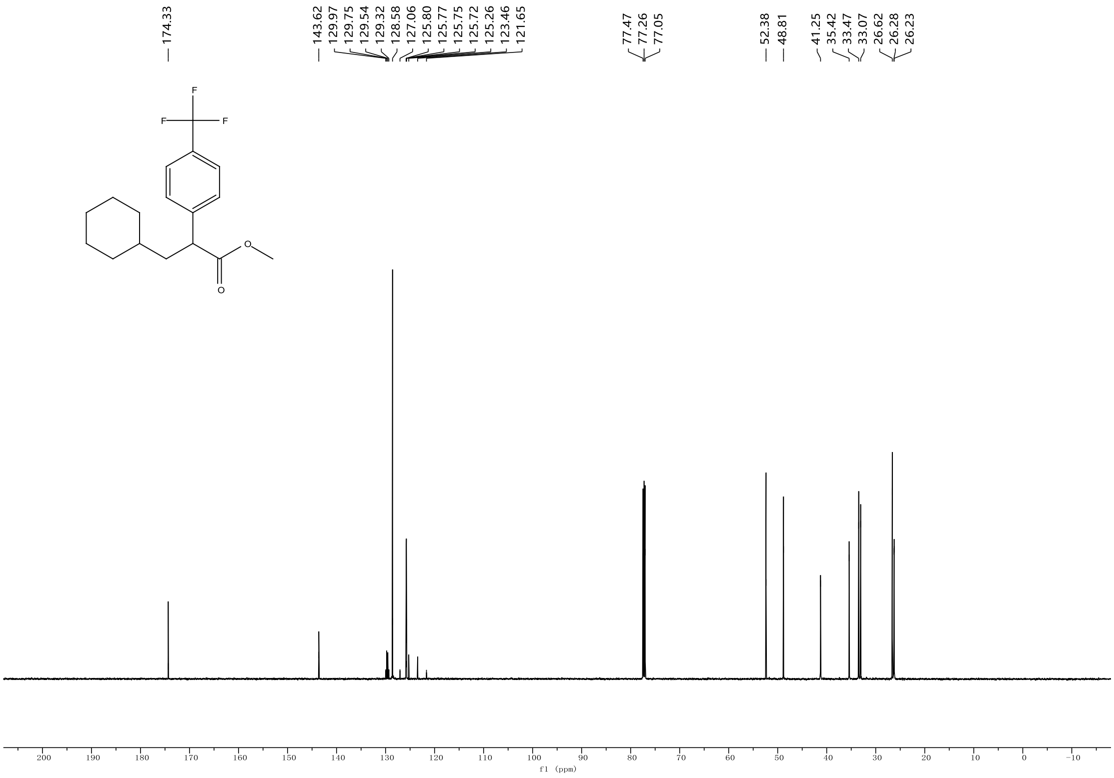

Supplementary Figure 141. ^{13}C NMR spectra of compound **41** (151 MHz, r.t., $CDCl_3$).

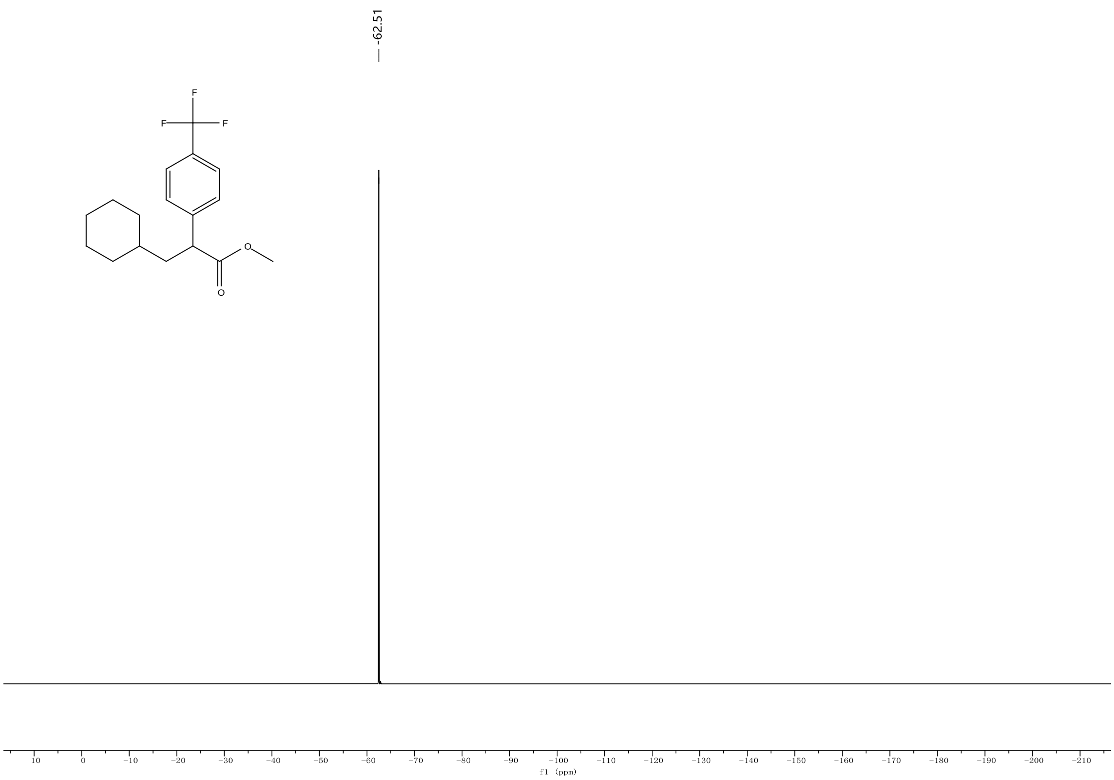

Supplementary Figure 142. ^{19}F NMR spectra of compound **41** (565 MHz, r.t., $CDCl_3$).

Compound 42

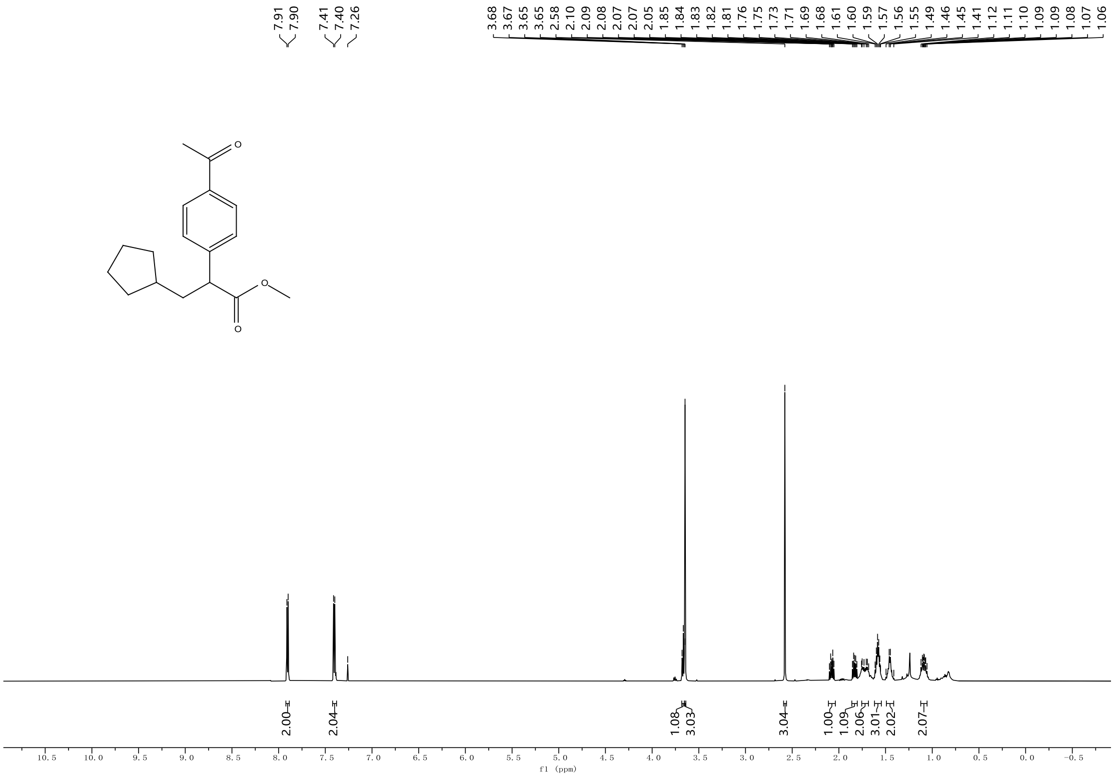

Supplementary Figure 143. ^{1}H NMR spectra of compound **42** (600 MHz, r.t., $CDCl_3$).

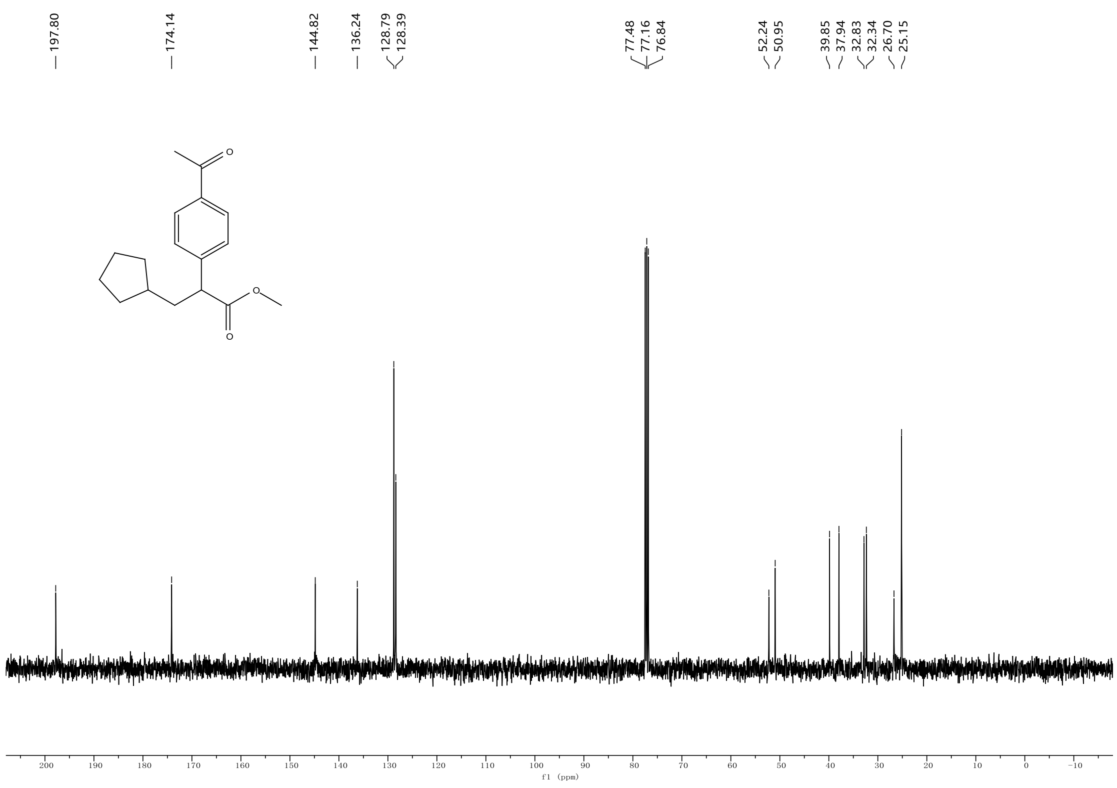

Supplementary Figure 144. ^{13}C NMR spectra of compound **42** (101 MHz, r.t., $CDCl_3$).

Compound 43

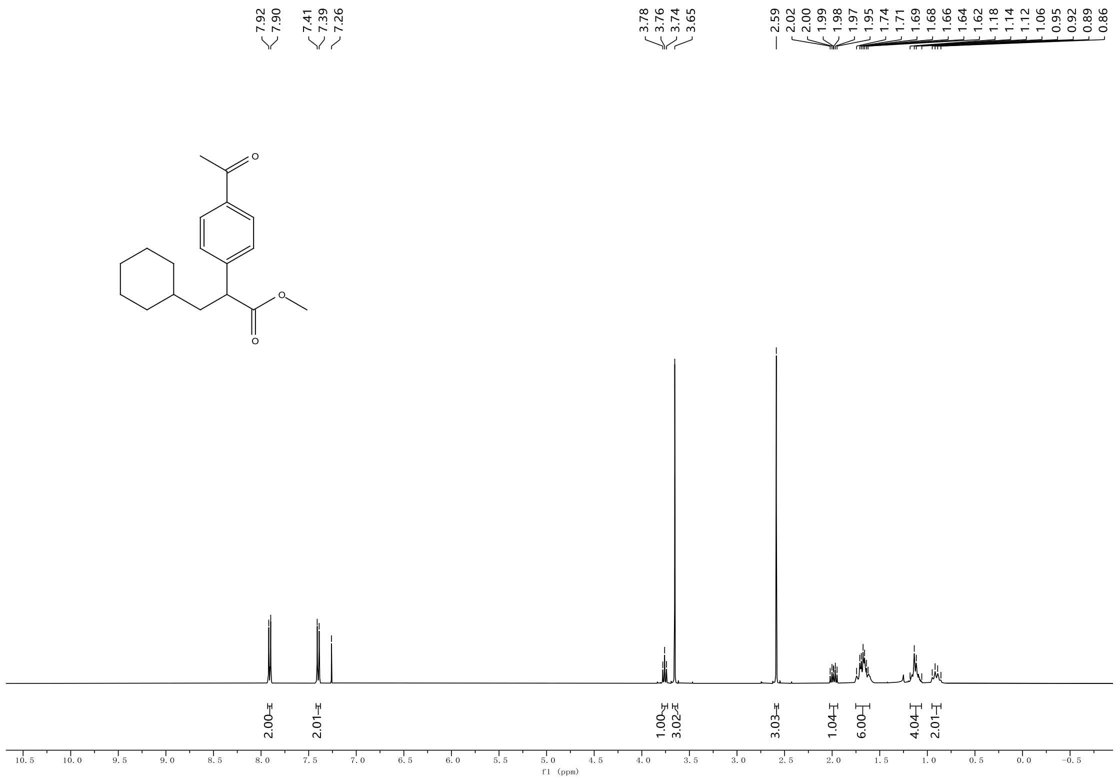

Supplementary Figure 145. ^{1}H NMR spectra of compound **43** (400 MHz, r.t., $CDCl_3$).

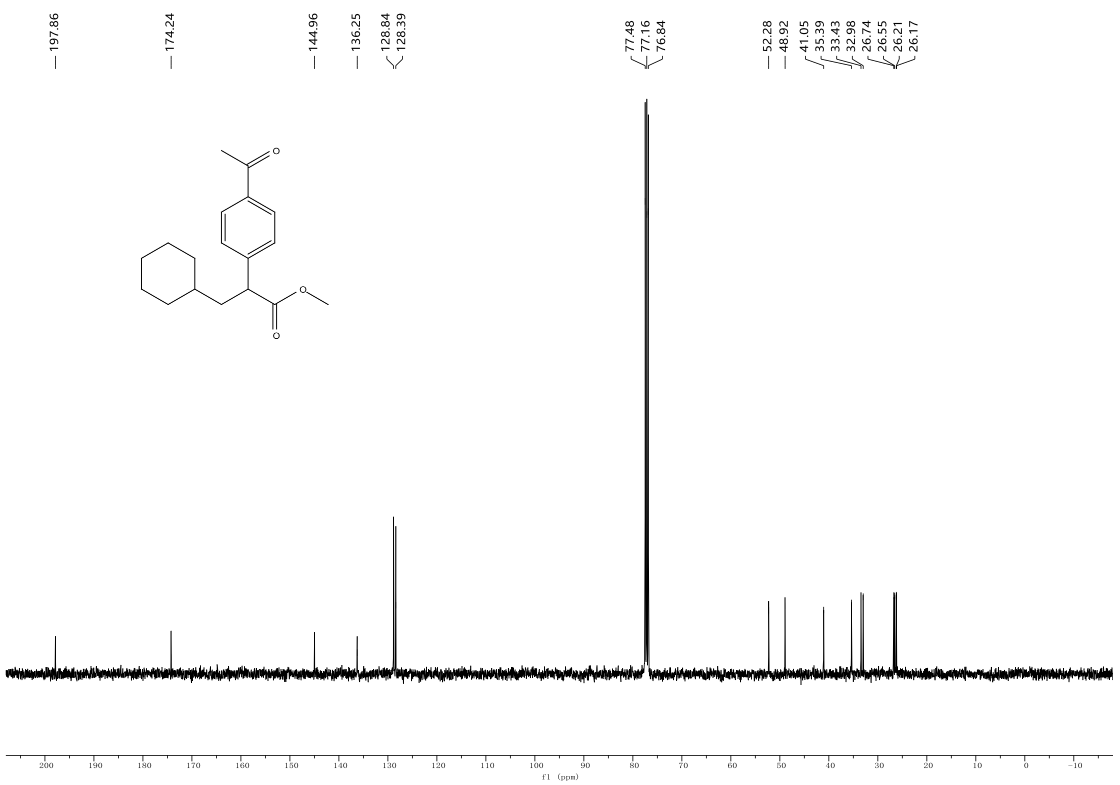

Supplementary Figure 146. ^{13}C NMR spectra of compound **43** (101 MHz, r.t., $CDCl_3$).

Compound 44

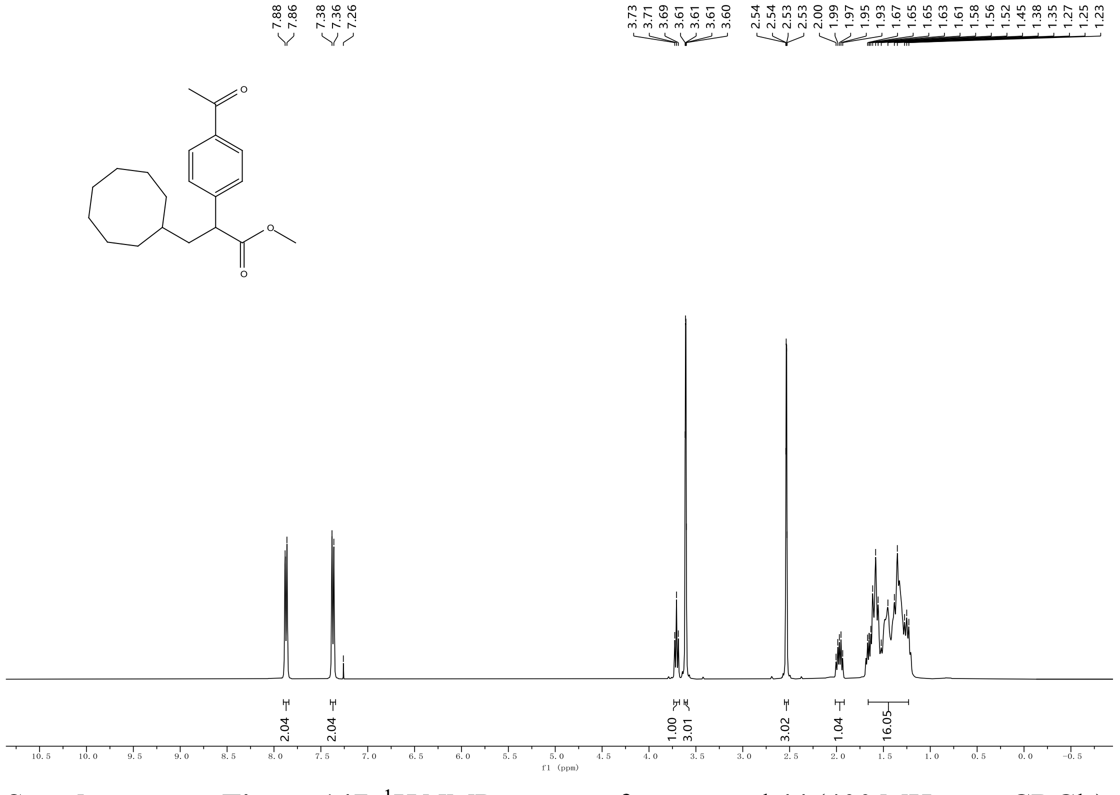

Supplementary Figure 147. ^{1}H NMR spectra of compound **44** (400 MHz, r.t., $CDCl_3$).

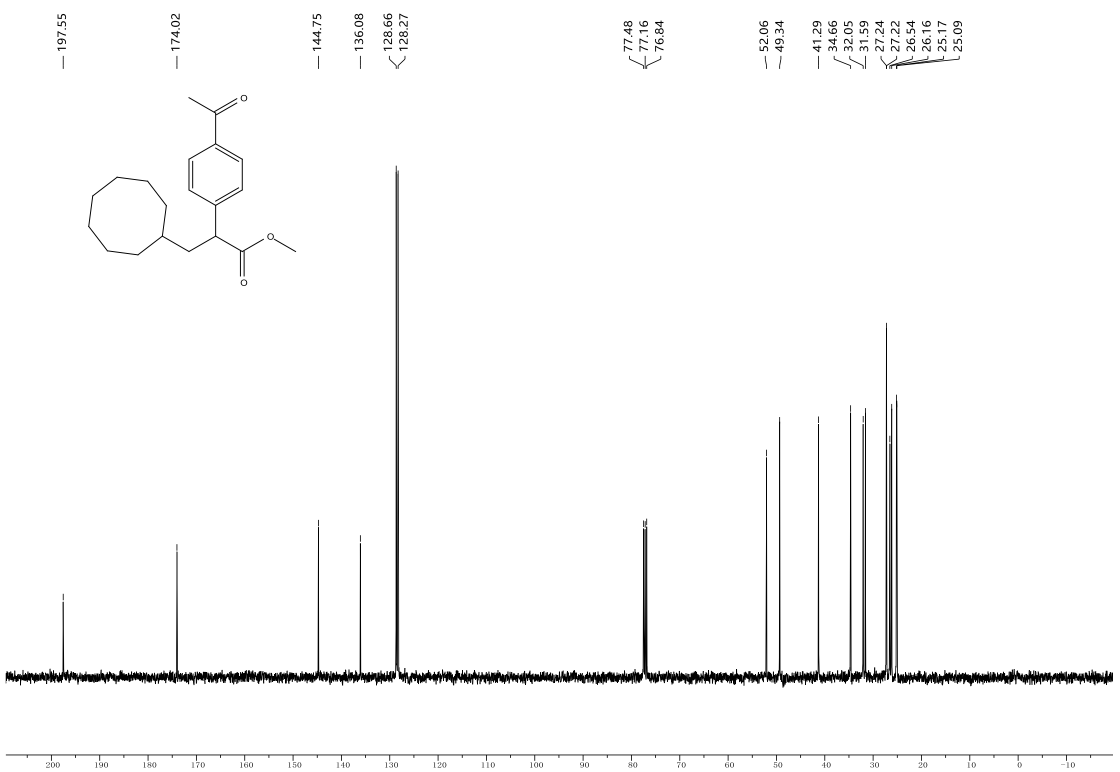

Supplementary Figure 148. ^{13}C NMR spectra of compound **44** (101 MHz, r.t., $CDCl_3$).

Compound 45

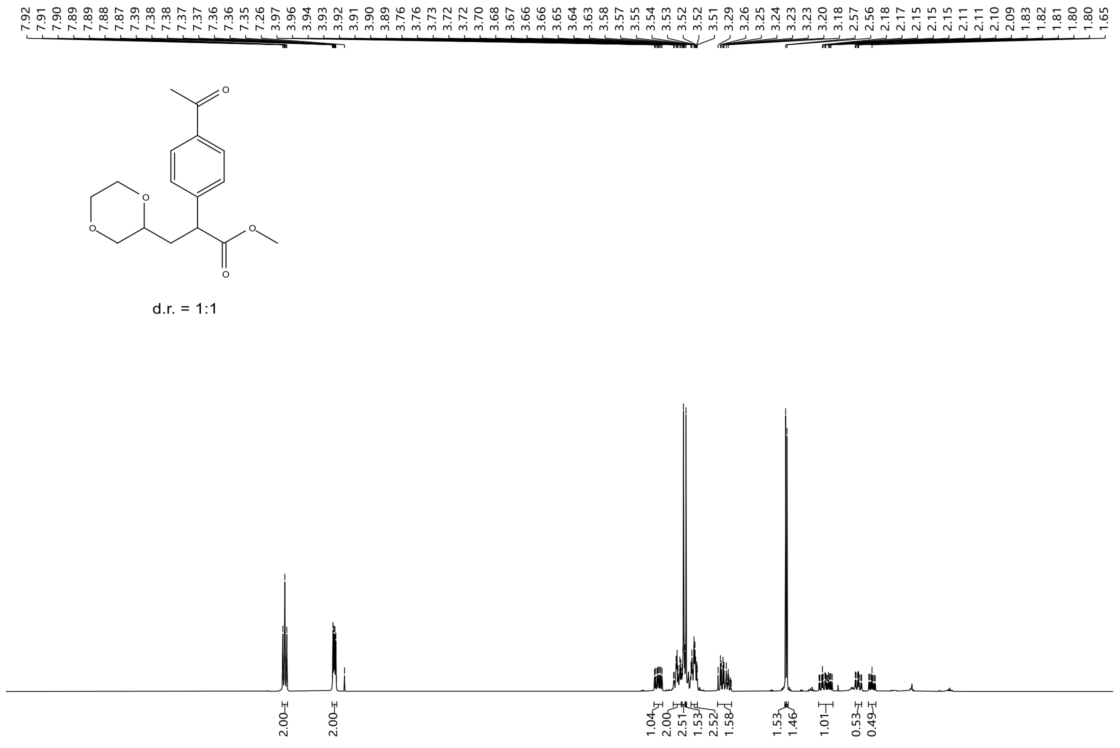

Supplementary Figure 149. ^{1}H NMR spectra of compound **45** (400 MHz, r.t., $CDCl_3$).

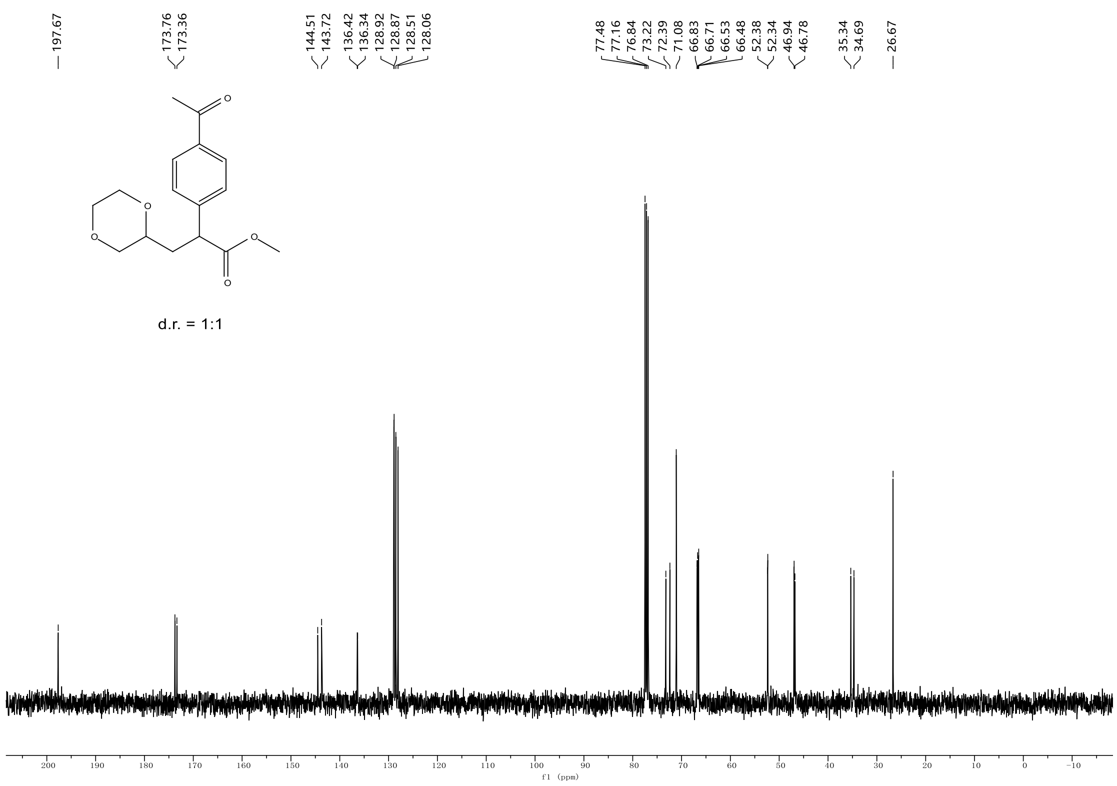

Supplementary Figure 150. ^{13}C NMR spectra of compound **45** (101 MHz, r.t., $CDCl_3$).

Compound 46

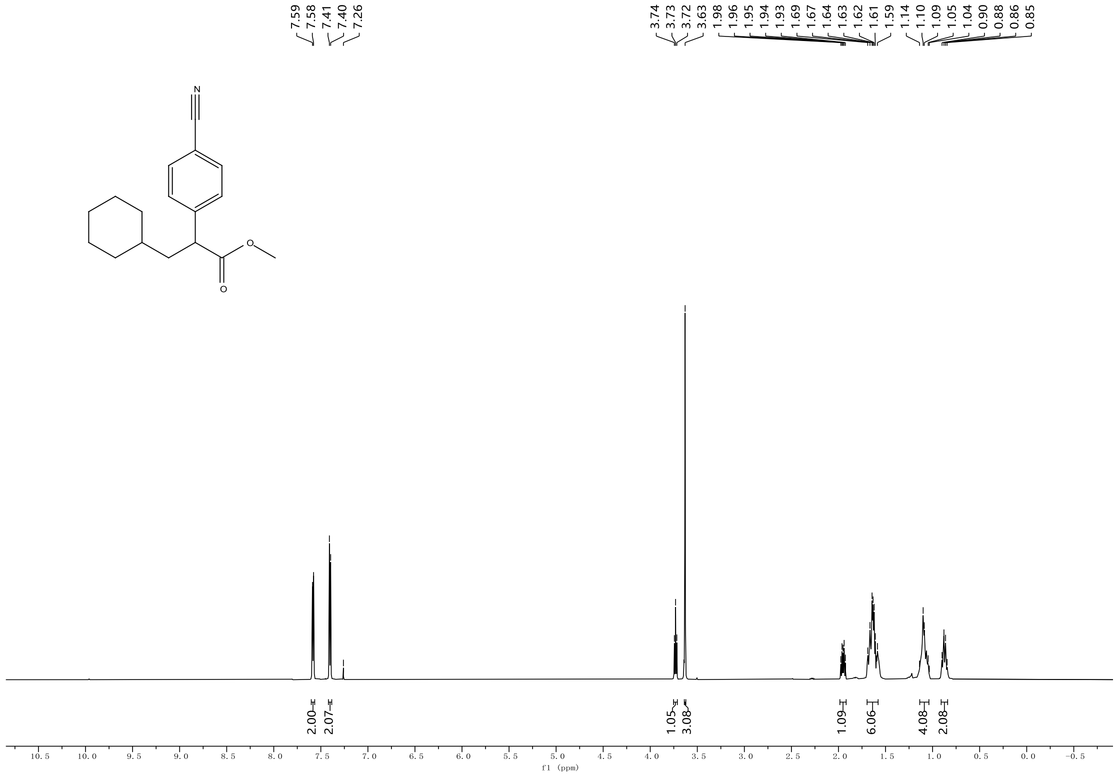

Supplementary Figure 151. ^{1}H NMR spectra of compound **46** (600 MHz, r.t., $CDCl_3$).

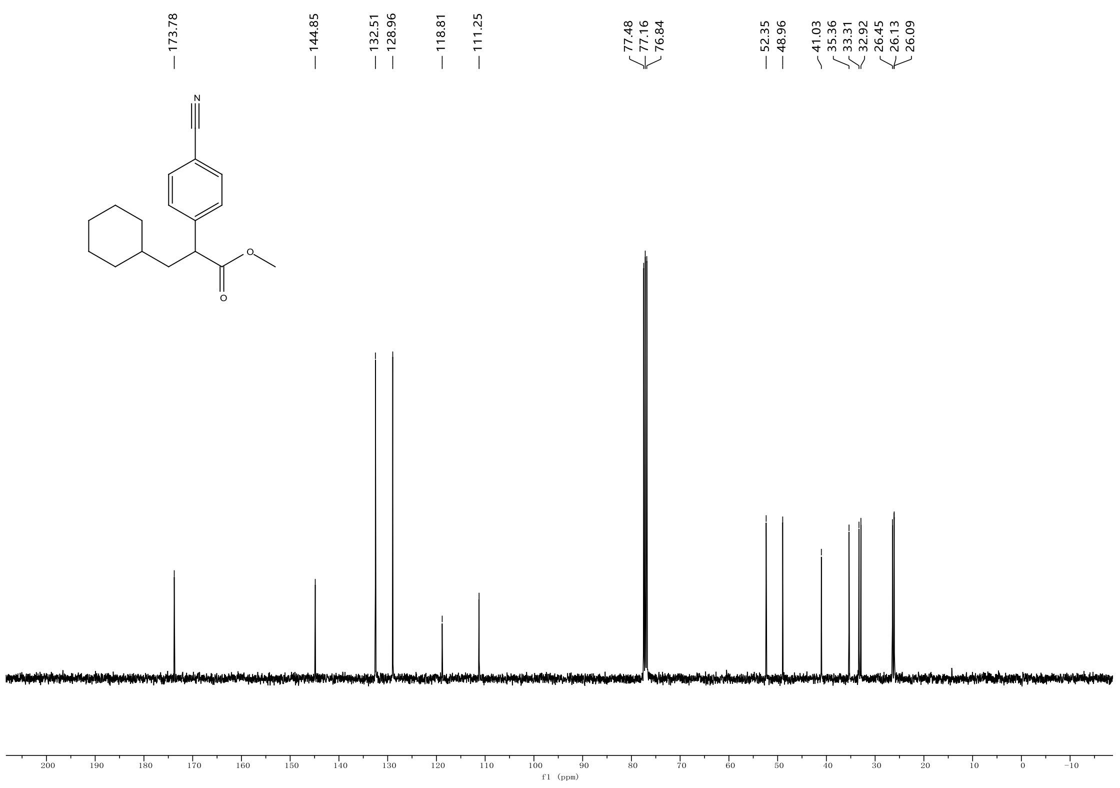

Supplementary Figure 152. ^{13}C NMR spectra of compound **46** (101 MHz, r.t., $CDCl_3$).

Compound 47

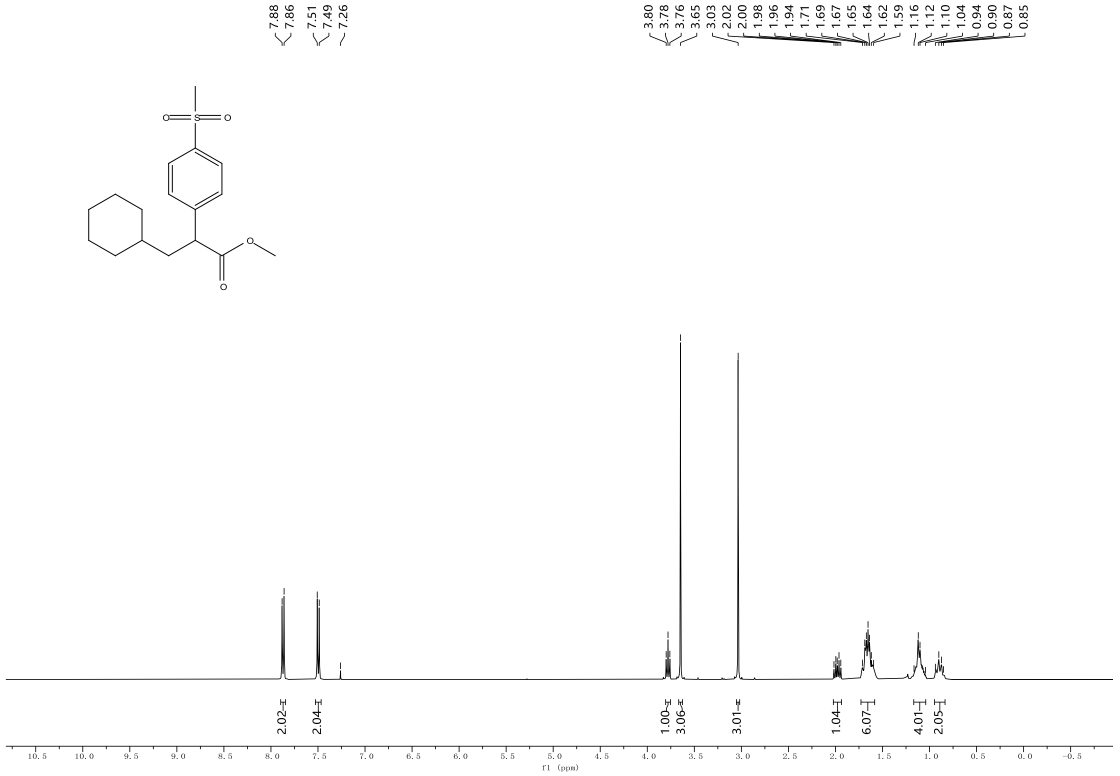

Supplementary Figure 153. ^{1}H NMR spectra of compound **47** (400 MHz, r.t., $CDCl_3$).

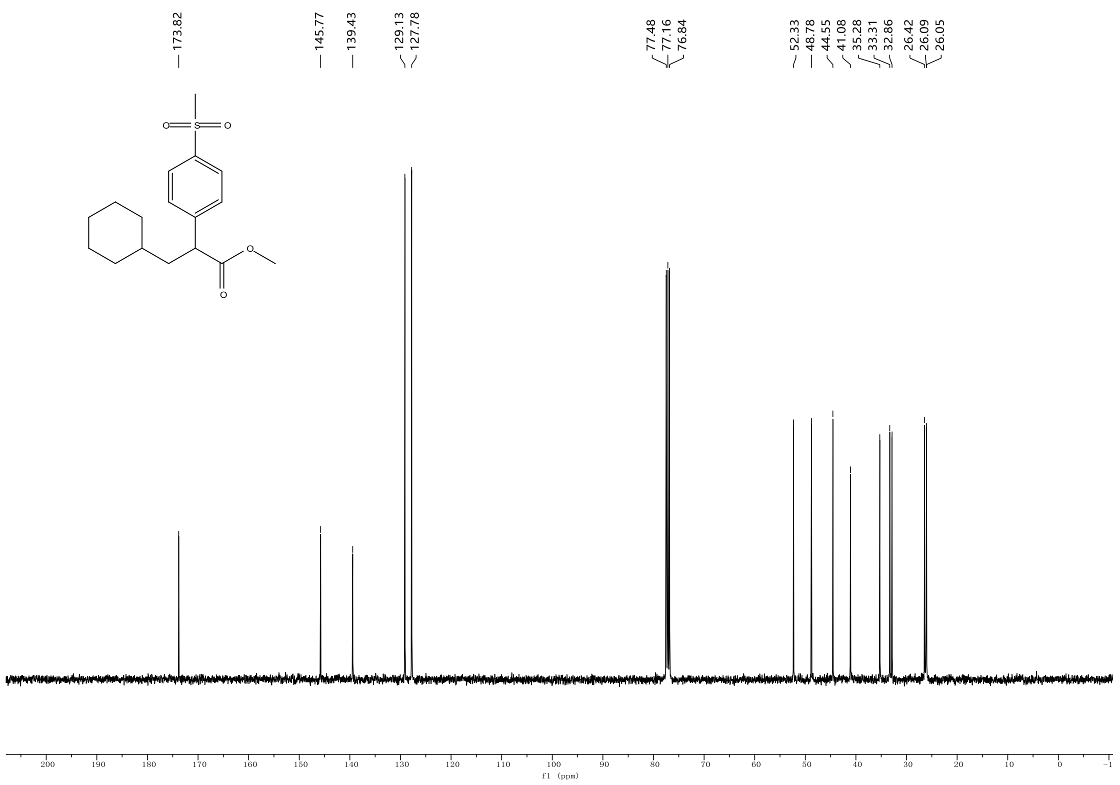

Supplementary Figure 154. ^{13}C NMR spectra of compound **47** (101 MHz, r.t., $CDCl_3$).

Compound 48

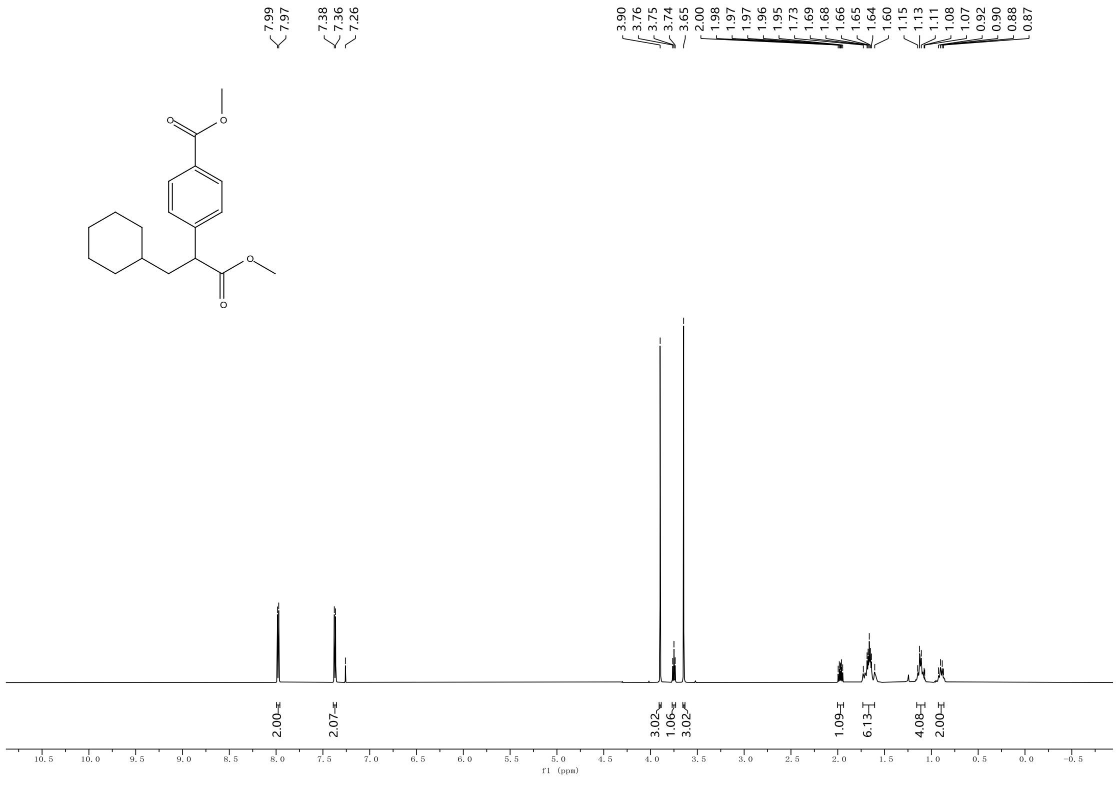

Supplementary Figure 155. ^{1}H NMR spectra of compound **48** (600 MHz, r.t., $CDCl_3$).

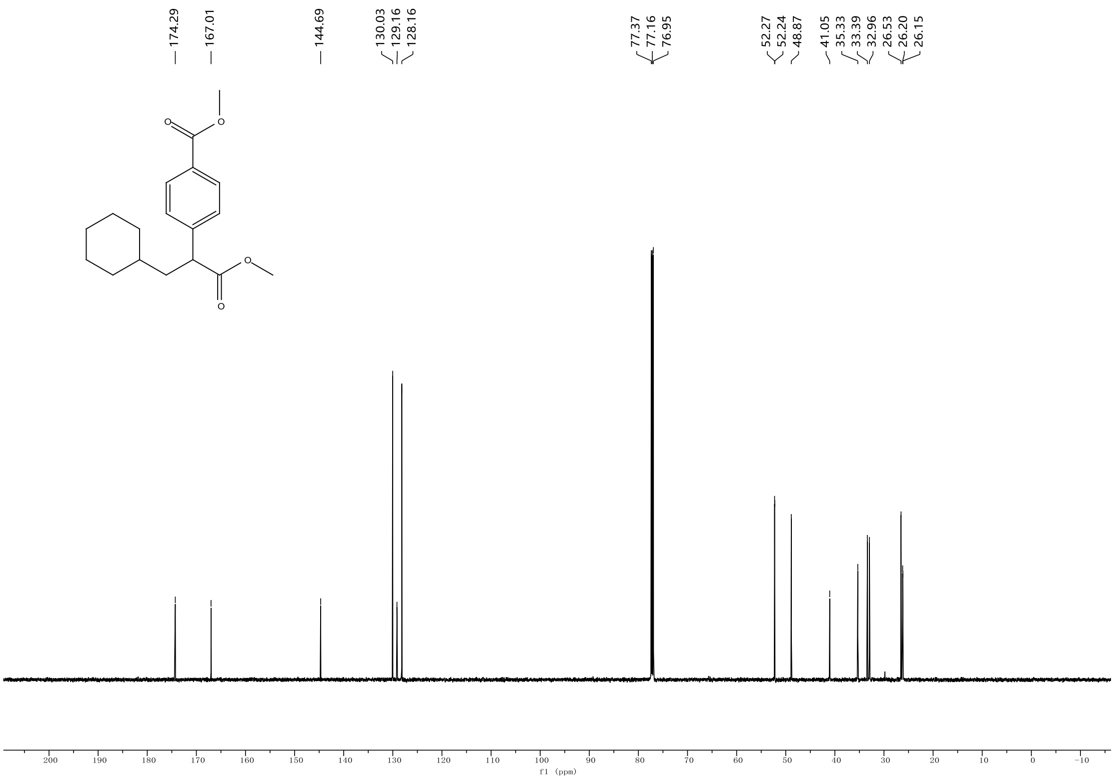

Supplementary Figure 156. ^{13}C NMR spectra of compound **48** (151 MHz, r.t., $CDCl_3$).

Compound 49

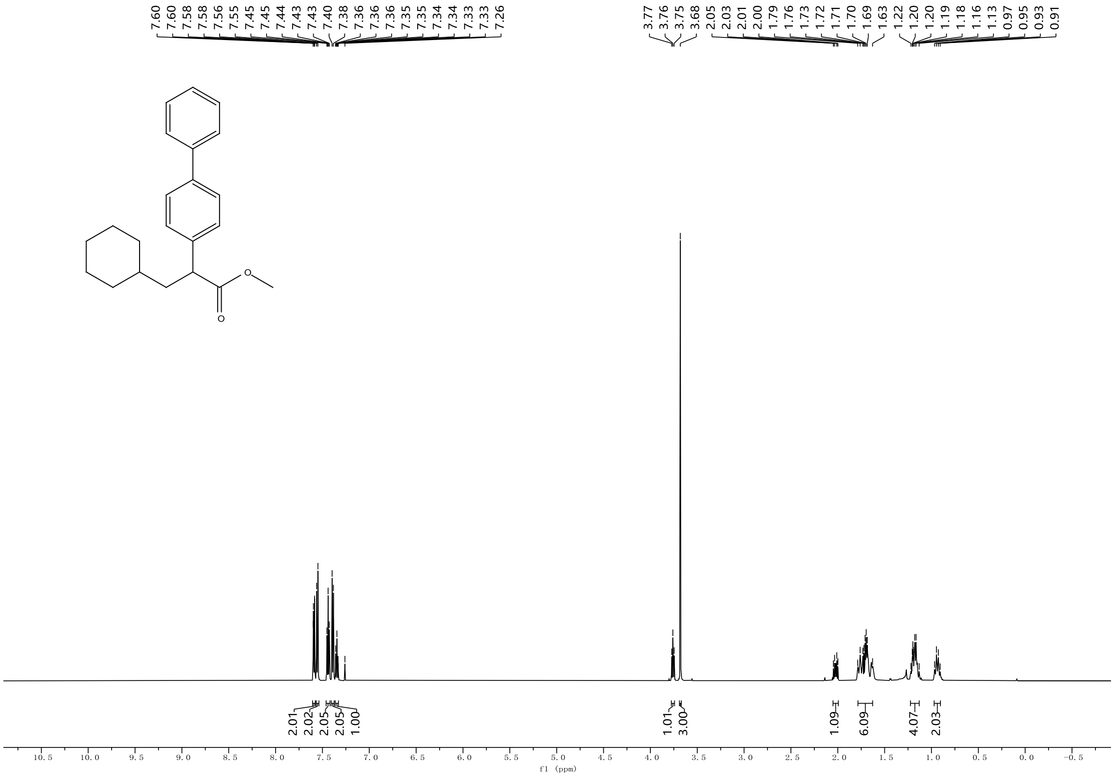

Supplementary Figure 157. ^{1}H NMR spectra of compound **49** (600 MHz, r.t., $CDCl_3$).

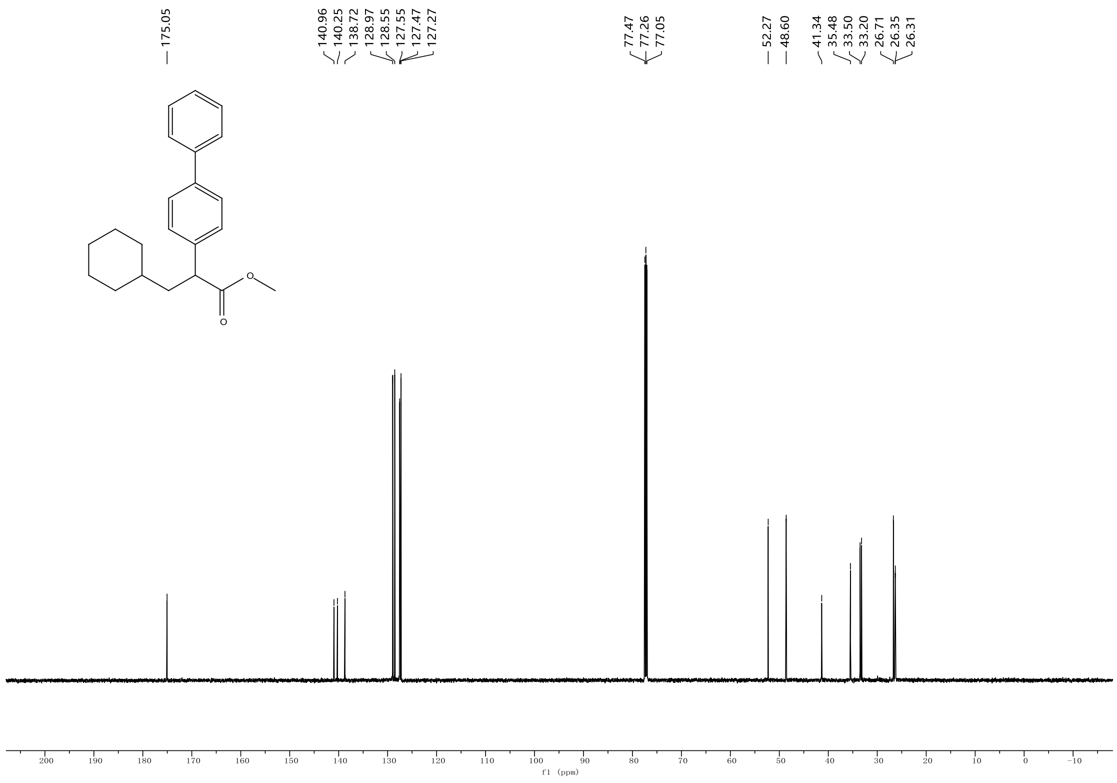

Supplementary Figure 158. ^{13}C NMR spectra of compound **49** (151 MHz, r.t., $CDCl_3$).

Compound 50

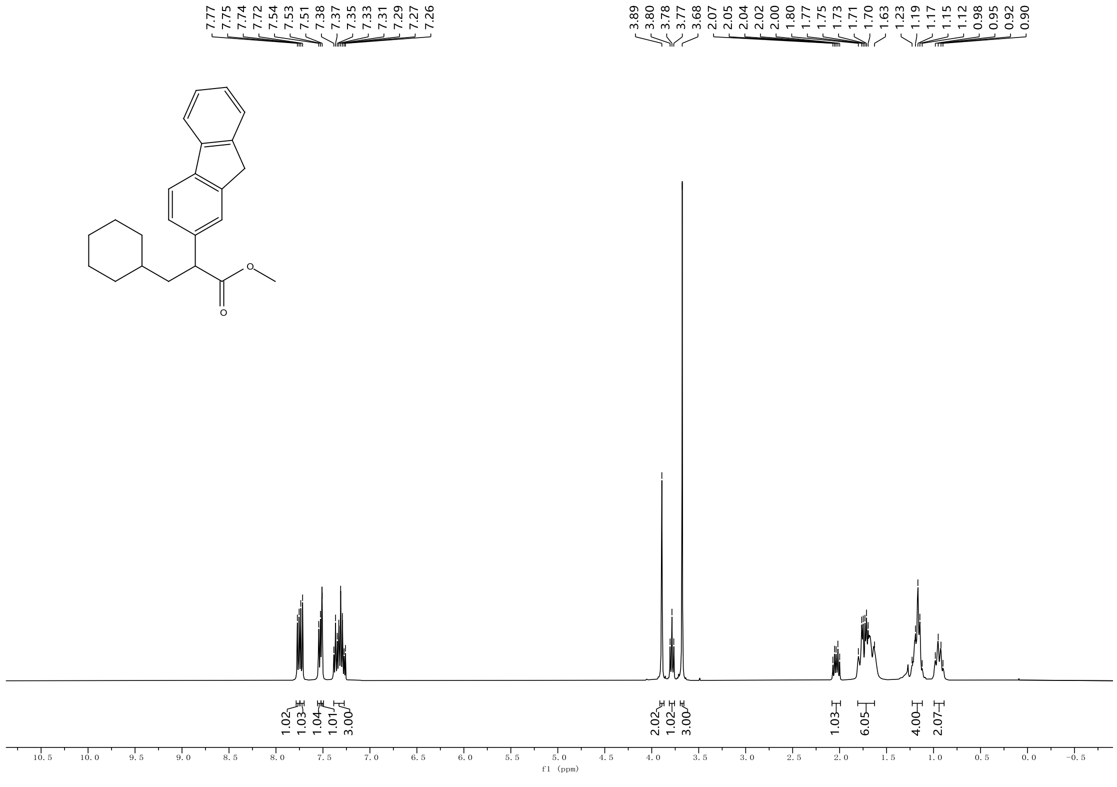

Supplementary Figure 159. ^{1}H NMR spectra of compound **50** (400 MHz, r.t., $CDCl_3$).

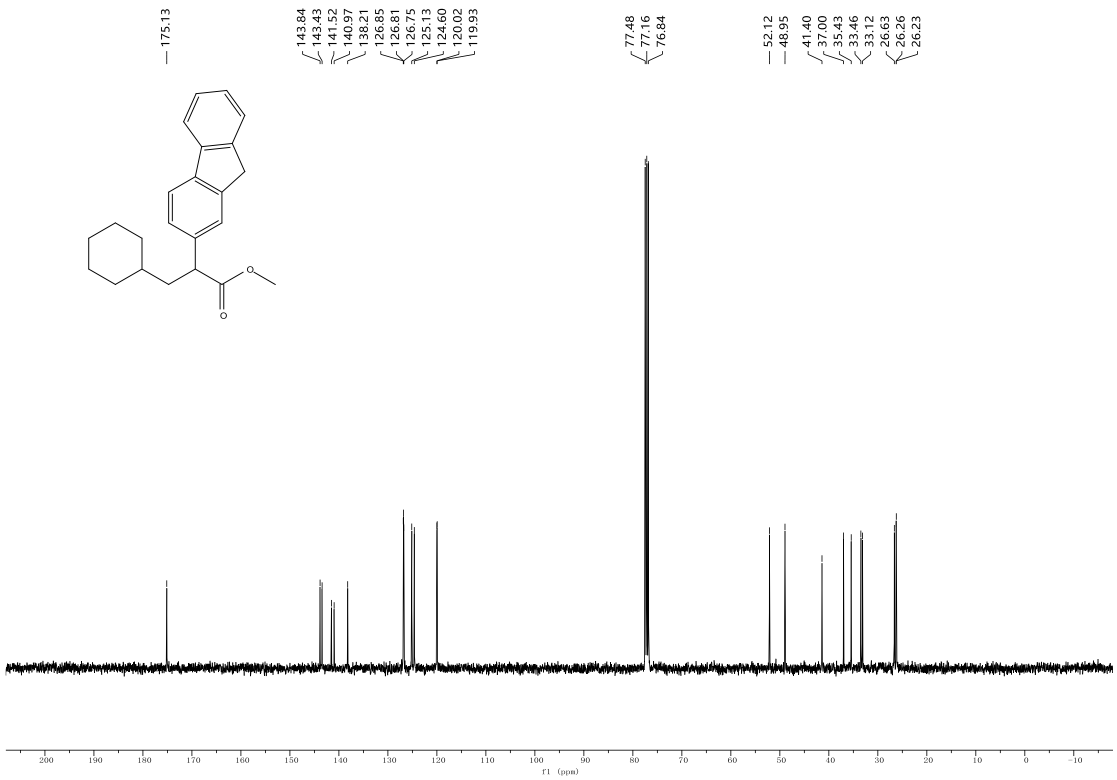

Supplementary Figure 160. ^{13}C NMR spectra of compound **50** (101 MHz, r.t., $CDCl_3$).

Compound 51

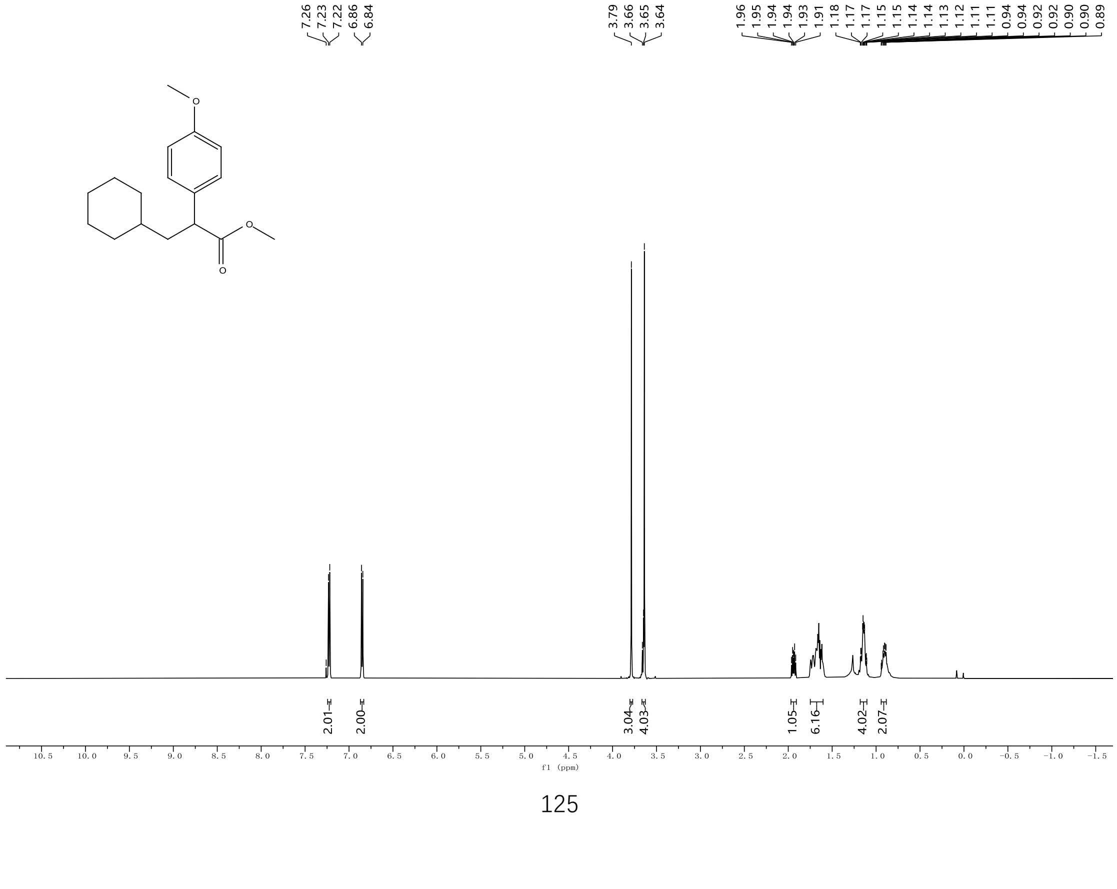

Supplementary Figure 161. ^{1}H NMR spectra of compound **51** (600 MHz, r.t., $CDCl_3$).

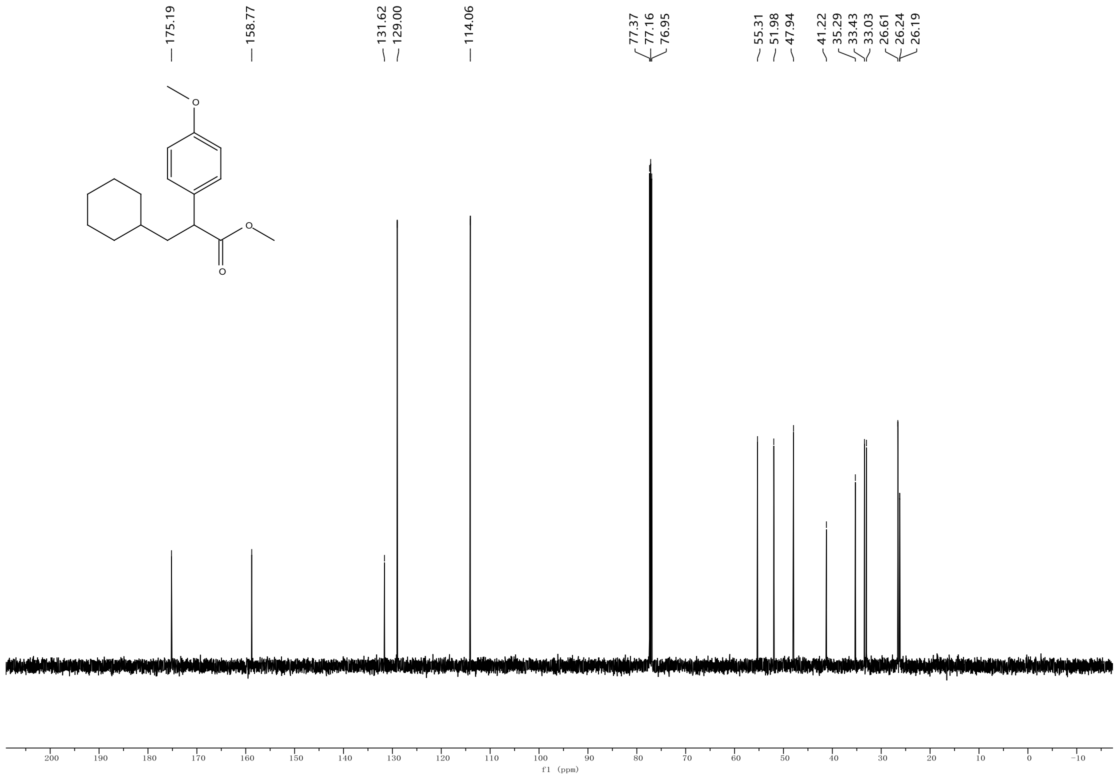

Supplementary Figure 162. ^{13}C NMR spectra of compound **51** (151 MHz, r.t., $CDCl_3$).

Compound 52

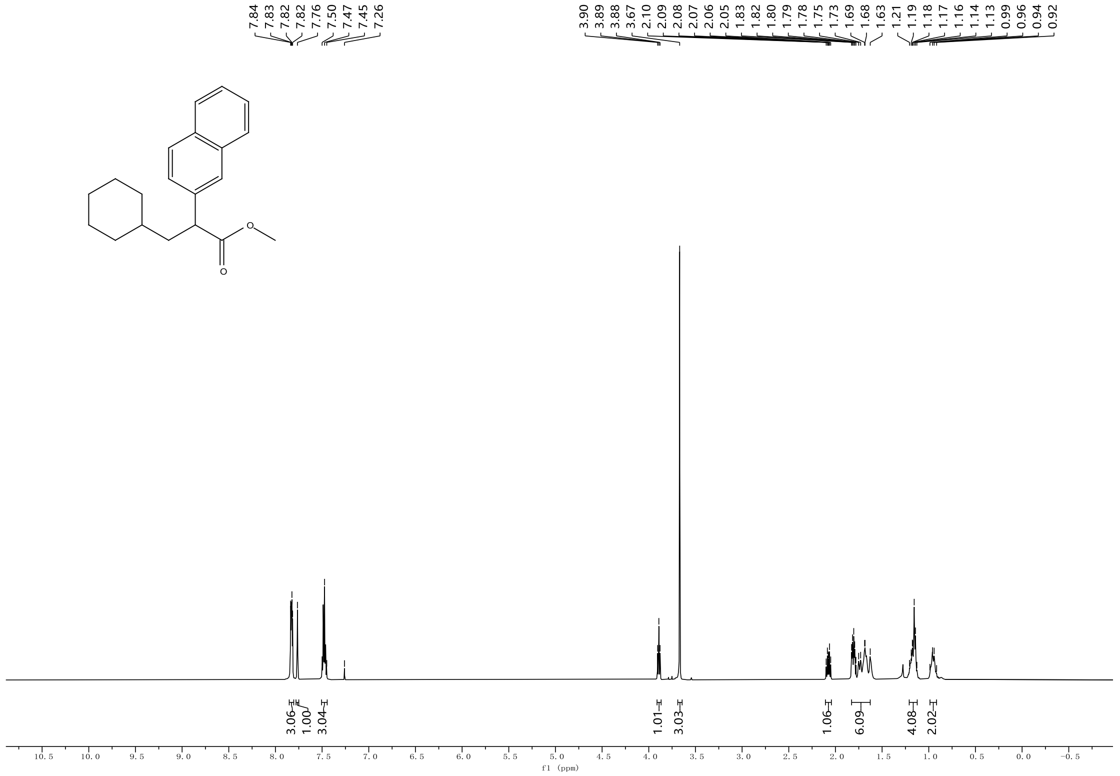

Supplementary Figure 163. ^{1}H NMR spectra of compound **52** (600 MHz, r.t., $CDCl_3$).

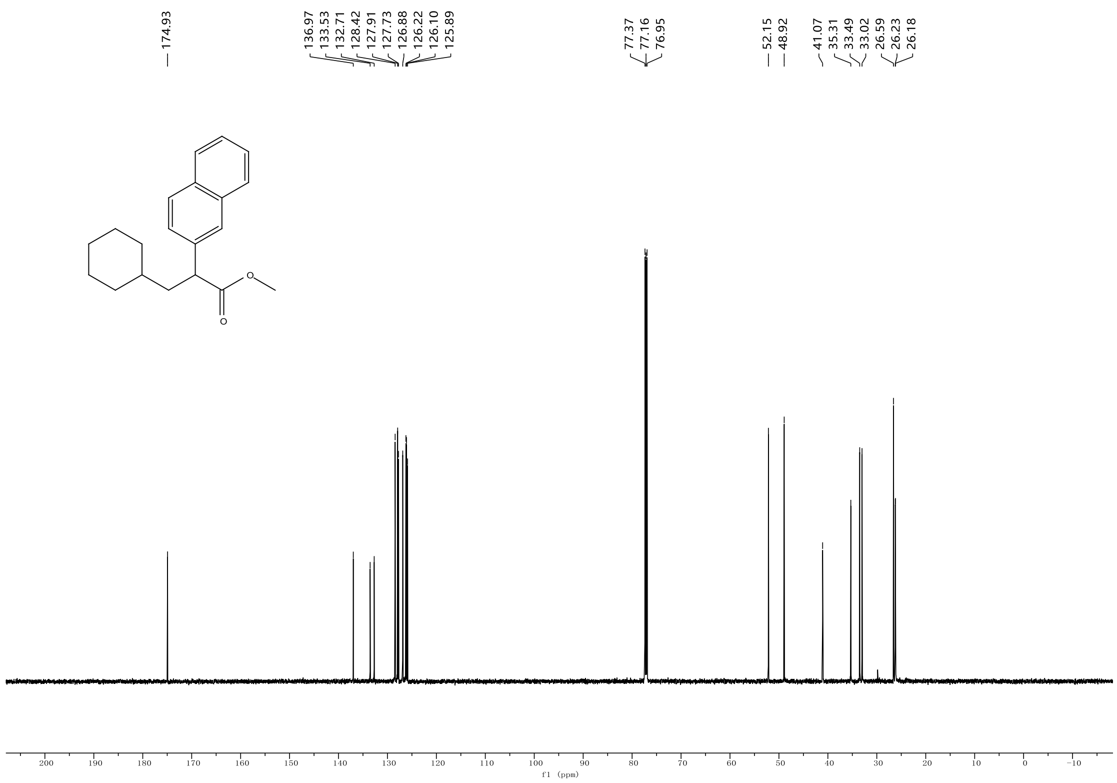

Supplementary Figure 164. ^{13}C NMR spectra of compound **52** (151 MHz, r.t., $CDCl_3$).

Compound 53

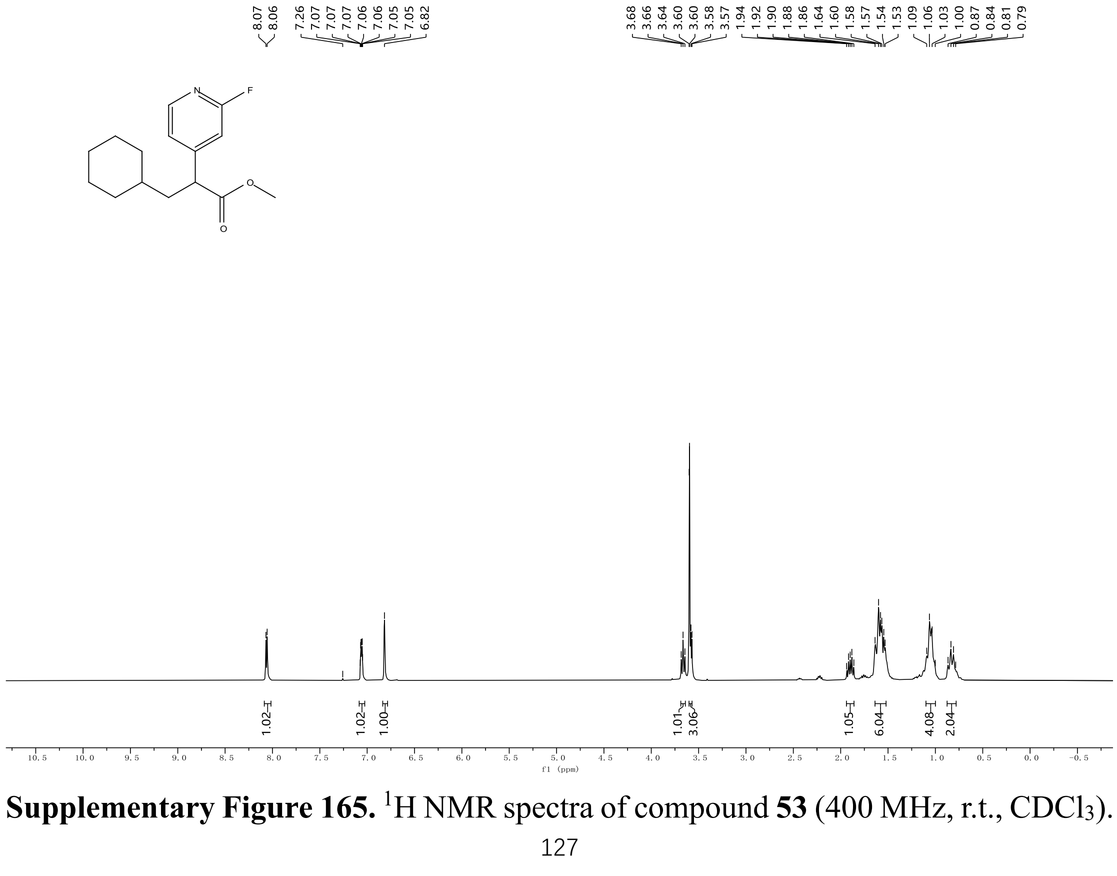

Supplementary Figure 165. ^{1}H NMR spectra of compound **53** (400 MHz, r.t., $CDCl_3$).

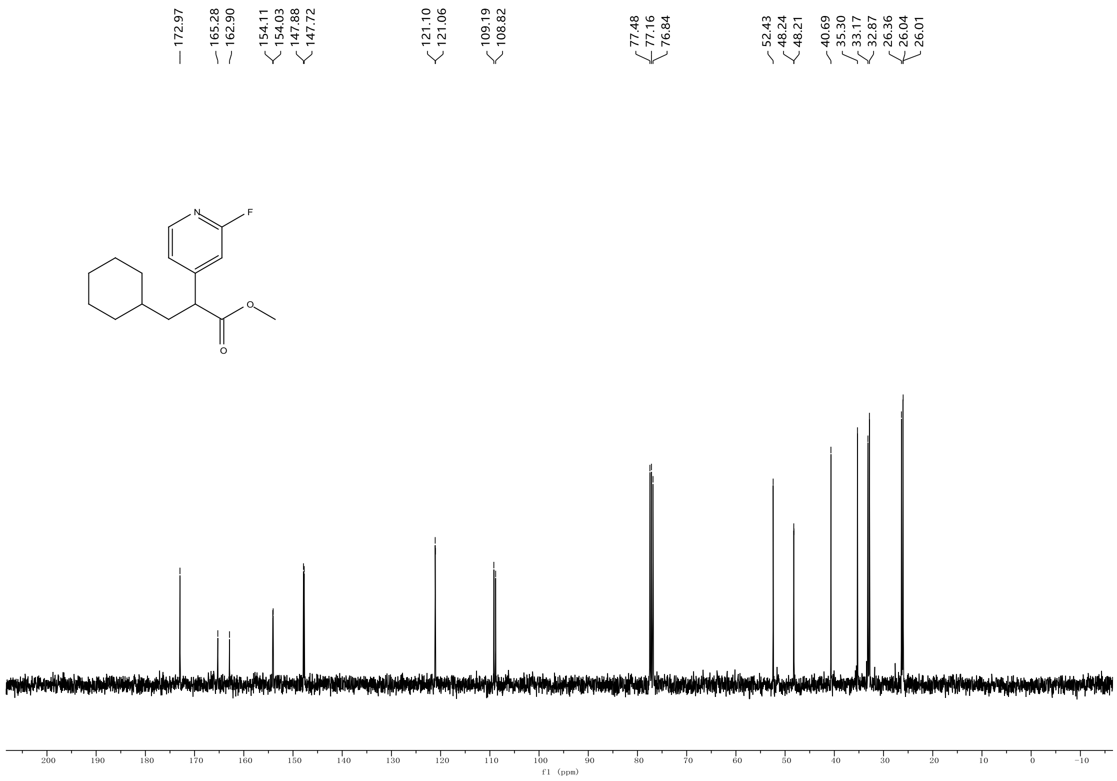

Supplementary Figure 166. ^{13}C NMR spectra of compound **53** (101 MHz, r.t., $CDCl_3$).

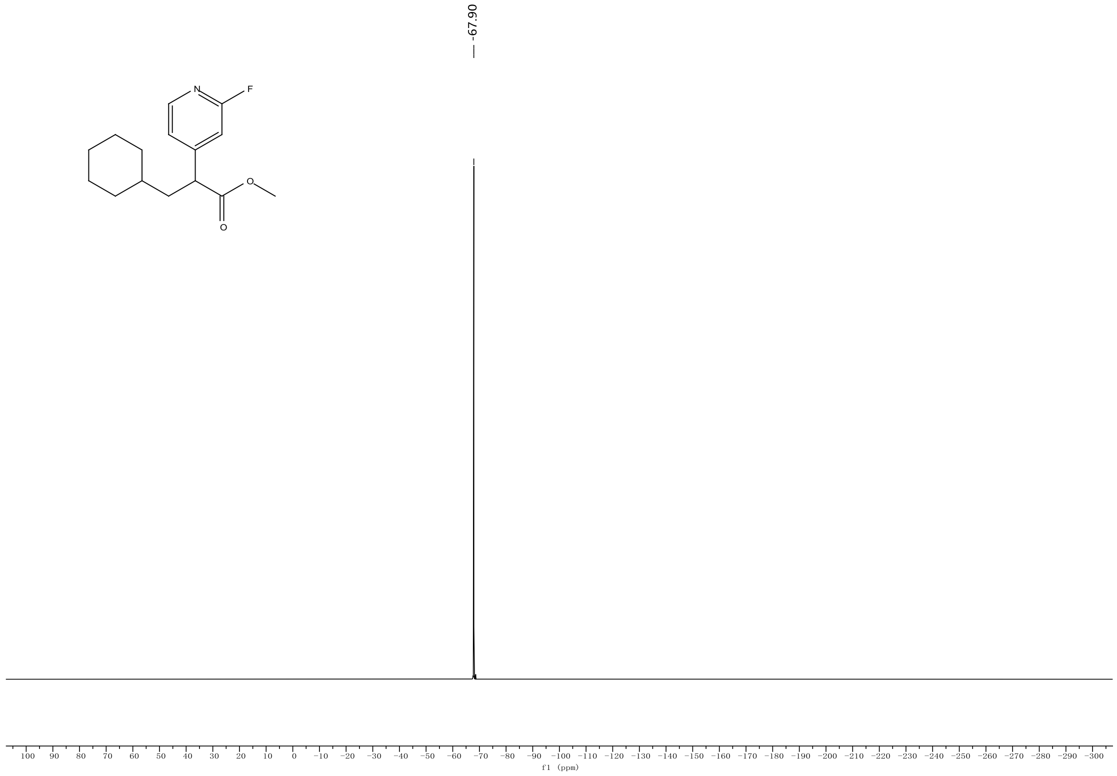

Supplementary Figure 167. ^{19}F NMR spectra of compound **53** (565 MHz, r.t., $CDCl_3$).

Compound 54

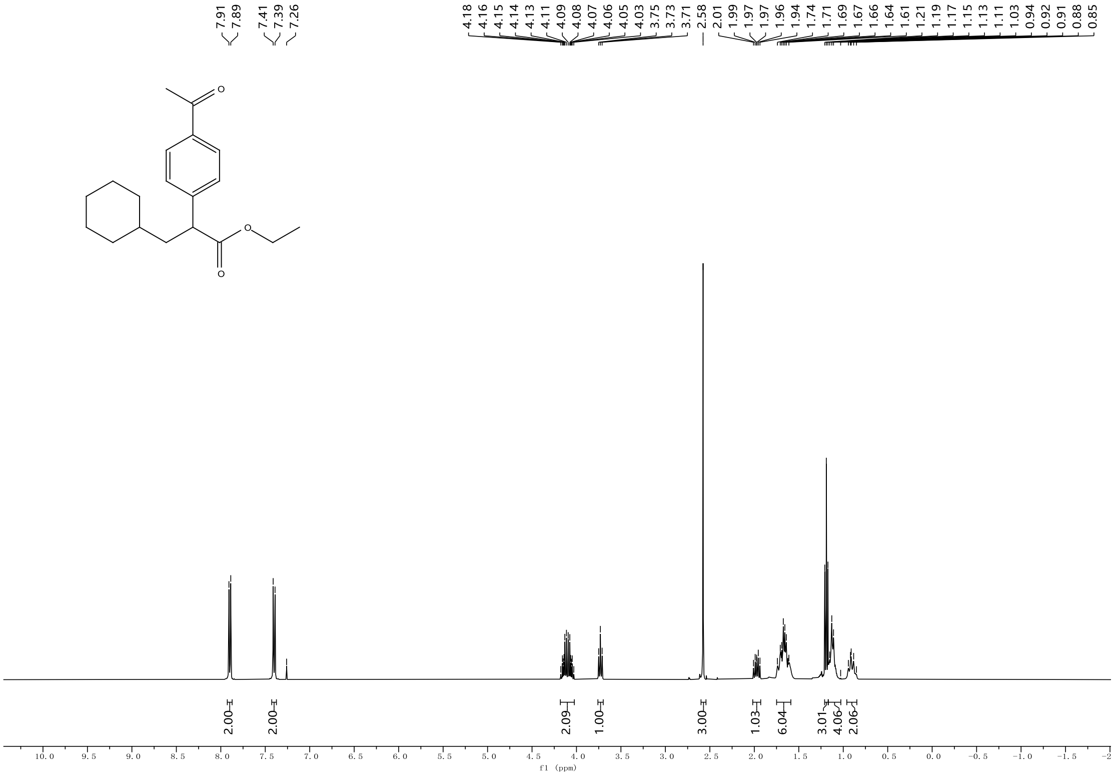

Supplementary Figure 168. ^{1}H NMR spectra of compound **54** (400 MHz, r.t., $CDCl_3$).

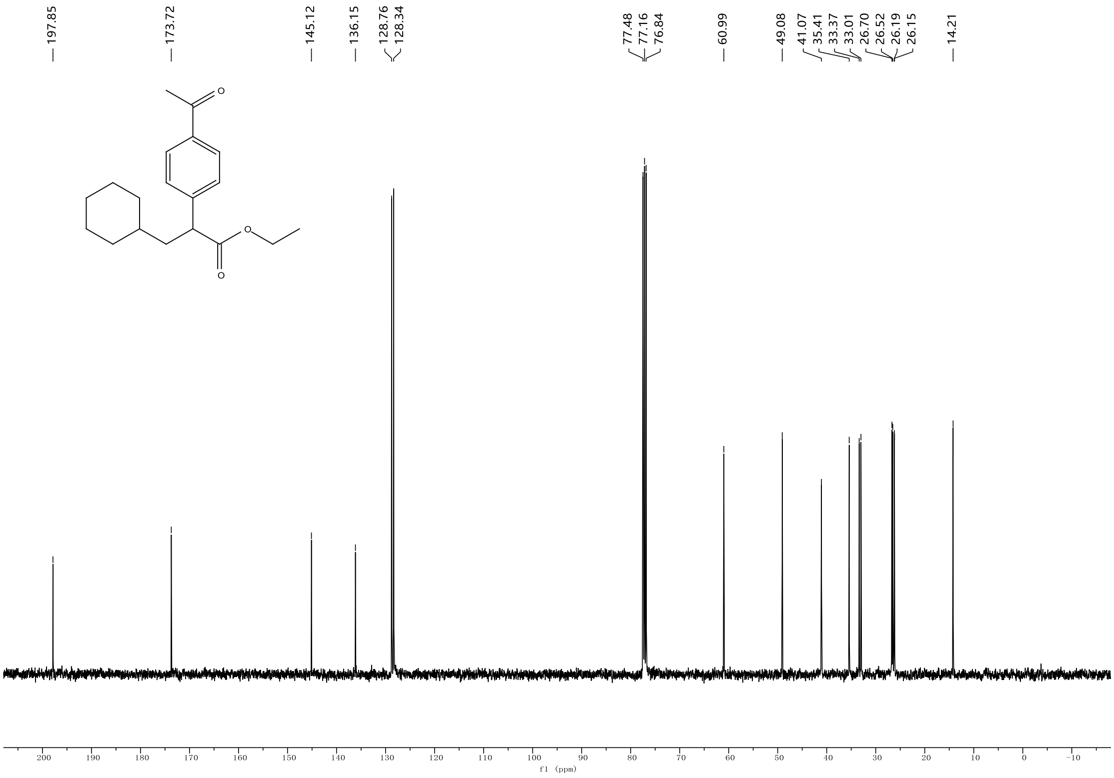

Supplementary Figure 169. ^{13}C NMR spectra of compound **54** (101 MHz, r.t., $CDCl_3$).

Compound 55

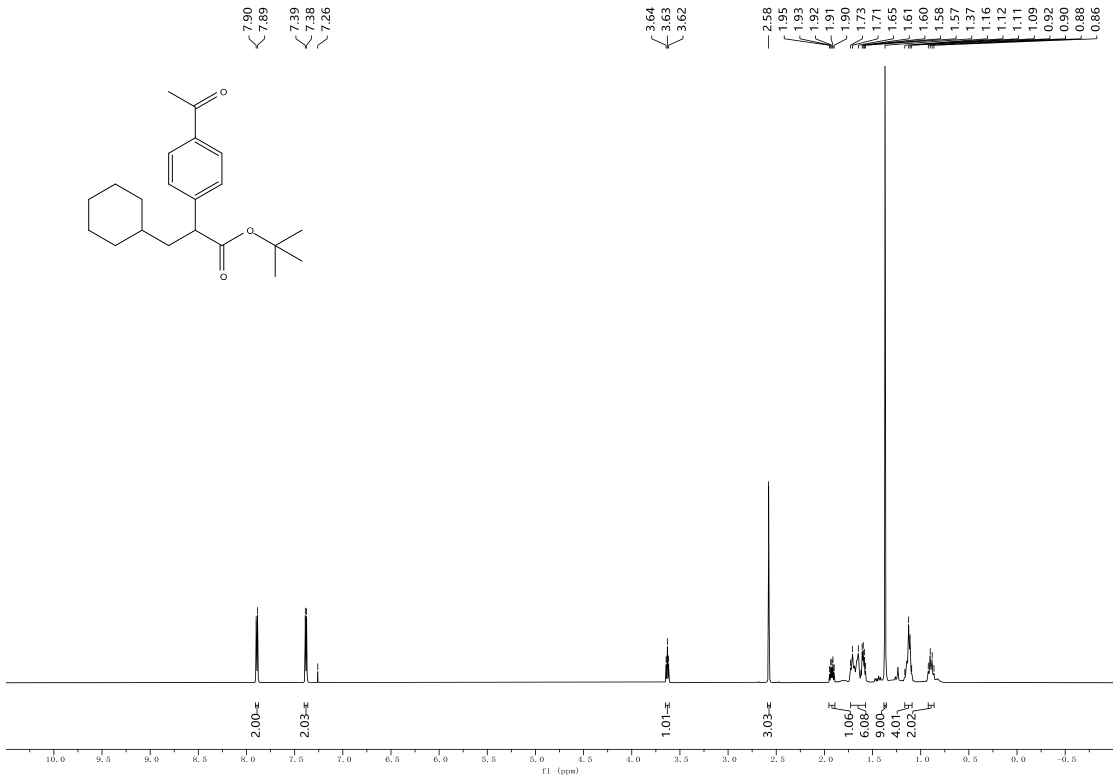

Supplementary Figure 170. ^{1}H NMR spectra of compound **55** (600 MHz, r.t., $CDCl_3$).

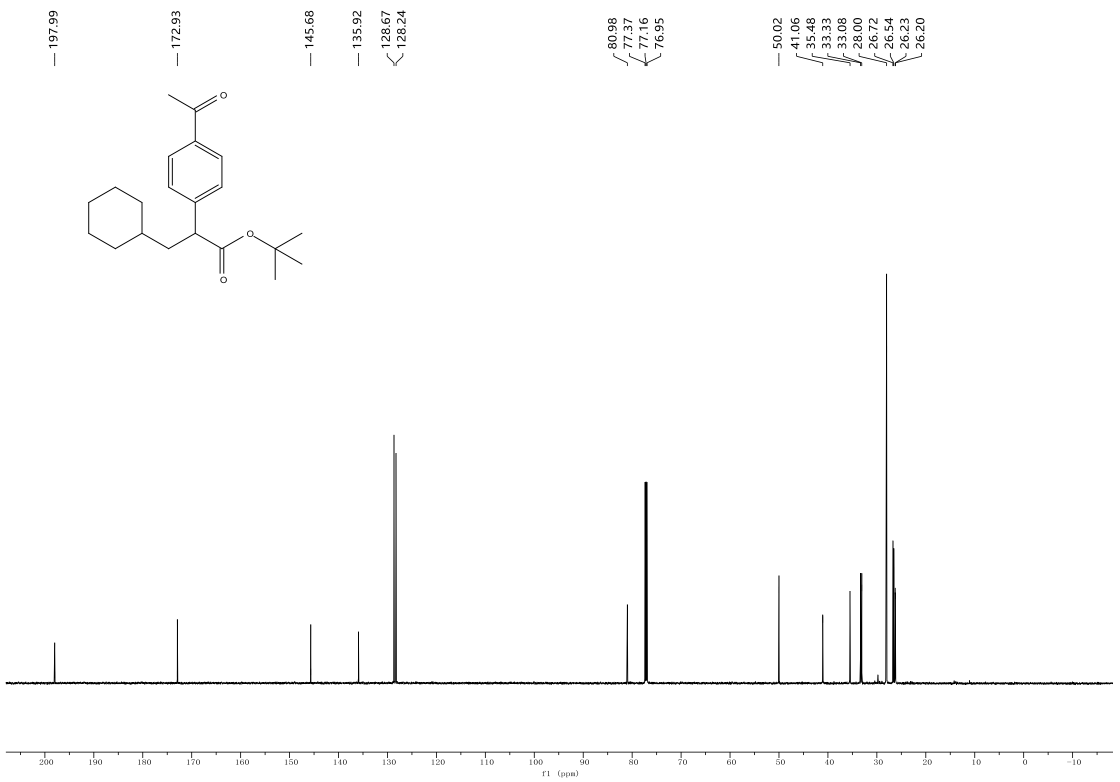

Supplementary Figure 171. ^{13}C NMR spectra of compound **55** (151 MHz, r.t., $CDCl_3$).

Compound 56

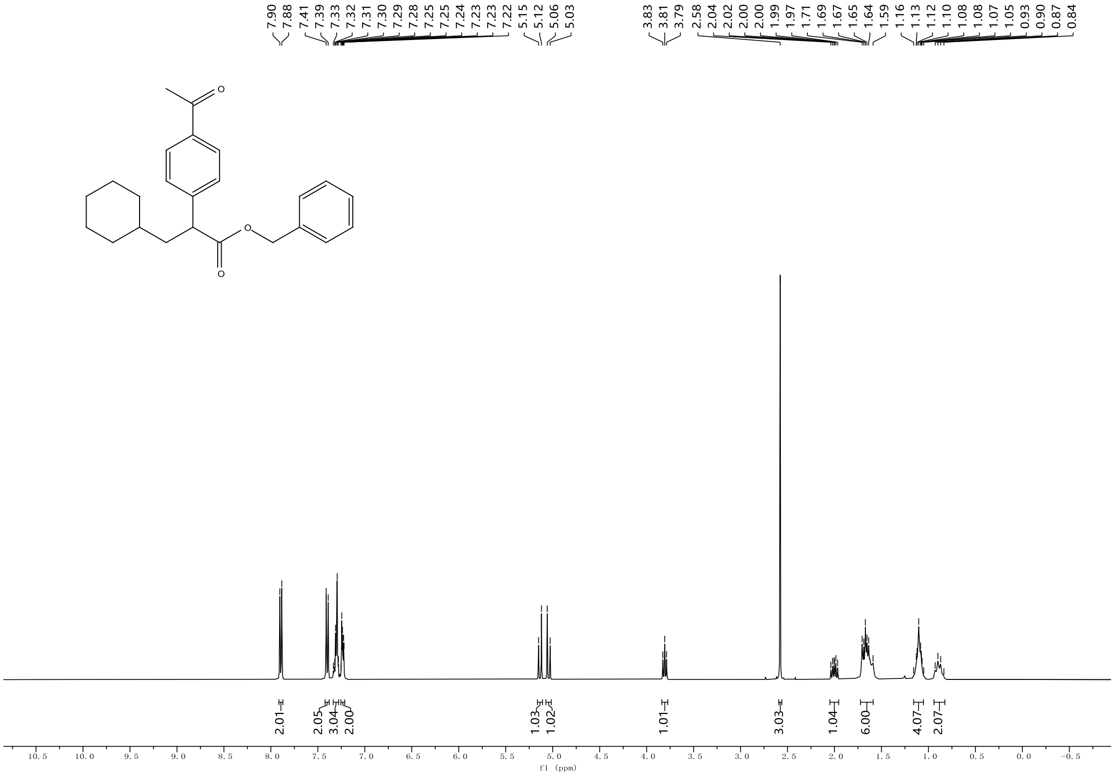

Supplementary Figure 172. ^{1}H NMR spectra of compound **56** (400 MHz, r.t., $CDCl_3$).

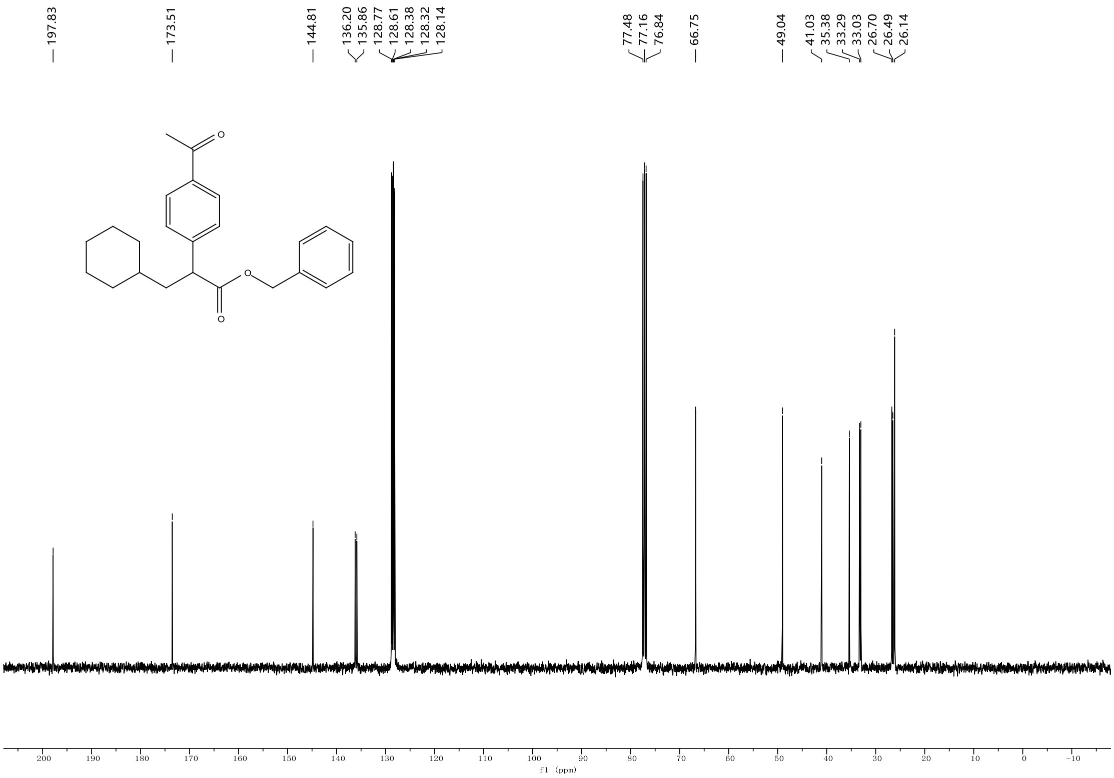

Supplementary Figure 173. ^{13}C NMR spectra of compound **56** (101 MHz, r.t., $CDCl_3$).

Compound 57

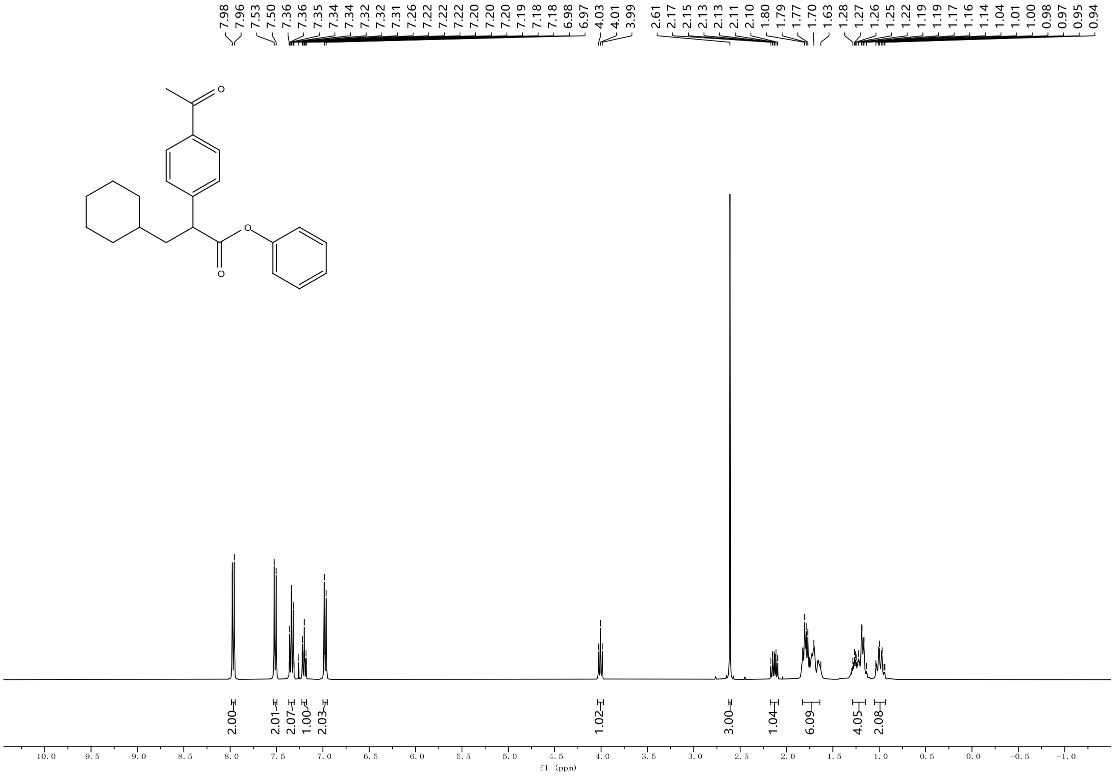

Supplementary Figure 174. ^{1}H NMR spectra of compound **57** (400 MHz, r.t., $CDCl_3$).

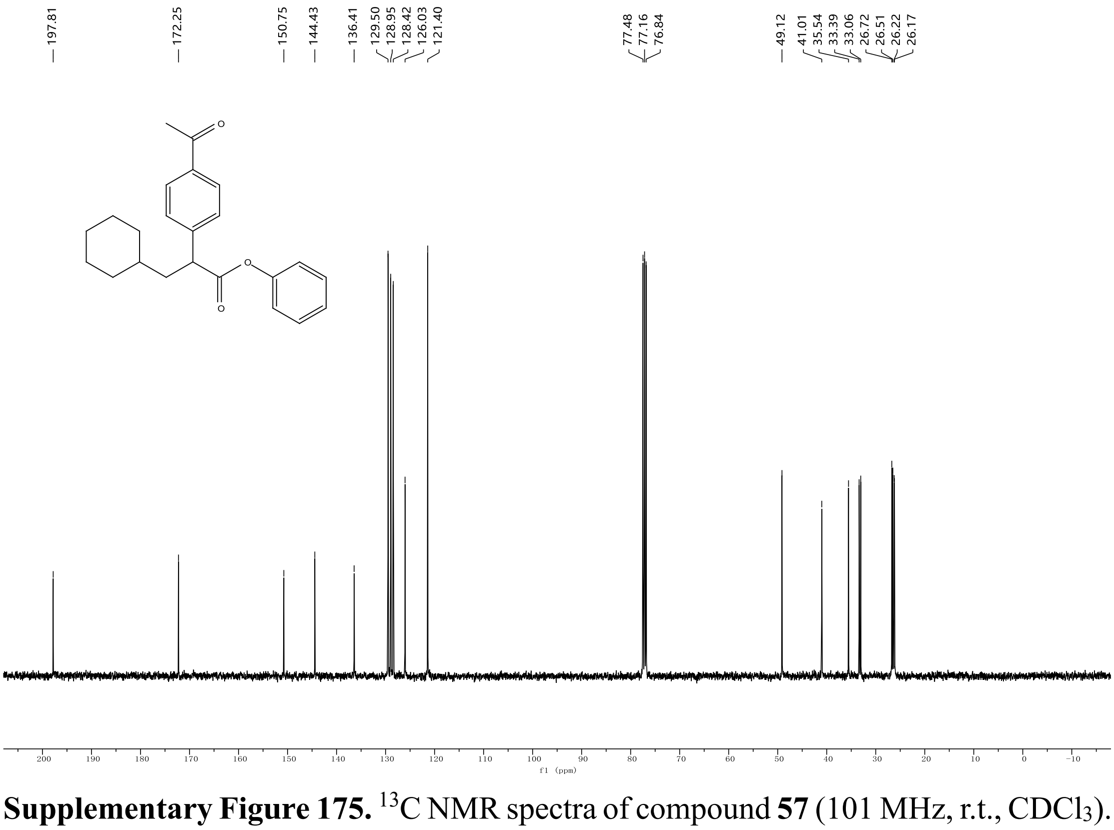

Supplementary Figure 175. ^{13}C NMR spectra of compound **57** (101 MHz, r.t., $CDCl_3$).

Compound 58

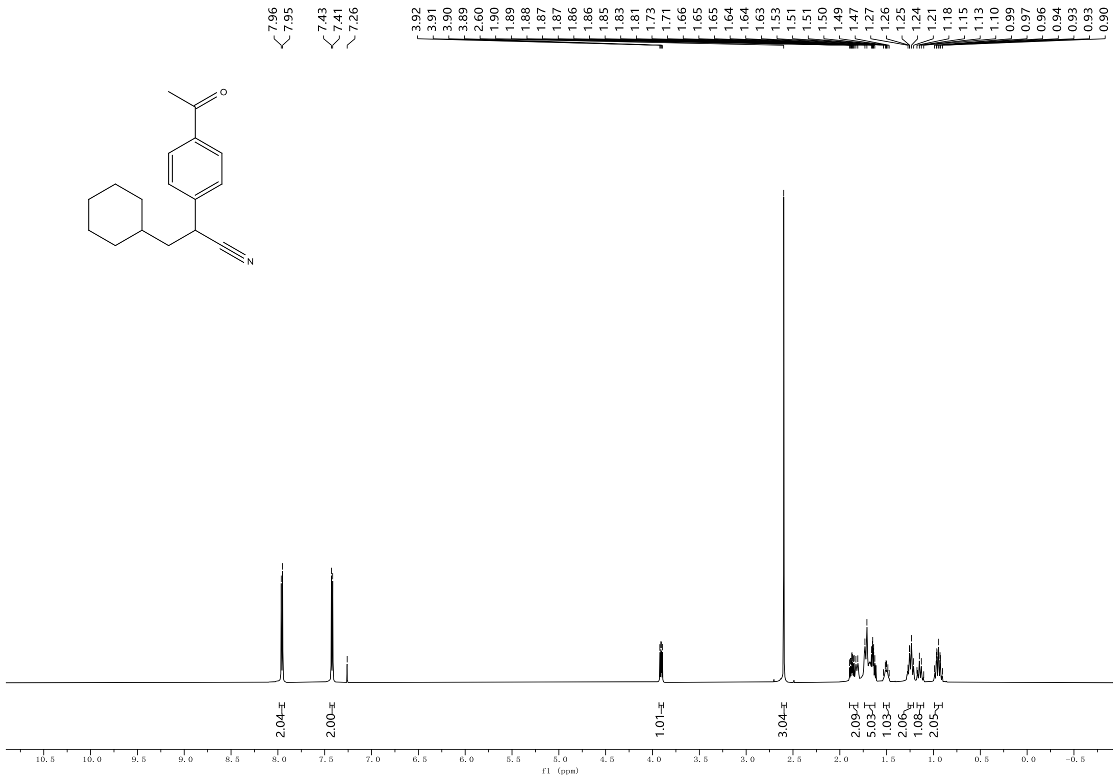

Supplementary Figure 176. ^{1}H NMR spectra of compound **58** (600 MHz, r.t., $CDCl_3$).

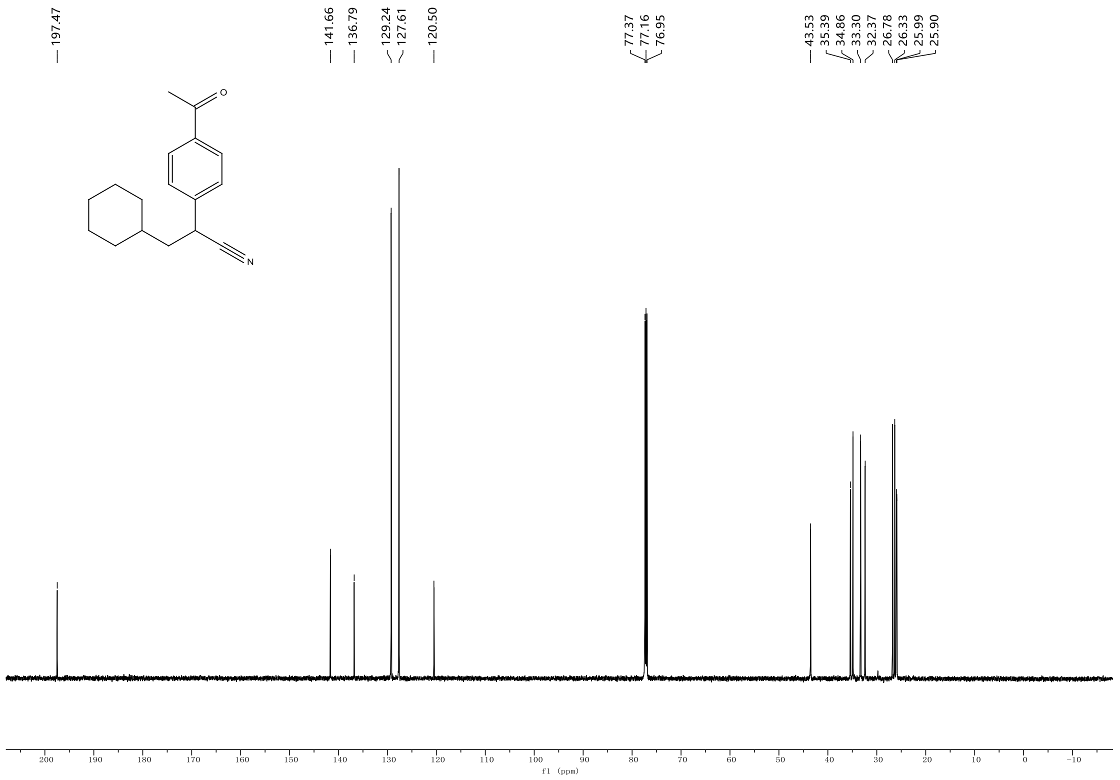

Supplementary Figure 177. ^{13}C NMR spectra of compound **68** (151 MHz, r.t., $CDCl_3$).

Compound 59

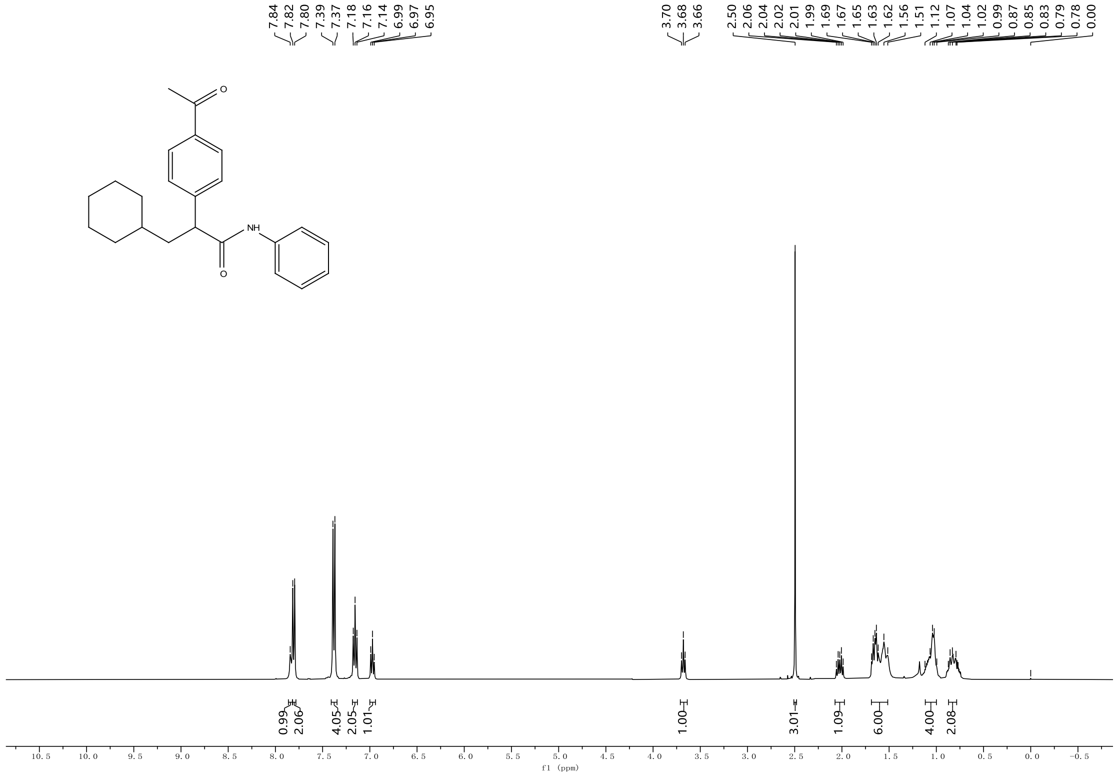

Supplementary Figure 178. ^{1}H NMR spectra of compound **59** (400 MHz, r.t., $CDCl_3$).

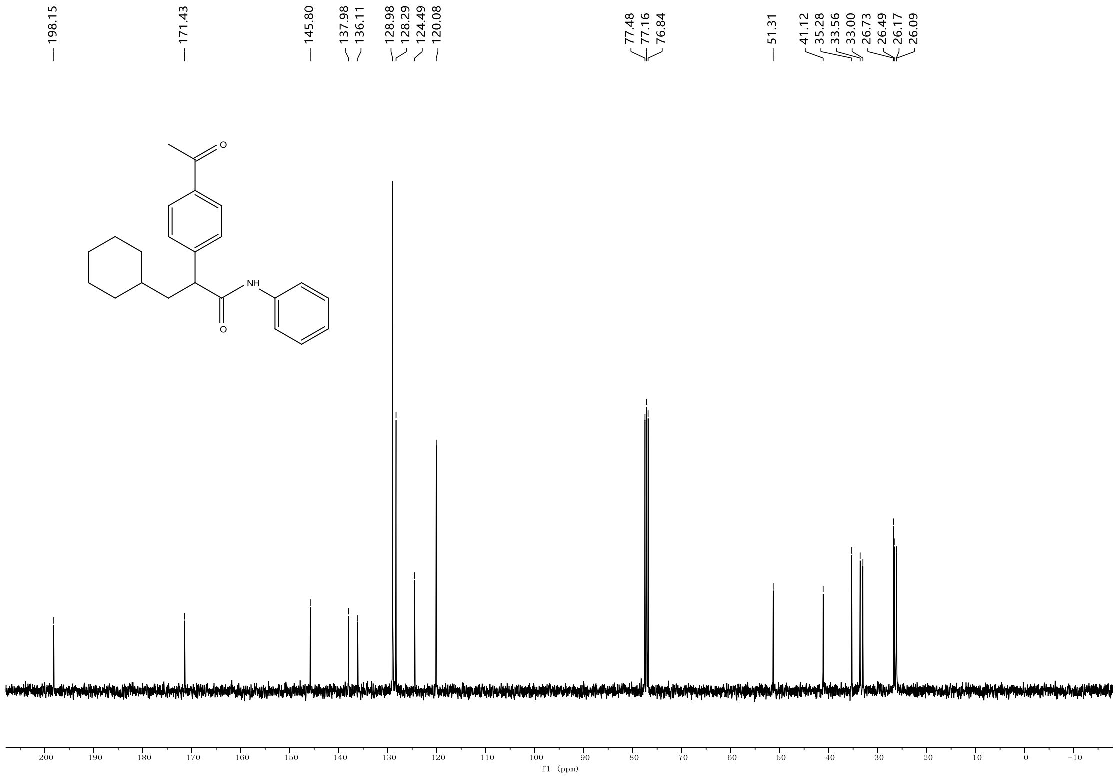

Supplementary Figure 179. ^{13}C NMR spectra of compound **59** (101 MHz, r.t., $CDCl_3$).

Compound 60

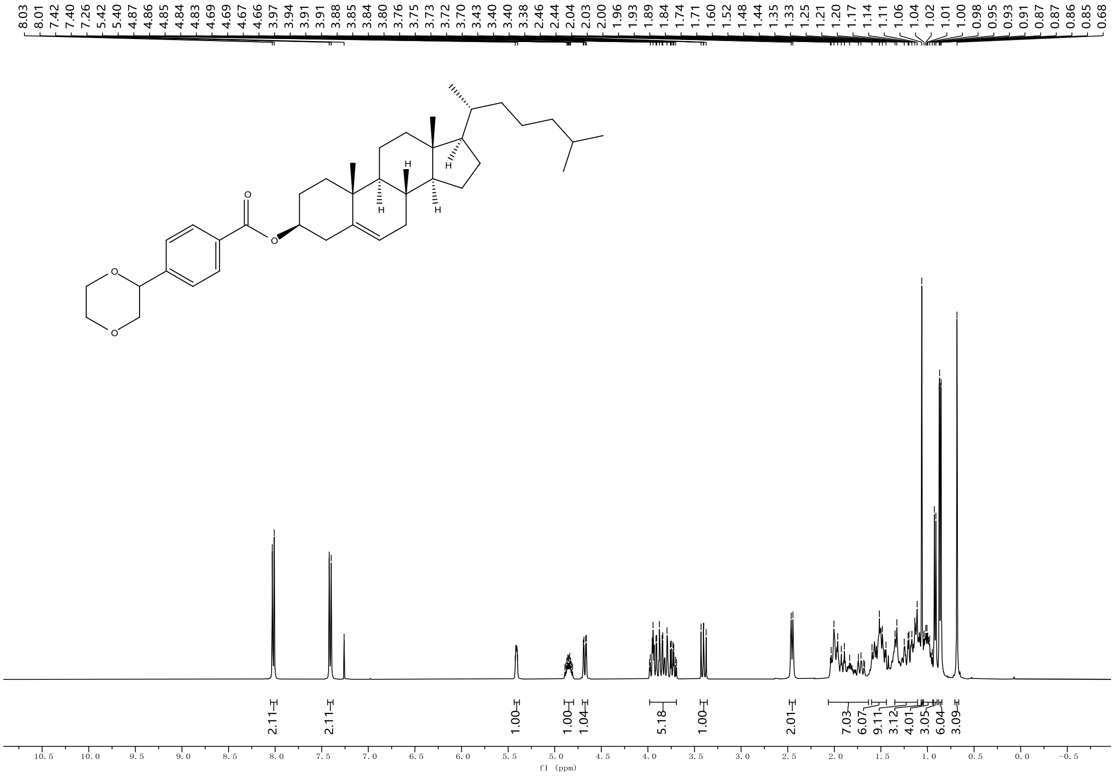

Supplementary Figure 180. ^{1}H NMR spectra of compound **60** (400 MHz, r.t., $CDCl_3$).

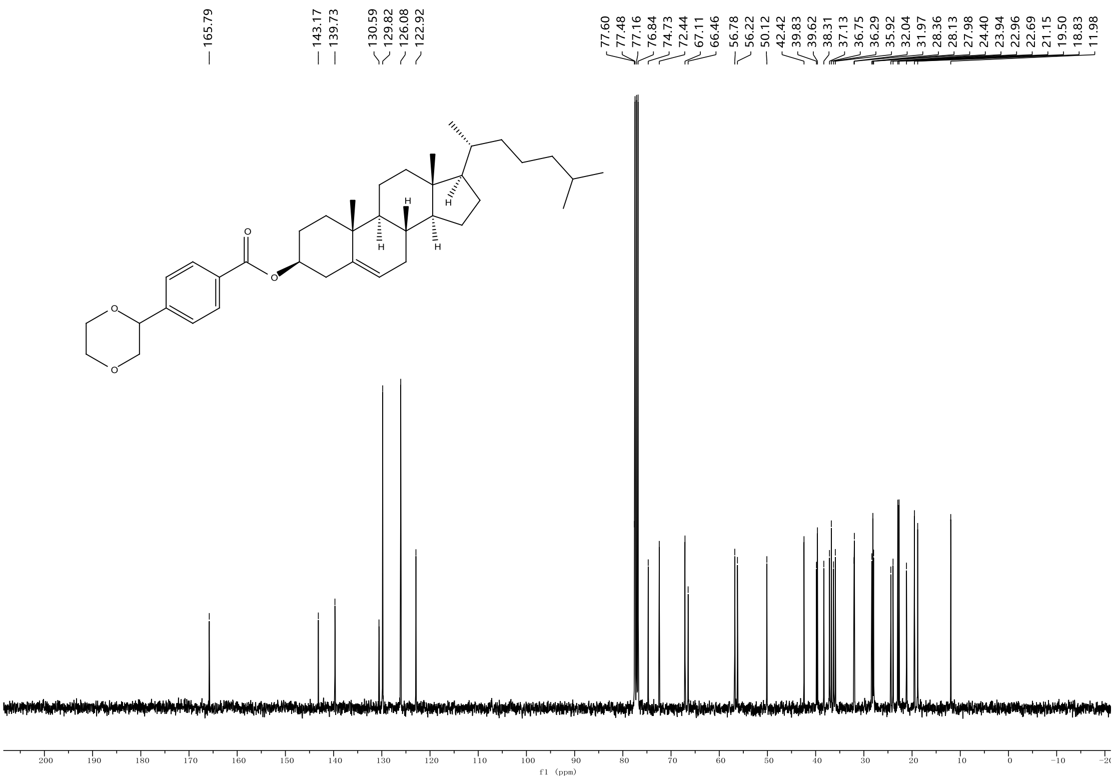

Supplementary Figure 181. ^{13}C NMR spectra of compound **60** (101 MHz, r.t., $CDCl_3$).

Compound 61

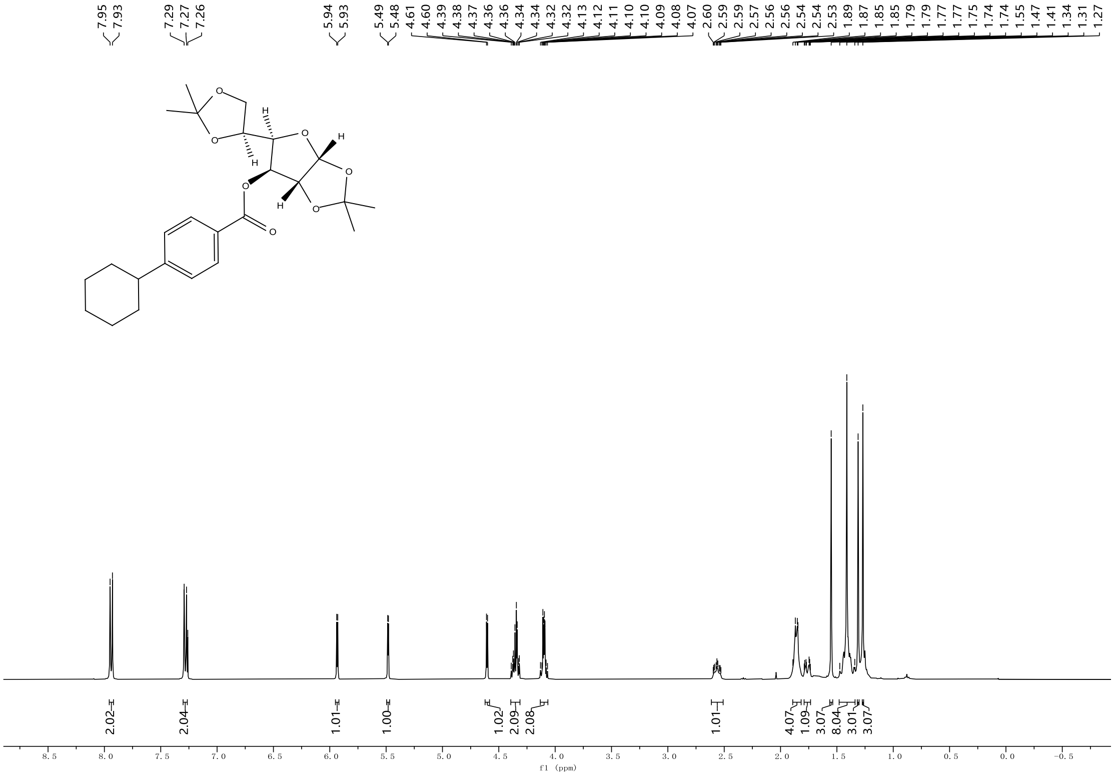

Supplementary Figure 182. ^{1}H NMR spectra of compound **61** (400 MHz, r.t., $CDCl_3$).

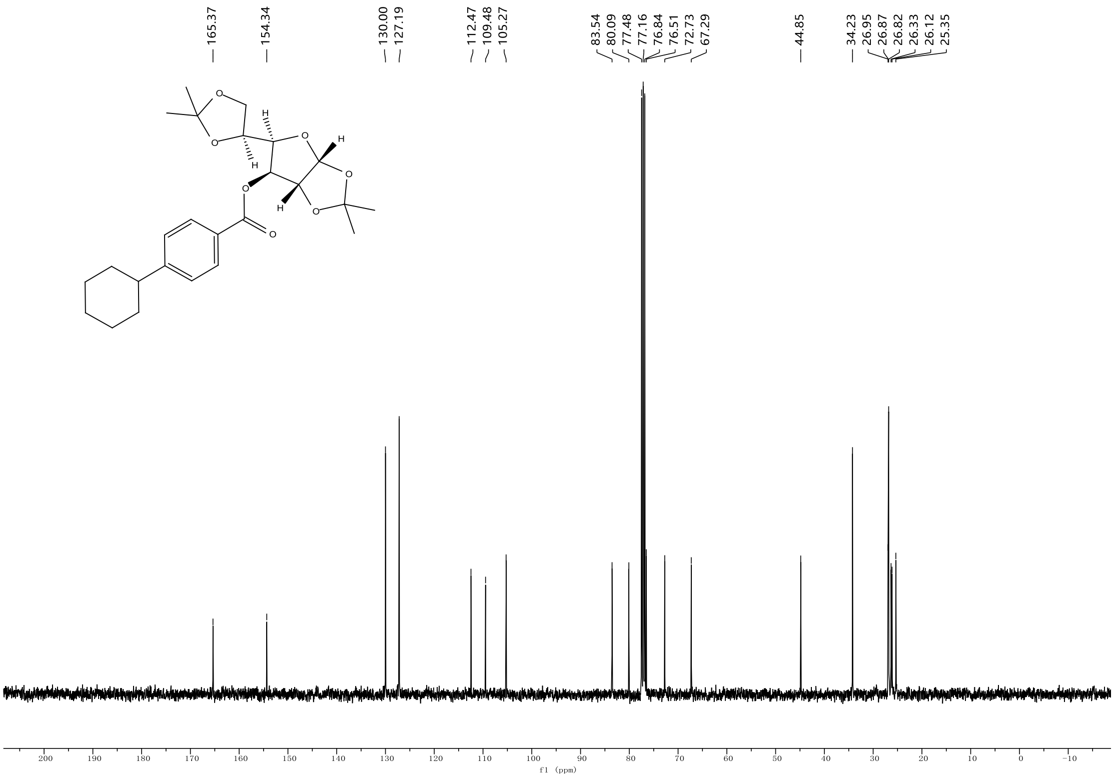

Supplementary Figure 183. ^{13}C NMR spectra of compound **61** (101 MHz, r.t., $CDCl_3$).

Compound 62

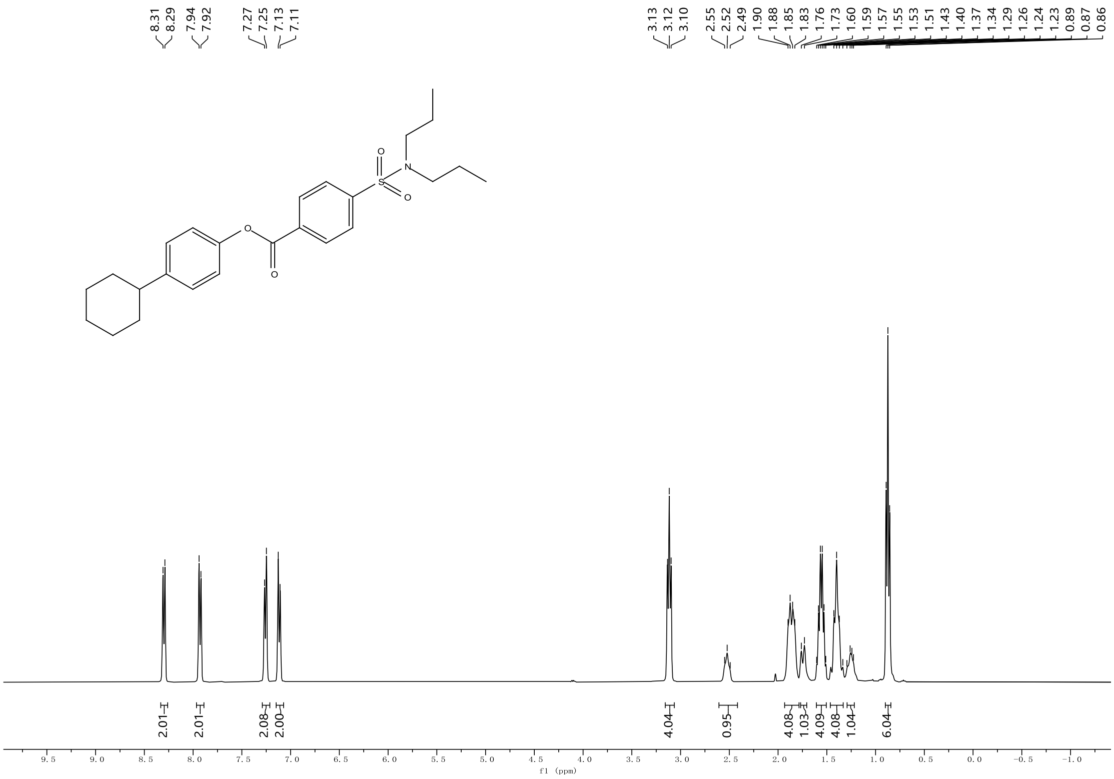

Supplementary Figure 184. ^{1}H NMR spectra of compound **62** (400 MHz, r.t., $CDCl_3$).

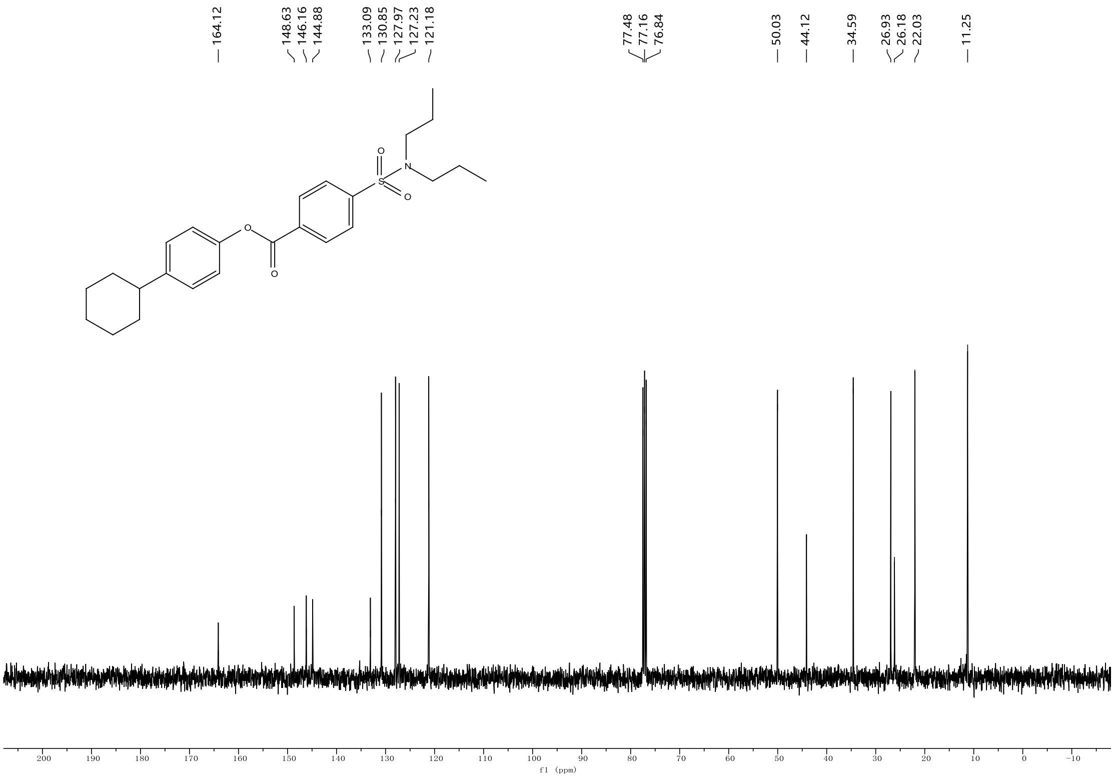

Supplementary Figure 185. ^{13}C NMR spectra of compound **62** (101 MHz, r.t., $CDCl_3$).

Compound 63

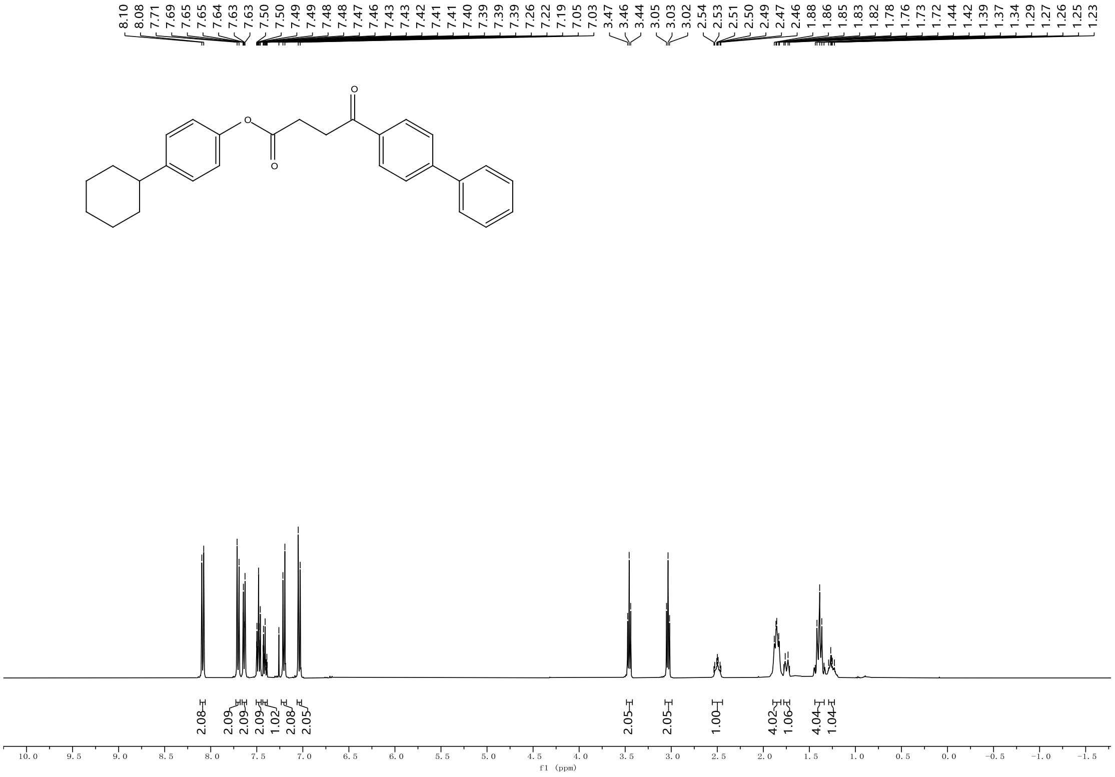

Supplementary Figure 186. ^{1}H NMR spectra of compound **63** (400 MHz, r.t., $CDCl_3$).

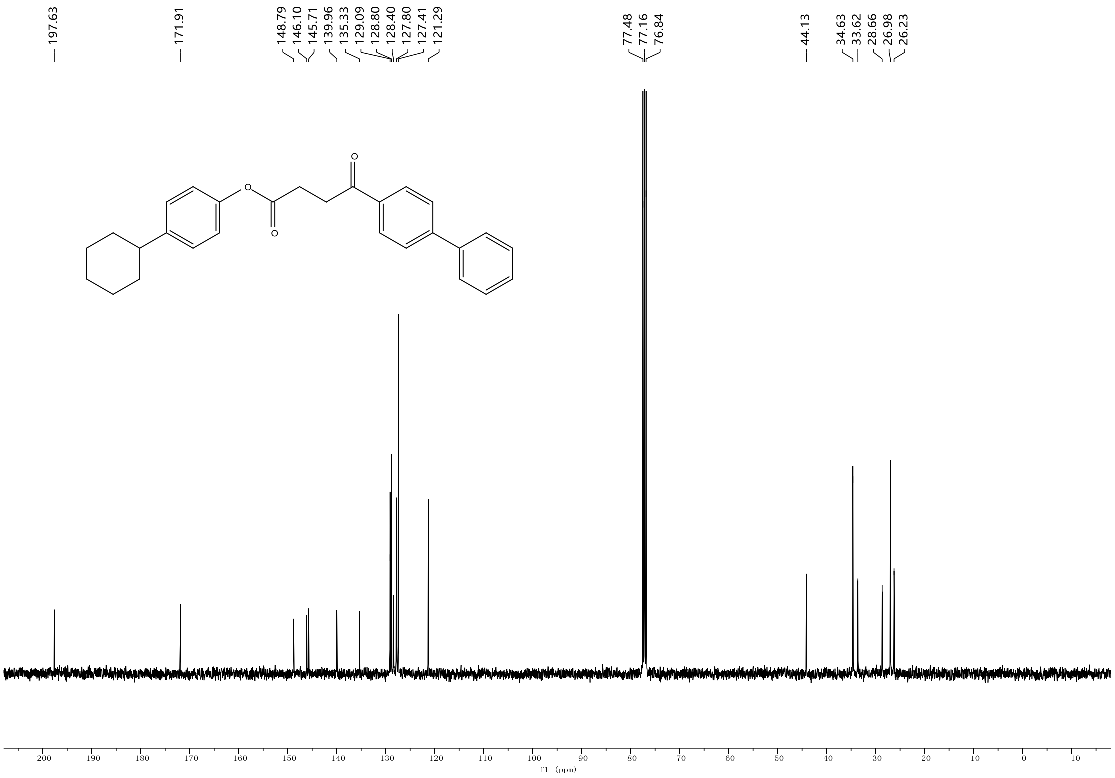

Supplementary Figure 187. ^{13}C NMR spectra of compound **63** (101 MHz, r.t., $CDCl_3$).

Compound 64

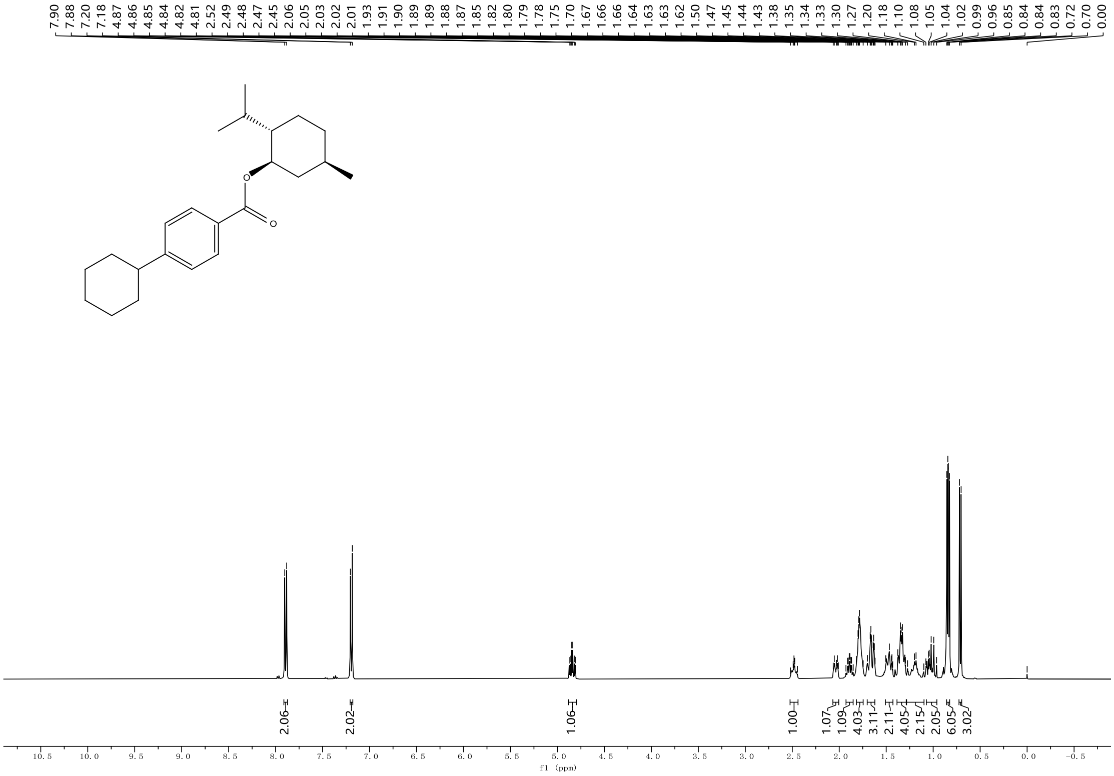

Supplementary Figure 188. ^{1}H NMR spectra of compound **64** (400 MHz, r.t., $CDCl_3$).

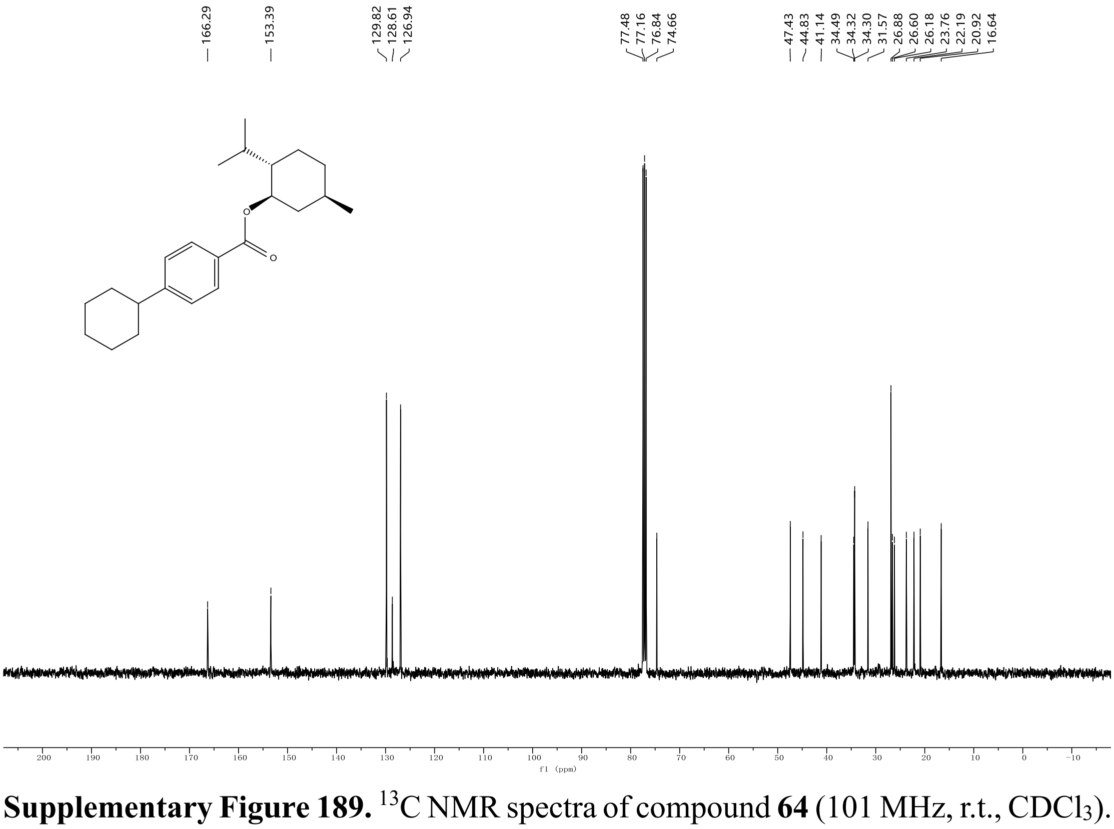

Supplementary Figure 189. ^{13}C NMR spectra of compound **64** (101 MHz, r.t., $CDCl_3$).

Compound 65

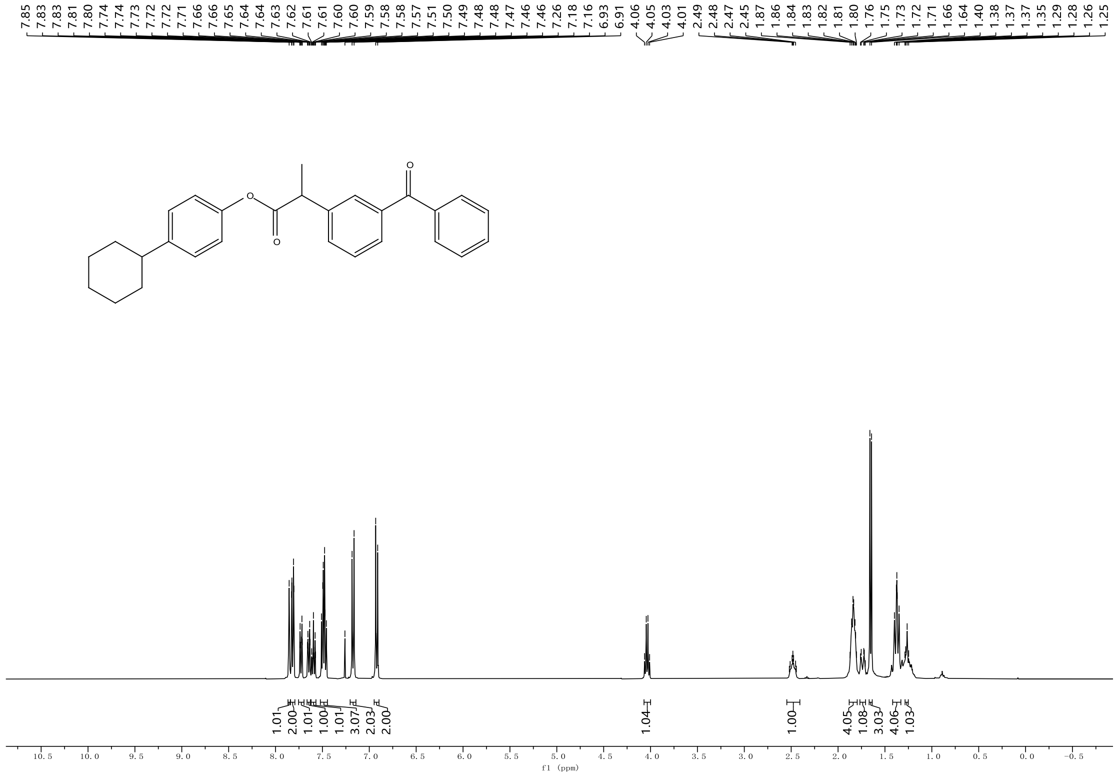

Supplementary Figure 190. ^{1}H NMR spectra of compound **65** (400 MHz, r.t., $CDCl_3$).

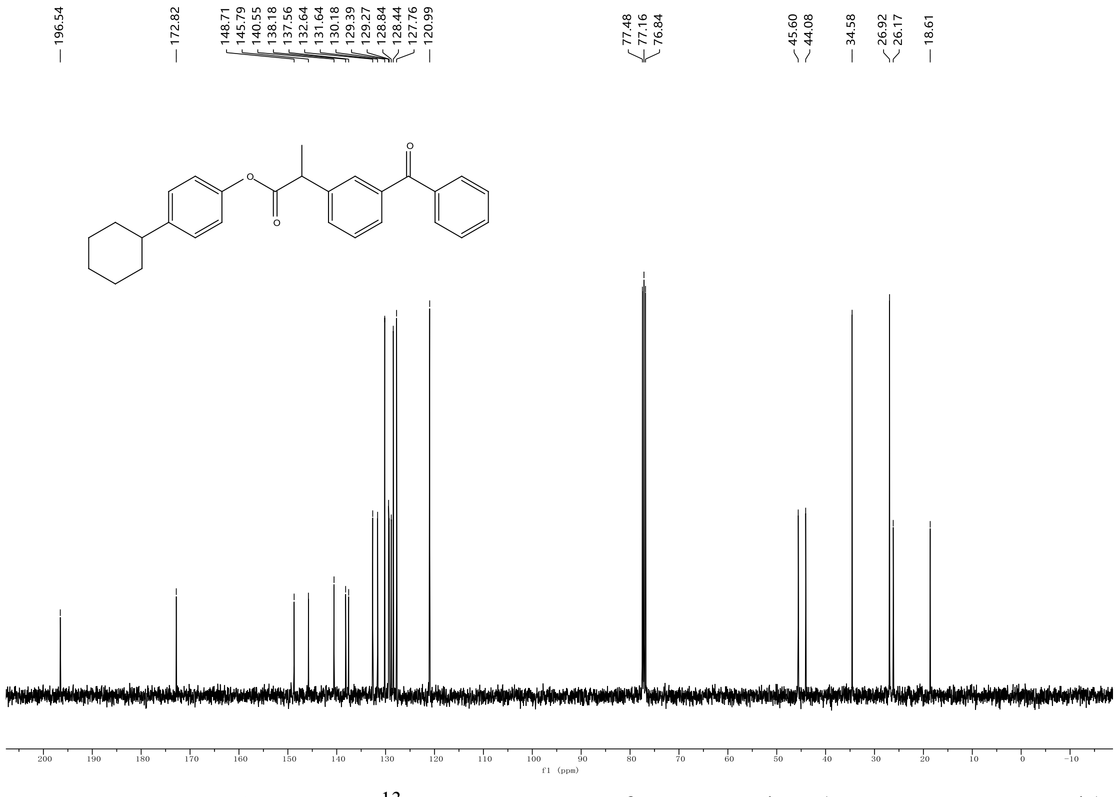

Supplementary Figure 191. ^{13}C NMR spectra of compound **65** (101 MHz, r.t., $CDCl_3$).

Compound 66

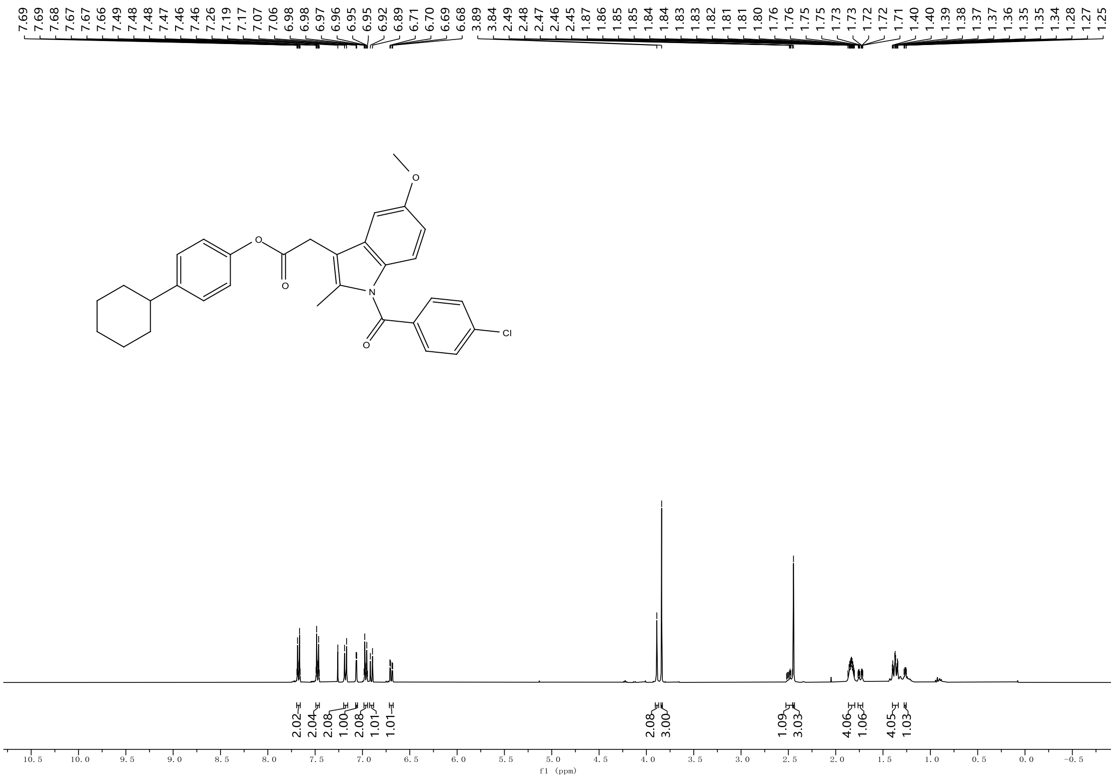

Supplementary Figure 192. ^{1}H NMR spectra of compound **66** (400 MHz, r.t., $CDCl_3$).

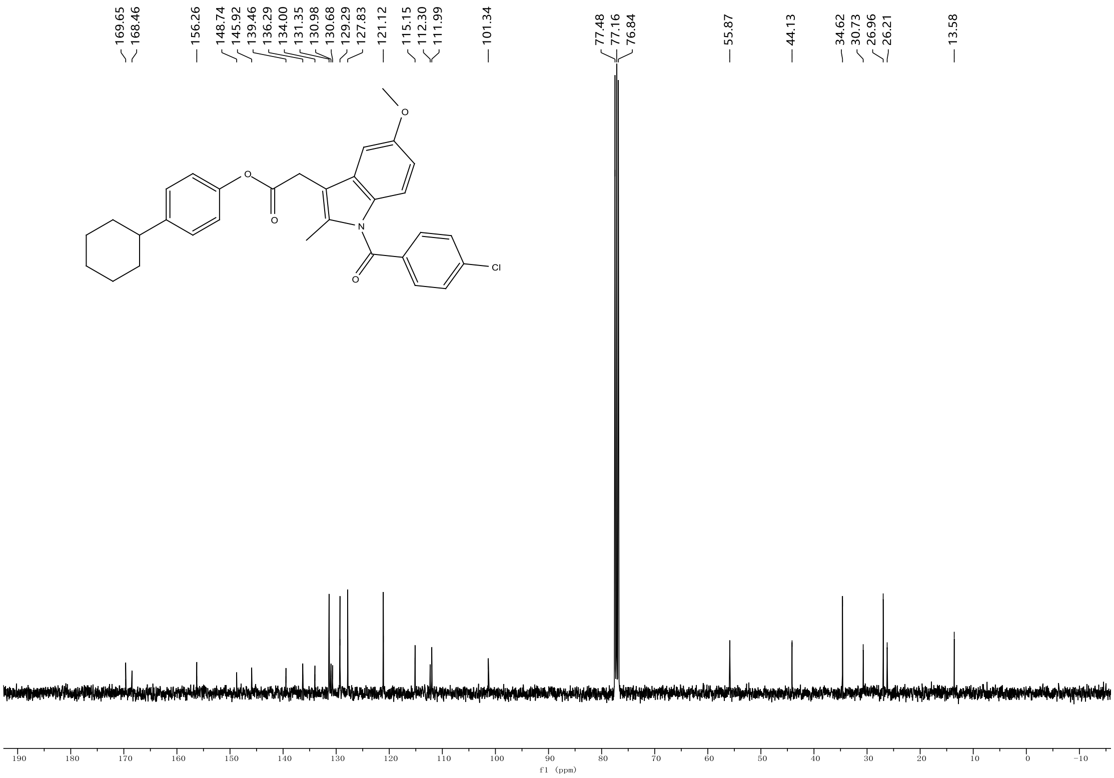

Supplementary Figure 193. ^{13}C NMR spectra of compound **66** (101 MHz, r.t., $CDCl_3$).

Compound 67

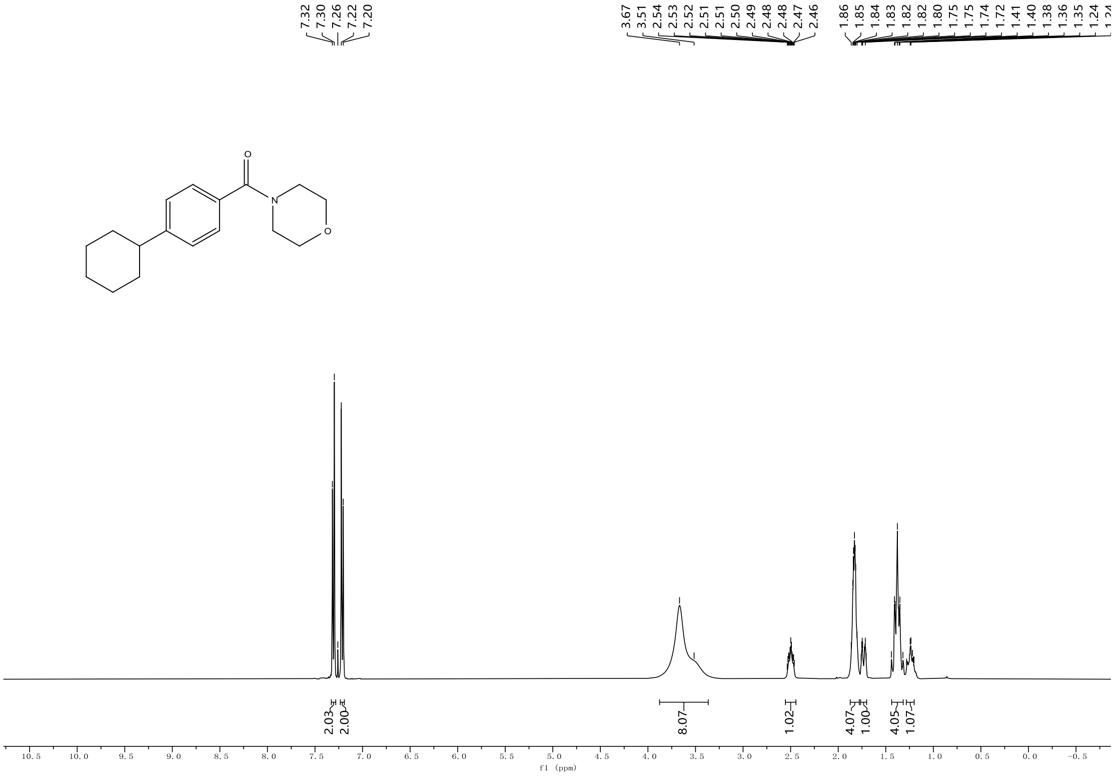

Supplementary Figure 194. ^{1}H NMR spectra of compound **67** (400 MHz, r.t., $CDCl_3$).

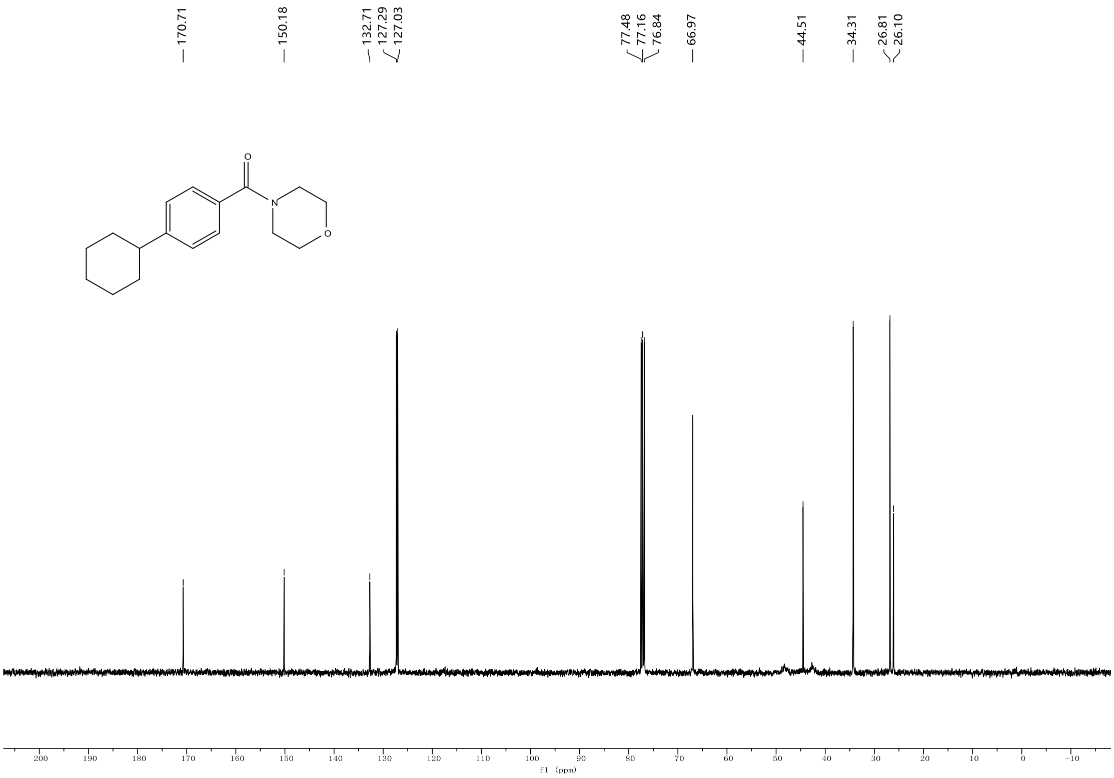

Supplementary Figure 195. ^{13}C NMR spectra of compound **67** (101 MHz, r.t., $CDCl_3$).

Compound 68

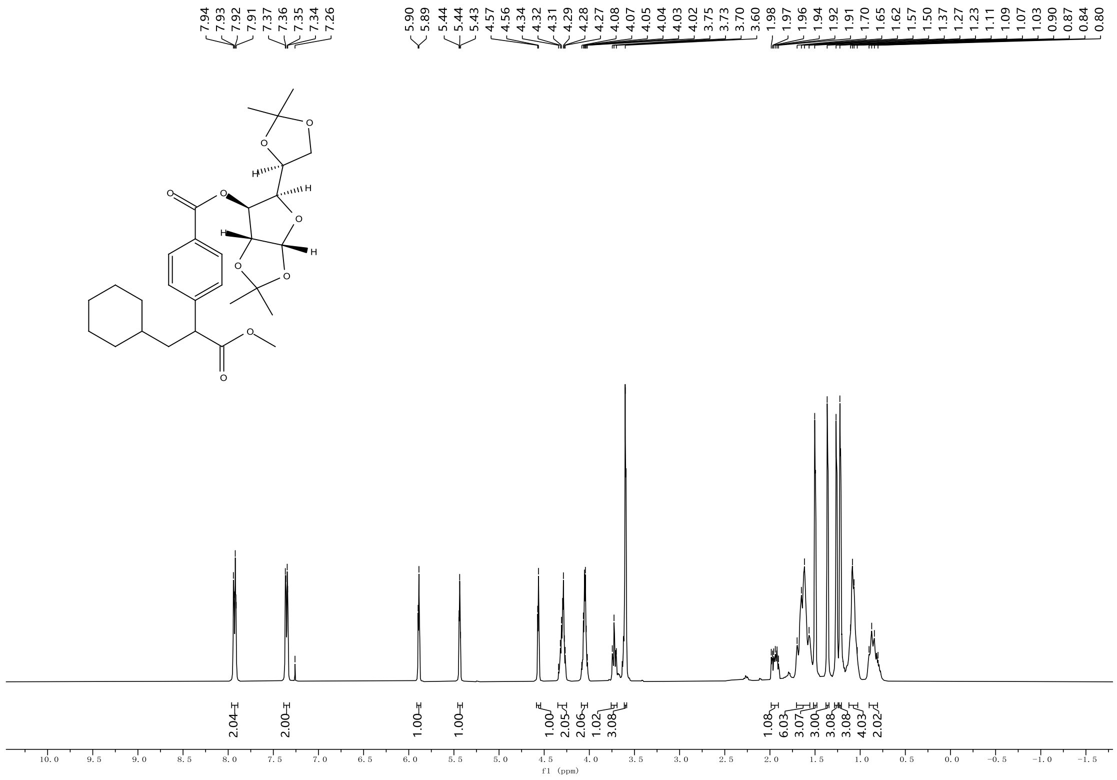

Supplementary Figure 196. ^{1}H NMR spectra of compound **68** (400 MHz, r.t., $CDCl_3$).

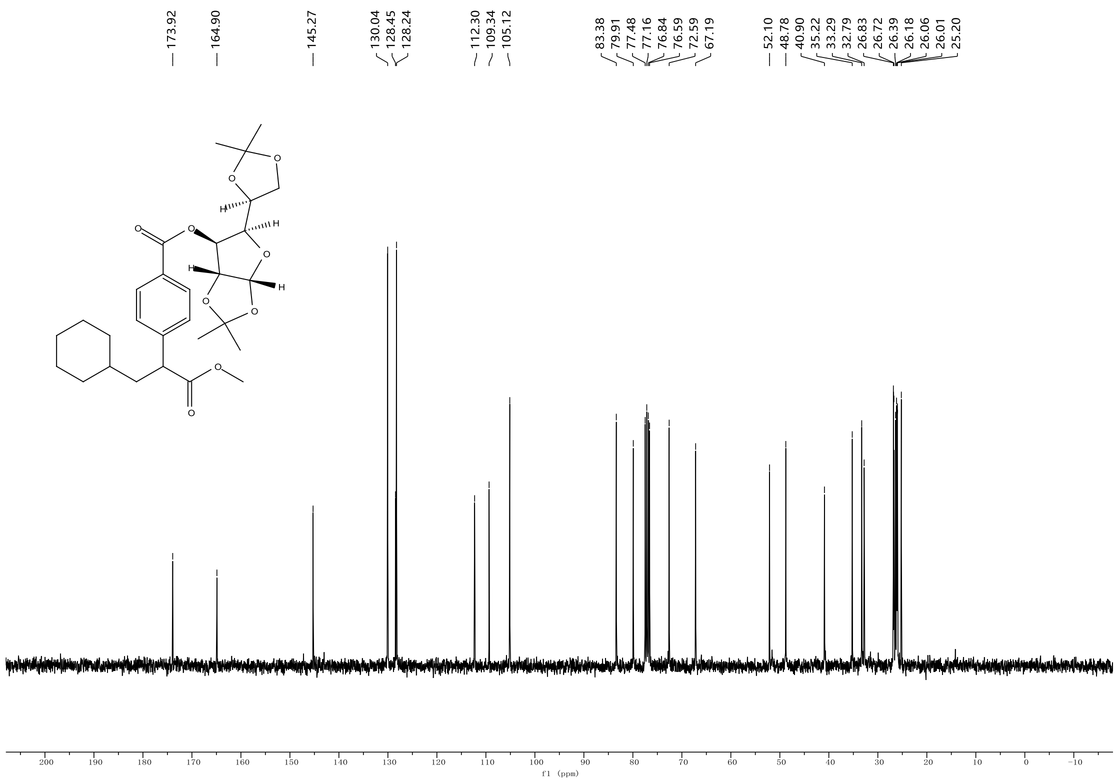

Supplementary Figure 197. ^{13}C NMR spectra of compound **68** (101 MHz, r.t., $CDCl_3$).

Compound 69

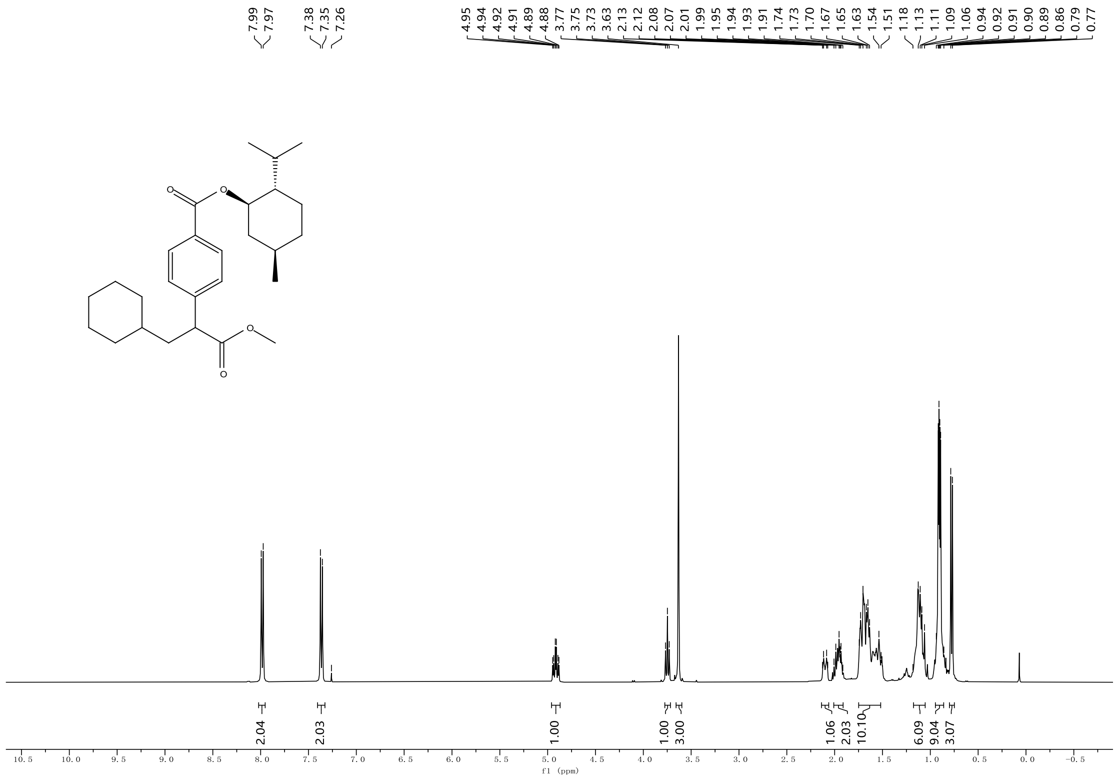

Supplementary Figure 198. ^{1}H NMR spectra of compound **69** (400 MHz, r.t., $CDCl_3$).

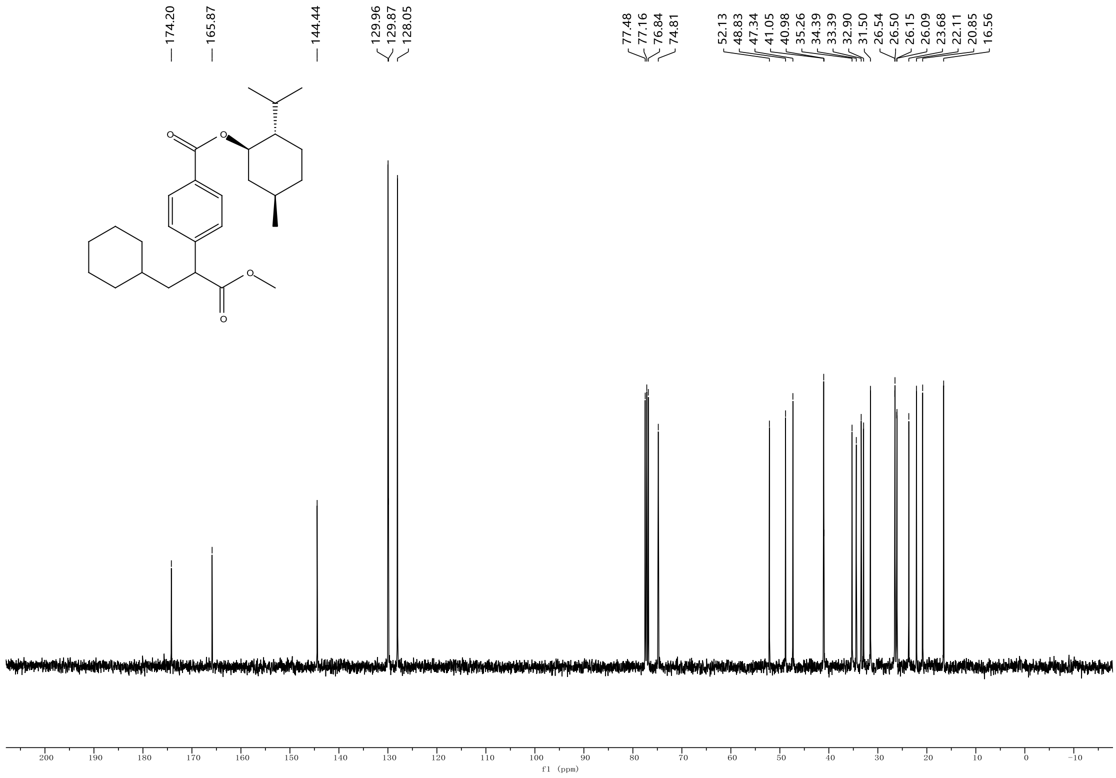

Supplementary Figure 199. ^{13}C NMR spectra of compound **69** (101 MHz, r.t., $CDCl_3$).

Compound 70

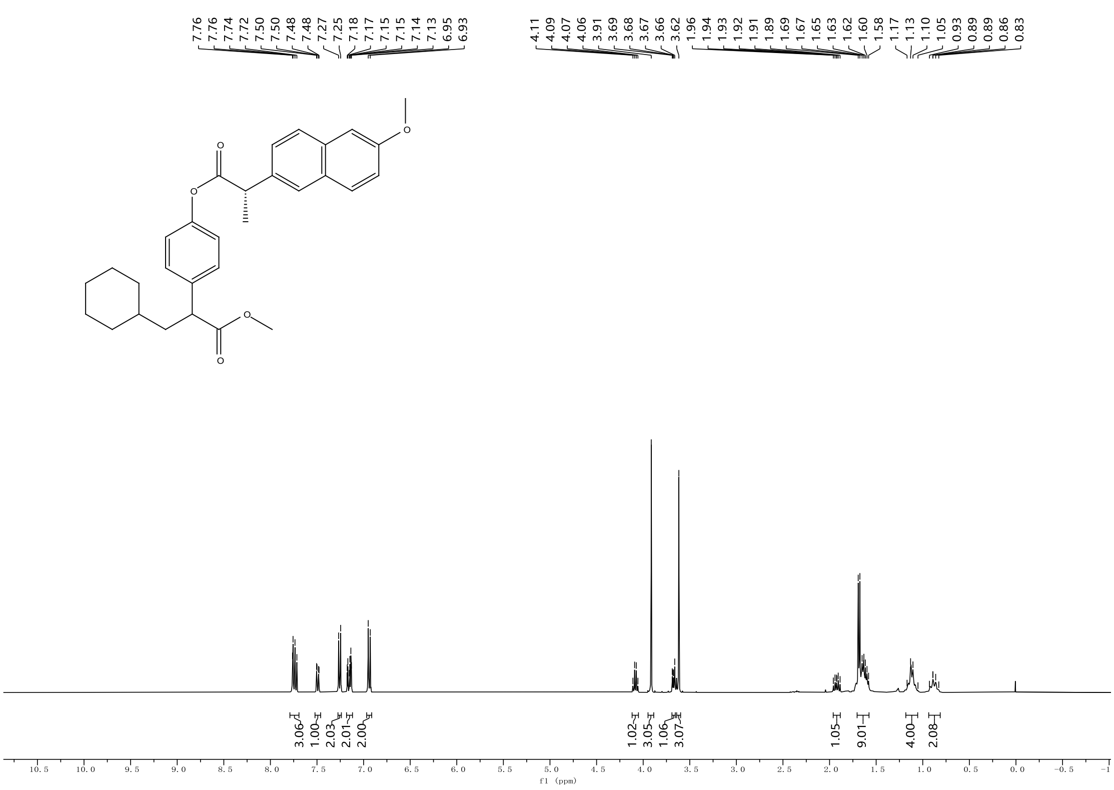

Supplementary Figure 200. ^{1}H NMR spectra of compound **70** (400 MHz, r.t., $CDCl_3$).

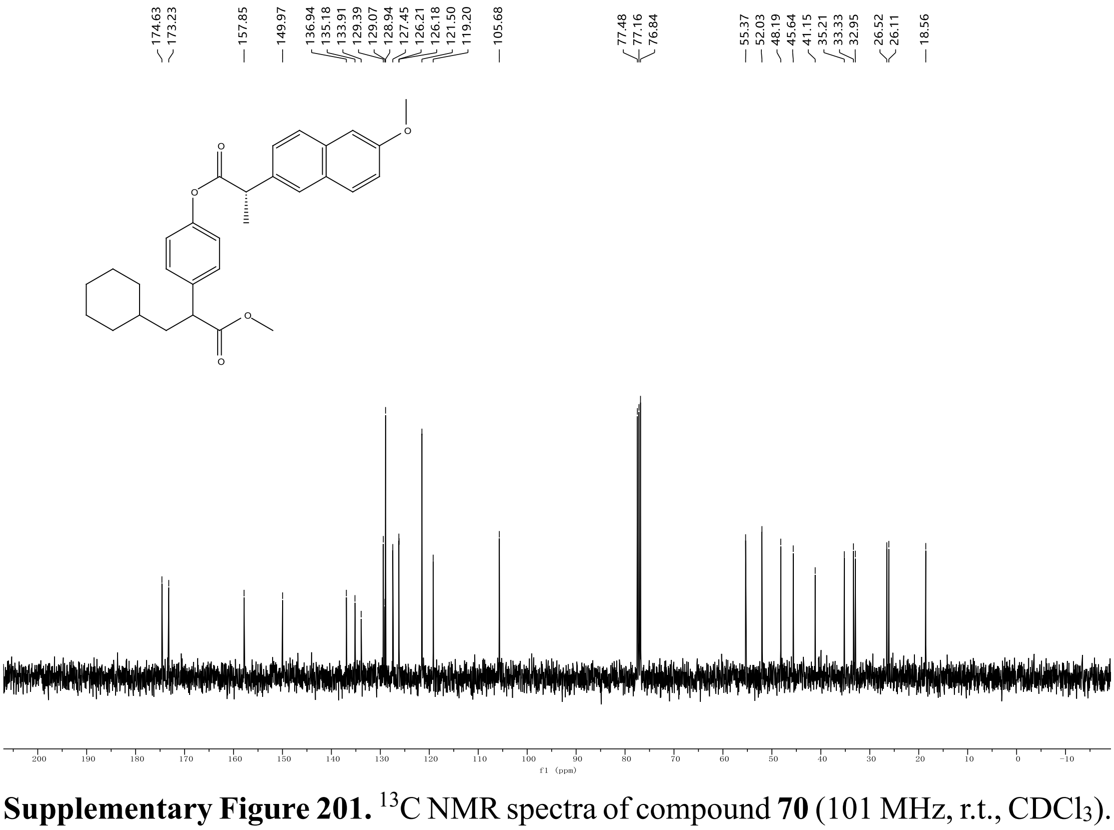

Supplementary Figure 201. ^{13}C NMR spectra of compound **70** (101 MHz, r.t., $CDCl_3$).

Compound 71

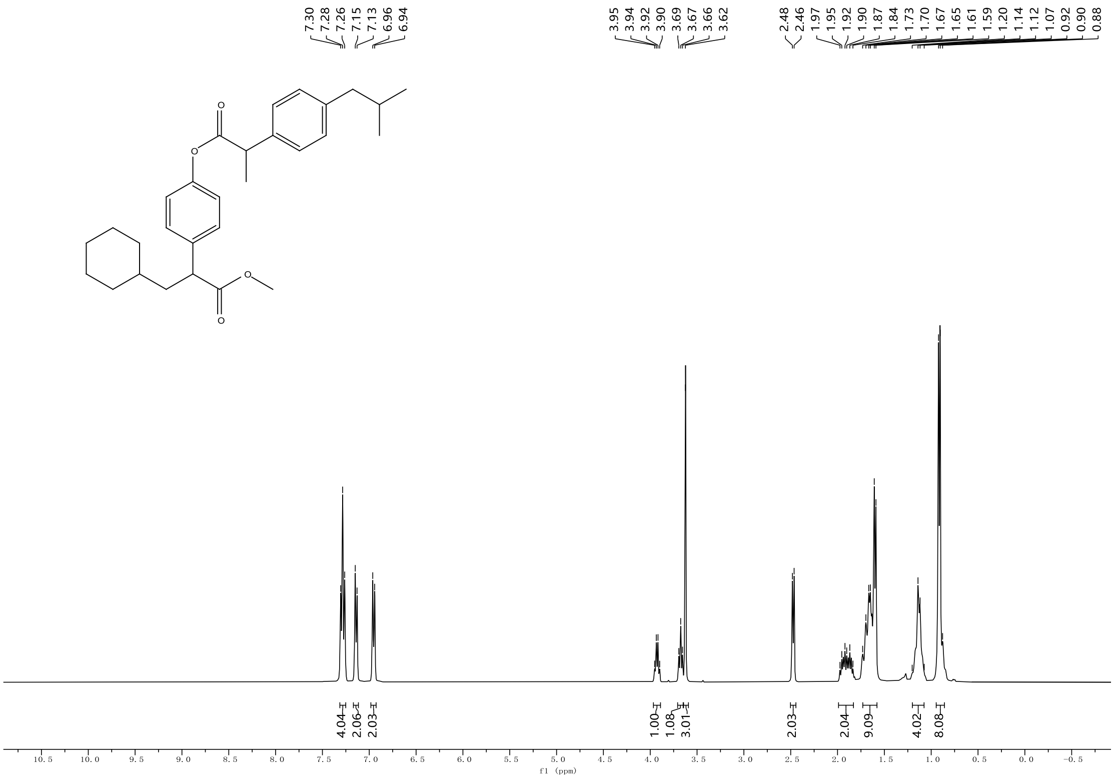

Supplementary Figure 202. ^{1}H NMR spectra of compound **71** (400 MHz, r.t., $CDCl_3$).

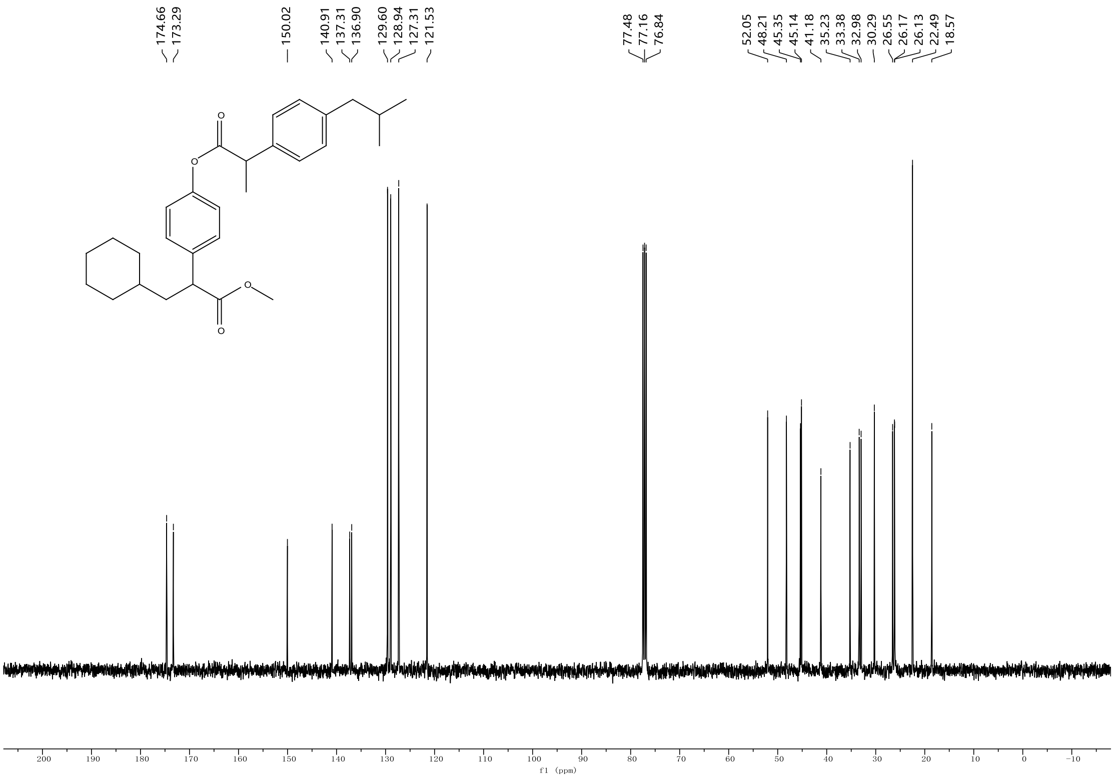

Supplementary Figure 203. ^{13}C NMR spectra of compound **71** (101 MHz, r.t., $CDCl_3$).

4 Supplementary References

[1] Luo, J.; Hu, B.; Wu, W.-D.; Hu, M.-W.; Liu, T.-L. Nickel catalyzed electrochemical C(sp^3)−C(sp^2) cross-coupling reactions of benzyl trifluoroborate and organic halide, *Angew. Chem., Int. Ed.,* **2021**, *60,* 6107.

[2] Shen, Y.-Y.; Gu, W.-T.; Martin, R. sp^3 C−H arylation and alkylation enabled by the synergy of triplet excited ketones and nickel catalysts, *J. Am. Chem. Soc.,* **2018**, *140,* 12200.

[3] Takemiya, A.; Hartwig, J. F. Palladium-catalyzed synthesis of aryl ketones by coupling of aryl bromides with an acyl anion equivalent, *J. Am. Chem. Soc.,* **2006**, *128,* 14800.

[4] Takahashi, M.; Hirota, I.; Nakano, T.; Kotani, T.; Takani, D.; Shiratori, K.; Choi, Y.; Haba, M.; Hosokawa, M. Effects of steric hindrance and electron density of ester prodrugs on controlling the metabolic activation by human carboxylesterase, *Drug Metabol. Pharmacokinet.,* **2021**, *38,* 100391.

[5] Kwon, E.-M.; Kim, C.-G.; Goh, A.-R.; Park, J.; Jun, J.-G. Preparation of benzoyloxy benzophenone derivatives and their inhibitory effects of ICAM-1 expression, *Bull. Korean. Chem. Soc.,* **2012**, *33,* 1939.

[6] Wang, C.; Lei, Y.-J.; Guo, M.-Z.; Shang, Q.-Y.; Liu, H.; Xu, Z.-Q.; Wang, R. Photoinduced, copper-promoted regio- and stereoselective decarboxylative alkylation of *α,β*-unsaturated acids with alkyl iodides, *Org. Lett.,* **2017**, *19,* 6412.

[7] Chen, Y.-G.; Wang, X.; He, X.; An, A.; Zuo, Z.-W. Photocatalytic dehydroxymethylative arylation by synergistic cerium and nickel catalysis, *J. Am. Chem. Soc.,* **2021**, *143,* 4896.

[8] Jia, X.-G.; Guo, P.; Duan, J.-C.; Shu, X.-Z. Dual nickel and lewis acid catalysis for cross-electrophile coupling: the allylation of aryl halides with allylic alcohols. *Chem. Sci.,* **2018**, *9*, 640.

[9] Toriyama, F.; Cornella, J.; Wimmer, L.; Chen, T.-G.; Dixon, D. D.; Creech, G.; Baran, P. S. Redox active esters in Fe-catalyzed C-C coupling, *J. Am. Chem. Soc.,* **2016**, *138,* 11132.

[10] Martin-Montero, R.; Yatham, V.-R.; Yin, H.-F.; Davies, J.; Martin, R. Ni-catalyzed reductive deaminative arylation at sp^3 carbon centers. *Org. Lett.,* **2019**, *21,* 2947.

[11] González-Bobes, F.; Fu, G. C. Amino alcohols as ligands for nickel-catalyzed suzuki reactions of unactivated alkyl halides, including secondary alkyl chlorides, with arylboronic acids. *J. Am. Chem. Soc.,* **2006**, *128,* 5360.

[12] Xu, S.; Chen, H.-R.; Zhou, Z.-J.; Kong, W.-Q. Three-component alkene difunctionalization by direct and selective activation of aliphatic C−H bonds, *Angew. Chem., Int. Ed.,* **2021**, *60,* 7405.

[13] Bisz, E.; Szostak, M. Iron-catalyzed $C(sp^2)-C(sp^3)$ cross-coupling of chlorobenzamides with alkyl grignard reagents: development of catalyst system, synthetic scope, and application, *Adv. Synth. Catal.,* **2019**, *361,* 85.

[14] Perry, I. B.; Brewer, T. F.; Sarver, P. J.; Schultz, D. M.; DiRocco, D. A.; MacMillan, D. A. C. Direct arylation of strong aliphatic C-H bonds. *Nature,* **2018**, *560,* 70.

[15] Agrawal. T.; Cook, S. P. Iron-Catalyzed Cross-Coupling Reactions of Alkyl Grignards with Aryl Sulfamates and Tosylates, *Org. Lett.,* **2013**, *15,* 96.

[16] Liu. D.; Li, Y.-X.; Qi, X.-T.; Liu. C.; Lan. Y.; Lei, A. W. Nickel-catalyzed selective oxidative radical cross-coupling: an effective strategy for inert Csp^3–H functionalization, *Org. Lett.,* **2015**, *17,* 998.

[17] Zhu. D.; Shi, L. Ni-catalyzed cross-coupling of aryl thioethers with alkyl grignard reagents via C–S bond cleavage, *Chem. Commun.,* **2018**, *54,* 9313.

[18] Guo, X.-Y.; Li, C. J. Ruthenium-catalyzed para-selective oxidative cross-coupling of arenes and cycloalkanes, *Org. Lett.,* **2011**, *13,* 4977.
